# Lead Optimization of Benzoxazolone Carboxamides as Orally Bioavailable and CNS Penetrant Acid Ceramidase Inhibitors

**DOI:** 10.1021/acs.jmedchem.9b02004

**Published:** 2020-03-16

**Authors:** Simona Di Martino, Piero Tardia, Vincenzo Cilibrasi, Samantha Caputo, Marco Mazzonna, Debora Russo, Ilaria Penna, Natalia Realini, Natasha Margaroli, Marco Migliore, Daniela Pizzirani, Giuliana Ottonello, Sine Mandrup Bertozzi, Andrea Armirotti, Duc Nguyen, Ying Sun, Ernesto R. Bongarzone, Peter Lansbury, Min Liu, Renato Skerlj, Rita Scarpelli

**Affiliations:** ^‡^Drug Discovery and Development (D3)-Validation, ^§^D3-Pharma Chemistry, ^∥^Analytical Chemistry Lab, Fondazione Istituto Italiano di Tecnologia, Via Morego 30, I-16163 Genova, Italy; ⊥ Lysosomal Therapeutics Inc., 19 Blackstone Street, Cambridge, Massachusetts 02139, United States; # The Myelin Regeneration Group at the Dept. Anatomy & Cell Biology, College of Medicine, University of Illinois at Chicago, Chicago, Illinois, 60612, United States; ∇ The Division of Human Genetics, Cincinnati Children’s Hospital Medical Center, Department of Pediatrics, University of Cincinnati College of Medicine, Cincinnati, Ohio 45229-3039, United States

## Abstract

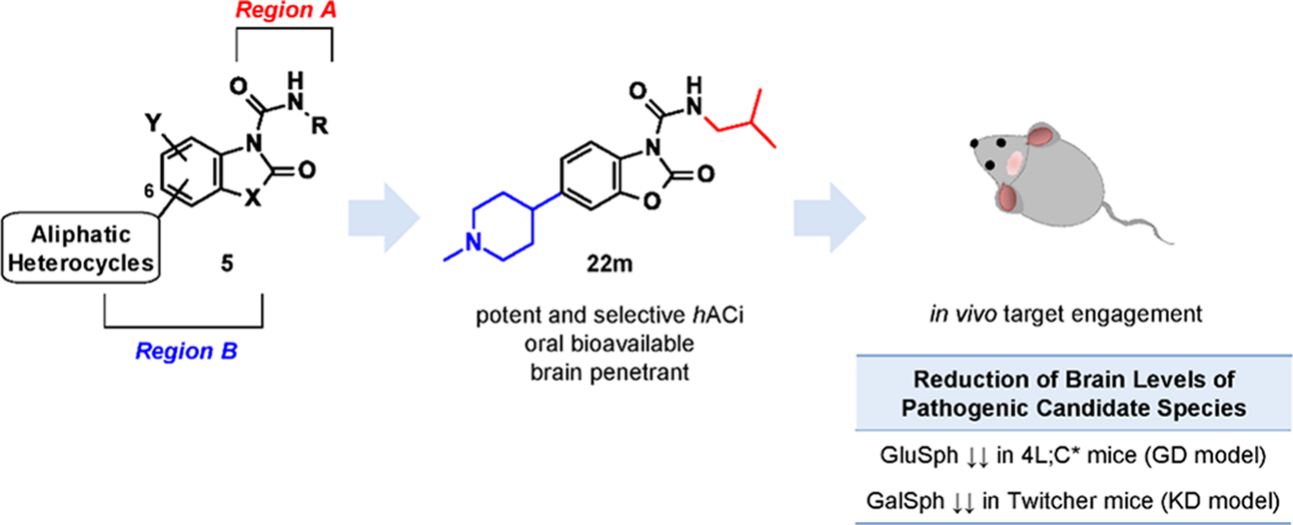

Sphingolipids (SphLs) are a diverse class of molecules that are regulated by a complex network of enzymatic pathways. A disturbance in these pathways leads to lipid accumulation and initiation of several SphL-related disorders. Acid ceramidase is one of the key enzymes that regulate the metabolism of ceramides and glycosphingolipids, which are important members of the SphL family. Herein, we describe the lead optimization studies of benzoxazolone carboxamides resulting in piperidine **22m**, where we demonstrated target engagement in two animal models of neuropathic lysosomal storage diseases (LSDs), Gaucher’s and Krabbe’s diseases. After daily intraperitoneal administration at 90 mg kg^–1^, **22m** significantly reduced the brain levels of the toxic lipids glucosylsphingosine (GluSph) in 4L;C* mice and galactosylsphingosine (GalSph) in Twitcher mice. We believe that **22m** is a lead molecule that can be further developed for the correction of severe neurological LSDs where GluSph or GalSph play a significant role in disease pathogenesis.

## Introduction

Sphingolipids (SphLs) are a large class of diverse amphipathic molecules found in abundance in plasma membranes.^1,2^ Besides being important as structural cellular components, SphLs play a central role in different biological processes, which are essential to maintain the homeostasis and the development of eukaryotic cells. These processes include signaling, angiogenesis, cell growth, proliferation, and death, senescence, inflammation, immune responses, metabolism, autophagy, and brain development and functions.^2^ Aided by recent technological advances, much has been accomplished in terms of the identification of the basic biological components of the complex network in dynamic and interconnected enzymatic pathways that regulate the biosynthesis of SphLs and the formation of a variety of bioactive metabolites in distinct cellular compartments.^1^

In recent years, both academia and industry have shown growing interest in advancing our understanding of the multifaceted roles of SphL species under physiological and pathological conditions.^2^ Collected evidence suggests that a disturbance between the synthesis and catabolism of SphLs leads to their accumulation in specific cellular compartments, such as the lysosomes, and the initiation of several SphL-related disorders. Lysosomes are critical organelles responsible for cellular homeostasis.^3^ They contain different degradative enzymes that can hydrolyze proteins, DNA, RNA, polysaccharides, and lipids.^4^

**Figure 1:**
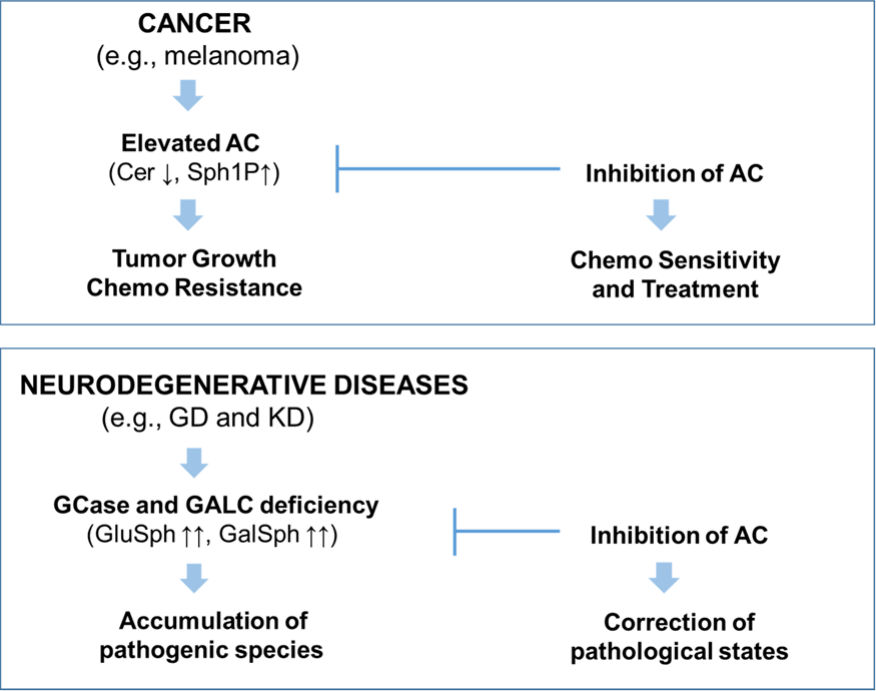
Some potential applications of AC inhibition therapy.

Acid ceramidase (AC, also known as *N*-acylsphingosine amidohydrolase-1, ASAH-1) is a lysosomal cysteine amidase that catalyzes the hydrolysis of ceramides (Cer) into fatty acids and sphingosine, which is then converted into sphingosine 1-phosphate (Sph1P) by sphingosine kinase.^5−7^ Cer and Sph1P are important members of the SphL class and have opposing actions in the control of the cellular fate;^8−10^ while Cer mediates cellular senescence^11^ and apoptosis,^12,13^ Sph1P promotes cell survival and proliferation.^14−17^ Recent studies have shown that AC is abnormally expressed in various types of human cancer (for example, prostate, head and neck, colon, and glioblastoma), and serum AC levels are elevated in patients with melanoma relative to control subjects.^18^ Therefore, inhibition of AC has been envisaged as a potential cancer drug target (Figure 1). Aberrant AC activity has also been described in several other common diseases, including inflammation, pain, and various pulmonary disorders.^19,20^

Over the recent years, the multifaceted catabolic role of AC has attracted much attention for its potential therapeutic applications in many other altered conditions. Important genetic studies have identified specific mutations in several genes that encode defective expressions of some lysosomal enzymes as the causes of the onset and progression of severe pathological conditions, called lysosomal storage diseases (LSDs).^21−24^ For example, Gaucher’s disease (GD) is caused by a defective function of acid β-glucocerebrosidase (GCase), a lysosomal membrane-associated protein responsible for the hydrolysis of glucosylceramide (GluCer) to glucose and ceramides.^25−27^ Krabbe’s disease (KD) is associated with defective β-galactosyl-ceramidase (GALC) activity, a lysosomal enzyme responsible for the hydrolysis of galactosylceramide (GalCer).^28^

As a result of either enzyme absences or deficiencies, these metabolic lysosomal disorders are characterized by an abnormal storage of substrates or metabolites to concentration levels that are toxic or otherwise detrimental to the cells in various compartments, including the skeleton, skin, liver, spleen, lung, heart, and central nervous system (CNS). The substrate or metabolite accumulations are believed to be responsible for the disease progression.^24^ In GD patients, for example, the accumulation of GluCer (3-fold) and/or glucosylsphingosine (GluSph) (200-fold) has been related to the brain pathogenesis of neuronopathic GD patients due to neuronal death, which is propagated by the toxic effects of GluCer and/or GluSph.^29,30^ Recent evidence demonstrates an active role of AC in an alternative catabolic pathway, which causes GluSph accumulation through the deacylation of the lysosomal GluCer.^31,32^ In KD patients, deficiency of GALC activity leads to accumulation of neurotoxic galactosylsphingosine (GalSph or psychosine) in tissues, especially in the brain. It is possible that accumulation of GalSph mediates pathology of KD. A very recent report suggests that genetic ablation of AC or pharmacological inhibition of AC could eliminate psychosine accumulation and prolong the life span of Twitcher mice, a model of KD.^33,34^ No approved therapeutic approaches are available to treat neuropathic GD and KD; inhibiting AC may provide an efficacious strategy for treating these two devastating diseases.

**Figure 2:**
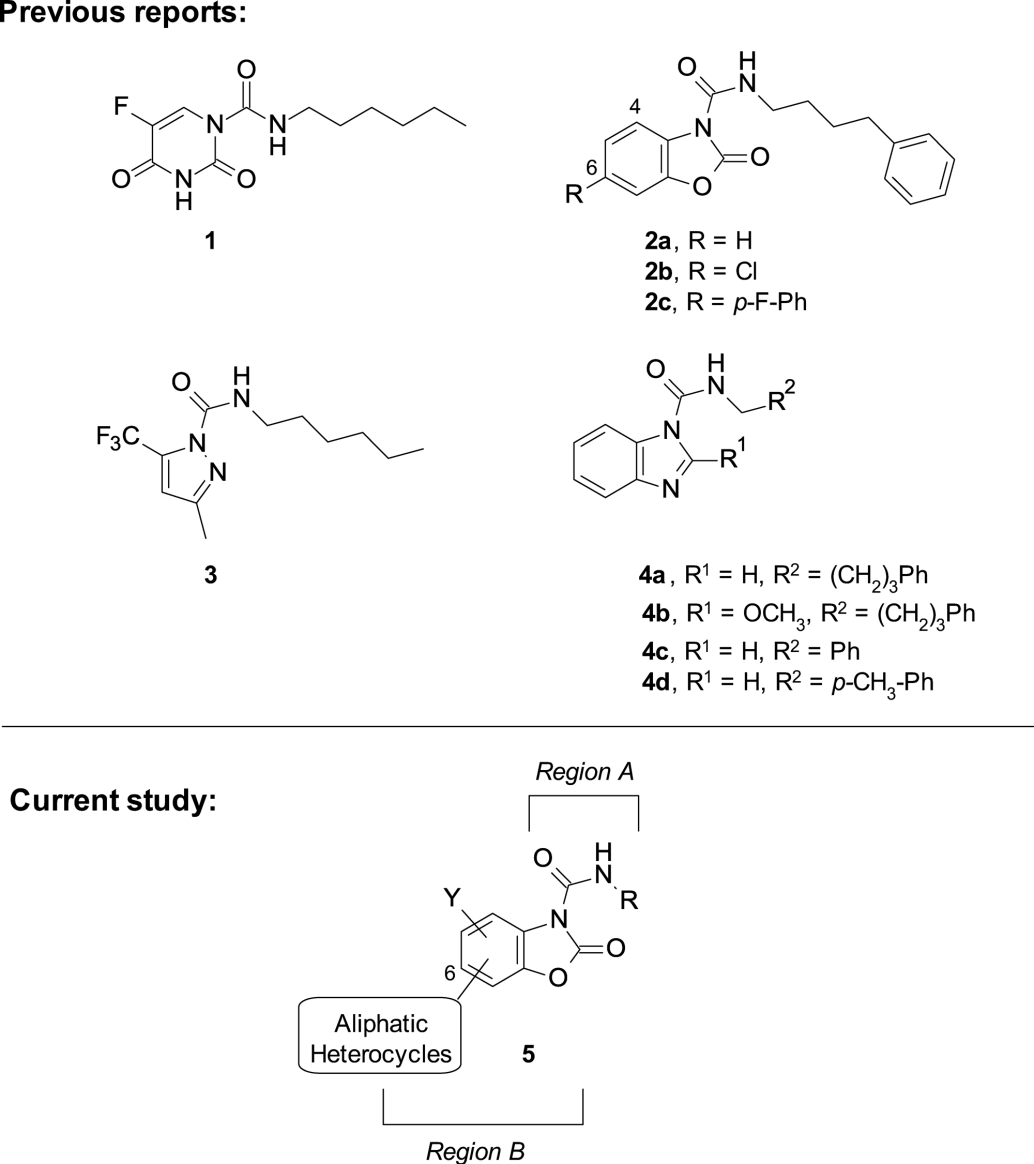
Structures of representative known AC inhibitors (**1**–**4**) (top) and general structure of the benzoxazolone carboxamide series **5** explored in this study (bottom).

Efforts over the last decade to develop potent AC inhibitors have resulted in limited success. The first structural analysis of mammalian AC has recently been solved by Gebai and co-workers,^35^ which may aid future medicinal chemistry programs. In 2013, Realini et al. reported the discovery of carmofur **1** and some close uracil analogs as the first class of single-digit nanomolar inhibitors of intracellular AC activity and studied their potential use as chemosensitizing agents (Figure 2).^36,37^ Despite being potent AC inhibitors, the uracil derivatives suffered from low chemical and metabolic stability. Subsequently, Diamanti et al. selected compound **1** as a ligand template for a computational-assisted virtual screening approach, leading to the identification of a new class of potent AC inhibitors, the pyrazole carboxamides.^38^ However, although very potent against AC activity, as exemplified by pyrazole **3**, this class of molecules suffered from low metabolic stability, limiting their therapeutic potential (Figure 2).

An alternative approach, consisting of a screening campaign of a small compound library, led to the identification of a novel and very promising class of covalent AC inhibitors, the benzoxazolone carboxamides, exemplified by the hit **2a** (Figure 2).^39^ Preliminary chemical exploration of this series led to the identification of **2b**(39) and **2c**(40) as more advanced and systematically active analogs. More recently, Ortega et al. reported a systematic computational investigation of the general pharmacophore model for AC inhibition, comprising a 6 + 5 fused ring heterocycle linked to an aliphatic substituent via a urea moiety. These studies resulted in the identification of the novel class of benzimidazole derivatives **4a**–**d** with promising activity in different melanoma cell lines (Figure 2).^41^

Although some of the molecules discussed above exhibited potent inhibitory effects toward AC, they generally suffer from low aqueous solubility and moderate chemical or metabolic stability, which hamper their further development. As part of our continued efforts to optimize the class of benzoxazolone carboxamides, we further extended the preliminary studies around **2b**(39) (and **2c**)^40^ and performed a focused structure–activity relationship (SAR) study around this scaffold (compound **5**, Figure 2), with the aim of identifying an optimal compound with improved physicochemical and pharmacokinetic profiles favoring oral administration. The subject of this manuscript describes the lead optimization and medicinal chemistry strategies that led to the discovery of **22m** as a lead candidate with improved oral bioavailability and excellent distribution to the CNS.

## Chemistry

**Scheme 1:**
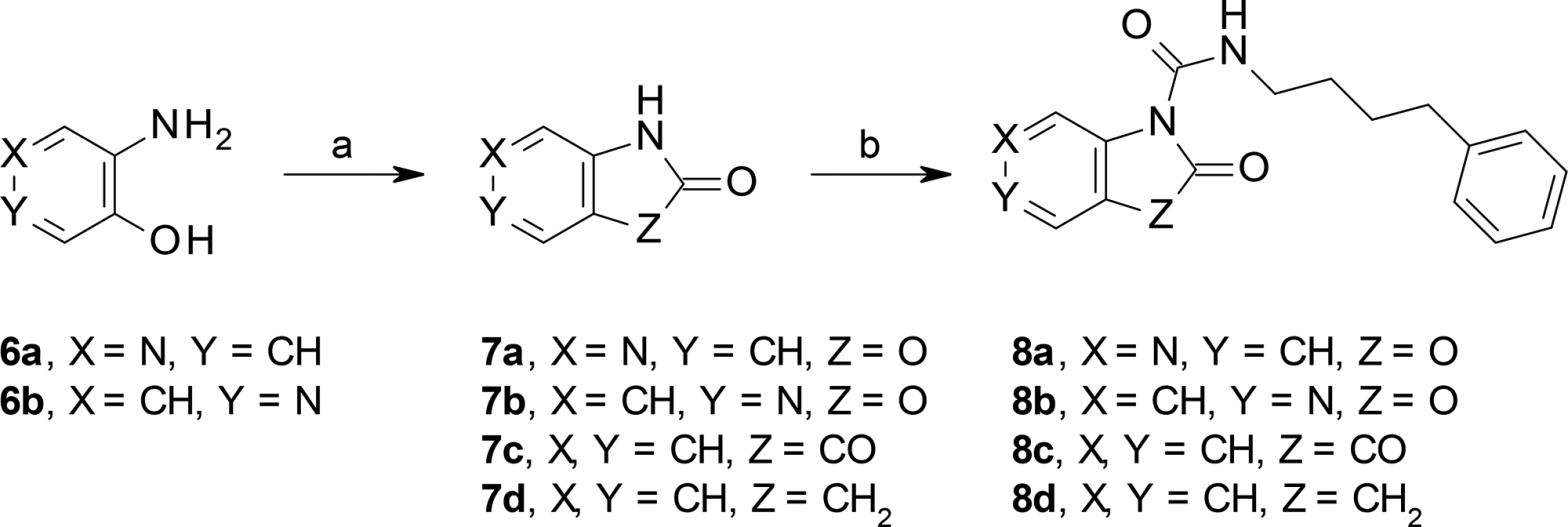
Synthesis of **8a**–**d** Reagents and conditions: (a) CDI, MeCN, rt, 2 h; (b) 4-phenylbutyl isocyanate, DMAP, toluene/DMF, rt, 2 h (20–60% over two steps for **8a** and **8b**); 4-phenylbutyl isocyanate, Et_3_N, MeCN, rt, 2 h (20–26% for **8c** and **8d**).

**Scheme 2:**
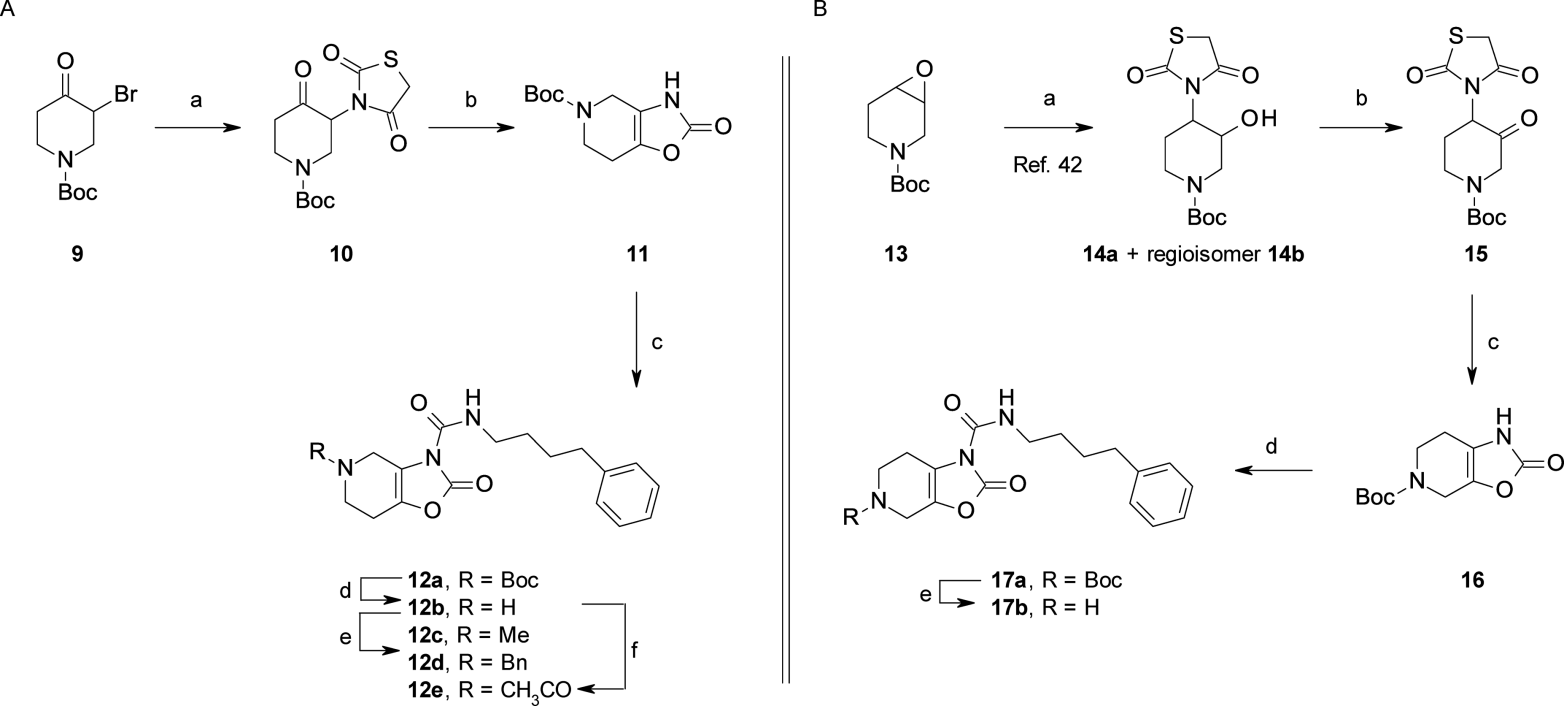
Synthesis of fused bicyclic piperidine-oxazolone derivatives **12a**–**e**, **17a**, and **17b** Reagents and conditions: for the synthesis of **12a**–**e**: (a) TZD, K_2_CO_3_, DMF, rt, 2 h (85%); (b) *t*BuOK, THF, rt, 30 min (60%); (c) 4-phenylbutyl isocyanate, DMAP, MeCN, rt, 16 h (68%); (d) 4 M HCl, dioxane, rt, 1 h (60%); (e) HCHO (for **12c**) or PhCHO (for **12d**), NaBH(OAc)_3_, AcOH, MeCN, rt, 3 h (56–90%); (f) AcCl, Et_3_N, DCM, rt, 3 h (62%). For the synthesis of **17a** and **17b**: (a) TZD, Mg(ClO_4_)_2_, DMF, 115 °C, 5 h (50%); (b) Dess–Martin reagent, DCM, 0 °C to rt, 12 h (70%); (c) *t*BuOK, THF, rt, 30 min; (d) 4-phenylbutyl isocyanate, DMAP, MeCN, rt, 30 min (25% over two steps); (e) 4 M HCl, dioxane, rt, 1 h (60%).

**Scheme 3:**
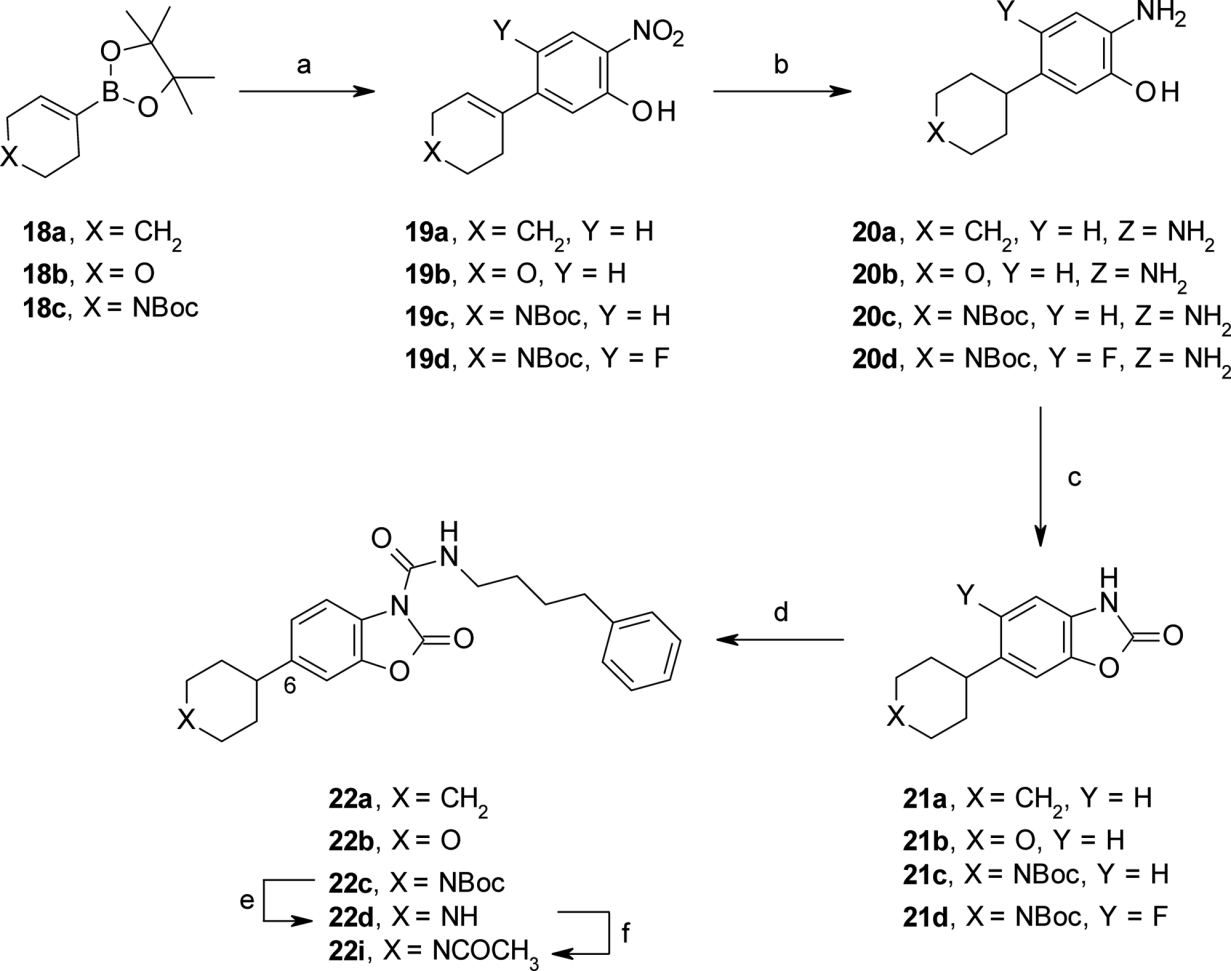
Synthesis of C(6)-substituted benzoxazolone carboxamides **22a**–**d** and **22i** Reagents and conditions: (a) 5-bromo-2-nitrophenol (for **19a**–**c**), 5-bromo-4-fluoro-2-nitrophenol (for **19d**), Pd(PPh_3_)_4_, 2 M Na_2_CO_3_, dioxane, reflux, 18 h (56–90%); (b) H-Cube, Pd/C, EtOAc, rt, 1–2 h; (c) CDI, MeCN, 60 °C, 2 h (60–84% over two steps); (d) 4-phenylbutyl isocyanate, DMAP, MeCN, rt, 16 h (50–98%); (e) 4 M HCl, dioxane, rt, 3 h (86%); (f) AcCl, Et_3_N, THF, rt, 4 h (90%).

**Scheme 4:**
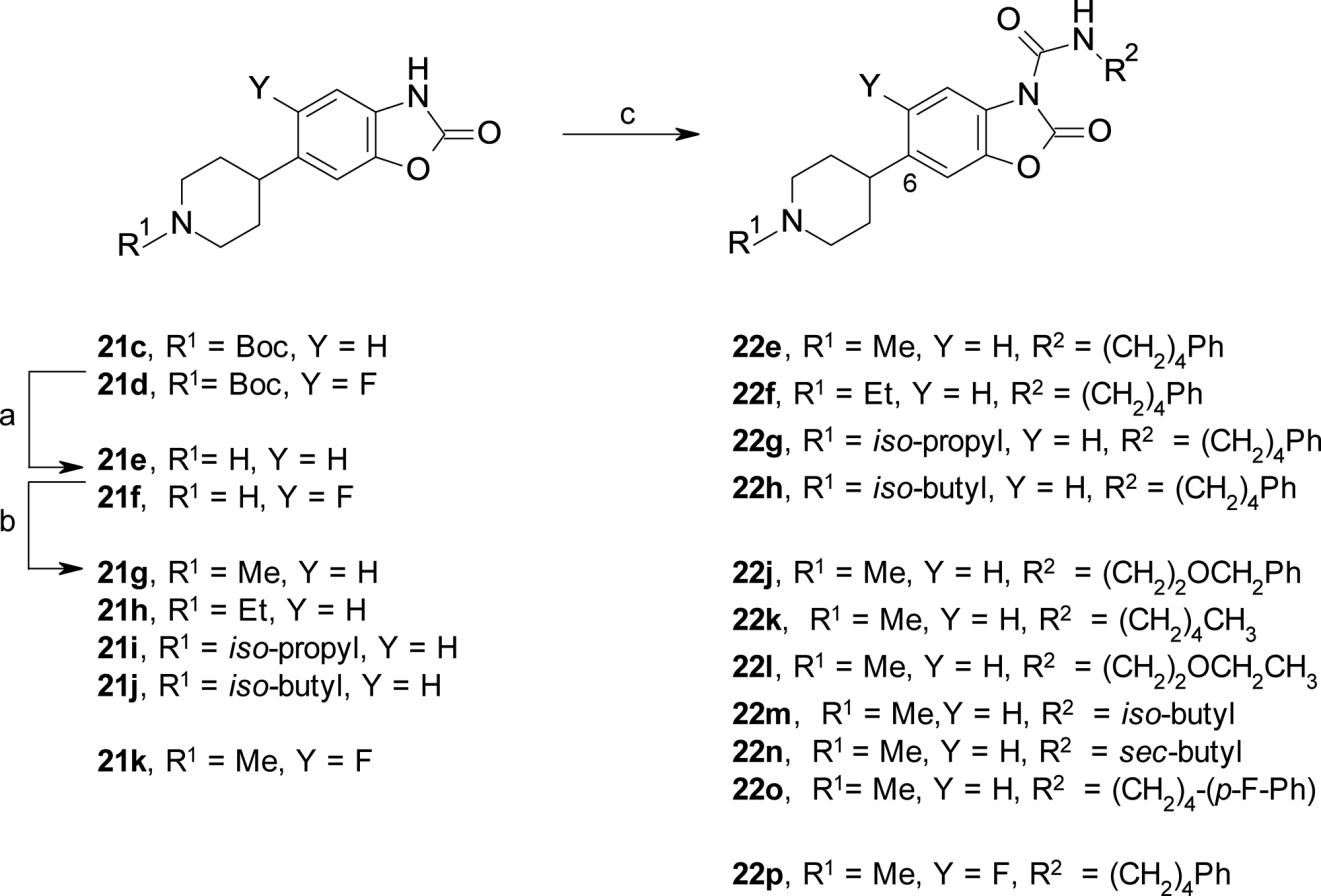
Synthesis of C(6)-substituted benzoxazolone carboxamides **22e**–**h** and **22j**–**p** Reagents and conditions: (a) 4 M HCl, dioxane, rt, 3 h; (b) RCHO, AcOH, NaBH(OAc)_3_, DCE, THF or MeCN, rt, 1–3 h (40% over two steps for **21i**, quant. For **21h**); (c) RNCO, DMAP, MeCN, rt, 2–16 h (50–73% for **22e**–**h**, **22k**, and **22p**); or RNH_2_, triphosgene, Et_3_N, DCM, 0 °C to rt, 2 h (45% for **22m**), or RNH_2_, Boc_2_O, DMAP, MeCN, rt, 1 h (24–40% for **22j**, **22l**, **22n**, and **22o**).

**Scheme 5:**
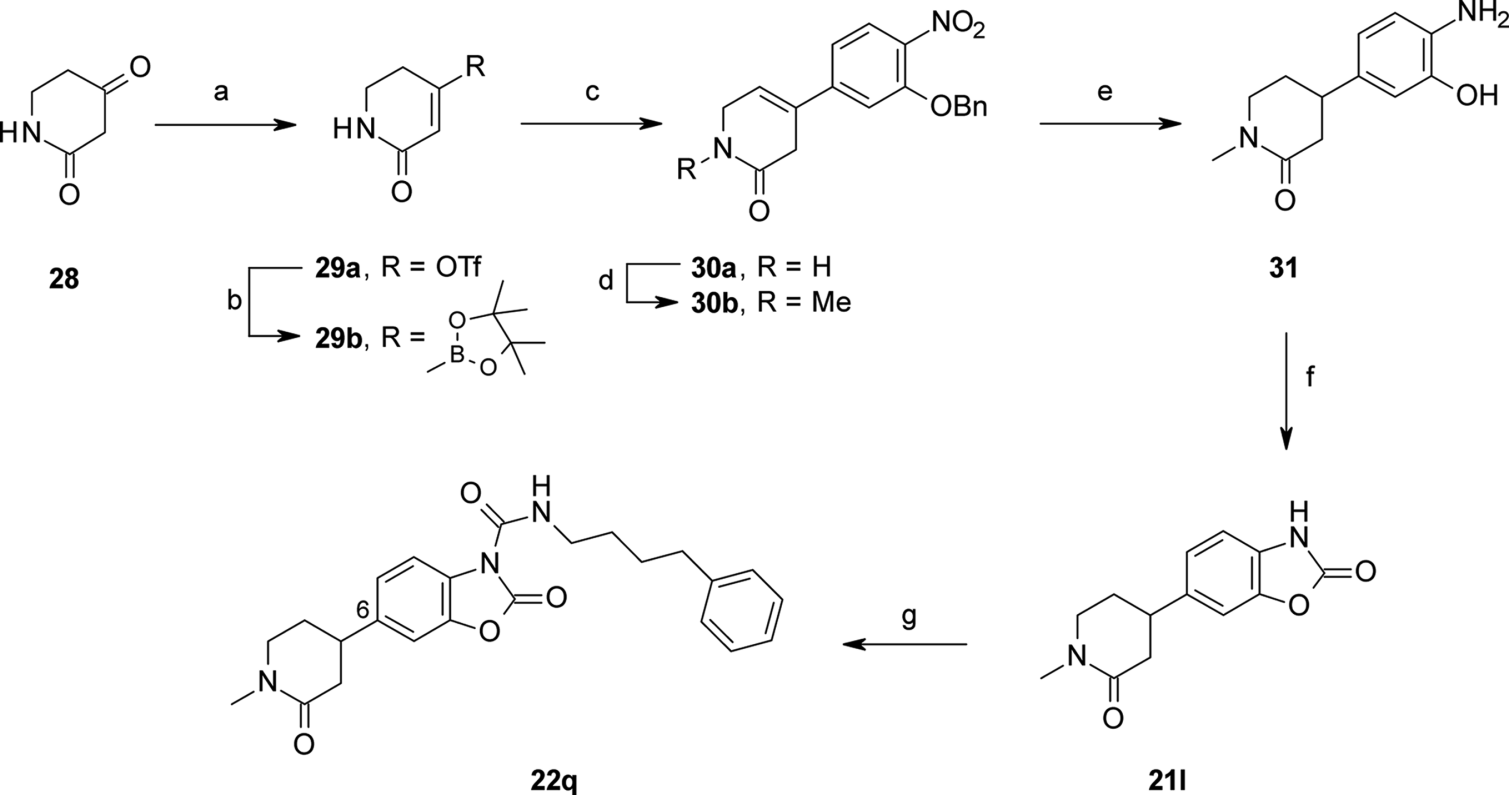
Synthesis of **22q** Reagents and conditions: (a) *N*,*N*-bis(trifluoromethanesulfonyl)aniline, Et_3_N, THF, 0 °C, 16 h (74%); (b) Pd(dppf)Cl_2_, [B_2_(pin)_2_], KOAc, dioxane, 70 °C, 3 h; (c) 2-(benzyloxy)-4-bromo-1-nitrobenzene, Na_2_CO_3_ 2 M, 70 °C, 1 h (93% over two steps) (d) NaH, MeI, THF, 0 °C, 20 h (45%); (e) H_2_, 10% Pd/C, EtOH, rt, 1 h; (f) CDI, MeCN, rt, 1 h (70% over two steps); (g) 4-phenylbutyl isocyanate, DMAP, pyridine, rt, 16 h (90%).

**Scheme 6:**
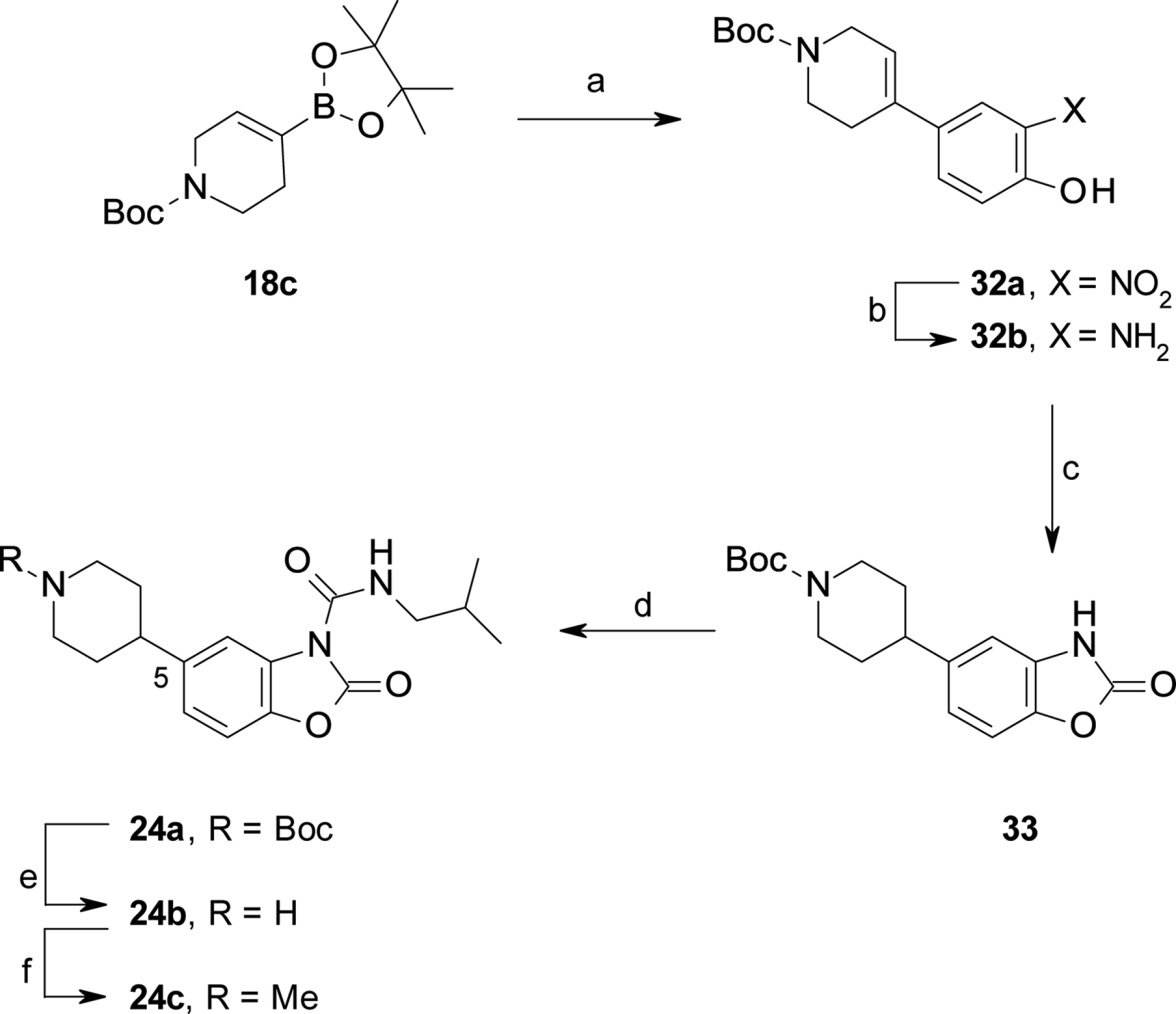
Synthesis of **24c** Reagents and conditions: (a) 4-bromo-2-nitrophenol, PdCl_2_(PPh_3_)_2_, 2 M Na_2_CO_3_, dioxane, reflux, 2 h (95%); (b) 10% Pd/C, cyclohexene, MeOH, reflux, 4 h; (c) CDI, MeCN, rt, 3 h (60% over two steps); (d) isobutylamine, triphosgene, Et_3_N, DCM (70%); (e) 4 M HCl, dioxane (95%); (f) HCHO, NaBH(OAc)_3_, AcOH, MeCN, rt, 2 h (83%).

**Scheme 7:**
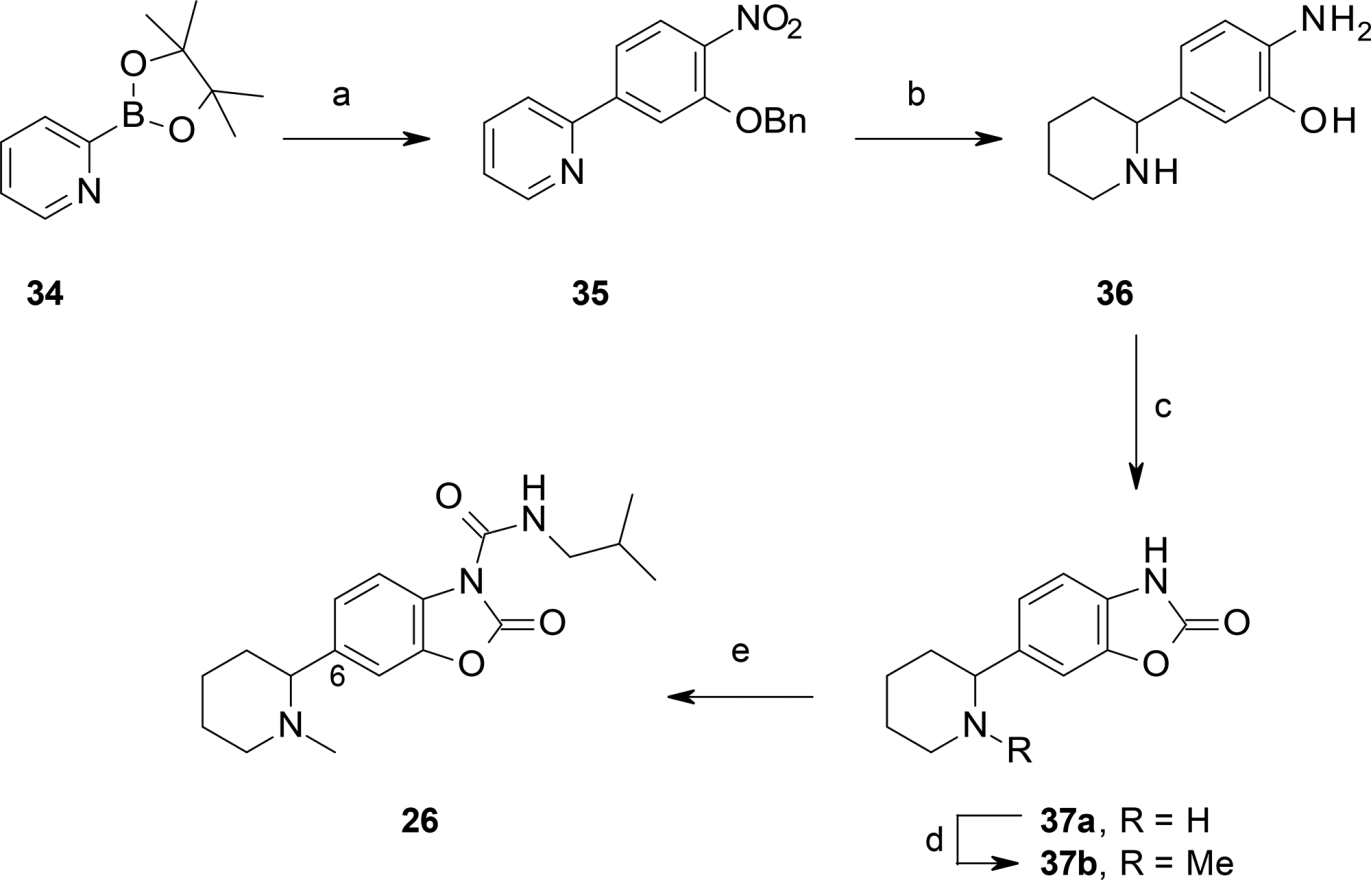
Synthesis of **26** Reagents and conditions: (a) 2-benzyloxy-4-bromo-1-nitrobenzene, Pd(dppf)Cl_2_, Na_2_CO_3_, dioxane, reflux, 16 h (40%); (b) cyclohexene, Pd/C, MeOH, 70 °C, 2 h; (c) CDI, MeCN, rt, 16 h; (d) HCHO, NaBH(OAc)_3_, AcOH, MeCN, rt, 2 h (43% over three steps); (e) isobutylamine, triphosgene, Et_3_N, DCM, rt, 4 h (30%).

**Scheme 8:**
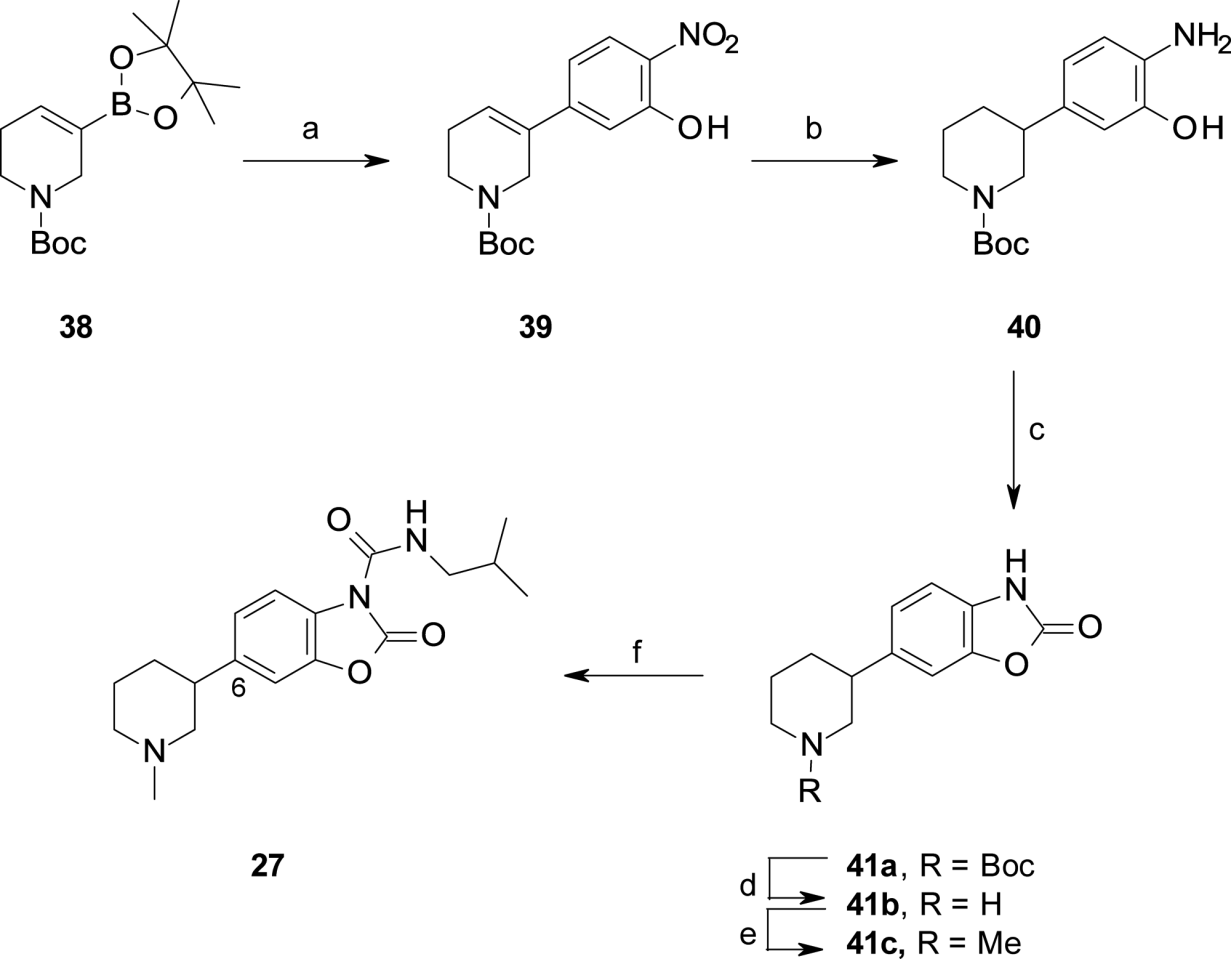
Synthesis of **27** Reagents and conditions: (a) 5-bromo-2-nitrophenol, Pd(dppf)Cl_2_, Na_2_CO_3_, dioxane, reflux, 2 h (53%); (b) 10% Pd/C, cyclohexene, EtOH, 65 °C, 4 h; (c) CDI, MeCN, rt, 1 h (90% over two steps) (d) 4 M HCl, dioxane, rt, 30 min; (e) HCHO, NaBH(OAc)_3_, AcOH, MeCN, rt, 30 min (70% over two steps); (f) isobutylamine, triphosgene, Et_3_N, DCM, rt, 3 h (70%).

**Scheme 9:**
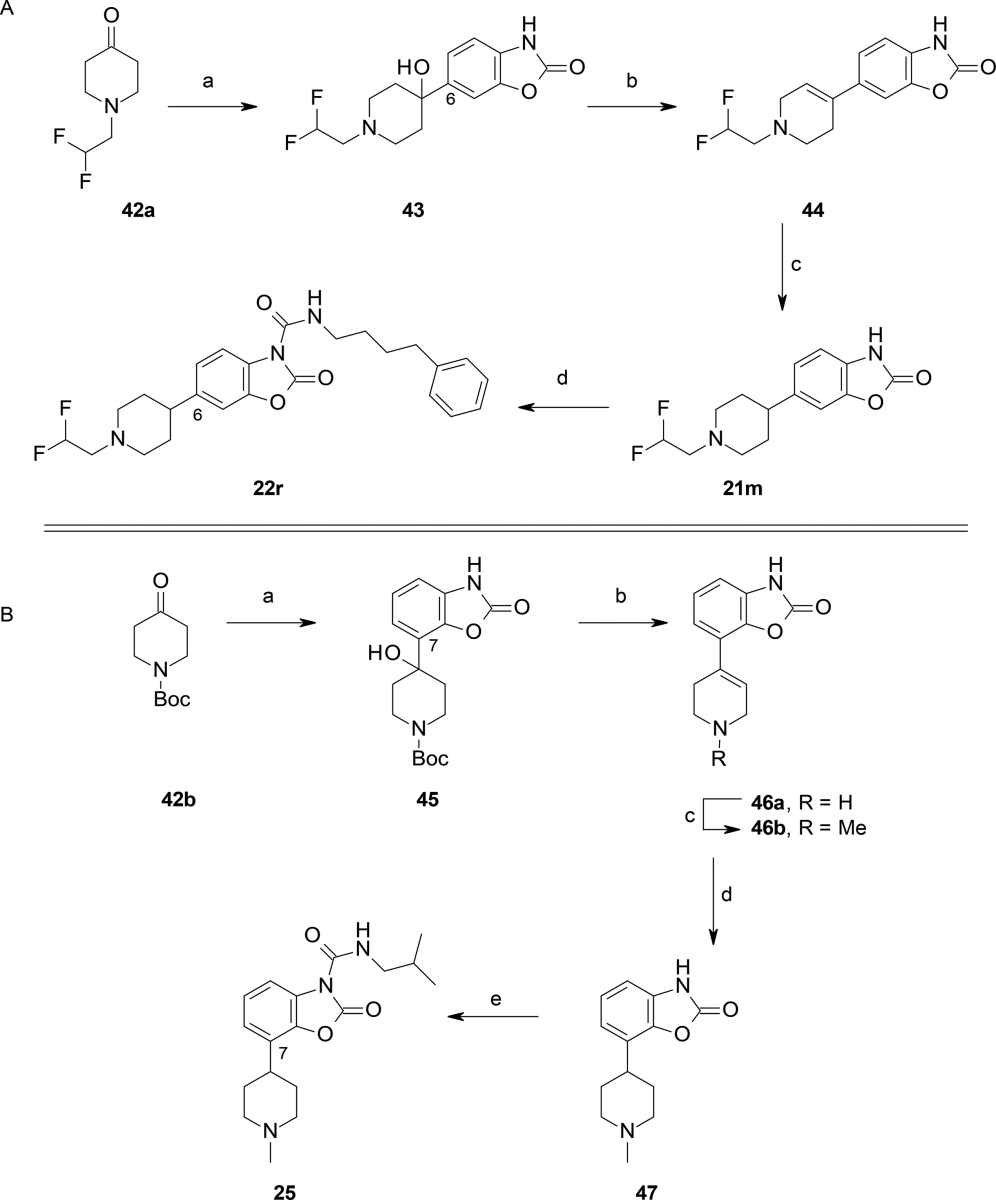
Synthesis of **22r** and **25** Reagents and conditions: for the synthesis of **22r**: (a) 6-bromo-3*H*-1,3-benzoxazol-2-one, MeMgBr, *n*-BuLi, THF, −78 °C, 2 h (30%); (b) *p*-TsOH, toluene, reflux, 1 h (quant.); (c) H_2_, 10% Pd/C, MeOH, 60 °C, 2 h; (d) 4-phenylbutyl isocyanate, DMAP, pyridine (73% over two steps). For the synthesis of **25**: (a) 7-bromo-3*H*-1,3-benzoxazol-2-one, MeMgBr, *n*-BuLi, THF, −78 °C 1.5 h (44%); (b) *p*-TsOH, toluene, 90 °C, 3 h (quant.); (c) HCHO, NaBH(OAc)_3_, MeCN, rt, 16 h; (d) H_2_, Pd/C, MeOH, 40 °C, 2 h; (e) isobutylamine, triphosgene, Et_3_N, DCM (30% over three steps).

**Scheme 10:**
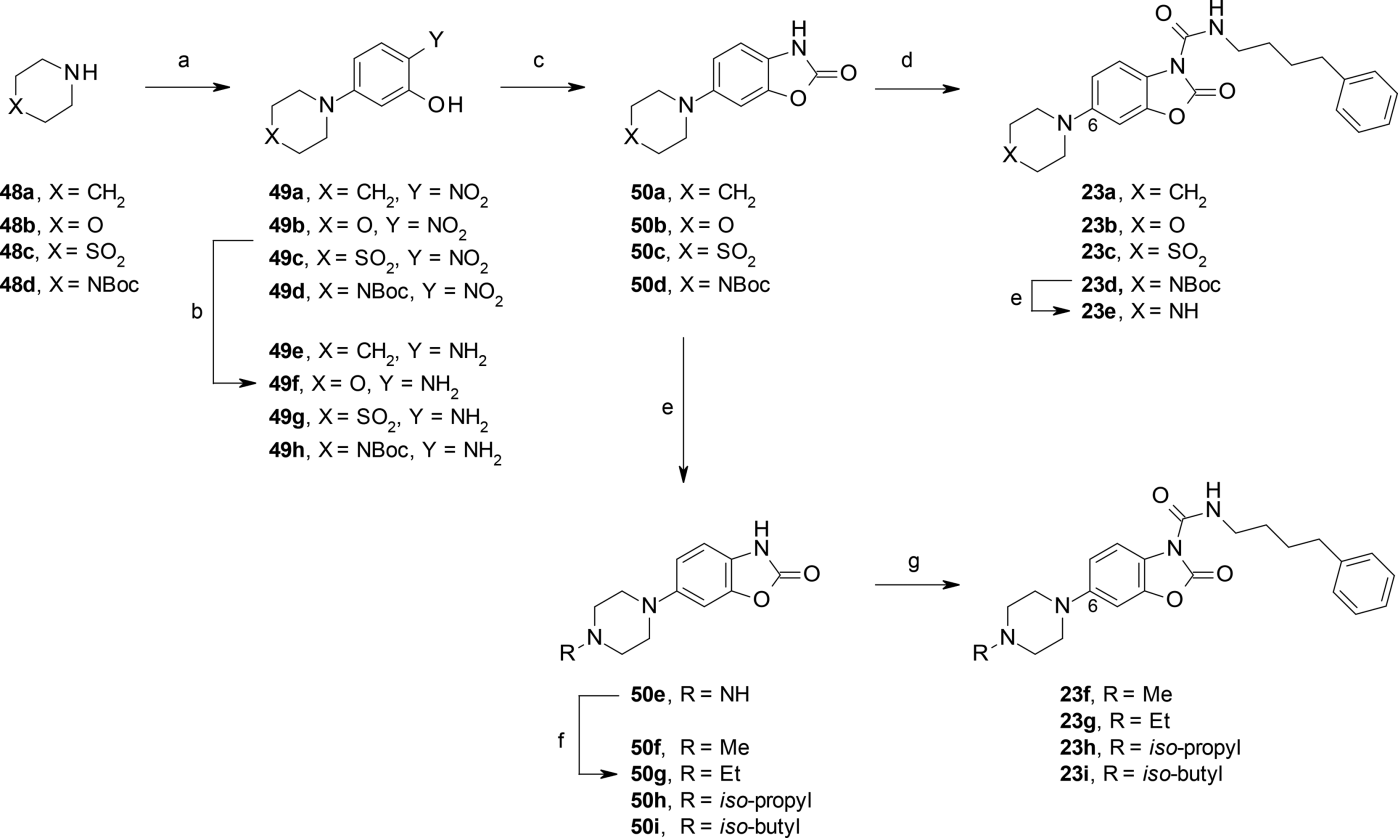
Synthesis of C(6)-substituted benzoxazolone carboxamides **23a**–**i** Reagents and conditions: (a) 5-fluoro-2-nitrophenol, DIPEA, MeCN, 60–80 °C, 15 h (40% for **49c**); (b) 10% Pd/C, cyclohexene, MeOH, reflux, 2–16 h; (c) CDI, MeCN, rt (or 50 °C for **49g**), 2 h (75–80% over three steps for **50a** and **50d**; 45–60% over two steps for **50b** and **50c**); (d) 4-phenylbutyl isocyanate, DMAP, MeCN, rt, 16 h (20–85%); (e) 4 M HCl, dioxane, rt, 3 h (quant.); (f) RCHO, AcOH, NaBH(OAc)_3_, DCE, THF or MeCN, rt, 2 h (60–90%); (g) 4-phenylbutyl isocyanate, DMAP, MeCN, rt, 16 h (30–75%).

**Scheme 11:**
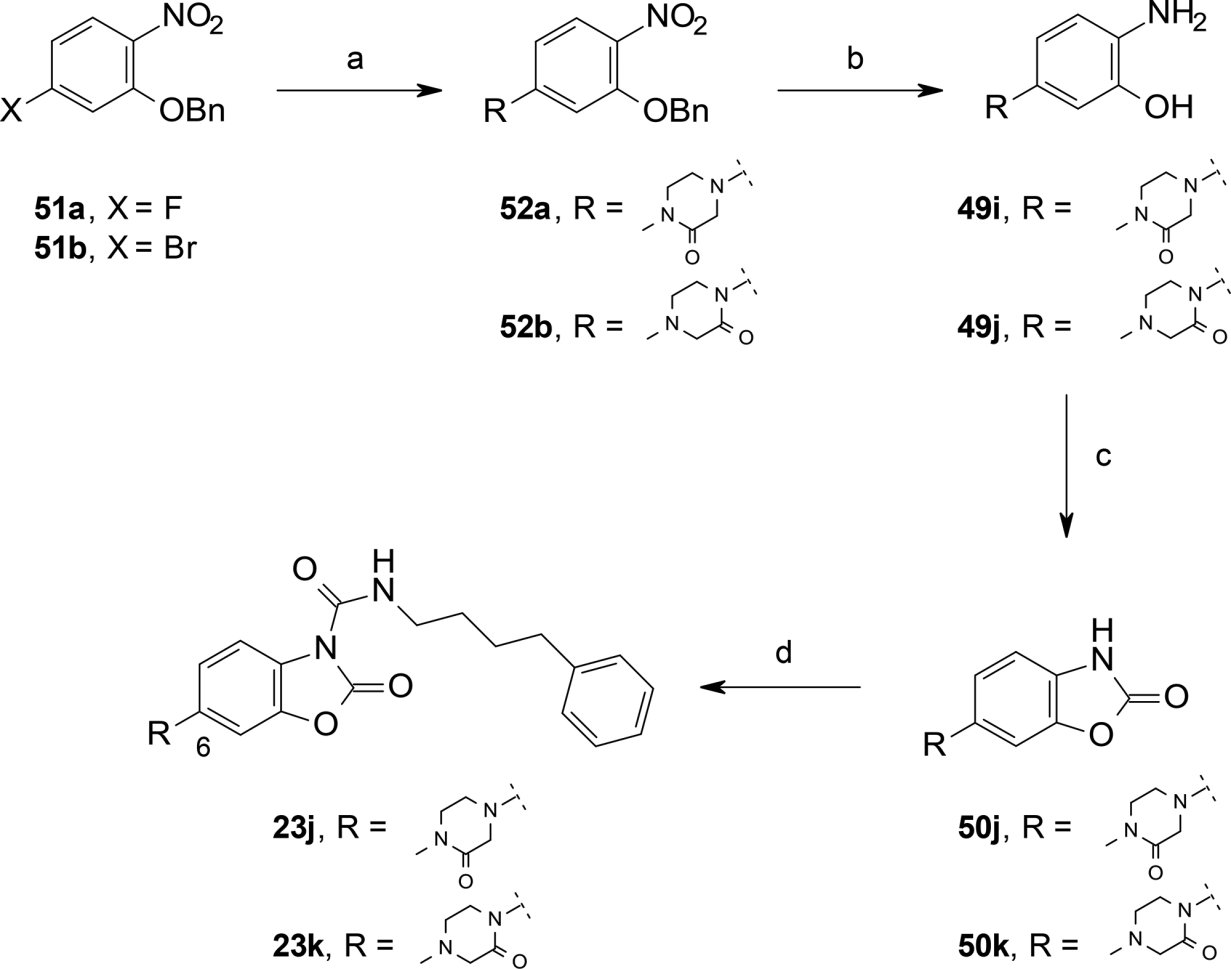
Synthesis of **23j** and **23**k Reagents and conditions: (a) 1-methylpiperazin-2-one (for **52a**), Et_3_N, MeCN, 80 °C, 16 h (60%); 4-methylpiperazin-2-one (for **52b**), CuI, K_3_PO_4_, *N*,*N*-dimethyl-1,2-ethanediamine, dioxane, reflux, 24 h (50%); (b) 10% Pd/C, cyclohexene, EtOH, 65 °C, 16 h; (c) CDI, MeCN, rt, 1 h (70–80% over two steps); (d) 4-phenylbutyl isocyanate, DMAP, MeCN, rt, 16 h (10–20%).

All target compounds were prepared by synthetic routes outlined in Schemes 1–11. Compounds **8a**–**d** were synthesized under standard conditions by reacting **7a–d** with 4-phenylbutyl isocyanate (Scheme 1). The novel core scaffold of **12a** was prepared in three steps from the α-bromo ketone **9** (Scheme 2A). Reaction with TZD gave compound **10**, which was then converted in moderate yield to the fused bicyclic derivative **11** via an intramolecular cyclization under basic conditions in anhydrous THF. Subsequent coupling of **11** to 4-phenylbutyl isocyanate gave **12a**, which upon removal of the *N*-Boc protecting group gave the key intermediate **12b**, which was subsequently transformed to **12c**–**e** via standard reductive amination and acetylation reactions. Alternatively, the isomeric key intermediate **17b** was prepared in four steps from the commercially available epoxide **13** (Scheme 2B). Ring opening^42^ and subsequent oxidation of the corresponding alcohol **14a** followed by intramolecular cyclization of **15** afforded compound **16**. Finally, as discussed for the synthesis of **12b**, standard reactions transformed **16** to **17b**.

We introduced cyclic and heterocyclic groups at C(5)-, C(6)-, and C(7)-positions of the benzoxazolone cores by exploring different synthetic pathways (Schemes 3–11). The exploration at the C(4)-position of the benzoxazolone scaffold was abandoned because, in accordance with previously reported results on the 4-Me and 4-Ph derivatives of **2a** (Figure 2),^40^ we experienced a pronounced chemical instability of our targeted C(4)-derivatives.

The C(6)-substituted benzoxazolones **21a**–**d** were prepared in three steps starting from boronic esters **18a**–**c**, using Pd-catalyzed cross coupling reactions with the corresponding bromo-nitrophenols followed by hydrogenation and intramolecular cyclization in the presence of CDI (Scheme 3). An additional step consisting of the in situ formation of boronic ester **29b**, from ketone **28** via enol triflate **29a**, was necessary for the preparation of the benzoxazolone **21l** (Scheme 5). A Pd-catalyzed cross coupling procedure was also used for the synthesis of the C(5)-substituted benzoxazolone **33** (Scheme 6) and other C(6)-substituted benzoxazolones, such as **37a** and **41a** (Schemes 7 and 8). In contrast, to overcome some synthetic problems in the Pd-catalyzed cross coupling reaction, we performed an alternative synthetic approach for the preparation of the benzoxazolone **21m** (Scheme 9A). Lithium-halogen exchange of 6-bromo-3*H-*1,3-benzoxazol-2-one^43^ followed by the addition of the ketone **42a** afforded the alcohol **43**, which upon dehydration and hydrogenation led to the key intermediate **21m**. A similar synthetic procedure was applied to insert the functionalization at the C(7)-position of the benzoxazolone, as in **47** (Scheme 9B).

Other C(6)-substituted benzoxazolones, for example, **23a**–**d** and **50j** (Schemes 10 and 11), were prepared in three steps and in satisfactory yields using a nucleophilic aromatic substitution (SNAr)^44^ reaction of activated fluoro-phenyls with a set of heterocyclic amines followed by hydrogenation and intramolecular cyclization reaction with CDI. An alternative approach was used for the synthesis of **50k** (Scheme 11). In this case, a Cu-catalyzed cross coupling *N*-arylation of *O*-Bn-protected bromo-nitrophenol **51b** with 4-methylpiperazin-2-one afforded **52b** in acceptable yield,^45^ which upon standard reactions led to the benzoxazolone **50k**.

Finally, the carboxamide functionalities were introduced under standard conditions, which involved the reaction of the benzoxazolone intermediates with the corresponding commercially available isocyanates, as in the preparation of **22a**–**c** (Scheme 3) and **23a**–**d** (Scheme 10). Alternatively, the isocyanates were prepared in situ, upon activation of the corresponding amines by reaction with Boc_2_O in the presence of DMAP in MeCN,^46^ as in the synthesis of **22j**, **22l**, **22n**, and **22o** (Scheme 4), or by reaction with triphosgene in the presence of Et_3_N in DCM,^47^ as in the synthesis of **22m** (Scheme 4).

## Results and Discussion

The four most potent classes of AC inhibitors described to date are illustrated in Figure 2. Each class is defined by the presence of a common chemical warhead—the urea-like functionality—that can covalently react with the catalytic cysteine (Cys-143) of AC to form a thioester bond.^35^ It has been reported that carboxamides **2a**(39) and **4a**–**b**^41^ form, upon incubation experiments with the protein, the corresponding cysteine adducts. This has recently been confirmed by Dementiev and co-workers, who described the crystal structural analysis of the uracil **1** covalently bound to Cys-143 at 2.7 Å resolution.^48^

While potent and, in some cases, systemically active,^39,40^ these molecules share two features that limit their use as oral drugs. First, the presence of a reactive warhead on the molecular scaffolds described to date contributes to their chemical and metabolic instability (e.g., uracil **1**),^37^ and second, the hydrophobic linear side chain that ensures target recognition and some degree of specificity negatively affects their drug-likeness (e.g., benzoxazolone **2a**).^39^ Thus, the need for optimized AC inhibitors remains an important issue to be addressed.^49^

**Figure 3:**
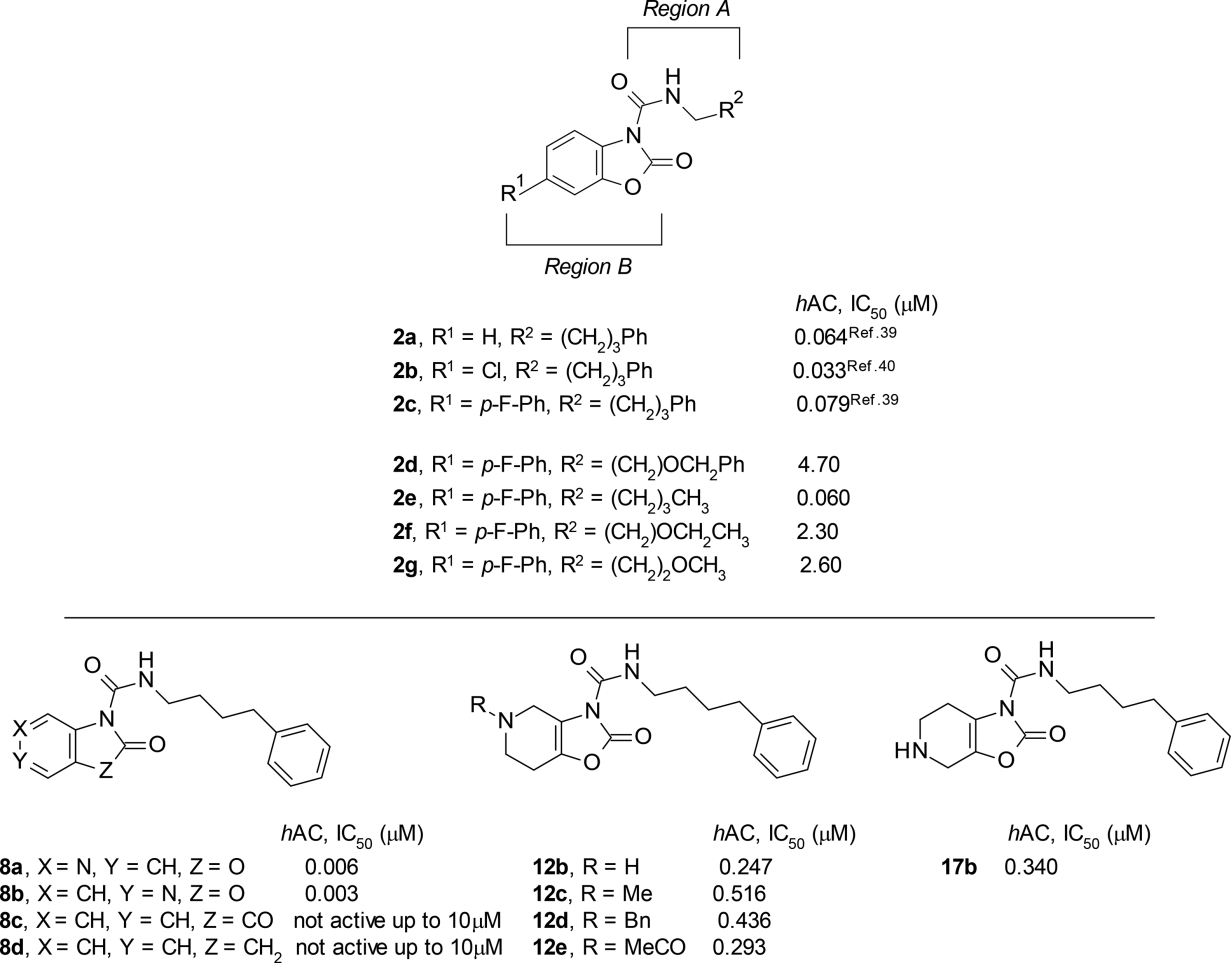
Inhibitory potencies (IC_50_ in μM) of compounds **2d**–**g**, **8a**–**d**, **12b**–**e**, and **17b** on the activity of *h*AC expressed in HEK-293 cells.

As previously reported, preliminary structural modifications of **2a** by variation of the lateral side chain of the urea functionality (*Region A*) and substitution of the benzoxazolone moiety (*Region B*) led to the identification of **2b**(39) and **2c**(40) (Figure 3). Despite good potency and enhanced drug-likeness compared to the previous uracil^37^ series, compounds **2b** and **2c** suffer from low solubility in aqueous media and moderate chemical and metabolic stability that limit their utility as oral drugs.^39,40^ To address these issues, our lead optimization strategy focused on designing additional structural modifications on *Regions A* and *B* (compound **5**, Figure 2) with the aim of improving the physicochemical and metabolic properties while maintaining inhibitory potency.

We initially investigated modifications of the lateral side chain (*Region A*) of **2c** confirming that, as previously reported with **2a** analogs,^40^ this region is involved in lipophilic interactions important for target recognition (Figure 3). In fact, different attempts to improve solubility and metabolic stability by reducing lipophilicity of the side chain were detrimental regarding potency (Figure 3). Although the removal of the phenyl ring was tolerated, as for the *n*-pentyl analog **2e** (*h*AC IC_50_ = 60 nM), no enhancement of solubility was observed (<1 μM, PBS, pH 7.4). Replacement of one methylene unit with an oxygen (e.g., ethers **2d**, **2f**, and **2g**) to increase the hydrophilicity significantly reduced the inhibitory potency to the μM range. A similar trend was observed for the corresponding analogs in the **2b** series (data not shown), indicating that the lipophilic side chain of the urea was very likely occupying a hydrophobic pocket.

We then shifted our attention to the left-hand side (*Region B*) of the scaffold by evaluating the replacement of the benzoxazolone moiety with some bioisosteric 6 + 5 fused ring heterocyclic systems (Figure 3), alternative to those already reported by Ortega et al.^41^

However, both the isatin analog **8c** and the oxindole analog **8d** were inactive at concentrations up to 10 μM. We then investigated the bioisosteric insertion of an aza-group in the phenyl ring of the benzoxazolone moiety, and this change resulted in very potent compounds. For example, compounds **8a** and **8b** gave *h*AC IC_50_’s of 6 and 3 nM, respectively, compared to the earlier compound **2a**,^39^ which has an *h*AC IC_50_ of 64 nM. We envisaged that the insertion of a polar group on the left-hand side of the scaffold (*Region B*) could have an impact on the solubility of this series in aqueous buffer (PBS, pH 7.4), but, unfortunately, both **8a** and **8b** had very poor chemical stability in these conditions (*t*_1/2_ < 15 min).

These findings prompted us to evaluate the inhibitory potency of the fused bicyclic derivatives **12b** and **17b** (Figure 3). We speculated that changing the left-hand-side leaving group at the urea functionality could have an effect on the chemical stability of the scaffold. Although we generally observed a loss in potency to the sub-μM range, regardless of the substituent (**12b**–**e**) or the position of the nitrogen atom (**17b**), we were pleased to notice that, as for **12b** and **17b**, this novel class of *h*AC inhibitors showed improved chemical stability in PBS at pH 7.4 (*t*_1/2_ > 8 h) and improved aqueous solubility (82 and 230 μM, respectively). Despite the novel chemotype of these AC inhibitors with promising physicochemical properties, our attempts to improve the potency of this series were unsuccessful (data not shown). In addition, although these compounds exhibited high mouse plasma stability (e.g., *t*_1/2_ > 2 h, for **12b** and **17b**), this class of molecules also suffered from poor mouse liver microsomal stability (*t*_1/2_ < 15 min).

Overall, these results confirmed the benzoxazolone moiety as a “privileged scaffold”, thus focusing our SAR strategy on *Region B* with the intention of reducing the lipophilicity by replacing the phenyl ring of **2c** at the C(6)-position with aliphatic heterocyclic rings (**5**, Figure 2). We envisaged that the reduction of the number of sp^2^-hybridized carbon atoms and the insertion of heteroatoms in this region could improve the overall physicochemical and metabolic stability of this class of inhibitors.^50,51^

We were pleased to observe that both the cyclohexyl analog **22a** and the tetrahydropyrane analog **22b** resulted in equipotent inhibition (*h*AC IC_50_ = 0.089 and 0.068 μM, respectively) compared to the corresponding phenyl derivative **2c**(39) (Table 1 and Figure 2). With these results in hand, we were then interested in evaluating the effect of increasing the hydrophilicity by the addition of a polar basic amine, as in the piperidine analogs **22d** and **22e**. With this modification, we observed that both compounds showed only a slight loss of potency compared to the aliphatic analog **22a** (*h*AC IC_50_ = 0.134 and 0.129 μM, respectively). Encouraged by these results, we explored the effect of other *N*-containing heterocyclic systems, such as the piperidine **23a**, the morpholine **23b**, the 1,1-dioxothiomorpholine **23c**, and the piperazines **23e** and **23f**. Overall, this set of compounds showed similar potency to the initial cyclohexyl analog **22a**. Notably, the piperidine **23a** and the piperazine **23f** were the most potent compounds, showing IC_50_ values of 0.080 and 0.116 μM, respectively (Table 1). Based on these promising results, we selected the piperidine and piperazine series, exemplified by **22d** and **23e**, respectively, as novel scaffolds for further studies, exploiting the presence of a distal nitrogen atom as an anchor point for additional structural modifications (Table 2). First, we evaluated the SAR exploration around the piperidine series by introducing both linear and branched alkyl chains on the nitrogen atom, such as the ethyl **22f**, isopropyl **22g**, and isobutyl **22h** analogs (Table 2). In general, no significant differences were observed on the inhibitory potency of these derivatives, with **22f**–**h** almost being equipotent to the unsubstituted **22d**. Moreover, the removal of the basic center by the introduction of either an exocyclic (**22i**) or endocyclic *N*-acyl group (**22q**) was tolerated, showing IC_50_ values of 0.064 and 0.105 μM, respectively. These results further confirmed that different polar groups were tolerated in this region of the scaffold.

**Table 1:**
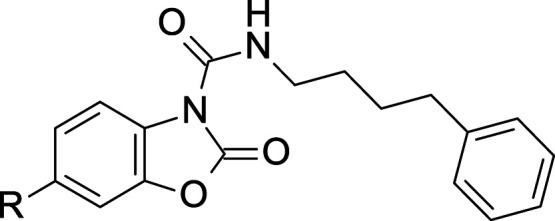

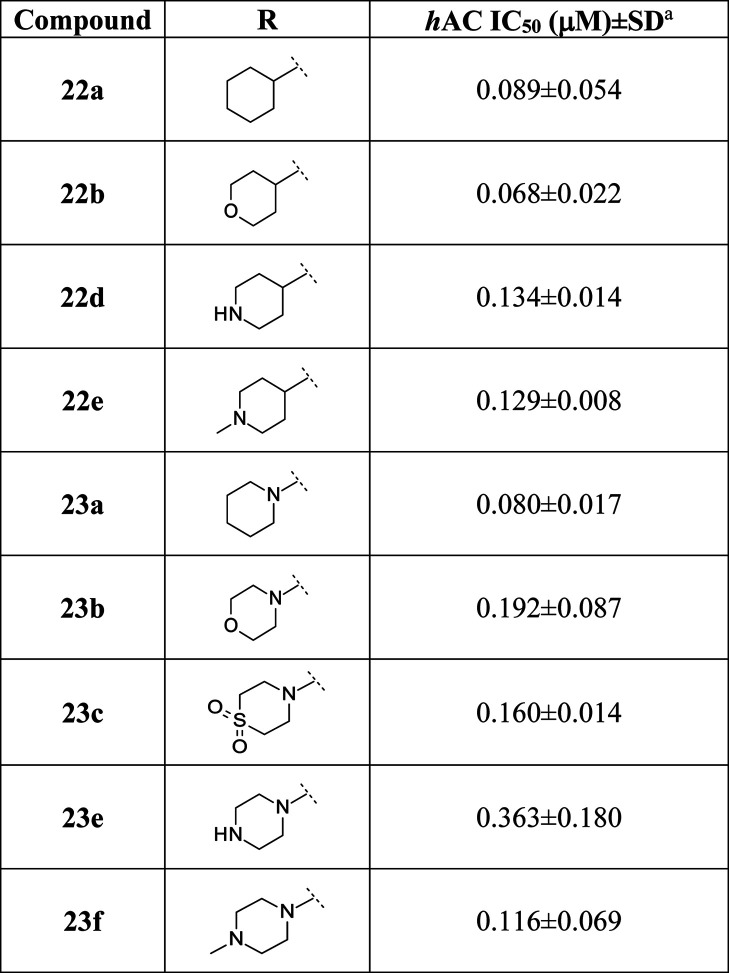
Inhibitory Potencies of Compounds **22a**, **22b**, **22d**, **22e**, **23a**–**c**, **23e**, and **23f** on the Activity of *h*AC

a IC_50_ values are the mean of at least three independent experiments, performed in three technical replicates.

**Table 2:**
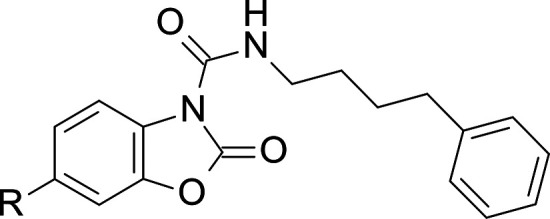

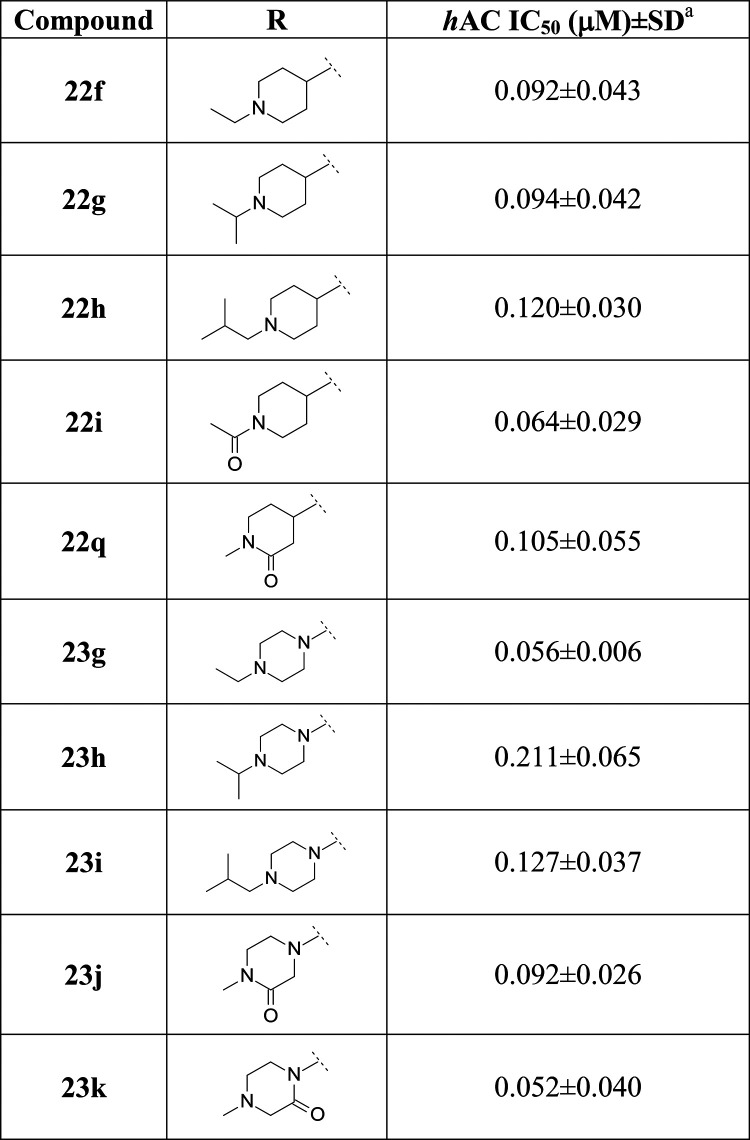
Inhibitory Potencies of Piperidines **22f**–**i** and **22q** and Piperazines **23g**–**k** on the Activity of *h*AC

a IC_50_ values are the mean of at least three independent experiments performed in three technical replicates.

The same strategy was applied to the piperazine series (Table 2). Specifically, both the *N*-alkyl derivatives **23g**–**i** and the piperazinones **23j** and **23k** resulted in more potent AC inhibition than the parent **23e**. For example, the *N*-ethyl piperazine **23g** and the piperazinone **23k** were almost 7-fold more potent than **23e** (*h*AC IC_50_ = 0.363 μM), showing IC_50_ values of 0.056 and 0.052 μM, respectively.

However, a comparison of the piperazine and piperidine series in terms of aqueous kinetic solubility (PBS, pH 7.4) in vitro metabolism highlighted some significant differences (Table 3). Interestingly, the piperidine analogs, bearing small linear alkyl groups (**22d**–**f**), were highly soluble (kinetic solubility >100 μM) and, in some cases (**22d**), had acceptable stability profiles both in mouse plasma and in liver microsomes. On the other hand, the piperidine derivatives, bearing more sterically hindered lipophilic alkyl groups, such as the isopropyl **22g** and isobutyl **22h**, or the acyls **22i** and **22q** suffered from low solubility and, with the exception of **22h**, poor stability in mouse plasma (Table 3). Conversely, all the piperazine derivatives generally suffered from poor aqueous solubility and poor microsomal and plasma stability (Table 3). As illustrative examples, the piperazines **23f** and **23g** showed poor solubility in water (<1 μM), rapid metabolism in liver microsomes, and poor plasma stability (*m*-plasma and *m*-liver microsomes, *t*_1/2_ < 5 min). Some improvement in microsomal stability was observed with the *des*-methylated **23e** and with **23k**, which bears a heterocyclic ring at a higher oxidative state compared to **23f**.

**Table 3:** Aqueous Kinetic Solubility and In Vitro Metabolism of Some Selected Compounds in the Piperidines **22d**–**i**, **22q** and Piperazines **23e**–**g**, **23j**, and **23k** Series

compound	solubility (μM)^a^ (PBS, pH 7.4)	*m-*plasma^b^*t*_1/2_ (min)	*m-*LM^c^ *t*_1/2_ (min)[% at 60 min]
piperidine series			
**22d**	150	60	>60 [70%]
**22e**	120	50	40
**22f**	198	50	60
**22g**	50	40	60
**22h**	<1	60	60
**22i**	<1	30	60
**22q**	20	36	35
piperazine series			
**23e**	20	30	45
**23f**	<1	<5	<5
**23g**	<1	<5	<5
**23j**	<1	20	30
**23k**	20	20	<5

a Aqueous kinetic solubility in phosphate-buffered saline. Values are the mean of at least two independent experiments performed in two technical replicates.

b Mouse plasma. Values are the mean of at least two independent experiments performed in two technical replicates.

c Mouse liver microsomes. Values are the mean of at least two independent experiments performed in two technical replicates.

With these results in hand, we focused our efforts on exploring *Regions A* and *B* of the *N*-methyl piperidine **22e**, as reported in Table 4. In order to reduce the lipophilicity and improve metabolic stability of this scaffold, we followed different strategies: (a) insertion of a heteroatom, removal of the phenyl ring, and reduction of the side chain length (*Region A*); and (b) removal of potential metabolic soft spots (*Regions A* and *B*).

**Table 4:**
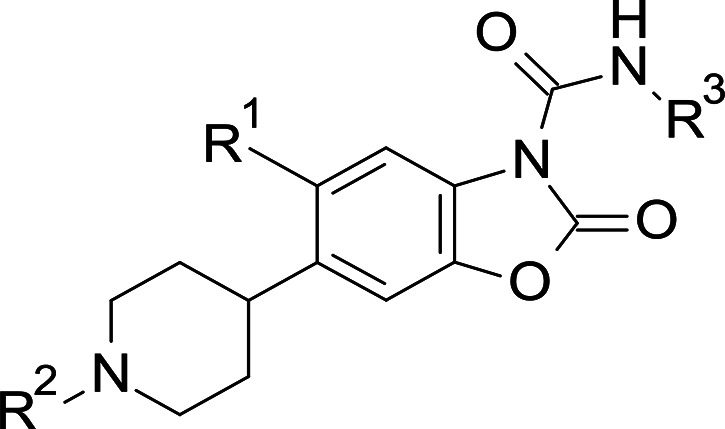

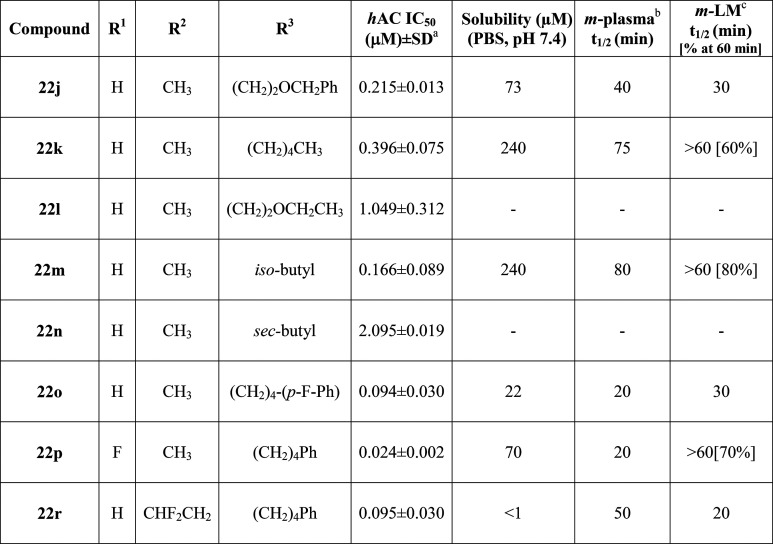
Inhibitory Potencies of Piperidines **22j**–**p** and **22r** on the Activity of *h*AC and Aqueous Kinetic Solubility and In Vitro Metabolism of Some Selected Compounds

a IC_50_ values are the mean of at least three independent experiments performed in three technical replicates.

b Aqueous kinetic solubility in phosphate-buffered saline. Values are the mean of at least two independent experiments performed in two technical replicates.

c Mouse plasma. Values are the mean of at least two independent experiments performed in two technical replicates.

d Mouse liver microsomes. Values are the mean of at least two independent experiments performed in two technical replicates.

An immediate loss in potency was observed with the removal of the lipophilic phenyl ring (**22 k**) or the insertion of an oxygen on the lateral chain (**22j** and **22l**), while the bioisosteric replacement of a fluorine on the distal phenyl ring resulted in **22o**, being almost equipotent to **22e** (Table 4). Nonetheless, exploration of *Region A* continued with the insertion of branched alkyl groups. We were pleased that the isobutyl analog **22m** (*h*AC IC_50_ = 0.166 μM) was equipotent to the corresponding butyl phenyl **22e**, demonstrating that it was possible to remove the phenyl group and reduce the overall lipophilicity without compromising potency. On the other hand, a methyl group adjacent to the urea functionality, such as the *sec*-butyl analog **22n**, was detrimental for potency, with an IC_50_ of 2.1 μM. Moving the SAR exploration back to *Region B*, insertion of a fluorine on the benzoxazolone ring **22p** boosted the inhibitory potency (IC_50_ = 0.024 μM), while the difluoroethyl analog **22r** showed similar potency (IC_50_ = 0.095 μM) to **22e**. The kinetic aqueous solubility and in vitro metabolic stability of a selection of compounds in the piperidine series are summarized in Table 4. Notably, while the insertion of an oxygen did not affect either the solubility or metabolic stability in microsomes of **22j** compared to **22e**, reducing lipophilicity with small aliphatic groups (**22k** and **22m**) was particularly beneficial. For example, **22k** and **22m** showed high aqueous solubility (240 μM) and improved plasma and liver microsomal stabilities (*t*_1/2_ > 60 min). On the other hand, attempts to improve the liver microsomal stability of **22e** by inserting a fluorine atom at different potential metabolic soft spots of both *Regions A* and *B* (compounds **22o**, **22p**, and **22r**) were not successful. Not surprisingly, these bioisosteric replacements negatively affected the aqueous solubilities of **22o**, **22p**, and **22r**, without a substantial improvement of the metabolic stability in microsomes.

With these results in hand, SAR studies continued on the scaffold of compound **22m** (Table 5). Specifically, we evaluated the effect of the location of both the *N-methyl piperidine ring*, at C(5)- and C(7)-positions of the benzoxazolone moiety (compounds **24c** and **25**), and the *N-methylated nitrogen atom*, within the piperidine nucleus (compounds **26** and **27**). Overall, we generally observed a loss in the inhibitory potency of these targeted analogs compared to **22m**, which was even more pronounced with compounds **26** and **27**, showing IC_50_ values in the μM range. Finally, the evaluation of the kinetic aqueous solubility and in vitro metabolism of **22m** and close analogs was completed (Table 5). In general, all the targeted compounds showed high solubility values in aqueous media, except for **24c**, which bears the piperidine ring at the C(5)-position of the benzoxazolone system. On the other hand, major differences were observed comparing their metabolic stability properties. In particular, we observed that substitution at the C(6)-position was critical to maintaining acceptable mouse plasma and liver microsomal stabilities (compound **22m**, *m*-plasma *t*_1/2_ = 80 min and *m*-liver microsomes *t*_1/2_ > 60 min (76% remaining at 1 h)). On the other hand, both derivatives with the piperidine ring at the C(5)- and C(7)-positions, **24c** and **25** showed reduced mouse plasma and liver microsomal stability. A similar trend was observed by moving the nitrogen atom to a different position on the piperidine ring, except for **27**, which showed a similar liver microsomal stability to **22m**. Due to its inhibitory potency and improved overall drug-likeness profile, the piperidine **22m** was selected for further biological and pharmacological investigations.

**Table 5:**
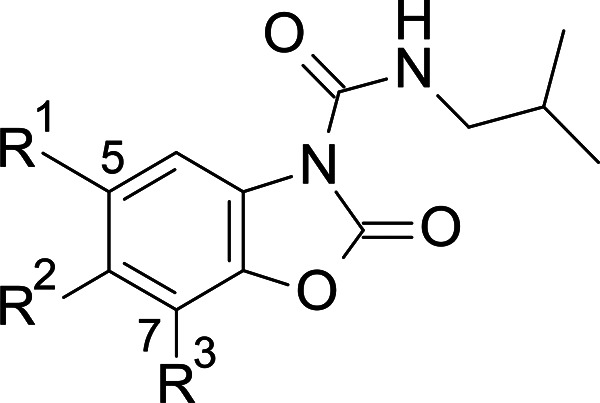

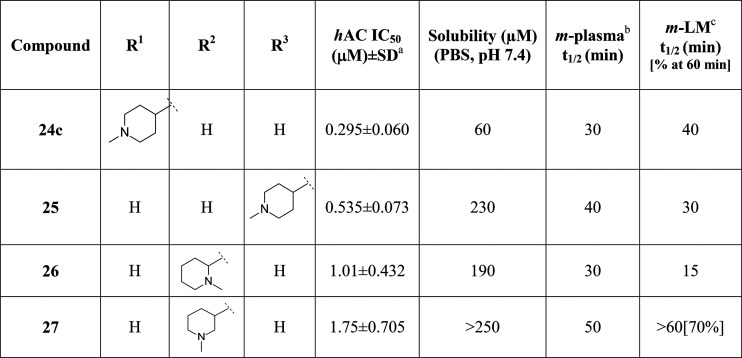
Inhibitory Potencies of Compounds **24c** and **25**–**27** on *h*AC and Aqueous Kinetic Solubility and In Vitro Metabolism

a IC_50_ values are the mean of at least three independent experiments performed in three technical replicates.

b Aqueous kinetic solubility in phosphate-buffered saline. Values are the mean of at least two independent experiments performed in two technical replicates.

c Mouse plasma. Values are the mean of at least two independent experiments performed in two technical replicates.

d Mouse liver microsomes. Values are the mean of at least two independent experiments performed in two technical replicates.

**Figure 4:**
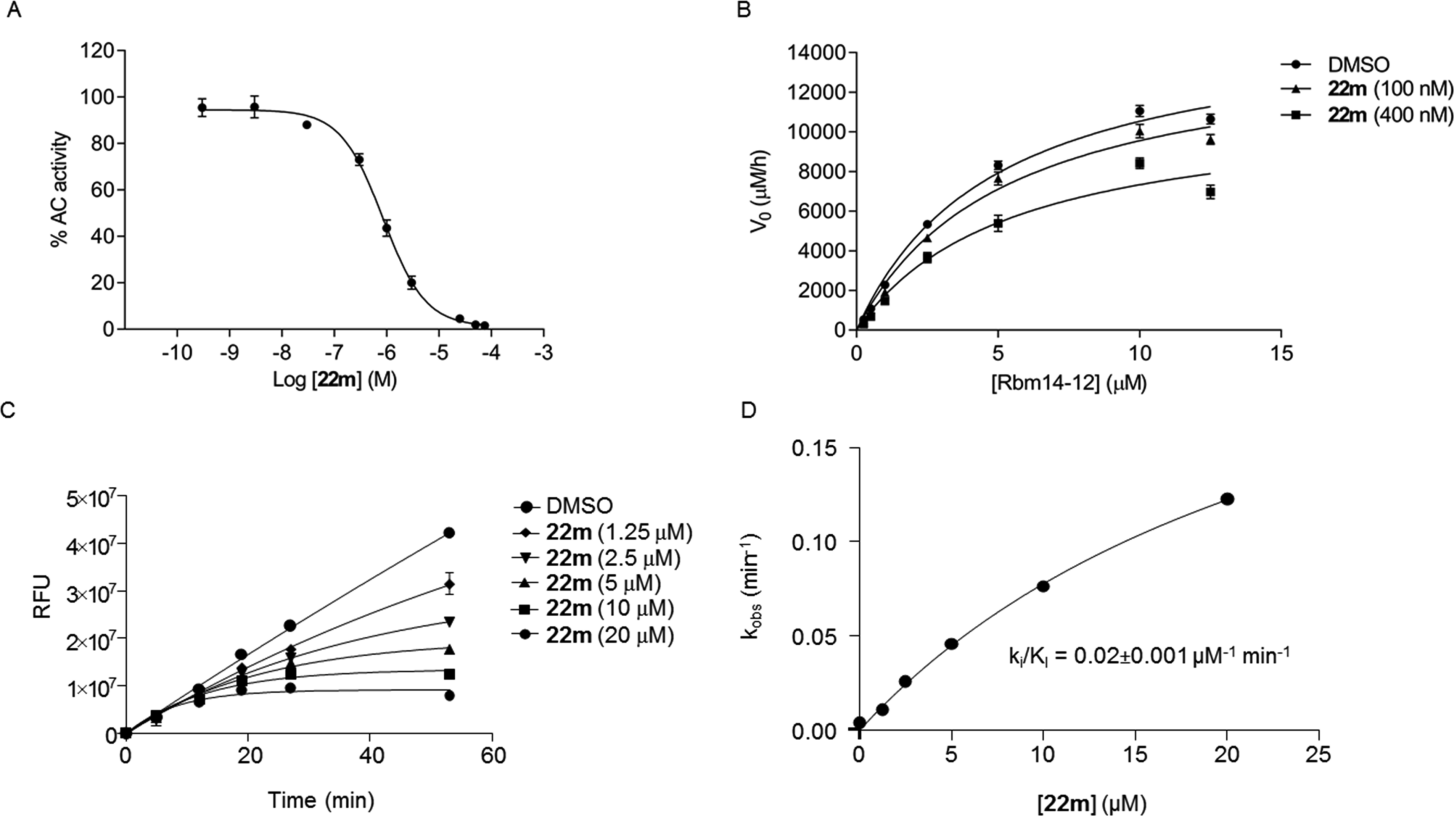
(A) Concentration–response curve for inhibition of *h*AC activity by **22m**; (B) Michaelis–Menten analysis of the reaction of *h*AC in the presence of vehicle (DMSO 1%, ●) or **22m** (100 nM, ▲; 400 nM, ■). Rbm 14–12: fluorogenic substrate of *h*AC; (C) time-dependent inhibition of *h*AC by **22m** (two independent experiments, each performed in two technical replicates); (D) determination of kinetic parameter *k*_i_/*K*_I_ of **22m** (two independent experiments, each performed in two technical replicates).

We first envisaged that the inhibition of **22m**, belonging to the same class of the benzoxazolone carboxamide **2c**,^39^ should occur through the same covalent AC modification. According to our hypothesis, the corresponding benzoxazolone **21g** (Scheme 4), tested at 1 and 10 μM, was not able to inhibit *h*AC due to the lack of the reactive urea-like functionality. Moreover, kinetic studies on *h*AC-enriched lysates showed that **22m** causes a concentration-dependent reduction in the maximal catalytic velocity of AC (*V*_max_) without influencing the Michaelis–Menten constant (*K*_M_) (Figure 4B and Table S1) and time-dependent inhibition at different **22m** concentrations with *k*_i_/*K*_I_ = 0.02 μM^–1^ min^–1^ and *k*_i_ = 0.15 min^–1^ (Figure 4C,D), suggesting a very fast covalent bond formation to the enzyme.^52,53^

The selectivity of **22m** was evaluated against a set of related lysosomal enzymes. The compound showed only a weak inhibitory effect (IC_50_ = 8.0 μM) on human *N*-acylethanolamine acid amidase (*h*NAAA), a lysosomal cysteine amidase that shares 33–34% sequence identity and a very similar reactive site to AC.^54^**22m** had no effect at the concentrations tested (1 and 10 μM) on the activity of either acid sphingomyelinase (ASM) and GCase. We next assessed the selectivity of **22m** against two of the most representative members of serine hydrolases, human fatty acid amide hydrolyase (FAAH)^55^ and monoacylglycerol lipase (MAGL):^56^**22m** showed inhibitory activity on FAAH with an IC_50_ of 0.070 μM and no effect on monoacylglycerol lipase (MAGL) at the concentrations tested (1 and 10 μM). Although off-target activity of **22m** against FAAH is observed, to our knowledge, no evidence for biological cross-talk between the sphingolipid-signaling pathways^2^ and the FAAH-signaling pathway^55,57^ has been reported that could preclude further development of **22m**.

**Figure 5:**
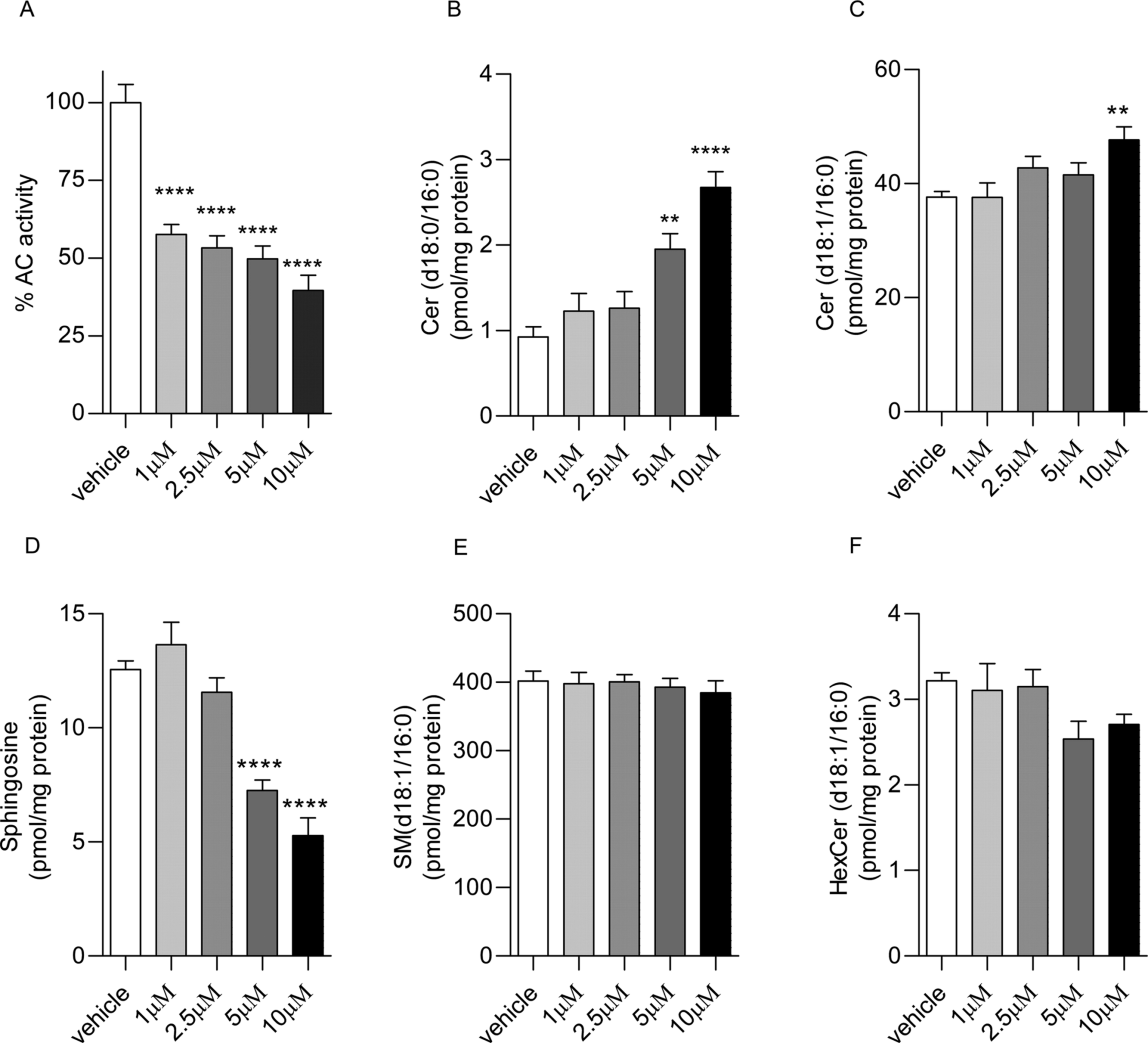
Effects of **22m** in SH-SY5Y cells after a 3 h of incubation. Concentration dependence of the effects on AC activity (A) and sphingolipid levels (B–F). GraphPad Prism software (GraphPad Software, Inc., USA) was used for statistical analysis. Data were analyzed using the Student *t* test or one-way ANOVA followed by the Bonferroni post hoc test for multiple comparisons. Differences between groups were considered statistically significant at values of *p* < 0.05. Values are expressed as means ± S.E.M of at least six determinations. Experiments were repeated twice with similar results.

**Figure 6:**
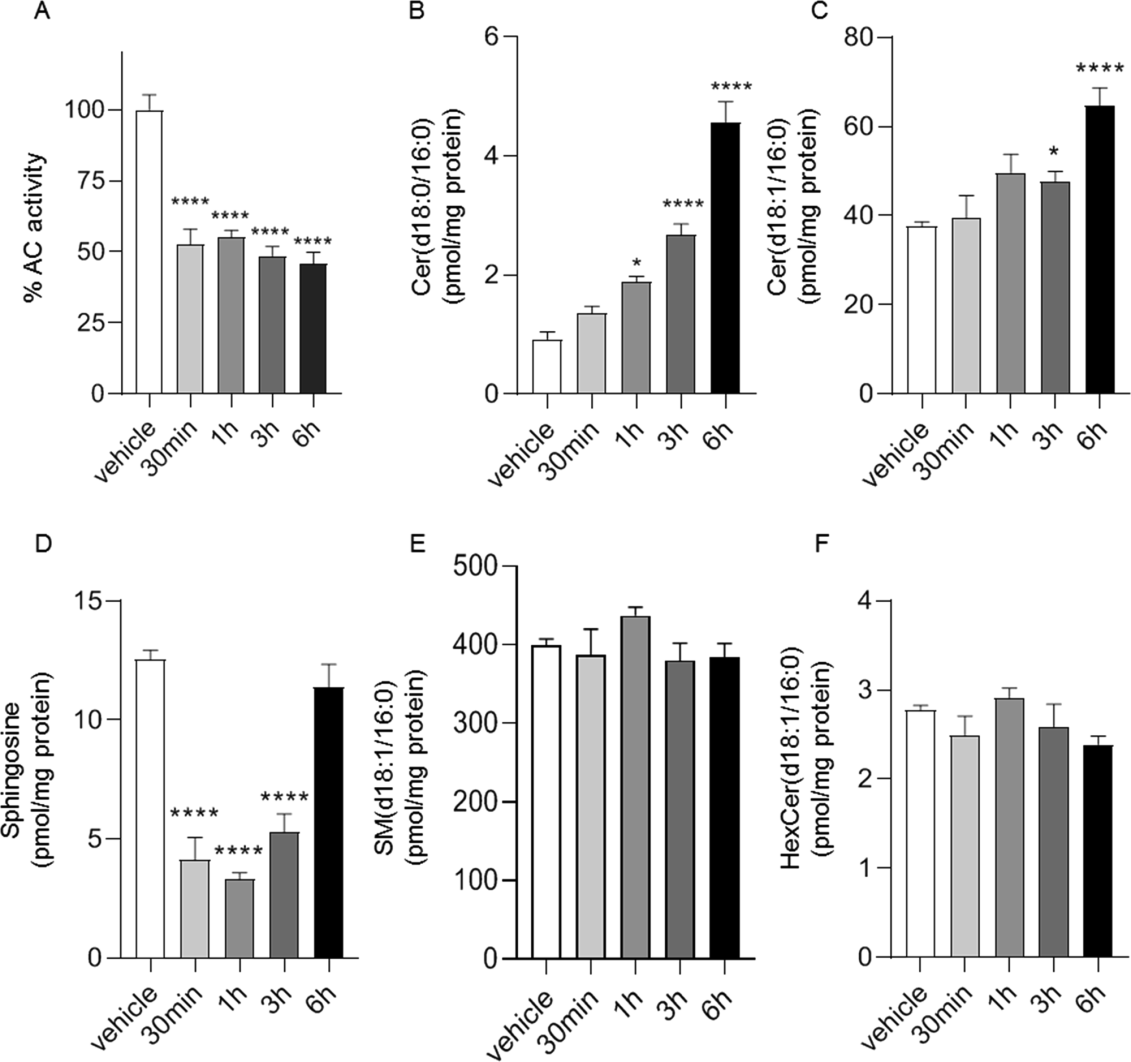
Time course of the effects of **22m** (10 μM) in SH-SY5Y cells on AC activity (A) and sphingolipid levels (B–F). GraphPad Prism software (GraphPad Software, Inc., USA) was used for statistical analysis. Data were analyzed using the Student *t* test or one-way ANOVA followed by the Bonferroni post hoc test for multiple comparisons. Differences between groups were considered statistically significant at values of *p* < 0.05. Values are expressed as means ± S.E.M of at least six determinations. Experiments were repeated twice with similar results.

The favorable overall profile of **22m** prompted us to test its ability to inhibit AC in intact cells. Human neuroblastoma SH-SY5Y cells were incubated in the presence of **22m** at different doses (1, 2.5, 5, and 10 μM). AC activity was measured with a liquid chromatography/mass spectrometry (LC/MS)-based activity assay after different incubation times (30 min, 1 h, 3 h, and 6 h), and SphL levels were identified and quantified by LC/MS, showing that **22m** effectively engages AC in these cells leading to the expected variations in the SphL levels, as reported in Figures 5 and 6. Treatment of cultures of human neuroblastoma SH-SY5Y cells with **22m** caused a concentration- (Figure 5A) and time-dependent reduction of AC activity (Figure 6A). After 3 h of incubation, this effect resulted in an intracellular accumulation of various ceramide species, including Cer (d18:0/16:0) and Cer (d18:1/16:0) (Figure 5B,C) and a corresponding decrease in the levels of sphingosine (Figure 5D) in a concentration-dependent manner. The effect of **22m** (10 μM) on AC activity inhibition and SphL persisted for up to 6 h under our experimental conditions (Figure 6B–D). The results indicated that **22m** inhibits AC in the complex cellular environment leading to an increased Cer (d18:0/16:0) and Cer (d18:1/16:0) (Figure 6B,C) and decreased sphingosine levels with a partial recovery of sphingosine levels after 3–6 h (Figure 6D). Conversely, as expected, no major variations were observed in the levels of sphingomyelin (SM) (d18:1/16:0) (Figures 5E and 6E) and hexosylceramide (HexCer) (d18:1/16:0) (Figures 5F and 6F).

Pharmacokinetic studies of **22m** were determined in CD1 mice, and relevant pharmacokinetic parameters are reported in Table 6. Values of plasma clearance (Cl_p_), volume of distribution (Vd_ss_), and plasma half-life (*t*_1/2_) were calculated after intravenous administration of **22m** at 3 mg kg^–1^. Clearance was moderately high (14.1 L h^–1^ kg^–1^), with a relatively short plasma half-life (1 h) and high Vd_ss_ (12.5 L kg^–1^) indicating that **22m** well distributed out of the circulating plasma compartment. Good oral bioavailability was observed dosing **22m** at 10 mg kg^–1^ (*F* = 58%), with significant exposures in plasma, brain, and cerebrospinal fluid (CSF) (AUC values = 412, 14648, and 119 (h × ng mL^–1^), respectively). A maximum tolerated dose (MTD) study in mice was also conducted in the same background as the pharmacodynamic model using C57BL/6 mice at intraperitoneal dose escalation of 20, 40, 80, and 120 mg kg^–1^ in the time range of 4 days, and no clinical abnormalities were observed in any animals within the doses and time range used.

**Table 6:** Pharmacokinetic Properties of **22m** after Intravenous (A, 3 mg kg^–1^, *N* = 18) and Oral Administration (B, 10 mg kg^–1^, *N* = 18) in Male CD1 Mice

A			
parameter (3mpk, i.v.)	plasma	brain	CSF
*t*_max_ (h)	-	0.250	0.250
*C*_max_ (ng mL^–1^)	-	6443	71.6
*t*_1/2_ (h)	1.26	1.01	0.661
Cl (L h^–1^ kg^–1^)	14.1	-	-
Vd_ss_ (L kg^–1^)	12.5	-	-
AUC (h × ng mL^–1^)	212	10128	77.8

B			
parameter (10mpk, p.o.)	plasma	brain	CSF
*t*_max_ (h)	0.5	1.00	1.00
*C*_max_ (ng mL^–1^)	216	6900	52.2
*t*_1/2_ (h)	1.03	1.18	1.23
AUC (h × ng mL^–1^)	412	14648	119
*F* (%)	58.3	-	-

**Figure 7:**
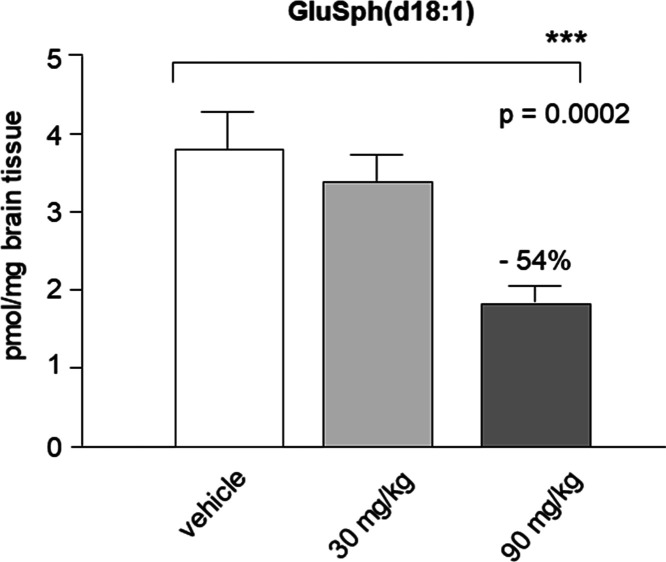
Dose response reduction of brain levels of GluSph (d18:1) after intraperitoneal injection of **22m** at 30 and 90 mg kg^–1^ in 4L;C* mice (*N* = 4–8 with mixed males and females for each group).

Based on these results, we decided to study the effect of dosing **22m** in 4L;C* mice, a validated genetic mutated animal model for neuropathic GD.^58^ 4L;C* mice have a marked increase (20- to 30-fold) of GluSph and moderate elevation (1.5- to 3-fold) of GluCer in the brain; therefore, they are a unique model suitable for testing GluSph reduction therapy. **22m** was administered at selected doses of 30 and 90 mg kg^–1^ by intraperitoneal injection (i.p.) once a day for 14 days starting at postnatal day 5. Preliminary results showed that compound **22m** significantly reduces GluSph (d18:1) in the brain of 4L;C* mice in a dose-dependent manner (Figure 7). Target engagement was demonstrated at a high dose of 90 mg kg^–1^ with 54% reduction of the GluSph levels relative to control.

**Figure 8:**
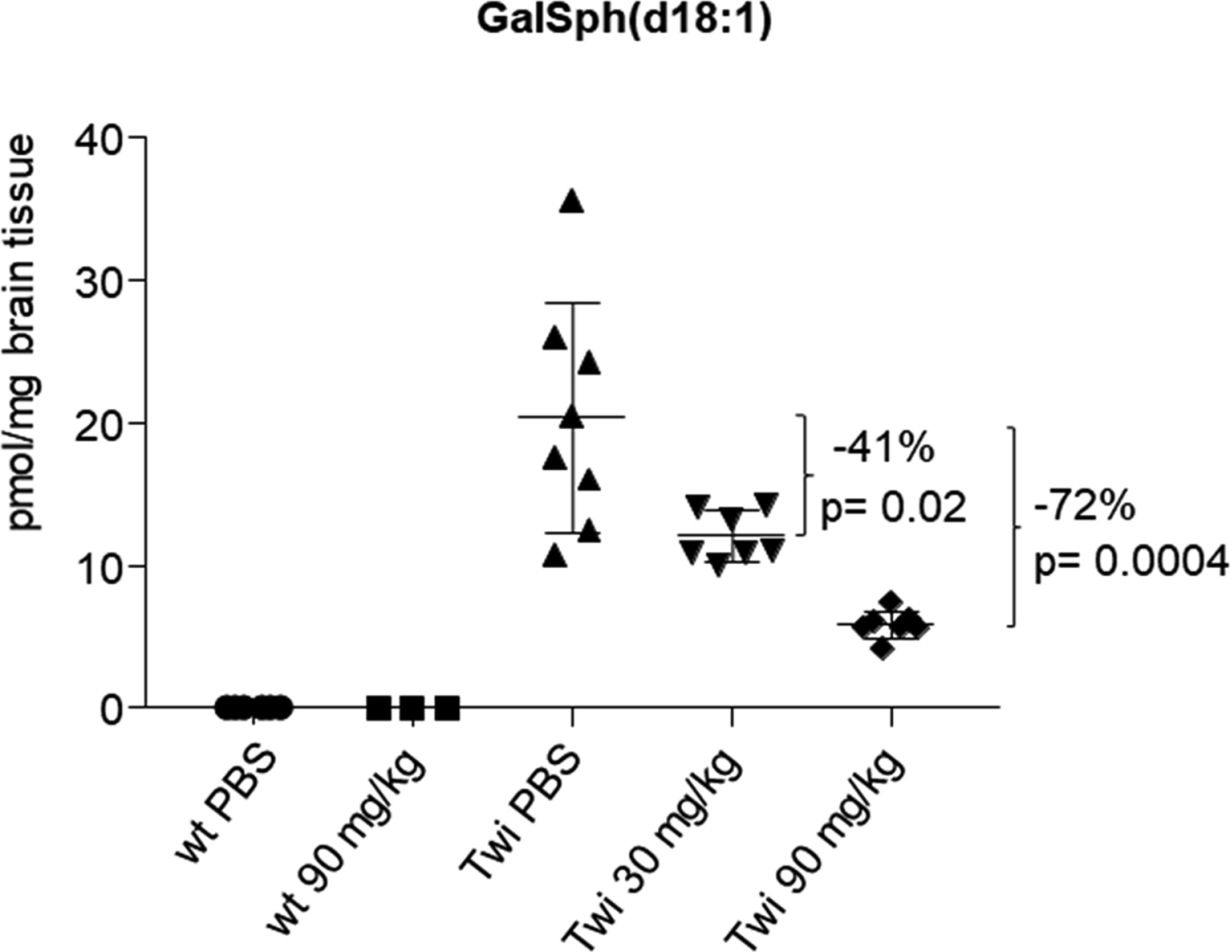
Dose response reduction of brain levels of GalSph (d18:1) after intraperitoneal injection of **22m** at 30 and 90 mg kg^–1^ in Twitcher (Twi) mice (*N* = 3 males + *N* = 3 females for each group).

Next, we evaluated **22m** in the Twitcher mouse, an animal model of Krabbe’s disease. The Twitcher mice naturally carry a GALC mutation that contains a premature stop codon in GALC and leads to a complete loss of GALC activity. As a result, a dramatic increase of the extremely toxic lipid GalSph is observed in Twitcher mouse brains. After i.p. administration at 30 and 90 mg kg^–1^ once daily for a treatment period of 20 days starting at postnatal day 10, **22m** showed dose-dependent reduction of the toxic lipid GalSph (d18:1) levels in the brains of Twitcher mice by 72 and 41% at high and low doses, respectively (Figure 8).

In the group of 4L;C* mice at 90 mg kg^–1^ doses, the unbound drug level in the brain 1 h post last dose (day 14) is 2.6 μM (6.4-fold higher than the EC_50_ value) (Table 7), while at a lower dose of 30 mg kg^–1^, the unbound drug level is 0.77 μM (1.9-fold higher than the EC_50_ value). In the group of Twitcher mice at 90 mg kg^–1^ doses, the unbound drug level in the brain 1 h post last dose (day 20) is 2.15 μM (5.2-fold higher than the EC_50_ value), while at a lower dose of 30 mg kg^–1^, the unbound drug level is 0.80 μM (2.0-fold higher than the EC_50_ value). Overall, these data support the observed dose responses in the two animal models.

**Table 7:** Plasma and Brain Concentrations of **22m** in 4L;C* and Twitcher Mice

EC_50_ (μM)^a^	Fp, u (%)^b^	Fb, u (%)^c^	mouse model	dose (mg kg^–1^)	Cp (μM)^d^	Cp,u (μM)^e^	Cb (μM)^f^	Cb, u (μM)^g^
0.410 ± 0.100	13.8	0.70	4L;C*^h^	90	16.81	2.32	373.34	2.61
				30	3.08	0.42	110.12	0.77
			Twitcher^i^	90	3.85	0.52	307.92	2.15
				30	0.85	0.11	114.50	0.80

a EC_50_ value as a mean of two independent experiments, each performed in two technical replicates. Primary fibroblast cells from Krabbe’s disease patients were incubated with **22m** for 2 h at different concentrations.

b Fp, u: plasma fraction unbounded. Values are the mean of two technical replicates.

c Fb, u: brain fraction unbounded. Values are the mean of two technical replicates.

d Cp: plasma concentration.

e Cp, u: plasma unbounded concentration.

f Cb: brain concentration.

g Cb, u: brain unbounded concentration.

h 4L;C* mice were sacrificed 1 h after the last doses (day 14), and the compound **22m** levels were measured in plasma and brain (*N* = 4–8 with mixed males and females).

i Twitcher mice were sacrificed 1 h after the last doses (day 20), and the compound **22m** levels were measured in plasma and brain (*N* = 3 males + *N* = 3 females for each group).

To our knowledge, this is the first report showing the efficacy of inhibiting AC on reducing the neurotoxic lipids GluSph in the brains of 4L;C* mice. Our result that inhibiting AC reduces neurotoxic lipid GalSph levels in the brains of Twitcher mice is consistent with the recent report.^34^

Further pharmacological studies of **22m** will be reported in due course.

## Conclusions

The present work outlines the lead optimization studies of a class of benzoxazolone carboxamides as AC inhibitors. We further extended the preliminary studies around **2b** (and **2c**)^39,40^ and performed a focused structure–activity relationship (SAR) study on *Regions A* and *B* of this scaffold with the aim of improving the physicochemical and metabolic properties of the series while maintaining the inhibitory potency. Introduction of different heterocyclic groups on the benzoxazolone moiety was tolerated regarding inhibitory potency, as for the tetrahydropyrane **22b**, the piperidines **22d** and **23a**, and the piperazines **23e** and **23f**. A more focused exploration around **22d** and **23e** by changing the nature of substitution on the distal nitrogen atom led to the identification of novel potent analogs with improved solubility, for example, the piperidines **22e** and **22f**. Targeted modifications on different positions of *Regions A* and *B* of the *N*-methylated piperidine series led to compound **22m** as a potent and oral bioavailable AC inhibitor with excellent brain penetration in mice. Preliminary results demonstrated target engagement of **22m** both in the 4L;C* and Twitcher mouse models where dose-dependent reductions in GluSph and GalSph were observed, supporting that further optimized AC inhibitors may be used in the correction of severe pathological neurological states of LSD where these toxic lipids may play a significant role in the pathology, such as GD and KD.

## Experimental Section

### Chemicals, Materials, and Methods

Solvents and reagents were obtained from commercial suppliers and were used without further purification. Automated column chromatography purifications were done using a Teledyne ISCO apparatus (CombiFlash Rf) with prepacked SiO_2_ columns of different sizes (from 4 to 40 g). Mixtures of increasing polarity of Cy and EtOAc or DCM and MeOH were used as eluents. TLC analyses were performed using a Supelco on TLC Al foils 0.2 mm with a fluorescence indicator at 254 nm. Purifications of basic compounds were done using an IST ISOLUTE SCX packed into SPE cartridges (SCX). Hydrogenation reactions were performed using H-Cube continuous hydrogenation equipment (SS-reaction line version), employing disposable catalyst cartridges (CatCart) preloaded with the required heterogeneous catalyst. Microwave heating was performed using an Explorer-48 positions instrument (CEM). NMR experiments of all the intermediates and final compounds were run on a Bruker Avance III 400 system (400.13 MHz for ^1^H and 100.62 MHz for ^13^C) equipped with a BBI probe and Z-gradients. Spectra were acquired at 300 K using deuterated dimethylsulfoxide (DMSO-*d*_6_) or deuterated chloroform (CDCl_3_) as solvent. Chemical shifts for ^1^H and ^13^C spectra were recorded in parts per million using the residual non-deuterated solvent as the internal standard (for DMSO-*d*_6_: 2.50 ppm, ^1^H; 39.52 ppm, ^13^C; for CDCl_3_: 7.26 ppm, ^1^H and 77.16 ppm, ^13^C). Data are reported as follows: chemical shift (ppm), multiplicity (indicated as bs, broad singlet; s, singlet; d, doublet; t, triplet; q, quartet; p, quintet, sx, sextet; m, multiplet, and combinations thereof), coupling constants (*J*) in hertz (Hz), and integrated intensity. Quantitative ^1^H-NMR analyses of the freshly prepared 10 mM DMSO-*d*_6_ stock solutions (used for biological screenings) of the final compounds were performed using the PULCON method (PUlse Length based CONcentration determination, Bruker software, topspin 3.0. References: (a) Wider G., Reires L. *J. Am. Chem. Soc*. **2006**, *128* (8), 2571–2576; (b) Burton I. W., Quilliam M. A., Valter J. A., *Anal. Chem*. **2005**, *77*, 3123–3131). UPLC/MS analyses of all the intermediates and final compounds were performed on a Waters ACQUITY UPLC/MS system consisting of a Single Quadrupole Detector (SQD) Mass Spectrometer (MS) equipped with an Electrospray Ionization (ESI) interface and a Photodiode Array Detector (PDA). The PDA range was 210–400 nm. Analyses were performed on an ACQUITY UPLC BEH C18 column (50 × 2.1 mm ID, particle size 1.7 μm) with a VanGuard BEH C18 precolumn (5 × 2.1 mm ID, particle size 1.7 μm). The mobile phase was 10 mM NH_4_OAc in H_2_O at pH 5 adjusted with AcOH (A) and 10 mM NH_4_OAc in MeCN/H_2_O (95:5) at pH 5 (B). ESI in both positive and negative modes was used in the mass scan range of 100–650 Da. Analyses were performed with *method A*, *B*, *C*, or *D*. *Method A*: gradient 5 to 95% B over 2.5 min. Flow rate 0.5 mL min^–1^. Temperature 40 °C. *Method B*: gradient 50 to 100% B over 2.5 min. Flow rate 0.5 mL min^–1^. Temperature 40 °C. *Method C*: gradient 0 to 100% B over 2.5 min. Flow rate 0.5 mL min^–1^. Temperature 40 °C. *Method D*: isocratic 55% B over 5 min. Flow rate 0.5 mL min^–1^. Temperature 40 °C. UPLC/MS analyses of the final compounds were performed with *method E* or *F* using freshly prepared 10 mM DMSO-*d*_6_ stock solutions (used for biological screenings), diluted 20-fold or 100 fold in MeCN/H_2_O (1:1), and directly analyzed. An ACQUITY UPLC BEH C18 (100 × 2.1 mm ID, particle size 1.7 μm) with a VanGuard BEH C18 precolumn (5 × 2.1 mm ID, particle size 1.7 μm) was used. The mobile phase was 10 mM NH_4_OAc in H_2_O at pH 5 adjusted with AcOH (A) and 10 mM NH_4_OAc in MeCN/H_2_O (95:5) at pH 5 (B). ESI in both positive and negative modes was used in the mass scan range of 100–650 Da. *Method E*: gradient: 10 to 90% B over 6 min. Flow rate 0.5 mL min^–1^. Temperature 40 °C. *Method F*: gradient: 50 to 100% B over 6 min. Flow rate 0.5 mL min^–1^. Temperature 40 °C. The detection wavelength (λ) was set at 215 nm for relative purity determination. *R_t_* of the final compounds are reported in Table S2. Accurate mass measurements were performed on a Synapt G2 Quadrupole-ToF Instrument (Waters, USA) equipped with an ESI ion source; compounds were diluted to 50 μM in H_2_O/MeCN and analyzed. Leucine enkephalin (2 ng mL^–1^) was used as a lock mass reference compound for spectral recalibration. All final compounds displayed ≥95% purity as determined by NMR and UPLC/MS analysis.

### General Procedure for Palladium-Catalyzed Cross Coupling Reaction (Procedure A)

To a solution of the appropriate phenyl bromide (1.0 equiv.) in dry 1,4-dioxane (0.5 M, previously degassed under a nitrogen atmosphere) was added the appropriate boronic acid or its corresponding boronic ester (1.1 equiv.) followed by the addition of Pd(PPh_3_)_4_ or Pd(dppf)Cl_2_ (0.05–0.2 equiv.) and 2 M Na_2_CO_3_ (2.5 equiv.). The dark reaction mixture was stirred at reflux for 15 h, then diluted with EtOAc, and filtered through a pad of Celite. The filtrate was concentrated under reduced pressure, and the crude was purified by column chromatography, eluting with Cy/EtOAc as indicated in each case.

### General Procedure for Catalytic Hydrogenation Reaction (Procedure B)

#### Method A

To a suspension of the appropriate 2-nitrophenol (1.0 equiv.) in MeOH, EtOH, or EtOAc (0.4 M) were added 10% Pd/C (0.25 equiv.) and cyclohexene (30 equiv.), and the reaction mixture was stirred at reflux until the disappearance of the starting material, as indicated by UPLC/MS analysis. The suspension was filtered through a pad of Celite, and the filtrate was quickly evaporated under reduced pressure. The crude was used in the next step without further purification.

#### Method B

A suspension of the appropriate 2-nitrophenol (1.0 equiv.) in MeOH (0.4 M) was hydrogenated with the H-Cube apparatus using 10% Pd/C at 60 °C and full H_2_ mode. After complete conversion (UPLC/MS analysis monitoring), the solvent was evaporated under reduced pressure. The crude was used in the next step without further purification.

### General Procedure for Intramolecular Cyclization Using CDI (Procedure C)

To a solution of the appropriate 2-aminophenol (1.0 equiv.) in MeCN (0.1 M) was added CDI (1.0–1.5 equiv.). The reaction mixture was stirred at rt for 2 h. Then the solvent was evaporated under reduced pressure, and the crude was redissolved in EtOAc, washed with H_2_O and brine, and dried over Na_2_SO_4_. After evaporation of the solvent, the crude was purified by column chromatography, eluting with Cy/EtOAc or DCM/MeOH, or used in the next step without further purification, as indicated in each case.

### General Procedure for Carboxamide Synthesis (Procedure D)

#### Method A

To a stirred solution of the appropriate oxazolone (1.0 equiv.) and DMAP (1.1 equiv.) in dry MeCN was added the appropriate isocyanate (1.1–3.0 equiv.). The reaction mixture was stirred at rt for 30 min under a nitrogen atmosphere. After evaporation of the solvent, the crude was purified by column chromatography, eluting with Cy/EtOAc or DCM/MeOH as indicated in each case.

#### Method B

To a stirred solution of triphosgene (0.33 equiv.) in dry DCM (0.2 M) were added the appropriate amine (1.5–3.0 equiv.) and dry Et_3_N (3.0 equiv.) at −15 °C. The resulting mixture was stirred at rt for 30 min under a nitrogen atmosphere and then added to a solution of the appropriate oxazolone (1.0 equiv.) and Et_3_N (1.0 equiv.) in dry DCM. The reaction mixture was stirred at rt for 30 min under nitrogen and then diluted with DCM. The organic phase was washed with saturated aqueous NH_4_Cl solution and brine and dried over Na_2_SO_4_. After evaporation of the solvent, the crude was purified by column chromatography, eluting with Cy/EtOAc or DCM/MeOH, as indicated in each case.

#### Method C

To a stirred solution of Boc_2_O (2.0 equiv.) in MeCN (0.4 M) were added DMAP (2.0 equiv.) and the appropriate amine (2.0 equiv.). The resulting solution was stirred at rt for 10 min, then the appropriate oxazolone derivative (1.0 equiv.) was added, and the mixture was stirred at rt for 1 h. After evaporation of the solvent, the crude was purified by flash column chromatography, eluting with Cy/EtOAc or DCM/MeOH, as indicated in each case.

### General Procedure for N-Boc Removal (Procedure E)

To a suspension of the appropriate N-Boc-protected derivative (1.0 equiv.) in 1,4-dioxane or DCM (0.1 M) was added HCl (30 equiv., 4 M in 1,4-dioxane), and the reaction mixture was stirred at rt for 2 h. After evaporation of the solvent, the crude was triturated with Et_2_O or used in the next step without further purification, as indicated in each case.

### General Procedure for Reductive Amination Reaction (Procedure F)

To a solution of the appropriate secondary amine (1.0 equiv.) in MeCN or THF (0.1 M) were added the appropriate aldehyde or ketone (1.6–5.0 equiv.), AcOH (1.6–5.0 equiv.), and NaBH(OAc)_3_ (1.6–3.0 equiv.). The mixture was stirred at rt for 2–16 h under a nitrogen atmosphere. Then the reaction mixture was poured into saturated aqueous NaHCO_3_ solution and extracted with EtOAc. The organic phase was washed with brine and dried over Na_2_SO_4_. After evaporation of the solvent, the crude was purified by SCX.

### General Procedure for Nucleophilic Aromatic Substitution Reaction (SNAr) (Procedure G)

To a solution of the appropriate 4-fluoronitrobenzene (1.0 equiv.) in MeCN were added the appropriate amine (2.0 equiv.) and DIPEA (2.0 equiv.). The reaction mixture was refluxed (or stirred under MW irradiation, 90 °C, power 200 W) until the disappearance of the starting material, as indicated by UPLC/MS analysis. After evaporation of the solvent, the crude was purified by flash column chromatography, eluting with Cy/EtOAc or DCM/MeOH, as indicated in each case.

### General Procedure for Intramolecular Cyclization under Basic Conditions (Procedure H)

To a solution of the appropriate thiazolidinedione derivative (1.0 equiv.) in dry THF (0.1 M) was added *t*BuOK (2.0–4.0 equiv.) at rt under a nitrogen atmosphere. After 30 min, the reaction mixture was diluted with EtOAc, washed with saturated aqueous NH_4_Cl solution and brine, and dried over Na_2_SO_4_. After evaporation of the solvent, the crude was used in the next step without further purification.

### General Procedure for Lithium/Halogen Exchange - Addition Reaction (Procedure I)

To a solution of the appropriate bromobenzoxazolone (1.0 equiv.) in dry THF (0.1 M) was added MeMgBr (1.5 equiv., 3.0 M in Et_2_O) at −78 °C under a nitrogen atmosphere for 30 min followed by the addition of *n*-BuLi (1.2 equiv., 2.5 M in hexanes). After 30 min, a solution of the appropriate piperidone (1.7 equiv.) in dry THF (0.7 M) was added dropwise at −78 °C under a nitrogen atmosphere, and then the reaction mixture was allowed to warm to rt. After 30 min, the reaction was quenched by addition of saturated aqueous NH_4_Cl solution, diluted with EtOAc, washed with brine, and dried over Na_2_SO_4_. After evaporation of the solvent, the crude was purified by column chromatography, eluting with Cy/EtOAc or DCM/MeOH, as indicated in each case.

### General Procedure for Dehydration Reaction of Tertiary Alcohols (Procedure L)

To a suspension of the appropriate tertiary alcohol (1.0 equiv.) in dry toluene (0.1 M) was added *p*-TsOH (3.0 equiv.), and the reaction mixture was stirred at reflux for 2 h. After evaporation of the solvent, the crude was purified by SCX or used in the next step without further purification, as indicated in each case.

#### Synthesis of *N*-(2-Benzyloxyethyl)-6-(4-fluorophenyl)-2-oxo-1,3-benzoxazole-3-carboxamide (**2d**)

Compound **2d** was prepared according to general procedure D (method B) using 6-(4-fluorophenyl)-3*H*-1,3-benzoxazol-2-one^39^ (0.060 g, 0.26 mmol), 2-(benzyloxy)-1-ethanamine hydrochloride (0.073 g, 0.39 mmol), and Et_3_N (0.11 mL, 0.079 g, 0.78 mmol) in dry DCM (3 mL). The crude was purified by column chromatography (Cy/EtOAc, 80:20) to afford **2d** as a white solid (0.06 g, 57%). ^1^H NMR (600 MHz, CDCl_3_) δ 8.35 (bs, 1H), 8.08 (d, *J* = 8.3 Hz, 1H), 7.52 (dd, *J* = 8.8, 5.2 Hz, 2H), 7.43 (dd, *J* = 8.3, 1.7 Hz, 1H), 7.40 (d, *J* = 1.7 Hz, 2H), 7.39–7.33 (m, 4H), 7.28 (tt, *J* = 7.0, 1.6 Hz, 1H), 7.14 (t, *J* = 8.6 Hz, 2H), 4.59 (s, 2H), 3.74–3.65 (m, 4H). ^13^C NMR (151 MHz, CDCl_3_) δ 162.82 (d, *J*_C–F_ = 247.5 Hz), 153.12, 149.94, 142.43, 137.91, 137.51, 136.18, 136.16, 128.86 (d, *J*_C–F_ = 8.2 Hz), 128.62, 127.96, 127.95, 127.28, 123.87, 116.03 (d, *J*_C–F_ = 21.4 Hz), 115.78, 108.57, 73.40, 68.26, 40.37. UPLC/MS (*method A*): *R_t_* 2.78 min. MS (ES) C_23_H_19_FN_2_O_4_ requires 406, found 407 [M + H]^+^. HRMS C_23_H_20_FN_2_O_4_ [M + H]^+^: calculated 407.1407, measured: 407.1424, Δppm 4.2.

#### Synthesis of 6-(4-Fluorophenyl)-2-oxo-*N*-pentyl-1,3-benzoxazole-3-carboxamide (**2e**)

Compound **2e** was prepared according to general procedure D (method A) using 6-(4-fluorophenyl)-3*H*-1,3-benzoxazol-2-one (0.080 g, 0.35 mmol) and 1-pentyl isocyanate (0.05 mL, 0.040 g, 0.39 mmol) in dry MeCN (3 mL). The crude was purified by column chromatography (Cy/EtOAc, 80:20) to afford **2e** as a white solid (0.100 g, 82%). ^1^H NMR (400 MHz, CDCl_3_) δ. 8.10 (d, *J* = 8.3 Hz, 1H), 8.04 (bs, 1H), 7.52 (dd, *J* = 8.6, 5.3 Hz, 2H), 7.47–7.42 (m, 1H), 7.41–7.38 (m, 1H), 7.14 (t, *J* = 8.6 Hz, 2H), 3.44 (q, *J* = 6.9 Hz, 2H), 1.66 (p, *J* = 7.2 Hz, 2H), 1.45–1.32 (m, 4H), 0.93 (t, *J* = 6.9 Hz, 3H). ^13^C NMR (101 MHz, CDCl_3_) δ 162.70 (d, *J*_C–F_ = 247.6 Hz), 153.23, 149.71, 142.28, 137.38, 136.06, 128.73 (d, *J*_C–F_ = 8.8 Hz), 123.78, 116.00 (d, *J*_C–F_ = 25.3 Hz), 115.75, 108.42, 99.96, 40.34, 29.12, 28.96, 22.30, 13.94. UPLC/MS (*method A*): *R_t_* 2.43 min. MS (ES) C_19_H_19_FN_2_O_3_ requires 342, found 343 [M + H]^+^. HRMS C_19_H_20_FN_2_O_3_ [M + H]^+^: calculated 343.1458, measured: 343.1449, Δppm −2.6.

#### Synthesis of *N*-(2-Ethoxyethyl)-6-(4-fluorophenyl)-2-oxo-1,3-benzoxazole-3-carboxamide (**2f**)

Compound **2f** was prepared according to general procedure D (method B) using 6-(4-fluorophenyl)-3*H*-1,3-benzoxazol-2-one (0.130 g, 0.57 mmol), 2-ethoxyethylamine (0.09 mL, 0.080 g, 0.85 mmol), and Et_3_N (0.20 mL, 0.140 g, 1.42 mmol) in dry DCM (15 mL). The crude was purified by column chromatography (Cy/EtOAc, 80:20) to afford **2f** as a white solid (0.03 g, 13%). ^1^H NMR (400 MHz, CDCl_3_) δ 8.32 (bs, 1H), 8.10 (d, *J* = 8.3 Hz, 1H), 7.57–7.49 (m, 2H), 7.46–7.38 (m, 2H), 7.18–7.10 (m, 2H), 3.68–3.61 (m, 4H), 3.56 (q, *J* = 7.0 Hz, 2H), 1.24 (t, *J* = 7.0 Hz, 3H). ^13^C NMR (101 MHz, CDCl_3_) δ 162.69 (d, *J*_C–F_ = 247.7 Hz), 153.16, 149.98, 142.47, 137.54, 136.20, 128.88 (d, *J*_C–F_ = 8.2 Hz), 127.34, 123.89, 116.04 (d, *J*_C–F_ = 21.7 Hz), 115.81, 108.60, 68.63, 66.81, 40.43, 15.25. UPLC/MS (*method A*): *R_t_* 2.56 min. MS (ES) C_18_H_17_FN_2_O_4_ requires 344, found 345 [M + H]^+^. HRMS C_18_H_18_FN_2_O_4_ [M + H]^+^: calculated 345.1251, measured: 345.1258, Δppm 2.

#### Synthesis of 6-(4-Fluorophenyl)-*N*-(3-methoxypropyl)-2-oxo-1,3-benzoxazole-3-carboxamide (**2g**)

Compound **2g** was prepared according to general procedure D (method B) using 6-(4-fluorophenyl)-3*H*-1,3-benzoxazol-2-one (0.08 g, 0.35 mmol), 3-methoxypropylamine (0.06 mL, 0.050 g, 0.52 mmol), and Et_3_N (0.12 mL, 0.09 g, 0.88 mmol) in dry DCM (15 mL). The crude was purified by column chromatography (Cy/EtOAc, 80:20) to afford **2g** as a white solid (0.020 g, 18%). ^1^H NMR (400 MHz, CDCl_3_) δ 8.35 (bs, 1H), 8.10 (d, *J* = 8.3 Hz, 1H), 7.52 (dd, *J* = 8.6, 5.3 Hz, 2H), 7.43 (dd, *J* = 8.4, 1.4 Hz, 1H), 7.41–7.39 (m, 1H), 7.14 (t, *J* = 8.6 Hz, 2H), 3.55 (dt, *J* = 16.4, 6.0 Hz, 4H), 3.39 (s, 3H), 1.92 (p, *J* = 6.1 Hz, 2H). ^13^C NMR (101 MHz, CDCl_3_) δ 162.67 (d, *J*_C–F_ = 247.4 Hz), 153.16, 142.84, 142.43, 137.44, 136.23, 128.70 (d, *J*_C–F_ = 8.1 Hz, 2C), 127.40, 123.84, 116.12, 115.88 (d, *J*_C–F_ = 11.3 Hz, 2C), 108.53, 70.95, 58.95, 38.60, 29.28. UPLC/MS (*method A*): *R_t_* 2.49 min. MS (ES) C_18_H_17_FN_2_O_4_ requires 344, found 345 [M + H]^+^. HRMS C_18_H_18_FN_2_O_4_ [M + H]^+^: calculated 345.1251, measured: 345.1258, Δppm 2.

#### Synthesis of 3*H*-Oxazolo[4,5-*c*]pyridin-2-one (**7a**)

Compound **7a** was prepared according to general procedure C using **6a** (0.10 g, 0.91 mmol) and CDI (0.290 g, 1.82 mmol, 2.0 equiv.) in a mixture of MeCN/DMF (9 mL, 2:1). The crude was triturated with DCM to afford **7a** as a whitish solid (0.100 g, 80%). ^1^H NMR (400 MHz, DMSO-*d*_6_) δ 8.34 (s, 1H), 8.32 (d, *J* = 5.3 Hz, 1H), 7.38 (d, *J* = 5.3 Hz, 1H). UPLC/MS (*method A*): *R_t_* 0.61 min. MS (ES) C_6_H_4_N_2_O_2_ requires 136, found 137 [M + H]^+^, 135 [M–H]^−^.

#### Synthesis of 1*H*-Oxazolo[5,4-*c*]pyridin-2-one (**7b**)

Compound **7b** was prepared according to general procedure C using **6b** (0.100 g, 0.91 mmol) and CDI (0.441 g, 2.72 mmol) in a mixture of MeCN/DMF (9 mL, 1:4). The crude was triturated with Et_2_O to afford **7b** as a brown solid (0.123 g, quant.). ^1^H NMR (400 MHz, DMSO-*d*_6_) δ 12.51 (bs, 1H), 8.38 (s, 1H), 8.23 (d, *J* = 5.5 Hz, 1H), 7.16 (d, *J* = 5.5 Hz, 1H). UPLC/MS (*method C*): *R_t_* 1.06 min. MS (ES) C_6_H_4_N_2_O_2_ requires 136, found 137 [M + H]^+^, 135 [M–H]^−^.

#### Synthesis of 2-Oxo-*N*-(4-phenylbutyl)oxazolo[4,5-*c*]pyridine-3-carboxamide (**8a**)

Compound **8a** was prepared according to general procedure D (method A) using **7a** (0.03 g, 0.22 mmol) and 4-phenylbutyl isocyanate (0.045 mL, 0.046 g, 0.26 mmol) in a mixture of DMF/toluene (3 mL, 2:1). The crude was purified by column chromatography (Cy/EtOAc, from 95:5 to 70:30) to afford **8a** as a white solid (0.015 g, 41%). ^1^H NMR (400 MHz, CDCl_3_) δ 9.28 (s, 1H), 8.55 (d, *J* = 5.1 Hz, 1H), 7.85 (bs, 1H), 7.31–7.21 (m, overlapped with CDCl_3_ signal, 3H), 7.21–7.13 (m, 3H), 3.47 (q, *J* = 6.5 Hz, 2H), 2.68 (t, *J* = 7.1 Hz, 2H), 1.79–1.64 (m, 4H). ^13^C NMR (101 MHz, CDCl_3_) δ 151.67, 148.95, 148.06, 146.45, 141.91, 136.75, 136.72, 128.53 (4C), 126.06, 105.75, 40.48, 35.54, 29.11, 28.63. UPLC/MS (*method A*): *R_t_* 2.23 min. MS (ES) C_17_H_17_N_3_O_3_ requires 311, found 312 [M + H]^+^. HRMS C_17_H_18_N_3_O_3_ [M + H]^+^: calculated 312.1348, measured: 312.134, Δppm −2.6.

#### Synthesis of 2-Oxo-*N*-(4-phenylbutyl)oxazolo[5,4-*c*]pyridine-1-carboxamide (**8b**)

Compound **8b** was prepared according to general procedure D (method A) using **7b** (0.08 g, 0.59 mmol) and 4-phenylbutyl isocyanate (0.11 mL, 0.113 g, 0.65 mmol) in a mixture of DMF/MeCN (12 mL, 4:1). The crude was purified by column chromatography (Cy/EtOAc, 80:20) to afford **8b** as a white solid (0.107 g, 59%). ^1^H NMR (400 MHz, CDCl_3_) δ 8.58 (s, 1H), 8.54 (d, *J* = 5.3 Hz, 1H), 8.04 (d, *J* = 5.3 Hz, 1H), 7.91 (bs, 1H), 7.33–7.23 (m, overlapped with CDCl_3_ signal, 2H), 7.23–7.13 (m, 3H), 3.46 (q, *J* = 6.4 Hz, 2H), 2.68 (t, *J* = 7.1 Hz, 2H), 1.80–1.61 (m, 4H). ^13^C NMR (101 MHz, CDCl_3_) δ 151.99, 148.79, 146.15, 141.86, 139.86, 130.48, 128.55 (4C), 128.53, 126.10, 110.80, 40.52, 35.54, 29.04, 28.62. UPLC/MS (*method A*): *R_t_* 2.26 min. MS (ES) C_17_H_17_N_3_O_3_ requires 311, found 312 [M + H]+. HRMS C_17_H_18_N_3_O_3_ [M + H]^+^: calculated 312.1348, measured: 312.1341, Δppm −2.2.

#### Synthesis of 2,3-Dioxo-*N*-(4-phenylbutyl)indoline-1-carboxamide (**8c**)

Compound **8c** was prepared according to general procedure D (method A) using **7c** (0.074 g, 0.50 mmol) and 4-phenylbutyl isocyanate (0.097 mL, 0.100 g, 0.55 mmol). The crude was purified by column chromatography (Cy/EtOAc, 85:15) to afford **8c** as a yellow solid (0.029 g, 21%). ^1^H NMR (400 MHz, DMSO-*d*_6_) δ 8.22 (t, *J* = 5.7 Hz, 1H), 8.16 (d, *J* = 8.2 Hz, 1H), 7.75–7.64 (m, 2H), 7.31–7.23 (m, 3H), 7.23–7.13 (m, 3H), 3.39–3.24 (m, overlapped with H_2_O signal, 2H), 2.62 (t, *J* = 7.3 Hz, 2H), 1.68–1.51 (m, 4H). ^13^C NMR (101 MHz, DMSO-*d*_6_) δ 180.90, 159.48, 151.08, 148.69, 142.51, 138.04, 128.70 (4C), 126.14, 125.16, 124.81, 119.47, 116.97, 39.70, 35.23, 29.14, 28.66. UPLC/MS (*method A*): *R_t_* 1.41 min. MS (ES) C_19_H_18_N_2_O_3_ requires 322, found 323 [M + H]^+^. HRMS C_19_H_19_N_2_O_3_ [M + H]^+^: calculated 323.1396, measured: 323.1391, Δppm −1.5.

#### Synthesis of 2-Oxo-*N*-(4-phenylbutyl)indoline-1-carboxamide (**8d**)

Compound **8d** was prepared according to general procedure D (method A) using **7d** (0.066 g, 0.50 mmol) and 4-phenylbutyl isocyanate (0.094 mL, 0.096 g, 0.55 mmol). The crude was purified by column chromatography (Cy/EtOAc, 90:10) to afford **8d** as a white solid (0.04 g, 26%). ^1^H NMR (400 MHz, CDCl_3_) δ 8.60 (bs, 1H), 8.25 (d, *J* = 8.2 Hz, 1H), 7.35–7.22 (m, overlapped with CDCl_3_ signal, 4H), 7.21–7.11 (m, 4H), 3.71 (s, 2H), 3.42 (q, *J* = 6.7 Hz, 2H), 2.67 (t, *J* = 7.3 Hz, 2H), 1.79–1.62 (m, 4H). ^13^C NMR (101 MHz, CDCl_3_) δ 177.17, 152.03, 141.89, 141.69, 128.22, 128.15, 128.11, 125.62, 124.11, 123.61, 122.71, 116.28, 39.52, 36.80, 35.32, 28.96, 28.50. UPLC/MS (*method A*): *R_t_* 2.61 min. MS (ES) C_19_H_20_N_2_O_2_ requires 308, found 309 [M + H]^+^. HRMS C_19_H_21_N_2_O_2_ [M + H]^+^: calculated 309.1603, measured 309.1598, Δppm −1.6.

#### Synthesis of *tert*-Butyl 3-(2,4-dioxothiazolidin-3-yl)-4-oxo-piperidine-1-carboxylate (**10**)

To a solution of **9** (0.782 g, 1.00 mmol, 1.0 equiv.) in dry DMF (5 mL) were added TZD (0.141 g, 1.20 mmol, 1.2 equiv.) and K_2_CO_3_ (0.207 g, 1.50 mmol, 1.5 eq.). The reaction was stirred at rt for 2 h and then diluted with EtOAc. The organic phase was washed with brine, dried over Na_2_SO_4_, and concentrated under reduced pressure to afford **10** as an orange oil (0.247 g, 79%). ^1^H NMR (400 MHz, CDCl_3_) δ 4.80–4.67 (m, 1H), 4.57–4.19 (m, 2H), 4.02 (s, 2H), 3.79–3.58 (m, 1H), 3.30–3.10 (m, 1H), 2.68–2.47 (m, 2H), 1.49 (s, 9H). UPLC/MS (*method A*): *R_t_* 1.88 min. MS (ES) C_13_H_18_N_2_O_5_S requires 314, found 313[M–H]^−^.

#### Synthesis of *tert*-Butyl 2-oxo-3,4,6,7-tetrahydrooxazolo[4,5-*c*]pyridine-5-carboxylate (**11**)

Compound **11** was prepared according to general procedure H using **10** (0.247 g, 0.79 mmol, 1.0 equiv.) and *t*BuOK (0.176 g, 1.57 mmol, 2.0 equiv.) in dry THF (8 mL). The crude was purified by column chromatography (Cy/EtOAc, 80:20) to afford **11** as yellow oil (0.055 g, 29%). UPLC/MS (*method A*): *R_t_* 1.64 min. MS (ES) C_11_H_16_N_2_O_4_ requires 240, found 241 [M + H]^+^.

#### Synthesis of *tert*-Butyl 2-oxo-3-(4-phenylbutylcarbamoyl)-6,7-dihydro-4*H*-oxazolo[4,5-*c*]pyridine-5-carboxylate (**12a**)

Compound **12a** was prepared according to general procedure D (method A) using **11** (0.055 g, 0.23 mmol) and 4-phenylbutyl isocyanate (0.079 mL, 0.081 g, 0.46 mmol, 2.0 equiv.) in dry MeCN (1 mL). The crude was purified by column chromatography (Cy/EtOAc, 90:10) to afford **12a** as an off-white solid (0.070 g, 68%). ^1^H NMR (400 MHz, CDCl_3_) δ 7.98 (bs, 1H), 7.31–7.23 (m, overlapped signals with CDCl_3_, 2H), 7.21–7.11 (m, 3H), 4.64–4.58 (m, 2H), 3.74–3.66 (m, 2H), 3.35 (q, *J* = 6.7 Hz, 2H), 2.64 (q, *J* = 7.6 Hz, 2H), 2.56–2.46 (m, 2H), 1.78–1.58 (m, overlapped with H_2_O signal, 4H), 1.48 (s, 9H). UPLC/MS (*method A*): *R_t_* 2.81 min. MS (ES) C_22_H_29_N_3_O_5_ requires 415, found 416 [M + H]^+^.

#### Synthesis of 2-Oxo-*N*-(4-phenylbutyl)-4,5,6,7-tetrahydrooxazolo[4,5-*c*]pyridine-3-carboxamide Hydrochloride (**12b**)

Compound **12b** was prepared according to general procedure E using compound **12a** (0.065 g, 0.16 mmol). The crude was triturated with Et_2_O to afford **12b** as a yellow solid (0.030 g, 60%). ^1^H NMR (400 MHz, CDCl_3_) δ 10.33 (bs, 2H), 7.82 (t, *J* = 5.6 Hz, 1H), 7.31–7.24 (m, overlapped with CDCl_3_ signal, 2H), 7.21–7.13 (m, 3H), 4.58–4.38 (m, 2H), 3.63–3.45 (m, 2H), 3.33 (q, *J* = 6.5 Hz, 2H), 2.99–2.87 (m, 2H), 2.65 (t, *J* = 7.3 Hz, 2H), 1.74–1.53 (m, 4H). ^13^C NMR (151 MHz, CDCl_3_) δ 152.81, 149.01, 141.92, 133.04, 128.54 (4C), 126.04, 114.29, 40.31, 35.53, 29.06, 28.63, 19.24. MS UPLC/MS (*method A*): *R_t_* 1.83 min. MS (ES) C_17_H_21_N_3_O_3_ requires 315, found 316 [M + H]^+^. HRMS C_17_H_22_N_3_O_3_ [M + H]^+^: calculated 316.1661, measured: 316.1661, Δppm 0.0.

#### Synthesis of 5-Methyl-2-oxo-*N*-(4-phenylbutyl)-6,7-dihydro-4*H*-oxazolo[4,5-*c*]pyridine-3-carboxamide Hydrochloride (**12c**)

Compound **12c** was prepared according to general procedure F using compound **12b** (0.030 g, 0.09 mmol), 37% aqueous solution of formaldehyde (0.005 mL, 0.18 mmol), NaBH(OAc)_3_ (0.381 g, 1.80 mmol), and AcOH (0.008 mL, 0.008 g, 0.14 mmol) in dry MeCN (1.0 mL). The crude was dissolved in DCM (1 mL) followed by the addition of HCl (0.68 mL, 2.70 mmol, 4 M in 1,4-dioxane). After evaporation of the solvent, the residue was triturated with Et_2_O to afford **12c** as a white solid (0.026 g, 90%). ^1^H NMR (400 MHz, CDCl_3_) δ 13.62 (bs, 1H), 7.80 (t, *J* = 5.5 Hz, 1H), 7.31–7.25 (m, overlapped with CDCl_3_ signal, 2H), 7.21–7.14 (m, 3H), 4.71 (d, *J* = 16.0 Hz, 1H), 4.14–4.01 (m, 1H), 3.75–3.60 (m, 1H), 3.54–3.39 (m, 1H), 3.34 (p, *J* = 6.3 Hz, 2H), 3.28–3.14 (m, 1H), 2.96 (s, 3H), 2.64 (t, *J* = 7.3 Hz, 2H), 1.74–1.51 (m, 4H). ^13^C NMR (101 MHz, CDCl_3_) δ 152.71, 149.00, 141.89, 132.64, 128.52 (4C), 126.04, 113.48, 50.56 (2C), 49.25 (2C), 43.26, 40.32, 35.52, 29.03 (2C), 28.60 (2C), 19.49. UPLC/MS (*method A*): *R_t_* 2.13 min. MS (ES) C_18_H_23_N_3_O_3_ requires 329, found 330[M + H]^+^. HRMS C_18_H_24_N_3_O_3_ [M + H]^+^: calculated 330.1818, measured: 330.182, Δppm 0.6.

#### Synthesis of 5-Benzyl-2-oxo-*N*-(4-phenylbutyl)-6,7-dihydro-4*H*-oxazolo[4,5-*c*]pyridine-3-carboxamide (**12d**)

Compound **12d** was prepared according to general procedure F using compound **12b** (0.050 g, 0.16 mmol), benzaldehyde (0.033 mL, 0.32 mmol), NaBH(OAc)_3_ (0.054 g, 0.26 mmol), and AcOH (0.015 mL, 0.015 g, 0.26 mmol) in dry MeCN (2 mL). The crude was purified by column chromatography (Cy/EtOAc, 85:15) to afford **12d** as a white solid (0.043 g, 68%). ^1^H NMR (400 MHz, CDCl_3_) δ 8.01 (bs, 1H), 7.41–7.23 (overlapped with CDCl_3_ signal, m, 7H), 7.21–7.13 (m, 3H), 3.85–3.64 (m, 4H), 3.32 (q, *J* = 6.8 Hz, 2H), 2.89–2.76 (m, 2H), 2.64 (t, *J* = 7.4 Hz, 2H), 2.58–2.43 (m, 2H), 1.82–1.52 (m, 4H). ^13^C NMR (101 MHz, CDCl_3_) δ 149.72, 142.06, 128.68 (2C), 128.53 (3C), 128.49 (4C), 125.99, 61.29, 48.43 (2C), 40.07, 35.58, 29.18, 28.67, 21.84. UPLC/MS (*method A*): *R_t_* 1.98 min. MS (ES) C_24_H_27_N_3_O_3_ requires 405, found 406 [M + H]^+^. HRMS C_24_H_28_N_3_O_3_ [M + H]^+^: calculated 406.2131, measured: 406.2126, Δppm −1.2.

#### Synthesis of 5-Acetyl-2-oxo-*N*-(4-phenylbutyl)-6,7-dihydro-4*H*-oxazolo[4,5-*c*]pyridine-3-carboxamide (**12e**)

To a solution of **12b** (0.030 g, 0.09 mmol) in dry DCM (0.9 mL) were added Et_3_N (0.025 mL, 0.018 g, 0.18 mmol, 2.0 equiv.) and acetyl chloride (0.008 g, 0.10 mmol, 1.1 equiv.) at 0 °C. The reaction mixture was stirred at rt for 3 h and then was diluted with EtOAc, washed with saturated aqueous NH_4_Cl solution and brine, and dried over Na_2_SO_4_. After evaporation of the solvent, the crude was triturated with Et_2_O to afford **12e** as a white solid (0.018 g, 56%). ^1^H NMR (400 MHz, CDCl_3_) δ 8.07–7.85 (m, 1H), 7.31–7.24 (m, overlapped with CDCl_3_ signal, 2H), 7.21–7.13 (m, 3H), 4.87–4.58 (m, 2H), 3.96–3.64 (m, 2H), 3.36 (q, *J* = 6.6 Hz, 2H), 2.65 (t, *J* = 7.2 Hz, 2H), 2.61–2.49 (m, 2H), 2.17 (s, 3H), 1.76–1.58 (m, 4H). ^13^C NMR (101 MHz, CDCl_3_) δ 128.52 (4C), 126.04, 43.33, 40.19, 38.26, 35.56, 29.16, 28.64, 22.23, 21.66. UPLC/MS (*method A*): *R_t_* 2.12 min. MS (ES) C_19_H_23_N_3_O_4_ requires 357, found 358 [M + H]^+^.

#### Synthesis of *tert*-Butyl 4-(2,4-Dioxothiazolidin-3-yl)-3-hydroxy-piperidine-1-carboxylate (**14a**)

To a solution of **13** (0.220 g, 1.10 mmol, 1.2 equiv.) in dry DMF (2 mL) were added TZD (0.100 g, 0.89 mmol, 1 equiv.) and magnesium perchlorate (0.040 g, 0.18 mmol, 0.2 equiv.). The reaction mixture was stirred at rt for 20 min and then gradually heated to 115 °C over 2 h. After 3 h, the reaction was cooled, diluted with EtOAc, washed with H_2_O, brine, and 15% LiCl in H_2_O, and dried over Na_2_SO_4_. After evaporation of the solvent, the crude was purified by column chromatography (Cy/EtOAc, 60:40) to afford **14a** as a white solid (0.139 g, 50%). ^1^H NMR (400 MHz, DMSO-*d*_6_) δ 5.37 (d, *J* = 3.3 Hz, 1H), 4.13 (d, *J* = 4.7 Hz, 2H), 4.11–3.86 (m, 4H), 2.88–2.61 (m, 1H), 2.06 (qd, *J* = 12.7, 4.7 Hz, 1H), 1.65–1.53 (m, 1H), 1.40 (s, 9H). UPLC/MS (*method A*): *R_t_* 1.77 min, MS (ES) C_13_H_20_N_2_O_5_S requires 316, found 315 [M–H]^−^.

#### Synthesis of 3-(1-Methyl-3-oxo-4-piperidyl)thiazolidine-2,4-dione (**15**)

To a solution of **14a** (0.100 g, 0.32 mmol) in dry DCM (3 mL) was added portionwise Dess–Martin periodinane (0.300 g, 0.70 mmol, 2.2 equiv.) under an argon atmosphere. The reaction was stirred at rt for 16 h, and then saturated aqueous NaHCO_3_ solution was added followed by the addition of 10% Na_2_SO_3_ in H_2_O. The mixture was stirred at rt for 30 min, and then the organic phase was separated, and the aqueous layer was extracted with DCM (3 times). The collected organic phases were dried over Na_2_SO_4_, and the solvent was removed under reduced pressure. The crude was purified by column chromatography (Cy/EtOAc, 75:15) to afford **15** as a white solid (0.070 g, 70%). ^1^H NMR (400 MHz, CDCl_3_) δ 4.80 (dd, *J* = 12.3, 6.6 Hz, 1H), 4.40 (d, *J* = 18.5 Hz, 1H), 4.24–3.91 (m, 3H), 3.29–3.49 (m, 1H), 2.63 (qd, *J* = 12.4, 5.1 Hz, 1H), 2.16–2.04 (m, 1H), 1.51–1.49 (m, 1H), 1.48 (s, 9H). UPLC/MS (*method A*): *R_t_* 1.83 min, MS (ES) C_13_H_18_N_2_O_5_S requires 314, found 315 [M + H]^+^.

#### Synthesis of *tert*-Butyl 2-Oxo-1,4,6,7-tetrahydrooxazolo[5,4-*c*]pyridine-5-carboxylate (**16**)

Compound **16** was prepared according to general procedure H using **15** (0.070 g, 0.22 mmol) and *t*BuOK (0.190 g, 0.89 mmol, 4.0 equiv.) in dry THF (2 mL). The crude was used in the next step without further purification. UPLC/MS (*method A*): *R_t_* 1.64 min. MS (ES) C_11_H_16_N_2_O_4_ requires 240, found 239 [M–H]^−^.

#### Synthesis of *tert*-Butyl 2-Oxo-1-(4-phenylbutylcarbamoyl)-6,7-dihydro-4*H*-oxazolo[5,4-*c*]pyridine-5-carboxylate (**17a**)

Compound **17a** was prepared according to general procedure D (method A) using **16** (0.052 g, 0.22 mmol) and 4-phenylbutyl isocyanate (0.039 mL, 0.040 g, 0.22 mmol, 1.0 equiv.) in dry MeCN (2 mL). The crude was purified by column chromatography (Cy/EtOAc, 90:10) to afford **17a** as a white solid (0.023 g, 25% over two steps). ^1^H NMR (400 MHz, CDCl_3_) δ 8.01 (bs, 1H), 7.31–7.24 (m, overlapped with CDCl_3_ signal, 2H), 7.22–7.14 (m, 3H), 4.32–4.19 (m, 2H), 3.72–3.59 (m, 2H), 3.34 (q, *J* = 6.7 Hz, 2H), 2.99–2.86 (m, 2H), 2.64 (t, *J* = 7.3 Hz, 2H), 1.75–1.59 (m, 4H), 1.48 (s, 9H). UPLC/MS (*method B*): *R_t_* 1.91 min. MS (ES) C_22_H_29_N_3_O_5_ requires 415, found 416 [M + H]^+^.

#### Synthesis of 2-Oxo-*N*-(4-phenylbutyl)-4,5,6,7-tetrahydrooxazolo[5,4-*c*]pyridine-1-carboxamide Hydrochloride (**17b**)

Compound **17b** was prepared according to general procedure E using **17a** (0.023 g, 0.055 mmol). After evaporation of the solvent, the crude was triturated with Et_2_O to obtain **17b** as a white solid (0.012 g, 60%). ^1^H NMR (400 MHz, DMSO-*d*_6_) δ 9.96 (bs, 2H), 8.05 (bs, 1H), 7.31–7.23 (m, 2H), 7.22–7.12 (m, 3H), 4.08–3.99 (m, 2H), 4.04 (s, 2H), 3.26 (q, *J* = 6.4 Hz, 2H), 3.01–2.93 (m, 2H), 2.59 (t, *J* = 6.9 Hz, 2H), 1.66–1.44 (m, 4H). ^13^C NMR (101 MHz, CDCl_3_) δ 153.08, 149.11, 141.97, 128.54 (2C), 126.02, 40.28 (2C), 38.16, 36.03, 35.56, 29.09, 28.66. UPLC/MS (*method A*): *R_t_* 1.88 min. MS (ES) C_17_H_21_N_3_O_3_ requires 315, found 316 [M + H]^+^. HRMS C_17_H_22_N_3_O_3_ [M + H]^+^: calculated 316.1661, measured: 316.1669, Δppm 2.5.

#### Synthesis of 5-(Cyclohexen-1-yl)-2-nitrophenol (**19a**)

Compound **19a** was prepared according to general procedure A using 5-bromo-2-nitrophenol (0.218 g, 1.0 mmol), **18a** (0.229 g, 1.10 mmol), Pd(PPh_3_)_4_ (0.058 g, 0.05 mmol), and 2 M Na_2_CO_3_ (1.30 mL, 2.50 mmol) in degassed 1,4-dioxane (20 mL). The crude was purified by column chromatography (Cy) to afford **19a** as colorless oil (0.200 g, 90%). ^1^H NMR (400 MHz, CDCl_3_) δ 10.66 (s, 1H), 8.01 (d, *J* = 9.0 Hz, 1H), 7.09 (d, *J* = 1.9 Hz, 1H), 7.03 (dd, *J* = 9.0, 2.0 Hz, 1H), 6.39 (tt, *J* = 4.0, 1.6 Hz, 1H), 2.43–2.34 (m, 2H), 2.31–2.22 (m, 2H), 1.85–1.74 (m, 2H), 1.73–1.62 (m, 2H). UPLC/MS (*method A*): *R_t_* 1.35 min. MS (ES) C_12_H_13_NO_3_ requires 219, found 220 [M + H]^+^.

#### Synthesis of 5-(3,6-Dihydro-2*H*-pyran-4-yl)-2-nitrophenol (**19b**)

Compound **19b** was prepared according to general procedure A using 5-bromo-2-nitrophenol (0.218 g, 1.00 mmol), **18b** (0.231 g, 1.10 mmol), Pd(PPh_3_)_4_ (0.058 g, 0.05 mmol), and 2 M Na_2_CO_3_ (1.30 mL, 2.50 mmol) in degassed 1,4-dioxane (20 mL). The crude was purified by column chromatography (Cy/EtOAc, 85:15) to afford **19b** as a white powder (0.123 g, 56%). ^1^H NMR (400 MHz, DMSO-*d*_6_) δ 10.89 (s, 1H), 7.95–7.85 (m, 1H), 7.15–7.10 (m, 2H), 6.50–6.45 (m, 1H), 4.30–4.20 (m, 2H), 3.82 (t, *J* = 5.4 Hz, 2H), 2.44–2.35 (m, 2H). UPLC/MS (*method A*): *R_t_* 0.51 min. MS (ES) C_11_H_11_NO_4_ requires 221, found 220 [M–H]^−^.

#### Synthesis of *tert*-Butyl 4-(3-Hydroxy-4-nitrophenyl)-3,6-dihydro-2*H*-pyridine-1-carboxylate (**19c**)

Compound **19c** was prepared according to general procedure A using 5-bromo-2-nitrophenol (1.60 g, 7.35 mmol), **18c** (2.50 g, 8.09 mmol), Pd(PPh_3_)_4_ (0.424 g, 0.36 mmol), and 2 M Na_2_CO_3_ (9.2 mL, 18.38 mmol) in degassed 1,4-dioxane (15 mL). The crude was purified by column chromatography (Cy/EtOAc, 80:20) to afford **19c** as a white powder (1.88 g, 80%). ^1^H NMR (400 MHz, DMSO-*d*_6_) δ 10.89 (s, 1H), 7.91 (d, *J* = 8.7 Hz, 1H), 7.19–7.02 (m, 2H), 6.44–6.27 (m, 1H), 4.09–3.97 (m, 2H), 3.54 (t, *J* = 5.7 Hz, 2H), 2.48–2.41 (m, 2H), 1.43 (s, 9H). UPLC/MS (*method A*): *R_t_* 2.54 min. MS (ES) C_16_H_20_N_2_O_5_ requires 320, found 319 [M–H]^−^.

#### Synthesis of *tert*-Butyl 4-(2-Fluoro-5-hydroxy-4-nitrophenyl)-3,6-dihydro-2*H*-pyridine-1-carboxylate (**19d**)

Compound **19d** was prepared according to general procedure A using 5-bromo-4-fluoro-2-nitrophenol (0.280 g, 1.18 mmol), **18c** (0.402 g, 1.3 mmol), Pd(PPh_3_)_4_ (0.07 g, 0.06 mmol), and 2 M Na_2_CO_3_ (1.48 mL, 2.95 mmol) in degassed 1,4-dioxane (12 mL). The crude was purified by column chromatography (Cy/EtOAc, 80:20) to afford **19d** as a yellow solid (0.267 g, 67%). UPLC/MS (*method A*): *R_t_* 2.45 min. MS (ES) C_16_H_19_FN_2_O_5_ requires 338, found 339 [M + H]^+^.

#### Synthesis of 2-Amino-5-cyclohexylphenol (**20a**)

Compound **20a** was prepared according to general procedure B (method B) using **19a** (0.200 g, 0.91 mmol). ^1^H NMR (400 MHz, CDCl_3_) δ 6.74–6.68 (m, 1H), 6.67–6.60 (m, 2H), 2.43–2.30 (m, 1H), 1.93–1.76 (m, 4H), 1.76–1.67 (m, 1H), 1.43–1.29 (m, 4H), 1.29–1.15 (m, 1H). UPLC/MS (*method A*): *R_t_* 0.98 min. MS (ES) C_12_H_17_NO requires 191, found 192 [M + H]^+^.

#### Synthesis of 2-Amino-5-tetrahydropyran-4-ylphenol (**20b**)

Compound **20b** was prepared according to general procedure B (method B) using **19b** (0.123 g, 0.56 mmol). UPLC/MS (*method A*): *R_t_* 0.60 min. MS (ES) C_11_H_15_NO_2_ requires 193, found 194 [M + H]^+^.

#### Synthesis of *tert*-Butyl 4-(4-Amino-3-hydroxyphenyl)piperidine-1-carboxylate (**20c**)

Compound **20c** was prepared according to general procedure B (method B) using **19c** (0.239 g, 0.75 mmol). UPLC/MS (*method A*): *R_t_* 0.98 min. MS (ES) C_16_H_24_N_2_O_3_ requires 292, found 293 [M + H]^+^.

#### Synthesis of *tert*-Butyl 4-(4-Amino-2-fluoro-5-hydroxyphenyl)piperidine-1-carboxylate (**20d**)

Compound **20d** was prepared according to general procedure B (method B) using **19d** (0.265 g, 0.78 mmol). UPLC/MS (*method D*): *R_t_* 1.51 min. MS (ES) C_16_H_23_FN_2_O_3_ requires 310, found 311 [M + H]^+^.

#### Synthesis of 6-Cyclohexyl-3*H*-1,3-benzoxazol-2-one (**21a**)

Compound **21a** was prepared according to general procedure C using **20a** (0.174 g, 0.91 mmol) and CDI (0.295 g, 1.82 mmol) in dry MeCN (9 mL). The crude was used in the next step without further purification. ^1^H NMR (400 MHz, CDCl_3_) δ 8.07 (bs, 1H), 7.09–7.06 (m, 1H), 7.04–6.89 (m, 2H), 2.61–2.44 (m, 1H), 1.96–1.80 (m, 4H), 1.81–1.62 (m, 1H), 1.47–1.31 (m, 4H), 1.31–1.16 (m, 1H). UPLC/MS (*method A*): *R_t_* 2.38 min. MS (ES) C_13_H_15_NO_2_ requires 217, found 218 [M + H]^+^.

#### Synthesis of 6-Tetrahydropyran-4-yl-3*H*-1,3-benzoxazol-2-one (**21b**)

Compound **21b** was prepared according to general procedure C using **20b** (0.108 g, 0.56 mmol) and CDI (0.136 g, 0.84 mmol) in dry MeCN (6 mL). The crude was purified by column chromatography (Cy/EtOAc, 70:30) to afford **21b** as a white powder (0.089 g, 72% over two steps). ^1^H NMR (400 MHz, DMSO-*d*_6_) δ 11.48 (s, 1H), 7.23–7.18 (m, 1H), 7.05–6.95 (m, 2H), 4.00–3.85 (m, 2H), 3.45–3.35 (m, 2H), 2.80–2.70 (m, 1H), 1.70–1.60 (m, 4H). UPLC/MS (*method A*): *R_t_* 1.59 min. MS (ES) C_12_H_13_NO_3_ requires 219, found 220 [M + H]^+^.

#### Synthesis of *tert*-Butyl 4-(2-Oxo-3*H*-1,3-benzoxazol-6-yl)piperidine-1-carboxylate (**21c**)

Compound **21c** was prepared according to general procedure D using **20c** (0.219 g, 0.75 mmol) and CDI (0.183 g, 1.13 mmol) in dry MeCN (8 mL). The crude was purified by column chromatography (Cy/EtOAc, 45:55) to afford **21c** as a white powder (0.195 g, 82% over two steps). ^1^H NMR (400 MHz, DMSO-*d*_6_) δ 11.49 (bs, 1H), 7.24–7.16 (m, 1H), 7.06–6.96 (m, 2H), 4.18–3.94 (m, 2H), 2.94–2.73 (m, 2H), 2.68 (tt, *J* = 11.9, 3.4 Hz, 1H), 1.78–1.70 (m, 2H), 1.55–1.43 (m, 2H), 1.42 (s, 9H). UPLC/MS (*method A*): *R_t_* 2.16 min. MS (ES) C_17_H_22_N_2_O_4_ requires 318, found 317 [M–H]^−^.

#### Synthesis of *tert*-Butyl 4-(5-Fluoro-2-oxo-3*H*-1,3-benzoxazol-6-yl)piperidine-1-carboxylate (**21d**)

Compound **21d** was prepared according to general procedure C using **20d** (0.242 g, 0.78 mmol) and CDI (0.19 g, 1.17 mmol) in dry MeCN (8 mL). The crude was purified by column chromatography (Cy/EtOAc, 70:30) to afford **21d** as a white solid (0.157 g, 60% over two steps). ^1^H NMR (400 MHz, DMSO-*d*_6_) δ 11.68 (bs, 1H), 7.29 (d, *J* = 6.0 Hz, 1H), 6.95 (d, *J* = 9.7 Hz, 1H), 4.18–3.97 (m, 2H), 2.95 (ddd, *J* = 12.0, 8.7, 3.4 Hz, 1H), 2.90–2.67 (m, 2H), 1.74–1.63 (m, 2H), 1.54 (qd, *J* = 12.5, 4.1 Hz, 2H), 1.41 (s, 9H). UPLC/MS (*method D*): *R_t_* 2.21 min. MS (ES) C_17_H_21_FN_2_O_4_ requires 336, found 337 [M + H]^+^.

#### Synthesis of 6-(4-Piperidyl)-3*H*-1,3-benzoxazol-2-one; Hydrochloric Salt (**21e**)

Compound **21e** was prepared according to general procedure E using **21c** (0.193 g, 0.61 mmol). The crude was used in the next step without further purification. UPLC/MS (*method A*): *R_t_* 0.91 min. MS (ES) C_12_H_14_N_2_O_2_ requires 218, found 219 [M + H]^+^.

#### Synthesis of 5-Fluoro-6-(4-piperidyl)-3*H*-1,3-benzoxazol-2-one; Hydrochloric Salt (**21f**)

Compound **21f** was prepared according to general procedure E using **21d** (0.157 g, 0.47 mmol). The crude used in the next step without further purification. UPLC/MS (*method D*): *R_t_* 0.37 min. MS (ES) C_12_H_13_FN_2_O_2_ requires 236, found 237 [M + H]^+^.

#### Synthesis of 6-(1-Methyl-4-piperidyl)-3*H*-1,3-benzoxazol-2-one (**21g**)

Compound **21g** was prepared according to general procedure F using **21e** (0.155 g, 0.61 mmol), 37% aqueous solution of formaldehyde (0.03 mL, 1.22 mmol), NaBH(OAc)_3_ (0.386 g, 1.83 mmol), and AcOH (0.07 mL, 0.073 g, 1.22 mmol) in dry MeCN (6 mL). The crude was used in the next step without further purification. ^1^H NMR (400 MHz, DMSO-*d*_6_) δ 7.19–7.15 (m, 1H), 7.03–6.96 (m, 2H), 2.90–2.80 (m, 2H), 2.53–2.40 (m, overlapped with DMSO signal, 1H), 2.18 (s, 3H), 1.95 (td, *J* = 11.5, 2.7 Hz, 2H), 1.76–1.57 (m, 4H). UPLC/MS (*method A*): *R_t_* 0.91 min. MS (ES) C_13_H_16_N_2_O_2_ requires 232, found 233 [M + H]^+^.

#### Synthesis of 6-(1-Ethyl-4-piperidyl)-3*H*-1,3-benzoxazol-2-one (**21h**)

Compound **21h** was prepared according to general procedure F using **21e** (0.254 g, 1.00 mmol), acetaldehyde (0.21 mL, 1.05 mmol, 5 M in THF), NaBH(OAc)_3_ (0.318 g, 1.6 mmol), and AcOH (0.150 g, 2.5 mmol) in dry THF (10 mL). The crude was purified by SCX to afford **21h** as a yellow powder (0.245 g, quant.). ^1^H NMR (400 MHz, DMSO-*d*_6_) δ 6.82 (s, 1H), 6.80 (d, *J* = 6.0 Hz, 1H), 6.59 (dd, *J* = 8.5, 2.3 Hz, 1H), 3.04–2.97 (m, 4H), 2.52–2.43 (m, overlapped with DMSO signal, 5H), 2.35 (q, *J* = 7.2 Hz, 2H), 1.02 (t, *J* = 7.2 Hz, 3H). UPLC/MS (*method A*): *R_t_* 0.98 min. MS (ES) C_14_H_18_N_2_O_2_ requires 246, found 247[M + H]^+^.

#### Synthesis of 6-(1-Isopropyl-4-piperidyl)-3*H*-1,3-benzoxazol-2-one (**21i**)

Compound **21i** was prepared according to general procedure F using **21e** (0.254 g, 1.00 mmol), NaBH(OAc)_3_ (0.318 g, 1.6 mmol), and AcOH (0.29 mL, 0.30 g, 5.0 mmol) in acetone (10 mL). The residue was purified by column chromatography (DCM/MeOH, 80:20) to afford **21i** as a pink powder (0.104 g, 40%). UPLC/MS (*method A*): *R_t_* 0.99 min. MS (ES) C_15_H_20_N_2_O_2_ requires 261, found 262 [M + H]^+^.

#### Synthesis of 6-(1-Isobutyl-4-piperidyl)-3*H*-1,3-benzoxazol-2-one (**21j**)

Compound **21j** was prepared according to general procedure F using **21e** (0.254 g, 1.00 mmol), NaBH(OAc)_3_ (0.318 g, 1.5 mmol), AcOH (0.29 mL, 0.30 g, 5.0 mmol), and isobutyraldehyde (0.36 g, 5.0 mmol) in dry MeCN (10 mL). The crude was purified by SCX to afford **21j** as a white solid (0.236 g, 86%). ^1^H NMR (400 MHz, DMSO-*d*_6_) δ 7.16–7.13 (m, 1H), 7.02–6.93 (m, 2H), 2.95–2.85 (m, 2H), 2.53–2.43 (m, overlapped with DMSO signal, 1H), 2.05 (d, *J* = 7.4 Hz, 2H), 1.93 (td, *J* = 11.6, 2.5 Hz, 2H), 1.84–1.69 (m, 3H), 1.69–1.56 (m, 2H), 0.86 (d, *J* = 6.5 Hz, 6H). UPLC/MS (*method A*): *R_t_* 1.23 min. MS (ES) C_16_H_22_N_2_O_2_ requires 274, found 275 [M + H]^+^.

#### Synthesis of 5-Fluoro-6-(1-methyl-4-piperidyl)-3*H*-1,3-benzoxazol-2-one (**21k**)

Compound **21k** was prepared according to general procedure F using **21f** (0.123 g, 0.47 mmol) 37% aqueous solution of formaldehyde (0.03 mL, 0.94 mmol), NaBH(OAc)_3_ (0.386 g, 1.83 mmol), and AcOH (0.054 mL, 0.056 g, 0.94 mmol) in dry MeCN (5 mL). The crude was used in the next step without further purification. ^1^H NMR (400 MHz, DMSO-*d*_6_) δ 7.24 (d, *J* = 6.03 Hz, 1H), 6.94 (d, *J* = 9.7 Hz, 1H), 2.93–2.84 (m, 2H), 2.73 (ddd, *J* = 15.5, 10.1, 4.4 Hz, 1H), 2.22 (s, 3H), 2.02 (td, *J* = 11.3, 3.1 Hz, 2H), 1.79–1.60 (m, 4H). UPLC/MS (*method A*): *R_t_* 0.99 min. MS (ES) C_13_H_15_FN_2_O_2_ requires 250, found 251 [M + H]^+^.

#### Synthesis of 6-Cyclohexyl-2-oxo-*N*-(4-phenylbutyl)-1,3-benzoxazole-3-carboxamide (**22a**)

Compound **22a** was prepared following general procedure D (method A) using **21a** (0.169 g, 0.78 mmol) and 4-phenylbutyl isocyanate (0.15 mL, 0.86 mmol) in dry pyridine (8 mL). The crude was purified by column chromatography (Cy/EtOAc, 80:20) to afford **22a** as a white solid (0.300 g, 98%). ^1^H NMR (400 MHz, CDCl_3_) δ 8.04 (t, *J* = 5.4 Hz, 1H), 7.93 (d, *J* = 8.2 Hz, 1H), 7.32–7.24 (m, overlapped with CDCl_3_ signal, 2H), 7.22–7.14 (m, 3H), 7.13–7.06 (m, 2H), 3.44 (q, *J* = 6.8 Hz, 2H), 2.67 (t, *J* = 7.2 Hz, 2H), 2.61–2.48 (m, 1H), 1.93–1.80 (m, 4H), 1.80–1.59 (m, 5H), 1.41 (q, *J* = 10.9, 9.8 Hz, 4H), 1.32–1.19 (m, 1H). ^13^C NMR (101 MHz, CDCl_3_) δ 153.60, 150.06, 145.57, 142.06, 128.56, 128.52, 126.01, 125.90, 123.62, 115.31, 108.28, 44.64, 40.21, 35.60, 34.78, 29.22, 28.71, 26.91, 26.16. UPLC/MS (*method A*): *R_t_* 2.45 min. MS (ES) C_24_H_28_N_2_O_3_ requires 392, found 393 [M + H]^+^. HRMS C_24_H_29_N_2_O_3_ [M + H]^+^: calculated 393.2178, measured: 393.218, Δppm 0.5.

#### Synthesis of 2-Oxo-*N*-(4-phenylbutyl)-6-tetrahydropyran-4-yl-1,3-benzoxazole-3-carboxamide (**22b**)

Compound **22b** was prepared according to general procedure D (method A) using **21b** (0.080 g, 0.37 mmol) and 4-phenylbutyl isocyanate (0.07 mL, 0.072 g, 0.41 mmol) in dry MeCN (2 mL). The crude was purified by column chromatography (Cy/EtOAc, 90:10) to afford **22b** as a white solid (0.075 g, 51%). ^1^H NMR (400 MHz, CDCl_3_) δ 8.03 (t, *J* = 5.4 Hz, 1H), 7.97 (d, *J* = 8.2 Hz, 1H), 7.35–7.25 (m, overlapped with H_2_O signal, 2H), 7.25–7.17 (m, 3H), 7.17–7.10 (m, 2H), 4.20–4.05 (m, 2H), 3.61–3.51 (m, 2H), 3.44 (q, *J* = 6.7 Hz, 2H), 2.88–2.76 (m, 1H), 2.67 (t, *J* = 7.2 Hz, 2H), 1.88–1.68 (m, 8H). ^13^C NMR (101 MHz, CDCl_3_) δ 153.45, 149.96, 143.24, 142.14, 142.03, 128.53, 128.50, 126.01, 123.52, 115.56, 108.24, 68.33, 41.61, 40.23, 35.58, 34.20, 29.19, 28.68. UPLC/MS (*method A*): *R_t_* 2.83 min. MS (ES) C_23_H_26_N_2_O_4_ requires 394, found 218 [M-H-CONH(CH_2_)_4_Ph]^−^. HRMS C_23_H_27_N_2_O_4_ [M + H]^+^: calculated 395.1955, measured: 395.1971, Δppm −4.0.

#### Synthesis of *tert*-Butyl 4-[2-Oxo-3-(4-phenylbutylcarbamoyl)-1,3-benzoxazol-6-yl]piperidine-1-carboxylate (**22c**)

Compound **22c** was prepared according to general procedure D (method A) using **21c** (0.087 g, 0.27 mmol), DMAP (0.037 g, 0.30 mmol), and 4-phenylbutyl isocyanate (0.05 mL, 0.053, 0.30 mmol) in dry MeCN (3 mL). The crude was purified by column chromatography (DCM/MeOH, 90:10) to afford **22c** as a white solid (0.112 g, 84%). ^1^H NMR (400 MHz, CDCl_3_) δ 8.02 (t, *J* = 5.6 Hz, 1H), 7.96 (d, *J* = 8.2 Hz, 1H), 7.31–7.24 (m, overlapped signals with CDCl_3_, 2H), 7.24–7.14 (m, 3H), 7.14–7.05 (m, 2H), 4.39–4.17 (m, 2H), 3.44 (q, *J* = 6.7 Hz, 2H), 2.80 (t, *J* = 12.3 Hz, 2H), 2.74–2.62 (m, 3H), 1.89–1.78 (m, 2H), 1.78–1.54 (m, overlapped with H_2_O signal, 6H), 1.48 (s, 9H). UPLC/MS (*method B*): *R_t_* 2.42 min. MS (ES) C_28_H_35_N_3_O_5_ requires 493, found 494 [M + H]^+^.

#### Synthesis of 2-Oxo-*N*-(4-phenylbutyl)-6-(4-piperidyl)-1,3-benzoxazole-3-carboxamide Hydrochloride (**22d**)

Compound **22d** was prepared according to general procedure E using **22c** (0.112 g, 0.23 mmol). The crude was triturated with Et_2_O to afford **22d** as a white solid (0.085 g, 86%). ^1^H NMR (400 MHz, DMSO-*d*_6_) δ 9.00 (bs, 2H), 8.11 (t, *J* = 5.8 Hz, 1H), 7.83 (d, *J* = 8.3 Hz, 1H), 7.32–7.23 (m, 3H), 7.23–7.08 (m, 4H), 3.40–3.28 (m, overlapped with H_2_O signal, 3H), 3.05–2.82 (m, 3H), 2.61 (t, *J* = 7.2 Hz, 2H), 2.02–1.77 (m, 4H), 1.68–1.49 (m, 4H). ^13^C NMR (101 MHz, DMSO-*d*_6_) δ 152.25, 149.26, 142.00, 141.70, 141.21, 128.27, 128.21, 126.64, 125.65, 122.52, 114.48, 108.08, 43.36, 38.67, 34.73, 29.38, 28.59, 28.12. UPLC/MS (*method A*): *R_t_* 2.40 min. MS (ES) C_23_H_27_N_3_O_3_ requires 393, found 394 [M + H]^+^. HRMS C_23_H_28_N_3_O_3_ [M + H]^+^: calculated 394.213, measured: 394.2131, Δppm −0.3.

#### Synthesis of 6-(1-Methyl-4-piperidyl)-2-oxo-*N*-(4-phenylbutyl)-1,3-benzoxazole-3-carboxamide (**22e**)

Compound **22e** was prepared according to general procedure D using **21g** (0.142 g, 0.61 mmol) and 4-phenylbutyl isocyanate (0.11 mL, 0.118 g, 0.67 mmol) in dry MeCN (3 mL). The crude was purified by column chromatography (DCM/MeOH, 70:30) to afford **22e** as a white solid (0.181 g, 73% over three steps). ^1^H NMR (400 MHz, DMSO-*d*_6_) δ 8.10 (t, *J* = 5.8 Hz, 1H), 7.78 (d, *J* = 8.2 Hz, 1H), 7.32 (d, *J* = 1.5 Hz, 1H), 7.30–7.24 (m, 2H), 7.23–7.13 (m, 4H), 3.38–3.25 (m, overlapped with H_2_O signal, 2H), 2.85 (d, *J* = 11.4 Hz, 2H), 2.61 (t, *J* = 7.3 Hz, 2H), 2.54–2.46 (m, overlapped with DMSO signal, 1H), 2.19 (s, 3H), 1.95 (dd, *J* = 11.4 , 2.8 Hz, 2H), 1.77–1.49 (m, 8H). ^13^C NMR (101 MHz, CDCl_3_) δ 154.40, 149.98, 143.59, 142.05, 140.24, 128.55, 128.51, 126.30, 126.01, 123.69, 115.50, 108.32, 56.28, 46.43, 42.04, 40.23, 35.60, 33.65, 29.21, 28.69. UPLC/MS (*method A*): *R_t_* 2.21 min. MS (ES) C_24_H_29_N_3_O_3_ requires 407, found 408 [M + H]^+^. HRMS C_24_H_30_N_3_O_3_ [M + H]^+^: calculated 408.2287, measured: 408.2291, Δppm 1.0.

#### Synthesis of 6-(1-Ethyl-4-piperidyl)-2-oxo-*N*-(4-phenylbutyl)-1,3-benzoxazole-3-carboxamide (**22f**)

Compound **22f** was prepared according to general procedure D (method A) using **21h** (0.100 g, 0.41 mmol) and 4-phenylbutyl isocyanate (0.08 mL, 0.079 g, 0.45 mmol). The crude was purified by column chromatography (DCM/MeOH, 70:30) to afford **22f** as a yellow solid (0.105 g, 61%). ^1^H NMR (400 MHz, CDCl_3_) δ 8.03 (t, *J* = 5.7 Hz, 1H), 7.95 (d, *J* = 8.9 Hz, 1H), 7.32–7.23 (m, overlapped with CDCl_3_ signal, 2H), 7.21–7.15 (m, 3H), 7.15–7.10 (m, 2H), 3.44 (q, *J* = 6.6 Hz, 2H), 3.09 (d, *J* = 12.0 Hz, 2H), 2.67 (t, *J* = 7.2 Hz, 2H), 2.61–2.51 (m, 1H), 2.47 (q, *J* = 7.2 Hz, 2H), 2.04 (td, *J* = 11.7, 2.9 Hz, 2H), 1.92–1.76 (m, 4H), 1.76–1.60 (m, 4H), 1.13 (t, *J* = 7.2 Hz, 3H). ^13^C NMR (101 MHz, CDCl_3_) δ 153.49, 149.97, 143.81, 142.09, 142.04, 128.54 (2C), 128.50, 126.00, 123.69, 115.44, 108.30, 53.89, 52.74, 42.81, 40.21, 35.59, 33.73, 29.20, 28.69, 12.25. UPLC/MS (*method A*): *R_t_* 2.22 min, MS (ES) C_25_H_31_N_3_O_3_ requires 421, found 422 [M + H]+, 245 [M–CONH(CH_2_)_4_Ph)]^−^. HRMS C_25_H_32_N_3_O_3_ [M + H]^+^: calculated 422.2444, measured 422.2449, Δppm 1.2.

#### Synthesis of 6-(1-Isopropyl-4-piperidyl)-2-oxo-*N*-(4-phenylbutyl)-1,3-benzoxazole-3-carboxamide (**22g**)

Compound **22g** was prepared according to general procedure D (method A) using **21i** (0.100 g, 0.38 mmol) and 4-phenylbutyl isocyanate (0.074 g, 0.42 mmol) in dry MeCN (2 mL). The crude was purified by column chromatography (DCM/MeOH, 70:30) to afford **22g** as a white solid (0.113 g, 68%). ^1^H NMR (400 MHz, CDCl_3_) δ 8.03 (t, *J* = 5.7 Hz, 1H), 7.94 (d, *J* = 8.84 Hz, 1H), 7.31–7.24 (m, overlapped with CDCl_3_ signal, 2H), 7.21–7.15 (m, 3H), 7.15–7.10 (m, 2H), 3.44 (q, *J* = 6.59 Hz, 2H), 3.09–2.96 (m, 2H), 2.85–2.72 (m, 1H), 2.67 (t, *J* = 7.2 Hz, 2H), 2.53 (tt, *J* = 12.1, 4.0 Hz, 1H), 2.32–2.20 (m, 2H), 1.92–1.61 (m, 8H), 1.09 (d, *J* = 6.5 Hz, 6H). ^13^C NMR (101 MHz, CDCl_3_) δ 153.50, 149.98, 144.01, 142.08, 128.53 (2C), 128.50, 126.15, 126.00, 123.70, 115.40, 108.32, 54.85, 49.44, 43.11, 40.21, 35.59, 34.12, 29.20, 28.69, 18.55. UPLC/MS (*method A*): *R_t_* 2.25 min, MS (ES) C_26_H_33_N_3_O_3_ requires 435, found 436 [M + H]^+^. HRMS C_26_H_34_N_3_O_3_ [M + H]^+^: calculated 436.2603, measured 436.26, Δppm 0.6.

#### Synthesis of 6-(1-Isobutyl-4-piperidyl)-2-oxo-*N*-(4-phenylbutyl)-1,3-benzoxazole-3-carboxamide (**22h**)

Compound **22h** was prepared according to general procedure D (method A) using **21j** (0.10 g, 0.36 mmol) and 4-phenylbutyl isocyanate (0.07 mL, 0.07 g, 0.4 mmol). The residue was purified by column chromatography (Cy/EtOAc, 75:25) to afford **22h** as a white powder (0.115 g, 72%). ^1^H NMR (400 MHz, CDCl_3_) δ 8.04 (t, *J* = 5.7 Hz, 1H), 7.94 (d, *J* = 8.86 Hz, 1H), 7.32–7.24 (m, overlapped with CDCl_3_ signal, 2H), 7.22–7.15 (m, 3H), 7.15–7.07 (m, 2H), 3.44 (q, *J* = 6.5 Hz, 2H), 3.08–2.92 (m, 2H), 2.67 (t, *J* = 7.2 Hz, 2H), 2.60–2.46 (m, 1H), 2.19–2.07 (m, 2H), 2.07–1.93 (m, 2H), 1.90–1.50 (m, 9H), 0.92 (d, *J* = 6.5 Hz, 6H). ^13^C NMR (101 MHz, CDCl_3_) δ 153.54, 150.01, 142.66, 142.09, 128.55 (2C), 128.52, 126.01, 123.70, 115.40, 108.35, 67.38, 54.75, 42.87, 40.23, 35.60, 33.83, 29.22, 28.71, 25.79, 21.21 (2C). UPLC/MS (*method A*): *R_t_* 2.43 min, MS (ES) C_27_H_35_N_3_O_3_ requires 449, found 450 [M + H]^+^. HRMS C_27_H_36_N_3_O_3_ [M + H]^+^: calculated 450.2756, measured 450.2757, Δppm −0.2.

#### Synthesis of 6-(1-Acetyl-4-piperidyl)-2-oxo-*N*-(4-phenylbutyl)-1,3-benzoxazole-3-carboxamide (**22i**)

To a solution of **22d** (0.184 g, 0.47 mmol, 1.0 equiv.) in dry THF (5 mL) was added Et_3_N (0.10 g, 0.98 mmol, 2.0 equiv.) dropwise at 0 °C followed by the addition of AcCl (0.039 g, 0.49 mmol, 1.05 equiv.). The reaction mixture was stirred at rt for 4 h, then diluted with EtOAc, washed with saturated aqueous NH_4_Cl solution and brine, and dried over NaSO_4_. After evaporation of the solvent, the crude was purified by column chromatography (DCM/MeOH, 90:10) to afford **22i** as a white powder (0.181 g, 89%). ^1^H NMR (400 MHz, CDCl_3_) δ 8.02 (t, *J* = 5.6 Hz, 1H), 7.97 (d, *J* = 8.2 Hz, 1H), 7.31–7.23 (m, overlapped with CDCl_3_ signal, 2H), 7.21–7.14 (m, 3H), 7.12–7.05 (m, 2H), 4.90–4.70 (m, 1H), 4.05–3.84 (m, 1H), 3.43 (q, *J* = 6.6 Hz, 2H), 3.28–3.07 (m, 1H), 2.79 (tt, *J* = 12.1, 3.6 Hz, 1H), 2.67 (t, *J* = 7.2 Hz, 2H), 2.65–2.55 (m,1H), 2.13 (s, 3H), 1.99–1.83 (m, 2H), 1.80–1.49 (m, overlapped with H_2_O signal, 6H). ^13^C NMR (101 MHz, CDCl_3_) δ 169.02, 149.90, 144.12, 142.54, 142.02, 128.53 (2C), 128.45, 126.56, 126.02, 123.53, 115.66, 108.24, 47.01, 42.78, 42.17, 40.24, 35.58, 29.19, 28.69, 21.64. UPLC/MS (*method A*): *R_t_* 2.48 min, MS (ES) C_25_H_29_N_3_O_4_ requires 435, found 436 [M + H]^+^. HRMS C_25_H_30_N_3_O_4_ [M + H]^+^: calculated 436.2244, measured 436.2236, Δppm 1.8.

#### Synthesis of *N*-(2-Benzyloxyethyl)-6-(1-methyl-4-piperidyl)-2-oxo-1,3-benzoxazole-3-carboxamide (**22j**)

Compound **22j** was prepared according to general procedure D (method C) using **21g** (0.080 g, 0.34 mmol) and 2-(benzyloxy)-1-ethanamine (0.056 g, 0.37 mmol) in dry MeCN (3 mL). The crude was purified by column chromatography (DCM/MeOH, 94:6) to afford **22j** as a white solid (0.033 g, 24%). ^1^H NMR (400 MHz, CDCl_3_) δ 8.32 (bs, 1H), 7.93 (d, *J* = 8.8 Hz, 1H), 7.40–7.30 (m, 4H), 7.29–7.23 (m, overlapped with CDCl_3_ signal, 1H), 7.16–7.08 (m, 2H), 4.57 (s, 2H), 3.71–3.60 (m, 4H), 3.06–2.94 (m, 2H), 2.59–2.48 (m, 1H), 2.35 (s, 3H), 2.09 (td, *J* = 11.4, 3.5 Hz, 2H), 1.92–1.74 (m, 4H). ^13^C NMR (101 MHz, CDCl_3_) δ 153.27, 150.07, 143.59, 128.61 (2C), 127.93 (2C), 127.90, 126.26, 123.63, 115.42, 108.33, 73.39, 68.32, 56.29 46.46, 42.05, 40.32, 33.68. UPLC/MS (*method A*): *R_t_* 1.92 min, MS (ES) C_23_H_27_N_3_O_4_ requires 409, found 410 [M + H]^+^. HRMS C_23_H_28_N_3_O_4_ [M + H]^+^: calculated 410.208, measured 410.2087, Δppm 1.7.

#### Synthesis of 6-(1-Methyl-4-piperidyl)-2-oxo-*N*-pentyl-1,3-benzoxazole-3-carboxamide (**22k**)

Compound **22k** was prepared according to general procedure D (method A) using **21g** (0.050 g, 0.22 mmol) and pentyl isocyanate (0.031 mL, 0.027 g, 0.24 mmol) in dry MeCN (1 mL). The crude was purified by column chromatography (DCM/MeOH, 95:5) to afford **22k** as a white solid (0.039 g, 51%). ^1^H NMR (400 MHz, CDCl_3_) δ 8.02 (t, *J* = 5.8 Hz, 1H), 7.95 (d, *J* = 8.2 Hz, 1H), 7.16–7.07 (m, 2H), 3.41 (q, *J* = 7.0 Hz, 2H), 3.08–2.92 (m, 2H), 2.62–2.45 (m, 1H), 2.35 (s, 3H), 2.09 (td, *J* = 11.3, 3.8 Hz, 2H), 1.91–1.74 (m, 4H), 1.69–1.57 (m, 2H), 1.44–1.29 (m, 4H), 0.98–0.85 (m, 3H). ^13^C NMR (101 MHz, CDCl_3_) δ 153.50, 149.96, 143.55, 126.32, 123.68, 115.50, 108.30, 56.28, 46.44, 42.03, 40.40, 33.66, 29.27, 29.11, 22.45, 14.09. UPLC/MS (*method A*): *R_t_* 2.00 min, MS (ES) C_19_H_27_N_3_O_3_ requires 345, found 346 [M + H]^+^. HRMS C_19_H_28_N_3_O_3_ [M + H]^+^: calculated 346.2131, measured 346.2116, Δppm −4.3.

#### Synthesis of *N*-(2-Ethoxyethyl)-6-(1-methyl-4-piperidyl)-2-oxo-1,3-benzoxazole-3-carboxamide (**22l**)

Compound **22l** was prepared according to general procedure D (method C) using **21g** (0.050 g, 0.22 mmol) and 2-ethoxyethylamine (0.021 g, 0.24 mmol) in dry MeCN (2 mL). The crude was purified by column chromatography (DCM/MeOH, 92:8) to afford **22l** as a white solid (0.030 g, 39%). ^1^H NMR (600 MHz, CDCl_3_) δ 8.29 (bs, 1H), 7.94 (d, *J* = 9.0 Hz, 1H), 7.14–7.08 (m, 2H), 3.63–3.59 (m, 4H), 3.54 (q, *J* = 7.0 Hz, 2H), 3.04–2.94 (m, 2H), 2.59–2.47 (m, 1H), 2.33 (s, 3H), 2.07 (td, *J* = 11.5, 3.3 Hz, 2H), 1.90–1.72 (m, 4H), 1.23 (t, *J* = 7.0 Hz, 3H). ^13^C NMR (151 MHz, CDCl_3_) δ 153.26, 150.06, 143.10, 142.13, 126.36, 123.63, 115.46, 108.35, 68.64, 66.77, 56.11, 46.13, 41.78, 40.33, 33.24, 15.23. UPLC/MS (*method A*): *R_t_* 1.57 min, MS (ES) C_18_H_25_N_3_O_4_ requires 347, found 348 [M + H]^+^. HRMS C_18_H_26_N_3_O_4_ [M + H]^+^: calculated 348.1923, measured 348.1921, Δppm 0.6.

#### Synthesis of *N*-Isobutyl-6-(1-methyl-4-piperidyl)-2-oxo-1,3-benzoxazole-3-carboxamide (**22m**)

Compound **22m** was prepared according to general procedure D (method B) using **21g** (0.404 g, 1.74 mmol) and isobutylamine (0.382 g, 5.22 mmol) in dry DCM (20 mL). The crude was purified by column chromatography (DCM/MeOH, 92:8) to afford **22m** as a white solid (0.259 g, 45%). ^1^H NMR (400 MHz, CDCl_3_) δ 8.08 (t, *J* = 5.9 Hz, 1H), 7.94 (d, *J* = 8.9 Hz, 1H), 7.16–7.05 (m, 2H), 3.24 (t, *J* = 6.4 Hz, 2H), 3.05–2.93 (m, 2H), 2.59–2.45 (m, 1H), 2.33 (s, 3H), 2.07 (td, *J* = 11.4, 3.4 Hz, 2H), 1.97–1.86 (m, 1H), 1.86–1.72 (m, 4H), 0.96 (d, *J* = 6.7 Hz, 6H). ^13^C NMR (101 MHz, CDCl_3_) δ 153.51, 150.06, 143.58, 142.08, 126.29, 123.65, 115.47, 108.27, 56.27, 47.66, 46.45, 42.03, 33.68, 28.58, 20.14. UPLC/MS (*method A*): *R_t_* 1.82 min, MS (ES) C_18_H_25_N_3_O_3_ requires 331, found 332 [M + H]^+^. HRMS C_18_H_26_N_3_O_3_ [M + H]^+^: calculated 332.1974, measured 332.1969, Δppm −1.5.

#### Synthesis of 6-(1-Methyl-4-piperidyl)-2-oxo-*N*-*sec*-butyl-1,3-benzoxazole-3-carboxamide (**22n**)

Compound **22n** was prepared according to general procedure D (method C) using **21g** (0.08 g, 0.34 mmol) and *sec*-butylamine (0.027 g, 0.37 mmol) in dry MeCN (1 mL). The crude was purified by column chromatography (DCM/MeOH, 95:5) to afford **22n** as a white solid (0.029 g, 26%). ^1^H NMR (400 MHz, CDCl_3_) δ 7.95 (d, *J* = 8.0 Hz, 1H), 7.87 (d, *J* = 7.8 Hz, 1H), 7.14–7.08 (m, 2H), 4.07–3.83 (m, 1H), 3.07–2.94 (m, 2H), 2.60–2.46 (m, 1H), 2.35 (s, 3H), 2.09 (td, *J* = 11.4, 3.3 Hz, 2H), 1.89–1.75 (m, 4H), 1.61 (p, *J* = 7.3, 6.9 Hz, 2H), 1.26 (d, *J* = 6.6 Hz, 3H), 0.97 (t, *J* = 7.4 Hz, 3H). ^13^C NMR (101 MHz, CDCl_3_) δ 159.92, 154.18, 149.39, 143.50, 126.36, 123.65, 115.51, 108.27, 56.28, 48.18, 46.45, 42.02, 33.67, 29.63, 20.49, 10.41. UPLC/MS (*method A*): *R_t_* 1.83 min. MS (ES) C_18_H_25_N_3_O_3_ requires 331, found 332 [M + H]^+^. HRMS C_18_H_26_N_3_O_3_ [M + H]^+^: calculated 332.1974, measured: 332.1967, Δppm −2.1.

#### Synthesis of *N*-[4-(4-Fluorophenyl)butyl]-6-(1-methyl-4-piperidyl)-2-oxo-1,3-benzoxazole-3-carboxamide (**22o**)

Compound **22o** was prepared according to general procedure D (method C) using **21g** (0.085 g, 0.51 mmol) and 4-fluorobenzenebutanamine (0.085 g, 0.51 mmol) in dry MeCN (2 mL). The crude was purified by column chromatography (DCM/MeOH, 87:13) to afford **22o** as a white solid (0.042 g, 29%). ^1^H NMR (600 MHz, CDCl_3_) δ 8.03 (t, *J* = 5.5 Hz, 1H), 7.94 (d, *J* = 8.2 Hz, 1H), 7.19–7.06 (m, 4H), 7.01–6.89 (m, 2H), 3.43 (q, *J* = 6.5 Hz, 2H), 3.06–2.95 (m, 2H), 2.63 (t, *J* = 7.0 Hz, 2H), 2.59–2.47 (m, 1H), 2.34 (s, 3H), 2.08 (td, *J* = 11.4, 3.2 Hz, 2H), 1.90–1.76 (m, 4H), 1.74–1.56 (m, 4H). ^13^C NMR (151 MHz, CDCl_3_) δ 161.41 (d, *J*_C–F_ = 243.5 Hz), 153.45, 149.96, 143.29, 142.08, 137.61 (d, *J*_C–F_ = 3.2 Hz), 129.82 (d, *J*_C–F_ = 7.7 Hz), 126.30, 123.70, 115.48, 115.22 (d, *J*_C–F_ = 21.0 Hz), 108.33, 56.14, 46.21, 41.84, 40.14, 34.75, 33.36, 29.10, 28.81. UPLC/MS (*method A*): *R_t_* 2.20 min. MS (ES) C_24_H_28_FN_3_O_3_ requires 425, found 426 [M + H]^+^. HRMS C_24_H_29_FN_3_O_3_ [M + H]^+^: calculated 426.2193, measured: 426.2188, Δppm −1.2.

#### Synthesis of 5-Fluoro-6-(1-methyl-4-piperidyl)-2-oxo-*N*-(4-phenylbutyl)-1,3-benzoxazole-3-carboxamide (**22p**)

Compound **22p** was prepared according to general procedure D (method A) using **21k** (0.117 g, 0.47 mmol) and 4-phenylbutyl isocyanate (0.088 mL, 0.091 g, 0.52 mmol) in dry MeCN (5 mL). The crude was purified by column chromatography (DCM/MeOH, 92:8) to afford **22p** as a white solid (0.099 g, 50% over three steps). ^1^H NMR (600 MHz, CDCl_3_) δ 7.98 (t, *J* = 5.3 Hz, 1H), 7.77 (d, *J* = 9.9 Hz, 1H), 7.31–7.23 (m, overlapped with CDCl_3_ signal, 2H), 7.22–7.14 (m, 3H), 7.11 (d, *J* = 5.8 Hz, 1H), 3.43 (q, *J* = 6.6 Hz, 2H), 3.05–2.97 (m, 2H), 2.95–2.84 (m, 1H), 2.66 (t, *J* = 7.4 Hz, 2H), 2.35 (s, 3H), 2.17 (td, *J* = 11.5, 2.9 Hz, 2H), 1.89–1.78 (m, 4H), 1.75–1.58 (m, overlapped with H_2_O signal, 4H). ^13^C NMR (151 MHz, CDCl_3_) δ 157.38 (d, *J*_C–F_ = 241.2 Hz), 153.43, 149.58, 141.98, 138.08 (d, *J*_C–F_ = 2.2 Hz), 129.37 (d, *J* = 17.7 Hz), 128.53, 128.51, 126.42 (d, *J* = 14.5 Hz), 126.02, 108.44 (d, *J*_C–F_ = 5.6 Hz), 103.97 (d, *J*_C–F_ = 33.4 Hz), 56.08, 46.27, 40.26, 35.56, 34.29, 32.05, 29.13, 28.66. UPLC/MS (*method A*): *R_t_* 2.20 min. MS (ES) C_24_H_28_FN_3_O_3_ requires 425, found 426 [M + H]^+^. HRMS C_24_H_29_FN_3_O_3_ [M + H]^+^: calculated 426.2193, measured: 426.2191, Δppm −0.5.

#### Synthesis of (6-Oxo-2,3-dihydro-1*H*-pyridin-4-yl) trifluoromethanesulfonate (**29a**)

To a solution of **28** (0.400 g, 3.54 mmol, 1.0 equiv.) in dry THF (30 mL) were added at 0 °C under stirring Et_3_N (0.716 g, 7.08 mmol, 2.0 equiv.) and *N*,*N*-bis(trifluoromethylsulfonyl)aniline (1.388 g, 4.24 mmol, 1.2 equiv.) dissolved in dry THF (6 mL). The reaction mixture was slowly warmed to rt and stirred for 16 h. The mixture was diluted with EtOAc, washed with a saturated aqueous NH_4_Cl solution, and dried over Na_2_SO_4_. After evaporation of the solvent, the crude was purified by column chromatography (DCM/MeOH, 94:6) to afford **29a** as a white solid (0.640 g, 74%). ^1^H NMR (400 MHz, DMSO-*d*_6_) δ 7.79 (s, 1H), 5.95–5.93 (m, 1H), 3.37 (td, *J* = 7.1, 2.7 Hz, 2H), 2.72 (td, *J* = 7.2, 1.4 Hz, 2H). UPLC/MS (*method A*): *R_t_* 1.47 min, MS (ES) C_6_H_6_F_3_NO_4_S requires 245, found 246 [M + H]^+^.

#### Synthesis of 4-(4,4,5,5-Tetramethyl-1,3,2-dioxaborolan-2-yl)-2,3-dihydro-1*H*-pyridin-6-one (**29b**)

To a stirred solution of compound **29a** (0.100 g, 0.41 mmol, 1.0 equiv.) in degassed dioxane (4 mL) were added ([B_2_(pin)_2_]) (0.124 g, 0.49 mmol, 1.2 equiv.), Pd(dppf)Cl_2_ (0.058 g, 0.08 mmol, 0.2 equiv.), and KOAc (0.080 g, 0.82 mmol, 2.0 equiv.). The reaction mixture was stirred at 70 °C for 90 min, then cooled to rt, and used directly in the next step. UPLC/MS (*method A*): *R_t_* 0.44 min, MS (ES) C_11_H_18_BNO_3_ requires 223, found 141 [M–(CH_3_)_2_CC(CH_3_)_2_]^+^.

#### Synthesis of 4-(3-Benzyloxy-4-nitrophenyl)-2,5-dihydro-1*H*-pyridin-6-ne (**30a**)

Compound **30a** was prepared according to general procedure A using **29b** (0.091 g, 0.41 mmol), 2-benzyloxy-4-bromo-1-nitrobenzene (0.138 g, 0.45 mmol), Pd(PPh_3_)_4_ (0.023 g, 0.02 mmol), and 2 M Na_2_CO_3_ (0.51 mL, 1.025 mmol) in degassed 1,4-dioxane (10 mL). The crude was purified by column chromatography (DCM/MeOH, 90:10) to afford **30a** as a brown powder (0.124 g, 93% over two steps). ^1^H NMR (400 MHz, DMSO-*d*_6_) δ 7.93 (d, *J* = 8.5 Hz, 1H), 7.61 (d, *J* = 1.7 Hz, 1H), 7.60 (bs, 1H), 7.50–7.47 (m, 2H), 7.46–7.40 (m, 2H), 7.39–7.33 (m, 2H), 6.34 (q, *J* = 1.5 Hz, 1H), 5.42 (s, 2H), 3.39 (td, *J* = 7.0, 2.6 Hz, 2H), 2.74 (td, *J* = 7.0, 1.5 Hz, 2H). UPLC/MS (*method A*): *R_t_* 1.80 min, MS (ES) C_18_H_16_N_2_O_4_ requires 324, found 325 [M + H]^+^.

#### Synthesis of 4-(3-Benzyloxy-4-nitrophenyl)-1-methyl-2,3-dihydropyridin-6-one (**30b**)

To a stirred solution of **30a** (0.124 g, 0.38 mmol, 1.0 equiv.) in dry THF (4 mL) was added NaH (0.018 g, 60% in mineral oil, 0.46 mmol, 1.2 equiv.) at 0 °C under stirring. After 30 min, CH_3_I (0.047 mL, 0.76 mmol, 2.0 equiv.) was added, and the mixture was slowly warmed to rt. After 5 h, saturated aqueous NH_4_Cl solution was added, and the mixture extracted with EtOAc. The combined organic phases were washed with brine, dried over MgSO_4_, and concentrated. The crude was purified by column chromatography (DCM/EtOAc, 70:30) to afford **30b** as a brown solid (0.058 g, 45%). ^1^H NMR (400 MHz, DMSO-*d*_6_) δ 7.94 (d, *J* = 8.5 Hz, 1H), 7.63 (d, *J* = 1.8 Hz, 1H), 7.51–7.46 (m, 2H), 7.46–7.39 (m, 2H), 7.40–7.32 (m, 2H), 6.41 (t, *J* = 1.4 Hz, 1H), 5.42 (s, 2H), 3.53 (t, *J* = 7.1 Hz, 2H), 2.92 (s, 3H), 2.83 (td, *J* = 7.2, 1.4 Hz, 2H). UPLC/MS (*method A*): *R_t_* 1.89 min, MS (ES) C_19_H_18_N_2_O_4_ requires 338, found 339 [M + H]^+^.

#### Synthesis of 4-(4-Amino-3-hydroxyphenyl)-1-methyl-piperidin-2-one (**31**)

Compound **31** was prepared according to general procedure B (method B) using **30b** (0.056 g, 0.16 mmol). UPLC/MS (*method A*): *R_t_* 1.05 min, MS (ES) C_12_H_16_N_2_O_2_ requires 220, found 221 [M + H]^+^.

#### Synthesis of 6-(1-Methyl-2-oxo-4-piperidyl)-3*H*-1,3-benzoxazol-2-one (**21l**)

Compound **21l** was prepared according to general procedure C using **31** (0.035 g, 0.16 mmol) and CDI (0.039 g, 0.24 mmol) in dry MeCN (2 mL). The crude was purified by column chromatography (DCM/MeOH, 95:5) to afford **21l** as a white powder (0.028 g, 70% over two steps). ^1^H NMR (400 MHz, DMSO-*d*_6_) δ 7.26–7.23 (m, 1H), 7.08–6.96 (m, 2H), 3.45–3.23 (m, 2H), 3.14–3.03 (m, 1H), 2.85 (s, 3H), 2.49–2.28 (m, 2H), 2.06–1.76 (m, 2H). UPLC/MS (*method A*): *R_t_* 1.17 min, MS (ES) C_13_H_14_N_2_O_3_ requires 246, found 247 [M + H]^+^.

#### Synthesis of 6-(1-Methyl-2-oxo-4-piperidyl)-2-oxo-*N*-(4-phenylbutyl)-1,3-benzoxazole-3-carboxamide (**22q**)

Compound **22q** was prepared according to general procedure D (method A) using **21l** (0.025 g, 0.10 mmol) and 4-phenylbutyl isocyanate (0.019 mL, 0.019 g, 0.11 mmol) in dry MeCN (1 mL). The crude was purified by column chromatography (DCM/EtOAc, 70:30) to afford **22q** as a white solid (0.038 g, 90%). ^1^H NMR (400 MHz, CDCl_3_) δ 8.07–7.95 (m, 2H), 7.33–7.23 (m, overlapped with CDCl_3_ signal, 2H), 7.22–7.13 (m, 3H), 7.13–7.05 (m, 2H), 3.51–3.38 (m, 3H), 3.38–3.28 (m, 1H), 3.21–3.08 (m, 1H), 2.99 (s, 3H), 2.79–2.68 (m, 1H), 2.67 (t, *J* = 7.2 Hz, 2H), 2.46 (dd, *J* = 17.3, 11.1 Hz, 1H), 2.19–2.08 (m, 1H), 2.07–1.91 (m, 1H), 1.80–1.67 (m, 4H). ^13^C NMR (101 MHz, CDCl_3_) δ 168.96, 149.85, 142.26, 142.01, 140.73, 128.53 (2C), 126.87, 126.02, 123.31, 115.86, 108.10, 100.13, 49.07, 40.27, 39.61, 38.89, 35.59, 34.65, 30.45, 29.19, 28.68. UPLC/MS (*method A*): *R_t_* 2.09 min, MS (ES) C_24_H_27_N_3_O_4_ requires 421, found 422 [M + H]^+^. HRMS C_24_H_28_N_3_O_4_ [M + H]^+^: calculated 422.208, measured 422.2074, Δppm −1.4.

#### Synthesis of 6-[1-(2,2-Difluoroethyl)-4-hydroxy-4-piperidyl]-3*H*-1,3-benzoxazol-2-one (**43**)

Compound **43** was prepared according to general procedure I using 6-bromo-3*H*-1,3-benzoxazol-2-one (0.150 g, 0.7 mmol), **42a** (0.171 g, 1.05 mmol), MeMgBr (0.35 mL, 0.125 g, 1.05 mmol, 3 M in Et_2_O), and *n-*BuLi (0.336 mL, 0.84 mmol, 2.5 M in hexanes) in dry THF (7 mL). The crude was purified by column chromatography (DCM/MeOH, 98:4) to afford **43** as a white solid (0.063 g, 30%). ^1^H NMR (400 MHz, DMSO-*d*_6_) δ 7.37 (d, *J* = 1.4 Hz, 1H), 7.26 (dd, *J* = 8.2, 1.7 Hz, 1H), 7.01 (d, *J* = 8.1 Hz, 1H), 6.14 (tt, *J* = 55.9, 4.3 Hz, 1H), 4.88 (bs, 1H), 2.82–2.68 (m, 4H), 2.63 (td, *J* = 11.6, 2.4 Hz, 2H), 1.93 (td, *J* = 12.8, 4.7 Hz, 2H), 1.57 (dd, *J* = 13.8, 2.5 Hz, 2H). UPLC/MS (*method A*): *R_t_* 1.11 min, MS (ES) C_14_H_16_F_2_N_2_O_3_ requires 298, found 299 [M + H]^+^.

#### Synthesis of 6-[1-(2,2-Difluoroethyl)-3,6-dihydro-2*H*-pyridin-4-yl]-3*H*-1,3-benzoxazol-2-one (**44**)

Compound **44** was prepared according to general procedure L using **43** (0.033 g, 0.11 mmol). The crude was purified by SCX to afford **44** as a pale brown solid (0.031 g, quant.). ^1^H NMR (400 MHz, DMSO-*d*_6_) δ 11.57 (bs, 1H), 7.38 (d, *J* = 1.6 Hz, 1H), 7.22 (dd, *J* = 8.2, 1.7 Hz, 1H), 7.03 (d, *J* = 8.1 Hz, 1H), 6.18 (tt, *J* = 55.8, 4.3 Hz, 1H), 6.10 (td, *J* = 3.5, 1.7 Hz, 1H), 3.27–3.19 (m, 2H), 2.92–2.74 (m, 4H), 2.49–2.41 (m, 2H). UPLC/MS (*method A*): *R_t_* 1.56 min, MS (ES) C_14_H_14_F_2_N_2_O_2_ requires 280, found 281 [M + H]^+^.

#### Synthesis of 6-[1-(2,2-Difluoroethyl)-4-piperidyl]-3*H*-1,3-benzoxazol-2-one (**21m**)

Compound **21m** was prepared according to general procedure B (method B) using **44** (0.028 g, 0.1 mmol). The crude was used in the next step without further purification. ^1^H NMR (400 MHz, DMSO-*d*_6_) δ 11.46 (bs, 1H), 7.20 (d, *J* = 1.4 Hz, 1H), 7.08–6.95 (m, 2H), 6.14 (tt, *J* = 55.8, 4.4 Hz, 1H), 3.04–2.95 (m, 2H), 2.75 (td, *J* = 15.7, 4.4 Hz, 2H), 2.26 (td, *J* = 11.6, 2.8 Hz, 2H), 1.80–1.56 (m, 4H). UPLC/MS (*method A*): *R_t_* 1.50 min, MS (ES) C_14_H_16_F_2_N_2_O_2_ requires 282, found 283 [M + H]^+^.

#### Synthesis of 6-[1-(2,2-Difluoroethyl)-4-piperidyl]-2-oxo-*N*-(4-phenylbutyl)-1,3-benzoxazole-3-carboxamide (**22r**)

Compound **22r** was prepared according to general procedure D (method A) using **21m** (0.028 g, 0.1 mmol) and 4-phenylbutyl isocyanate (0.019 mL, 0.019 g, 0.11 mmol) in dry MeCN (2.0 mL). The crude was purified by column chromatography (DCM/EtOAc, 90:10) to afford **22r** as a white solid (0.033 g, 73% over two steps). ^1^H NMR (400 MHz, CDCl_3_) δ 8.02 (t, *J* = 5.7 Hz, 1H), 7.95 (d, *J* = 8.3 Hz, 1H), 7.32–7.24 (m, overlapped with CDCl_3_ signal, 2H), 7.22–7.15 (m, 3H), 7.15–7.08 (m, 2H), 5.92 (tt, *J* = 56.0, 4.3 Hz, 1H), 3.44 (q, *J* = 6.6 Hz, 2H), 3.07 (d, *J* = 11.6 Hz, 2H), 2.79 (td, *J* = 15.0, 4.3 Hz, 2H), 2.67 (t, *J* = 7.2 Hz, 2H), 2.61–2.49 (m, 1H), 2.34 (td, *J* = 11.3, 3.5 Hz, 2H), 1.91–1.78 (m, 4H), 1.78–1.66 (m, 4H). ^13^C NMR (101 MHz, CDCl_3_) δ 153.47, 150.61, 149.97, 143.39, 142.04, 128.54 (2C), 128.49, 126.02, 123.62, 115.52 (2C), 108.28, 60.57 (t, *J*_C–F_ = 24.9 Hz), 55.07, 42.14, 40.24, 35.59, 33.63, 29.20, 28.69. UPLC/MS (*method A*): *R_t_* 1.82 min, MS (ES) C_25_H_29_F_2_N_3_O_3_ requires 457, found 458 [M + H]^+^. HRMS C_25_H_30_F_2_N_3_O_3_ [M + H]^+^: calculated 458.2255, measured 458.2258, Δppm 0.7.

#### Synthesis of 2-Nitro-5-(1-piperidyl)phenol (**49a**)

Compound **49a** was prepared according to general procedure G using 5-fluoro-2-nitrophenol (0.150 g, 0.95 mmol), **48a** (0.265 g, 1.43 mmol), and DIPEA (0.33 mL, 0.25 g, 1.90 mmol) in dry MeCN (8 mL) under MW irradiation. Saturated aqueous NH_4_Cl solution was added, and the aqueous phase was extracted with DCM. The organic layers were collected, dried over Na_2_SO_4_, and concentrated under reduced pressure. The crude was used in the next step without further purification. ^1^H NMR (400 MHz, CDCl_3_) δ 11.31 (s, 1H), 7.93 (d, *J* = 9.7 Hz, 1H), 6.45 (dd, *J* = 9.7, 2.7 Hz, 1H), 6.31 (d, *J* = 2.7 Hz, 1H), 3.47 (d, *J* = 5.8 Hz, 4H), 1.70 (s, 6H). UPLC/MS (*method A*): *R_t_* 2.45 min. MS (ES) C_11_H_14_N_2_O_3_ requires 222, found 223 [M + H]^+^.

#### Synthesis of 5-(1,1-Dioxo-1,4-thiazinan-4-yl)-2-nitrophenol (**49c**)

Compound **49c** was prepared according to general procedure G using 5-fluoro-2-nitrophenol (0.470 g, 3.00 mmol), **48c** (0.811 g, 6.00 mmol), and DIPEA (1.05 mL, 0.775 g, 6.00 mmol) in MeCN (15 mL), heating at reflux for 16 h. The crude was purified by column chromatography (EtOAc) to afford **49c** as a yellow powder (0.33 g, 40%). ^1^H NMR (400 MHz, DMSO-*d*_6_) δ 10.85 (bs, 1H), 7.91 (d, *J* = 9.6 Hz, 1H), 6.73 (dd, *J* = 9.6, 2.8 Hz, 1H), 6.59 (d, *J* = 2.8 Hz, 1H), 4.02–3.94 (m, 4H), 3.20–3.14 (m, 4H). UPLC/MS (*method A*): *R_t_* 1.49 min. MS (ES) C_10_H_12_N_2_O_5_S requires 272, found 273 [M + H]^+^.

#### Synthesis of *tert-*Butyl 4-(3-Hydroxy-4-nitrophenyl)piperazine-1-carboxylate (**49d**)

Compound **49d** was prepared according to general procedure G using 5-fluoro-2-nitrophenol (2.00 g, 12.73 mmol), **48d** (3.56 g, 19.09 mmol), and DIPEA (2.47 g, 19.1 mmol) in MeCN (25 mL), heating at reflux for 16 h. The crude was used in the next step without further purification. ^1^H NMR (400 MHz, DMSO-*d*_6_) δ 10.94 (bs, 1H), 7.88 (d, *J* = 9.7 Hz, 1H), 6.64 (dd, *J* = 9.7, 2.7 Hz, 1H), 6.42 (d, J = 2.7 Hz, 1H), 3.56–3.40 (m, 8H), 1.42 (s, 9H). UPLC/MS (*method A*): *R_t_* 2.36 min, MS (ES) C_15_H_21_N_3_O_5_ requires 323, found 324 [M + H]^+^.

#### Synthesis of 2-Amino-5-(1-piperidyl)phenol (**49e**)

Compound **49e** was prepared according to general procedure B (method A) using **49a** (0.130 g, 0.59 mmol). After evaporation of the solvent, the crude was used in the next step without purification. UPLC/MS (*method A*): *R_t_* 0.94 min. MS (ES) C_11_H_16_N_2_O requires 192, found 193 [M + H]^+^.

#### Synthesis of 2-Amino-5-morpholinophenol (**49f**)

Compound **49f** was prepared according to general procedure B (method A) using the commercially available **49b** (0.224 g, 1.0 mmol). After evaporation of the solvent, the crude was used in the next step without purification. UPLC/MS (*method A*): *R_t_* 1.18 min. MS (ES) C_10_H_14_N_2_O_2_ requires 194, found 195 [M + H]^+^.

#### Synthesis of 2-Amino-5-(1,1-dioxo-1,4-thiazinan-4-yl)phenol (**49g**)

Compound **49g** was prepared according to general procedure B (method A) using **49c** (0.272 g, 1.00 mmol). After evaporation of the solvent, the crude was used in the next step without purification. UPLC/MS (*method A*): *R_t_* 0.52 min. MS (ES) C_10_H_14_N_2_O_3_S requires 242, found 243 [M + H]^+^.

#### Synthesis of *tert*-Butyl 4-(4-Amino-3-hydroxyphenyl)piperazine-1-carboxylate (**49h**)

Compound **49h** was prepared according to general procedure B (method B) using **49d** (4.11 g, 12.73 mmol). After evaporation of the solvent, the crude was used in the next step without purification. UPLC/MS (*method A*): *R_t_* 1.77 min. MS (ES) C_15_H_23_N_3_O_3_ requires 293, found 294 [M + H]^+^.

#### Synthesis of 6-(1-Piperidyl)-3*H*-1,3-benzoxazol-2-one (**50a**)

Compound **50a** was prepared according to general procedure C using **49e** (0.110 g, 0.58 mmol) and CDI (0.141 g, 0.87 mmol) in dry MeCN (6 mL). The pink solid was triturated with Et_2_O to afford **50a** as a pinkish solid (0.165 g, 80% over three steps). ^1^H NMR (400 MHz, DMSO-*d*_6_) δ 11.26 (bs, 1H), 6.93 (d, *J* = 2.2 Hz, 1H), 6.90 (d, *J* = 8.5 Hz, 1H), 6.70 (dd, *J* = 2.3, 8.5 Hz, 1H), 3.08–2.93 (m, 4H), 1.61 (p, *J* = 5.7 Hz, 4H), 1.50 (p, *J* = 5.7 Hz, 2H). UPLC/MS (*method A*): *R_t_* 2.38 min. MS (ES) C_12_H_14_N_2_O_2_ requires 218, found 219 [M + H]^+^.

#### Synthesis of 6-Morpholino-3*H*-1,3-benzoxazol-2-one (**50b**)

Compound **50b** was prepared according to general procedure C using **49f** (0.194 g, 1.00 mmol) and CDI (0.243 g, 1.50 mmol) in dry MeCN (10 mL). The crude was purified by column chromatography (Cy/EtOAc, 70:30) to afford **50b** as a pink powder (0.132 g, 60%, over two steps). ^1^H NMR (400 MHz, DMSO-*d*_6_) δ 11.31 (bs, 1H), 6.98 (d, *J* = 2.3 Hz, 1H), 6.94 (d, *J* = 8.5 Hz, 1H), 6.72 (dd, *J* = 8.6, 2.3 Hz, 1H), 3.81–3.65 (m, 4H), 3.10–2.93 (m, 4H). UPLC/MS (*method A*): *R_t_* 1.32 min. MS (ES) C_11_H_12_N_2_O_3_ requires 220, found 221 [M + H]^+^.

#### Synthesis of 6-(1,1-Dioxo-1,4-thiazinan-4-yl)-3*H*-1,3-benzoxazol-2-one (**50c**)

Compound **50c** was prepared according to general procedure C using **49g** (0.242 g, 1.0 mmol) and CDI (0.162 g, 1.0 mmol) in dry MeCN (10 mL). The crude was purified by column chromatography (Cy/EtOAc 70:30) to afford **50c** as a yellow solid (0.120 g, 45% over two steps). ^1^H NMR (400 MHz, DMSO-*d*_6_) δ 6.94 (d, *J* = 2.4 Hz, 1H), 6.87 (d, *J* = 8.4 Hz, 1H), 6.72 (dd, *J* = 8.4, 2.4 Hz, 1H), 3.70–3.65 (m, 4H), 3.17–3.12 (m, 4H). UPLC/MS (*method A*): *R_t_* 1.15 min. MS (ES) C_11_H_12_N_2_O_4_S requires 268, found 267 [M-H]^−^.

#### Synthesis of *tert*-Butyl 4-(2-Oxo-3*H*-1,3-benzoxazol-6-yl)piperazine-1-carboxylate (**50d**)

Compound **50d** was prepared according to general procedure C using **49h** (3.73 g, 12.73 mmol) and CDI (3.096 g, 19.09 mmol) in dry MeCN (25 mL). The crude was purified by column chromatography (Cy/EtOAc, 80:20) to afford **50d** as a pink powder (3.046 g, 75% over three steps). ^1^H NMR (400 MHz, DMSO-*d*_6_) δ 11.34 (bs, 1H), 7.00 (d, *J* = 2.2 Hz, 1H), 6.94 (d, *J* = 8.5 Hz, 1H), 6.74 (dd, *J* = 8.5, 2.3 Hz, 1H), 3.50–3.40 (m, 4H), 3.05–2.96 (m, 4H), 1.42 (s, 9H). UPLC/MS (*method A*): *R_t_* 2.14 min, MS (ES) C_16_H_21_N_3_O_4_ requires 319, found 320 [M + H]^+^.

#### Synthesis of 6-Piperazin-1-yl-3*H*-1,3-benzoxazol-2-one Hydrochloride (**50e**)

Compound **50e** was prepared according to general procedure E using **50d** (1.50 g, 4.70 mmol). The reaction mixture was concentrated under reduced pressure to afford **50e** as a gray solid (1.198 g, quant.). ^1^H NMR (400 MHz, DMSO-*d*_6_) δ 11.41 (bs, 1H), 8.80 (bs, 2H), 7.05 (d, *J* = 2.2 Hz, 1H), 6.97 (d, *J* = 8.5 Hz, 1H), 6.77 (dd, *J* = 8.5, 2.3 Hz, 1H), 3.31–3.18 (m, 8H). UPLC/MS (*method A*): *R_t_* 0.55 min. MS (ES) C_11_H_13_N_3_O_2_ requires 219, found 220 [M + H]^+^.

#### Synthesis of 6-(4-Methylpiperazin-1-yl)-3*H*-1,3-benzoxazol-2-one (**50f**)

Compound **50f** was prepared according to general procedure F using **50e** (0.388 g, 1.52 mmol), 37% aqueous solution of formaldehyde (0.17 mL, 6.08 mmol), NaBH(OAc)_3_ (0.21 g, 1.0 mmol), and AcOH (0.096 mL, 0.101 g, 1.68 mmol) in dry MeCN (5 mL). The crude was used in the next step without further purification. ^1^H NMR (400 MHz, CDCl_3_) δ 6.97–6.90 (m, 2H), 6.73–6.69 (m, 1H), 3.09–3.02 (m, 4H), 2.47–2.41 (m, 4H) 2.22 (s, 3H). UPLC/MS (*method A*): *R_t_* 0.85 min. MS (ES) C_12_H_15_N_3_O_2_ requires 233, found 234 [M + H]^+^.

#### Synthesis of 6-(4-Ethylpiperazin-1-yl)-3*H*-1,3-benzoxazol-2-one (**50g**)

Compound **50g** was prepared according to general procedure F using **50e** (0.171 g, 0.67 mmol), NaBH(OAc)_3_ (0.21 g, 1.0 mmol), AcOH (0.096 mL, 0.101 g, 1.68 mmol), and acetaldehyde (0.14 mL, 0.70 mmol, 5 M in THF) in dry THF (7 mL). The crude was purified by SCX to afford **50g** as a white solid (0.150 g, 90%). UPLC/MS (*method A*): *R_t_* 0.88 min. MS (ES) C_13_H_17_N_3_O_2_ requires 247, found 248 [M + H]^+^.

#### Synthesis of 6-(4-Isopropylpiperazin-1-yl)-3*H*-1,3-benzoxazol-2-one (**50h**)

Compound **50h** was prepared according to general procedure F using **50e** (0.256 g, 1.00 mmol), NaBH(OAc)_3_ (0.636 g, 3.0 mmol), and AcOH (0.286 mL, 0.300 g, 5.00 mmol) in acetone (10 mL). The crude was purified by SCX to afford **50h** as a white solid (0.158 g, 60%). ^1^H NMR (400 MHz, DMSO-*d_6_*) δ 11.40 (bs, 1H), 7.10–7.03 (m, 1H), 6.97 (d, *J* = 8.5 Hz, 1H), 6.78 (dd, *J* = 8.5, 2.2 Hz, 1H), 3.94–2.79 (m, overlapped with H_2_O signal, 9H), 1.25 (s, 6H). UPLC/MS (*method A*): *R_t_* 0.99 min, MS (ES) C_14_H_19_N_3_O_2_ requires 261, found 262 [M + H]^+^.

#### Synthesis of 6-(4-Isobutylpiperazin-1-yl)-3*H*-1,3-benzoxazol-2-one (**50i**)

Compound **50i** was prepared according to general procedure F using **50e** (0.256 g, 1.0 mmol), isobutyraldehyde (0.361 g, 5.0 mmol), NaBH(OAc)_3_ (0.318 g, 1.50 mmol), and AcOH (0.286 mL, 0.300 g, 5.0 mmol) in dry MeCN (10 mL). The crude was purified by SCX to afford **50i** as a violet solid (0.220 g, 80%). ^1^H NMR (400 MHz, DMSO-*d*_6_) δ 6.88–6.80 (m, 2H), 6.63 (dd, *J* = 8.5, 2.3 Hz, 1H), 3.09–2.83 (m, 4H), 2.47–2.39 (m, 4H), 2.07 (d, *J* = 7.4 Hz, 2H), 1.78 (dt, *J* = 13.6, 6.8 Hz, 1H), 0.87 (d, *J* = 6.6 Hz, 6H). UPLC/MS (*method A*): *R_t_* 1.21 min. MS (ES) C_15_H_21_N_3_O_2_ requires 275, found 276 [M + H]^+^.

#### Synthesis of 2-Oxo-*N*-(4-phenylbutyl)-6-piperidin-1-ium-1-yl-1,3-benzoxazole-3-carboxamide Hydrochloride (**23a**)

Compound **23a** was prepared according to general procedure D (method A) using **50a** (0.055 g, 0.25 mmol) and 4-phenylbutyl isocyanate (0.047 mL, 0.049 g, 0.86 mmol) in a mixture of toluene/DMF (3 mL, 9:1). The crude was purified by column chromatography (Cy) (0.029 g, 30%). The free base of **23a** was dissolved in DCM (0.7 mL, 0.1 M) followed by the addition of HCl (0.55 mL, 0.08 g, 2.14 mmol, 4 M in 1,4-dioxane). After evaporation of the solvent, the residue was triturated with Et_2_O to afford **23a** as a white solid (0.026 g, 86%). ^1^H NMR (400 MHz, DMSO-*d*_6_) δ 8.10 (t, *J* = 5.8 Hz, 1H), 8.06–7.83 (m, 2H), 7.82–7.46 (m, 1H), 7.32–7.24 (m, 2H), 7.23–7.13 (m, 3H), 4.63 (bs, 1H), 3.58–3.40 (m, 4H), 3.34 (q, *J* = 6.4 Hz, 2H), 2.61 (t, *J* = 7.2 Hz, 2H), 2.22–1.79 (m 4H), 1.78–1.51 (m, 6H). ^13^C NMR (151 MHz, CDCl_3_) δ 152.59, 149.20, 141.91, 141.89, 139.51, 129.29, 128.52, 128.50, 126.05, 118.19, 116.86, 105.34, 58.14, 40.39, 35.52, 29.06, 28.63, 23.14, 21.80. UPLC/MS (*method A*): *R_t_* 2.33 min. MS (ES) C_23_H_27_N_3_O_3_ requires 393, found 394 [M + H]^+^. HRMS C_23_H_28_N_3_O_3_ [M + H]^+^: calculated 394.2131, measured: 394.2122, Δppm −2.3.

#### Synthesis of 6-Morpholino-2-oxo-*N*-(4-phenylbutyl)-1,3-benzoxazole-3-carboxamide (**23b**)

Compound **23b** was prepared according to general procedure D (method A) using **50b** (0.130 g, 0.6 mmol) and 4-phenylbutyl isocyanate (0.157 g, 0.9 mmol) in dry MeCN (6 mL). The crude was purified by column chromatography (Cy/EtOAc, 90:10) to afford **23b** as a white powder (0.142 g, 60%). ^1^H NMR (400 MHz, CDCl_3_) δ 7.99 (t, *J* = 5.7 Hz, 1H), 7.91 (d, *J* = 8.6 Hz, 1H), 7.32–7.24 (m, overlapped with CDCl_3_ signal, 2H), 7.20–7.07 (m, 3H), 6.90–6.78 (m, 2H), 3.95–3.82 (m, 4H), 3.44 (q, *J* = 6.6 Hz, 2H), 3.20–3.10 (m, 4H), 2.67 (t, *J* = 7.2 Hz, 2H), 1.78–1.60 (m, 4H). ^13^C NMR (101 MHz, CDCl_3_) δ 153.48, 150.00, 142.91, 142.05, 128.54, 128.51, 126.01, 115.97, 112.77, 98.66, 66.76, 50.27, 40.22, 35.60, 29.22, 28.70. UPLC/MS (*method A*): *R_t_* 2.63 min. MS (ES) C_22_H_25_N_3_O_4_ requires 395, found 396 [M + H]^+^. HRMS C_22_H_26_N_3_O_4_ [M + H]^+^: calculated 396.1923, measured 396.1925, Δppm 0.5.

#### Synthesis of 6-(1,1-Dioxo-1,4-thiazinan-4-yl)-2-oxo-*N*-(4-phenylbutyl)-1,3-benzoxazole-3-carboxamide (**23c**)

Compound **23c** was prepared according to general procedure D (method A) using **50c** (0.100 g, 0.37 mmol) and 4-phenylbutyl isocyanate (0.20 g, 1.12 mmol) in dry MeCN (4 mL). The crude was purified by column chromatography (Cy/EtOAc, 80:20) to afford **23c** as a white powder (0.050 g, 20%). ^1^H NMR (400 MHz, DMSO-*d*_6_) δ 8.06 (t, *J* = 5.8 Hz, 1H), 7.71 (d, *J* = 8.8 Hz, 1H), 7.32–7.24 (m, 2H), 7.24–7.12 (m, 4H), 6.94 (dd, *J* = 8.9, 2.5 Hz, 1H), 3.87–3.67 (m, 4H), 3.42–3.26 (m, 2H), 3.21–3.06 (m, 4H), 2.61 (t, *J* = 7.2 Hz, 2H), 1.73–1.43 (m, 4H). ^13^C NMR (101 MHz, DMSO-*d*_6_) δ 152.82, 149.82, 145.77, 143.21, 142.51, 128.76, 128.70, 126.14, 121.16, 115.39, 112.38, 99.29, 50.27, 47.85, 39.90, 35.22, 29.10, 28.63. UPLC/MS (*method A*): *R_t_* 2.49 min. MS (ES) C_22_H_25_N_3_O_5_S requires 443, found 267 [M–CO(CH_2_)_4_Ph)]^−^. HRMS C_22_H_26_N_3_O5S [M + H]^+^: calculated 444.1593, measured 444.1588, Δppm −1.1.

#### Synthesis of *tert*-Butyl 4-[2-Oxo-3-(4-phenylbutylcarbamoyl)-1,3-benzoxazol-6-yl]piperazine-1-carboxylate (**23d**)

Compound **23d** was prepared according to general procedure D (method A) using **50d** (0.10 g, 0.31 mmol), 4-phenylbutyl isocyanate (0.060 g, 0.34 mmol), and DMAP (0.042 g, 0.34 mmol) in dry MeCN (3 mL). The crude was purified by column chromatography (DCM/MeOH, 97:3) to afford **23d** as a white solid (0.130 g, 85%). UPLC/MS (*method B*): *R_t_* 2.21 min, MS (ES) C_27_H_34_N_4_O_5_ requires 494, found 495 [M + H]^+^.

#### Synthesis of 2-Oxo-*N*-(4-phenylbutyl)-6-piperazin-1-yl-1,3-benzoxazole-3-carboxamide Hydrochloride (**23e**)

Compound **23e** was prepared according to general procedure E using **23d** (0.120 g, 0.24 mmol). The reaction mixture was concentrated under reduced pressure to afford **23e** as a white solid (0.103 g, quant.). ^1^H NMR (400 MHz, DMSO-*d*_6_) δ 9.53 (bs, 2H), 8.06 (t, *J* = 5.8 Hz, 1H), 7.72 (d, *J* = 8.8 Hz, 1H), 7.33–7.24 (m, 2H), 7.24–7.11 (m, 4H), 6.90 (dd, *J* = 8.9, 2.4 Hz, 1H), 3.44–3.35 (m, 4H), 3.32 (q, *J* = 6.4 Hz, 2H), 3.27–3.15 (m, 4H), 2.62 (t, *J* = 7.2 Hz, 2H), 1.76–1.49 (m, 4H). ^13^C NMR (101 MHz, DMSO-*d*_6_) δ 152.35, 149.33, 147.49, 142.50, 142.02, 128.28, 128.22, 125.66, 121.16, 114.71, 112.07, 98.98, 45.99, 42.35, 39.42, 34.74, 28.62, 28.14. UPLC/MS (*method A*): *R_t_* 2.12 min, MS (ES) C_22_H_26_N_4_O_3_ requires 394, found 395 [M + H]^+^. HRMS C_22_H_27_N_4_O_3_ [M + H]^+^: calculated 395.2083, measured 395.2086, Δppm 1.4.

#### Synthesis of 6-(4-Methylpiperazin-1-yl)-2-oxo-*N*-(4-phenylbutyl)-1,3-benzoxazole-3-carboxamide (**23f**)

Compound **23f** was prepared according to general procedure D (method A) using **50f** (0.060 g, 0.26 mmol) and 4-phenylbutyl isocyanate (0.088 mL, 0.090 g, 0.51 mmol) in dry MeCN (3 mL). The crude was purified by column chromatography (DCM/MeOH, 98:2) to afford **23f** as a white powder (0.080 g, 75%). ^1^H NMR (400 MHz, CDCl_3_) δ 7.99 (t, *J* = 5.8 Hz, 1H), 7.93–7.85 (m, 1H), 7.33–7.23 (m, overlapped with CDCl_3_ signal, 2H), 7.22–7.14 (m, 3H), 6.86–6.77 (m, 2H), 3.43 (q, *J* = 6.4 Hz, 2H), 3.37–3.18 (m, 4H), 2.83–2.70 (m, 4H), 2.67 (t, *J* = 7.2 Hz, 2H) 2.47 (s, 3H), 1.80–1.61 (m, 4H). ^13^C NMR (101 MHz, CDCl_3_) δ 153.52, 150.03, 142.90, 142.06, 128.55, 128.52, 126.01, 115.93, 113.26, 99.01, 54.79, 49.32, 45.66, 40.21, 35.60, 29.23, 28.71. UPLC/MS (*method A*): *R_t_* 2.23 min. MS (ES) C_23_H_28_N_4_O_3_ requires 408, found 409 [M + H]^+^. HRMS C_23_H_29_N_4_O_3_ [M + H]^+^: calculated 409.224, measured 409.224, Δppm 0.0.

#### Synthesis of 6-(4-Ethylpiperazin-1-yl)-2-oxo-*N*-(4-phenylbutyl)-1,3-benzoxazole-3-carboxamide (**23g**)

Compound **23g** was prepared according to general procedure D (method A) using **50g** (0.100 g, 0.40 mmol) and 4-phenylbutyl isocyanate (0.077 g, 0.44 mmol) in dry MeCN (4 mL). The crude was purified by column chromatography (DCM/MeOH, 95:5) to afford **23g** as a yellow solid (0.109 g, 64%). ^1^H NMR (400 MHz, CDCl_3_) δ 8.00 (t, *J* = 5.8 Hz, 1H), 7.87 (d, *J* = 9.5 Hz, 1H), 7.32–7.23 (m, overlapped with CDCl_3_ signal, 2H), 7.22–7.14 (m, 3H), 6.83–6.77 (m, 2H), 3.43 (q, *J* = 6.6 Hz, 2H), 3.24–3.16 (m, 4H), 2.66 (t, *J* = 7.3 Hz, 2H), 2.64–2.59 (m, 4H), 2.49 (q, *J* = 7.2 Hz, 2H), 1.78–1.60 (m, overlapped with H_2_O signal, 4H), 1.14 (t, *J* = 7.2 Hz, 3H). ^13^C NMR (101 MHz, CDCl_3_) δ 153.57, 150.07, 149.47, 142.92, 142.06, 128.54, 128.50, 125.99, 120.71, 115.82, 112.81, 98.52, 52.79, 52.44, 49.78, 40.18, 35.59, 29.23, 28.70, 12.10. UPLC/MS (*method A*): *R_t_* 2.24 min, MS (ES) C_24_H_30_N_4_O_3_ requires 422, found 423 [M + H]^+^. HRMS C_24_H_31_N_4_O_3_ [M + H]^+^: calculated 423.2396, measured 423.2397, Δppm 0.2.

#### Synthesis of 6-(4-Isopropylpiperazin-1-yl)-2-oxo-*N*-(4-phenylbutyl)-1,3-benzoxazole-3-carboxamide (**23h**)

Compound **23h** was prepared according to general procedure D (method A) using **50h** (0.100 g, 0.38 mmol) and 4-phenylbutyl isocyanate (0.074 g, 0.42 mmol) in dry MeCN (4 mL). The crude was purified by column chromatography (DCM/MeOH, 95:5) to afford **23h** as a pink solid (0.050 g, 30%). ^1^H NMR (400 MHz, , CDCl_3_) δ 8.00 (t, *J* = 5.7 Hz, 1H), 7.87 (d, *J* = 9.5 Hz, 1H), 7.32–7.23 (m, overlapped with CDCl_3_ signal, 2H), 7.22–7.14 (m, 3H), 6.83–6.77 (m, 2H), 3.43 (q, *J* = 6.7 Hz, 2H), 3.27–3.11 (m, 4H), 2.79–2.69 (m, 5H), 2.67 (t, *J* = 7.3 Hz, 2H), 1.80–1.60 (m, 4H), 1.10 (d, *J* = 6.5 Hz, 6H). ^13^C NMR (101 MHz, , CDCl_3_) δ 153.56, 150.07, 149.43, 142.90, 142.06, 128.53, 128.49, 125.99, 120.77, 115.82, 112.89, 98.57, 54.86, 49.96, 48.65, 40.17, 35.59, 29.22, 28.69, 18.59. UPLC/MS (*method A*): *R_t_* 2.31 min, MS (ES) C_25_H_32_N_4_O_3_ requires 436, found 437 [M + H]^+^. HRMS C_25_H_33_N_4_O_3_ [M + H]^+^: calculated 437.2553, measured 437.2557, Δppm 0.9.

#### Synthesis of 6-(4-Isobutylpiperazin-1-Yl)-2-oxo-*N*-(4-phenylbutyl)-1,3-benzoxazole-3-carboxamide (**23i**)

Compound **23i** was prepared according to general procedure D (method A) using **50i** (0.100 g, 0.36 mmol) and 4-phenylbutyl isocyanate (0.07 mL, 0.07 g, 0.4 mmol) in dry MeCN (4 mL). The crude was purified by column chromatography (DCM/MeOH, 95:5) to afford **23i** as a white solid (0.116 g, 72%). ^1^H NMR (400 MHz, CDCl_3_) δ 8.00 (t, *J* = 5.7 Hz, 1H), 7.87 (d, *J* = 9.5 Hz, 1H), 7.32–7.23 (m, overlapped with CDCl_3_ signal, 2H), 7.22–7.14 (m, 3H), 6.83–6.77 (m, 2H), 3.43 (q, *J* = 6.7 Hz, 2H), 3.26–3.10 (m, 4H), 2.67 (t, *J* = 7.2 Hz, 2H), 2.61–2.46 (m, 4H), 2.22–2.06 (m, 2H), 1.90–1.78 (m, 1H), 1.77–1.62 (m, 4H), 0.93 (d, *J* = 6.6 Hz, 6H). ^13^C NMR (101 MHz, CDCl_3_) δ 150.11, 149.62, 142.93, 142.08, 128.55, 128.51, 126.00, 115.79, 112.76, 98.45, 66.91, 53.51, 49.79, 40.19, 35.60, 29.24, 28.71, 25.56, 21.06. UPLC/MS (*method B*): *R_t_* 2.08 min. MS (ES) C_26_H_34_N_4_O_3_ requires 450, found 451 [M + H]^+^. HRMS C_26_H_35_N_4_O_3_ [M + H]^+^: calculated 451.2719, measured: 451.2716, Δppm 1.6.

#### Synthesis of 4-(3-Benzyloxy-4-nitrophenyl)-1-methylpiperazin-2-one (**52a**)

Compound **52a** was prepared according to general procedure G using 2-benzyloxy-4-fluoro-1-nitrobenzene (0.490 g, 2.00 mmol), **51a** (0.300 g, 2.00 mmol), and Et_3_N (0.55 mL, 0.405 g, 4.00 mmol) in dry MeCN (20 mL), heating at reflux for 16 h. The crude was purified by column chromatography (EtOAc) to afford **52a** as a yellow solid (0.350 g, 60%). ^1^H NMR (400 MHz, CDCl_3_) δ 8.03 (d, *J* = 9.3 Hz, 1H), 7.51 (d, *J* = 7.2 Hz, 2H), 7.40 (t, *J* = 7.4 Hz, 2H), 7.33 (dd, *J* = 8.4, 6.3 Hz, 1H), 6.38 (dd, *J* = 9.3, 2.6 Hz, 1H), 6.29 (d, *J* = 2.5 Hz, 1H), 5.23 (s, 2H), 3.98 (s, 2H), 3.61 (dd, *J* = 6.5, 4.2 Hz, 2H), 3.50 (dd, *J* = 6.5, 4.2 Hz, 2H), 3.07 (s, 3H). UPLC/MS (*method A*): *R_t_* 1.98 min, MS (ES) C_18_H_19_N_3_O_4_ requires 341, found 342 [M + H]^+^.

#### Synthesis of 1-(3-Benzyloxy-4-nitrophenyl)-4-methylpiperazin-2-one (**52b**)

To a solution of 2-benzyloxy-4-bromo-1-nitrobenzene (1.00 g, 3.25 mmol, 1.0 equiv.) in degassed 1,4-dioxane (26 mL) were added **51b** (0.410 g, 3.6 mmol, 1.1 equiv.), K_3_PO_4_ (1.38 g, 6.50 mmol, 2.0 equiv.), and *N*,*N*′-dimethylethylenediamine (0.06 g, 0.07 mL, 0.65 mmol, 0.2 equiv.). The reaction mixture was degassed for another 15 min, and then copper(I) iodide (0.06 g, 0.33 mmol, 0.1 equiv.) was added. The reaction was refluxed for 24 h, then diluted with EtOAc, and filtered through a pad of Celite. The filtrate was concentrated under reduced pressure, and the crude was purified by column chromatography (EtOAc) to afford **52b** as an orange solid (0.554 g, 50%). ^1^H NMR (400 MHz, CDCl_3_) δ 7.93 (d, *J* = 8.8 Hz, 1H), 7.46 (d, *J* = 7.3 Hz, 2H), 7.43–7.36 (m, 2H), 7.36–7.30 (m, 2H), 6.97 (dd, *J* = 8.8, 2.1 Hz, 1H), 5.22 (s, 2H), 3.72 (t, *J* = 5.1 Hz, 2H), 3.33 (s, 2H), 2.83 (s, 2H), 2.43 (s, 3H). UPLC/MS (*method A*): *R_t_* 1.74 min. MS (ES) C_18_H_19_N_3_O_4_ requires 341, found 342 [M + H]^+^.

#### Synthesis of 4-(4-Amino-3-hydroxyphenyl)-1-methylpiperazin-2-one (**49i**)

Compound **49i** was prepared according to general procedure B (method A) using **52a** (0.341 g, 1.00 mmol). UPLC/MS (*method A*): *R_t_* 0.89 min. MS (ES) C_11_H_15_N_3_O_2_ requires 221, found 222 [M + H]^+^.

#### Synthesis of 1-(4-Amino-3-hydroxyphenyl)-4-methylpiperazin-2-one (**49j**)

Compound **49j** was prepared according to general procedure B (method A) using **52b** (0.50 g, 1.46 mmol). UPLC/MS (*method C*): *R_t_* 1.42 min. MS (ES) C_11_H_15_N_3_O_2_ requires 221, found 222 [M + H]^+^.

#### Synthesis of 6-(4-Methyl-3-oxo-piperazin-1-yl)-3*H*-1,3-benzoxazol-2-one (**50j**)

Compound **50j** was prepared according to general procedure C using **49i** (0.221 g, 1.00 mmol) and CDI (0.162 g, 1.00 mmol) in dry MeCN (10 mL). The crude was purified by column chromatography (DCM/MeOH, 90:10) to afford **50j** as a violet solid (0.197 g, 80% over two steps). ^1^H NMR (400 MHz, CDCl_3_) δ 6.95 (d, *J* = 8.5 Hz, 1H), 6.82 (d, *J* = 2.3 Hz, 1H), 6.67 (dd, *J* = 2.3, 8.5 Hz, 1H), 3.81 (s, 2H), 3.48 (dd, *J* = 3.4, 5.8 Hz, 2H), 3.42 (dd, *J* = 4.0, 6.2 Hz, 2H), 3.04 (s, 3H). UPLC/MS (*method A*): *R_t_* 1.16 min. MS (ES) C_12_H_13_N_3_O_3_ requires 247, found 248 [M + H]^+^.

#### Synthesis of 6-(4-Methyl-2-oxo-piperazin-1-yl)-3*H*-1,3-benzoxazol-2-one (**50k**)

Compound **50k** was prepared according to general procedure C using **49j** (0.323 g, 1.46 mmol) and CDI (0.240 g, 1.46 mmol) in dry MeCN (15 mL). The crude was purified by column chromatography (DCM/MeOH, 80:20) to afford **50k** as orange oil (0.270 g, 70% over two steps). ^1^H NMR (400 MHz, CDCl_3_) δ 7.73–7.65 (m, 1H), 7.03–6.86 (m, 2H), 3.76–3.66 (m, 2H), 3.29 (s, 2H), 2.87–2.68 (m, 2H), 2.42 (s, 4H). UPLC/MS (*method A*): *R_t_* 1.16 min. MS (ES) C_12_H_13_N_3_O_3_ requires 247, found 248 [M + H]^+^.

#### Synthesis of 6-(4-Methyl-3-oxo-piperazin-1-yl)-2-oxo-*N*-(4-phenylbutyl)-1,3-benzoxazole-3-carboxamide (**23j**)

Compound **23j** was prepared according to general procedure D (method A) using **50j** (0.170 g, 0.69 mmol) and 4-phenylbutyl isocyanate (0.13 mL, 0.130 g, 0.76 mmol) in dry MeCN (7 mL). The crude was purified by column chromatography (DCM/MeOH, 90:10) to afford **23j** as a white solid (0.060 g, 20%). ^1^H NMR (400 MHz, CDCl_3_) δ 7.98 (t, *J* = 5.3 Hz, 1H), 7.93 (d, *J* = 8.7 Hz, 1H), 7.32–7.23 (m, overlapped with CDCl_3_ signal, 2H), 7.21–7.14 (m, 3H), 6.82–6.73 (m, 2H), 3.85 (s, 2H), 3.54–3.46 (m, 4H), 3.44 (q, *J* = 6.7 Hz, 2H), 3.05 (s, 3H), 2.67 (t, *J* = 7.1 Hz, 2H), 1.77–1.62 (m, 4H). ^13^C NMR (101 MHz, CDCl_3_) δ 128.54 (2C), 126.05, 120.78, 115.97, 107.97, 47.76, 46.79, 40.34, 35.58, 35.35, 29.15, 28.67. UPLC/MS (*method A*): *R_t_* 2.32 min. MS (ES) C_23_H_26_N_4_O_4_ requires 422, found 423 [M + H]^+^. HRMS C_23_H_27_N_4_O_4_ [M + H]^+^: calculated 423.2032, measured: 423.202, Δppm −2.8.

#### Synthesis of 6-(4-Methyl-2-oxo-piperazin-1-yl)-2-oxo-*N*-(4-phenylbutyl)-1,3-benzoxazole-3-carboxamide (**23k**)

Compound **23k** was prepared according to general procedure D (method A) using **50k** (0.288 g, 1.17 mmol) and 4-phenylbutyl isocyanate (0.22 mL, 0.225 g, 1.28 mmol) in dry MeCN (8 mL). The crude was purified by column chromatography (DCM/MeOH, 90:10) to afford **23k** as a white solid (0.049 g, 10%). ^1^H NMR (400 MHz, CDCl_3_) δ 8.08 (d, *J* = 8.6 Hz, 1H), 7.98 (t, *J* = 5.5 Hz, 1H), 7.31–7.26 (m, overlapped with CDCl_3_ signal, 3H), 7.24–7.13 (m, 4H), 3.76 (s, 2H), 3.44 (q, *J* = 6.7 Hz, 2H), 3.33 (s, 2H), 2.93–2.80 (m, 2H), 2.67 (t, *J* = 7.2 Hz, 2H), 2.45 (s, 3H), 1.79–1.63 (m, 4H).^13^C NMR (101 MHz, CDCl_3_) δ 153.26, 149.71, 142.03, 141.92, 128.56, 128.55, 126.92, 126.05, 122.56, 116.07, 109.05, 59.54, 52.08, 50.30, 45.11, 40.32, 35.60, 29.18, 28.70. UPLC/MS (*method A*): *R_t_* 1.94 min. MS (ES) C_23_H_26_N_4_O_4_ requires 422, found 423 [M + H]^+^. HRMS C_23_H_27_N_4_O_4_ [M + H]^+^: calculated 423.2032, measured: 423.2026, Δppm −1.4.

#### Synthesis of *tert*-Butyl 4-(4-Hydroxy-3-nitrophenyl)-3,6-dihydro-2*H*-pyridine-1-carboxylate (**32a**)

Compound **32a** was prepared according to general procedure A using 4-bromo-2-nitrophenol (0.500 g, 2.29 mmol), **18c** (0.92 g, 2.98 mmol), PdCl_2_(PPh_3_)_4_ (0.016 g, 0.023 mmol), and 2 M Na_2_CO_3_ (2.87 mL, 5.73 mmol) in degassed 1,4-dioxane (25 mL). The crude was purified by column chromatography (heptane/EtOAc, 90:10) to afford **32a** as yellow oil (0.700 g, 95%). ^1^H NMR (400 MHz, CDCl_3_) δ 10.54 (s, 1H), 8.07 (d, *J* = 2.2 Hz, 1H), 7.65 (dd, *J* = 8.8, 2.3 Hz, 1H), 7.14 (d, *J* = 8.8 Hz, 1H), 6.07 (s, 1H), 4.09 (d, *J* = 2.8 Hz, 2H), 3.65 (t, *J* = 5.7 Hz, 2H), 3.53 (t, *J* = 5.8 Hz, 2H), 1.47 (s, 9H). UPLC/MS (*method B*): *R_t_* 1.48 min. MS (ES) C_16_H_20_N_2_O_5_ requires 320, found 319 [M–H]^−^.

#### Synthesis of *tert*-Butyl 4-(3-Amino-4-hydroxyphenyl)piperidine-1-carboxylate (**32b**)

Compound **32b** was prepared according to general procedure B (method A) using **32a** (0.078 g, 0.24 mmol). UPLC/MS (*method A*): *R_t_* 1.51 min. MS (ES) C_16_H_24_N_2_O_3_ requires 292, found 291 [M-H]^−^.

#### Synthesis of *tert*-Butyl 4-(2-Oxo-3*H*-1,3-benzoxazol-5-yl)piperidine-1-carboxylate (**33**)

Compound **33** was prepared according to general procedure C using **32b** (0.070 g, 0.24 mmol) and CDI (0.058 g, 0.36 mmol) in dry MeCN (2.5 mL). The crude was purified by column chromatography (Cy/EtOAc, 70:30) to afford **33** as a white solid (0.046 g, 60% over two steps). ^1^H NMR (400 MHz, DMSO-*d*_6_) δ 11.55 (bs, 1H), 7.22–7.14 (m, 1H), 6.99–6.90 (m, 2H), 4.07 (d, *J* = 13.0 Hz, 2H), 2.98–2.56 (m, 3H), 1.84–1.65 (m, 2H), 1.54–1.44 (m, 2H), 1.42 (s, 9H). UPLC/MS (*method A*): *R_t_* 2.20 min. MS (ES) C_17_H_22_N_2_O_4_ requires 318, found 319 [M + H]^+^.

#### Synthesis of *tert*-Butyl 4-[3-(Isobutylcarbamoyl)-2-oxo-1,3-benzoxazol-5-yl]piperidine-1-carboxylate (**24a**)

Compound **24a** was prepared according to general procedure D (method B) using **33** (0.200 g, 0.63 mmol), isobutylamine (0.094 mL, 0.069 g, 0.94 mmol), and Et_3_N (0.44 mL, 0.318 g, 3.14 mmol) in dry DCM (7 mL). The crude was purified by column chromatography (Cy/EtOAc, 90:10) to afford **24a** as a white solid (0.186 g, 70%). ^1^H NMR (400 MHz, CDCl_3_) δ 8.16 (t, *J* = 5.8 Hz, 1H), 7.98 (d, *J* = 1.8 Hz, 1H), 7.18 (d, *J* = 8.3 Hz, 1H), 7.08 (dd, *J* = 8.4, 1.8 Hz, 1H), 4.27 (d, *J* = 13.2 Hz, 2H), 3.28 (dd, *J* = 6.8, 5.9 Hz, 2H), 2.89–2.65 (m, 3H), 2.02–1.76 (m, 3H), 1.72–1.59 (m, 2H), 1.50 (s, 9H), 1.02 (d, *J* = 6.7 Hz, 6H). UPLC/MS (*method B*): *R_t_* 2.07 min. MS (ES) C_22_H_31_N_3_O_5_ requires 417, found 418 [M + H]^+^.

#### Synthesis of *N*-Isobutyl-2-oxo-5-(4-piperidyl)-1,3-benzoxazole-3-carboxamide Hydrochloride (**24b**)

Compound **24b** was prepared according to general procedure E using **24a** (0.160 g, 0.38 mmol). The crude was triturated with Et_2_O to afford **24b** as a white solid (0.128 g, 95%). ^1^H NMR (400 MHz, DMSO-*d*_6_) δ 9.16 (bs, 1H), 8.96 (bs, 1H), 8.14 (t, *J* = 5.9 Hz, 1H), 7.83 (d, *J* = 1.8 Hz, 1H), 7.39 (d, *J* = 8.3 Hz, 1H), 7.13 (dd, *J* = 8.4, 1.9 Hz, 1H), 3.37 (s, 1H), 3.17 (t, *J* = 6.4 Hz, 2H), 3.08–2.81 (m, 3H), 2.00–1.77 (m, 5H), 0.92 (d, *J* = 6.7 Hz, 6H). UPLC/MS (*method A*): *R_t_* 1.78 min. MS (ES) C_17_H_23_N_3_O_3_ requires 317, found 318 [M + H]^+^.

#### Synthesis of *N*-Isobutyl-5-(1-methyl-4-piperidyl)-2-oxo-1,3-benzoxazole-3-carboxamide (**24c**)

Compound **24c** was prepared according to general procedure F using **24b** (0.088 g, 0.25 mmol), 37% aqueous solution of formaldehyde (0.038 mL, 1.25 mmol), NaBH(OAc)_3_ (0.106 g, 0.5 mmol), and AcOH (0.03 mL, 0.024 g, 0.4 mmol) in dry MeCN (3 mL). The crude was triturated with Et_2_O to afford **24c** as a white solid (0.07 g, 83%). ^1^H NMR (400 MHz, DMSO-*d*_6_) δ 8.14 (t, *J* = 5.1 Hz, 1H), 7.80 (s, 1H), 7.33 (d, *J* = 8.3 Hz, 1H), 7.15 (d, *J* = 8.4 Hz, 1H), 3.15–3.07 (t, *J* = 6.4 Hz, 2H), 2.94–2.79 (m, 2H), 2.59–2.43 (m, overlapped with DMSO signal, 1H), 2.20 (s, 3H), 2.06–1.91 (m, 2H), 1.91–1.79 (m, 1H), 1.79–1.54 (m, 4H), 0.92 (d, *J* = 6.7 Hz, 6H). ^13^C NMR (101 MHz, DMSO-*d*_6_) δ 152.56, 149.50, 142.88, 139.85, 128.14, 122.36, 112.74, 109.60, 55.68, 46.79, 46.12, 41.27, 33.32, 27.90, 19.77. UPLC/MS (*method A*): *R_t_* 1.81 min. MS (ES) C_18_H_25_N_3_O_3_ requires 331, found 332 [M + H]^+^. HRMS C_18_H_26_N_3_O_3_ [M + H]^+^: calculated 332.1974, measured: 332.1964, Δppm −3.0.

#### Synthesis of *tert*-Butyl 4-Hydroxy-4-(2-oxo-3*H*-1,3-benzoxazol-7-yl)piperidine-1-carboxylate (**45**)

Compound **45** was prepared according to general procedure I using **42b** (0.793 g, 3.98 mmol), 7-bromo-3*H*-1,3-benzoxazol-2-one (0.500 g, 2.34 mmol), MeMgBr (1.17 mL, 0.418 g, 3.51 mmol, 3 M in Et_2_O), and *n-*BuLi (1.12 mL, 2.81 mmol, 2.5 M in hexanes) in dry THF (25 mL). The crude was purified by column chromatography (Cy/EtOAc, 35:65) to afford **45** as a white solid (0.345 g, 44%). ^1^H NMR (400 MHz, DMSO-*d*_6_) δ 11.60 (bs, 1H), 7.26 (dd, *J* = 8.1, 1.3 Hz, 1H), 7.12 (t, *J* = 7.9 Hz, 1H), 6.97 (dd, *J* = 7.7, 1.3 Hz, 1H), 5.34 (s, 1H), 3.97–3.78 (m, 2H), 3.25–2.99 (m, 2H), 2.09 (td, *J* = 13.1, 4.7 Hz, 2H), 1.63–1.52 (m, 2H), 1.43 (s, 9H). UPLC/MS (*method A*): *R_t_* 1.76 min. MS (ES) C_17_H_22_N_2_O_5_ requires 334, found 335 [M + H]^+^.

#### Synthesis of 7-(1,2,3,6-Tetrahydropyridin-4-yl)-3*H*-1,3-benzoxazol-2-one (**46a**)

Compound **46a** was prepared according to general procedure L using **45** (0.345 g, 1.03 mmol). The crude was purified by SCX to afford **46a** as a brown solid (quant.). UPLC/MS (*method A*): *R_t_* 0.95 min. MS (ES) C_12_H_12_N_2_O_2_ requires 216, found 217 [M + H]^+^.

#### Synthesis of 7-(1-Methyl-3,6-dihydro-2*H*-pyridin-4-yl)-3*H*-1,3-benzoxazol-2-one (**46b**)

Compound **46b** was prepared according to general procedure F using **46a** (0.108 g, 0.5 mmol), NaBH(OAc)_3_ (0.318 g, 1.50 mmol), AcOH (0.03 mL, 0.030 g, 0.50 mmol), and 37% aqueous solution of formaldehyde (0.080 mL, 2.5 mmol) in dry MeCN (5 mL). The crude was used in the next step without further purification. ^1^H NMR (400 MHz, DMSO-*d*_6_) δ 6.87 (d, *J* = 7.76 Hz, 1H), 6.80–6.74 (m, 2H), 6.36 (t, *J* = 3.49 Hz, 1H), 3.07–3.00 (m, 2H), 2.58–2.47 (m, overlapped with DMSO signal, 4H), 2.27 (s, 3H). UPLC/MS (*method A*): *R_t_* 0.98 min. MS (ES) C_13_H_14_N_2_O_2_ requires 230, found 231 [M + H]^+^.

#### Synthesis of 7-(1-Methyl-4-piperidyl)-3*H*-1,3-benzoxazol-2-one (**47**)

Compound **47** was prepared according to general procedure B (method B) using **46b** (0.115 g, 0.50 mmol). The crude was used in the next step without further purification. ^1^H NMR (400 MHz, DMSO-*d*_6_) δ 7.12–7.06 (m, 1H), 6.94 (t, *J* = 7.4, 1.2 Hz, 2H), 2.98 (d, *J* = 11.8, 3.4 Hz, 2H), 2.82–2.69 (m, 1H), 2.31 (s, 3H), 2.25–2.11 (m, 2H), 1.88–1.72 (m, 4H). UPLC/MS (*method A*): *R_t_* 0.98 min, MS (ES) C_13_H_16_N_2_O_2_ requires 232, found 233 [M + H]^+^.

#### Synthesis of *N*-Isobutyl-7-(1-methyl-4-piperidyl)-2-oxo-1,3-benzoxazole-3-carboxamide (**25**)

Compound **25** was prepared according to general procedure D (method B) using **47** (0.116 g, 0.5 mmol), isobutylamine (0.014 g, 0.19 mmol), and Et_3_N (0.15 mL, 0.111 g, 1.10 mmol) in dry DCM (7 mL). The crude was purified by column chromatography (DCM/MeOH, 92:8) to afford **25** as a pink solid (0.050 g, 30% over three steps). ^1^H NMR (400 MHz, CDCl_3_) δ 8.14 (t, *J* = 5.0 Hz, 1H), 7.91 (dd, *J* = 8.0, 1.3 Hz, 1H), 7.21 (t, *J* = 8.0 Hz, 1H), 7.11 (dd, *J* = 8.1, 1.3 Hz, 1H), 3.30–3.21 (m, 2H), 3.07–2.96 (m, 2H), 2.96–2.82 (m, 1H), 2.35 (s, 3H), 2.13 (td, *J* = 11.5, 3.2 Hz, 2H), 2.03–1.81 (m, 5H), 0.99 (d, *J* = 6.7 Hz, 6H). ^13^C NMR (101 MHz, CDCl_3_) δ 153.43, 150.10, 139.67, 128.76, 127.90, 125.28, 122.48, 113.52, 56.18, 47.71, 46.50, 35.66, 31.80, 28.61, 20.18. UPLC/MS (*method A*): *R_t_* 1.79 min, MS (ES) C_18_H_25_N_3_O_3_ requires 331, found 332 [M + H]^+^. HRMS C_18_H_26_N_3_O_3_ [M + H]^+^: calculated 332.1974, measured 332.1967, Δppm −2.1.

#### Synthesis of 2-(3-Benzyloxy-4-nitrophenyl)pyridine (**35**)

Compound **35** was prepared according to general procedure A using 2-benzyloxy-4-bromo-1-nitrobenzene (0.309 g, 1.00 mmol), **34** (0.174 g, 1.10 mmol), Pd(dppf)Cl_2_ (0.146 g, 0.2 mmol), KOAc (0.196 g, 2 mmol), and 2 M Na_2_CO_3_ (1.30 mL, 2.50 mmol) in degassed 1,4-dioxane (15 mL). The crude was purified by column chromatography (Cy/EtOAc, 80:20) to afford **35** as a yellow solid (0.121 g, 40%). ^1^H NMR (400 MHz, DMSO-*d*_6_) δ 8.75 (ddd, *J* = 4.8, 1.7, 0.9, 1H), 8.14 (d, *J* = 8.0 Hz, 1H), 8.12 (d, *J* = 1.6 Hz, 1H), 8.03 (d, *J* = 8.5 Hz, 1H), 7.98 (td, *J* = 7.8, 1.8 Hz, 1H), 7.85 (dd, *J* = 8.5, 1.7 Hz, 1H), 7.54–7.32 (m, 6H), 5.45 (s, 2H). UPLC/MS (*method A*): *R_t_* 2.47 min, MS (ES) C_18_H_14_N_2_O_3_ requires 306, found 307 [M + H]^+^.

#### Synthesis of 2-Amino-5-(2-piperidyl)phenol (**36**)

Compound **36** was prepared according to general procedure B (method B) using **35** (0.520 g, 1.69 mmol). UPLC/MS (*method A*): *R_t_* 0.93 min. MS (ES) C_11_H_16_N_2_O requires 192, found 193 [M + H]^+^.

#### Synthesis of 6-(2-Piperidyl)-3*H*-1,3-benzoxazol-2-one (**37a**)

Compound **37a** was prepared according to general procedure C using **36** (0.390 g, 1.69 mmol) and CDI (0.274 g, 1.69 mmol) in dry MeCN (17 mL). The crude was used in the next step without further purification. UPLC/MS (*method A*): *R_t_* 1.05 min. MS (ES) C_12_H_14_N_2_O_2_ requires 218, found 219 [M + H]^+^.

#### Synthesis of 6-(1-Methyl-2-piperidyl)-3*H*-1,3-benzoxazol-2-one (**37b**)

Compound **37b** was prepared according to general procedure F using **37a** (0.368 g, 1.69 mmol), 37% aqueous solution of formaldehyde (0.09 mL, 3.38 mmol), NaBH(OAc)_3_ (1.075 g, 5.07 mmol), and AcOH (0.15 mL, 0.162 g, 2.70 mmol) in dry MeCN (9 mL). The crude was purified by SCX to afford **37b** as a white solid (0.169 g, 43% over three steps). ^1^H NMR (400 MHz, DMSO-*d*_6_) δ 7.64 (bs, 1H), 7.14 (d, *J* = 1.2 Hz, 1H), 7.06–6.95 (m, 2H), 3.01–2.85 (m, 1H), 2.74 (dd, *J* = 10.8, 2.7 Hz, 1H), 2.01 (td, *J* = 11.6, 3.2 Hz, 1H), 1.88 (s, 3H), 1.78–1.66 (m, 1H), 1.67–1.50 (m, 3H), 1.50–1.36 (m, 1H), 1.37–1.22 (m, 1H). UPLC/MS (*method A*): *R_t_* 1.04 min. MS (ES) C_13_H_16_N_2_O_2_ requires 232, found 233 [M + H]^+^.

#### Synthesis of (±)-*N*-Isobutyl-6-(1-methyl-2-piperidyl)-2-oxo-1,3-benzoxazole-3-carboxamide (**26**)

Compound **26** was prepared according to general procedure D (method B) using **37b** (0.170 g, 0.73 mmol) and isobutylamine (0.160 g, 2.19 mmol) in dry DCM (10 mL). The crude was purified by column chromatography (DCM/MeOH, 92:8) to afford **26** as a white solid (0.069 g, 29%). ^1^H NMR (400 MHz, CDCl_3_) δ 8.10 (t, *J* = 5.3 Hz, 1H), 7.94 (d, *J* = 8.2 Hz, 1H), 7.30–7.23 (m, overlapped with CDCl_3_ signal, 1H), 7.20 (dd, *J* = 8.3, 1.6 Hz, 1H), 3.25 (dd, *J* = 6.8, 5.8 Hz, 2H), 3.09–3.00 (m, 1H), 2.95 (dd, *J* = 11.1, 2.8 Hz, 1H), 2.80 (dd, *J* = 11.1, 2.8 Hz, 1H), 2.18–2.05 (m, 1H), 1.98 (s, 3H), 1.96–1.85 (m, 1H), 1.85–1.76 (m, 1H), 1.76–1.65 (m, 2H), 1.61–1.47 (m, 1H), 1.42–1.28 (m, 1H), 0.98 (d, *J* = 6.7 Hz, 6H). ^13^C NMR (101 MHz, CDCl_3_) δ 153.56, 150.07, 142.34, 142.16, 126.95, 124.20, 115.37, 108.82, 70.79, 57.48, 47.68, 44.59, 36.37, 28.60, 26.12, 24.94, 20.15. UPLC/MS (*method A*): *R_t_* 1.88 min, MS (ES) C_18_H_25_N_3_O_3_ requires 331, found 332 [M + H]^+^. HRMS C_18_H_26_N_3_O_3_ [M + H]^+^: calculated 332.1971, measured 332.1974, Δppm −0.9.

#### Synthesis of *tert*-Butyl 5-(3-Hydroxy-4-nitrophenyl)-3,4-dihydro-2*H*-pyridine-1-carboxylate (**39**)

Compound **39** was prepared according to general procedure A using **38** (0.834 g, 2.7 mmol), 5-bromo-2-nitrophenol (0.530 g, 2.43 mmol), Pd(PPh_3_)_4_ (0.156 g, 0.135 mmol), and 2 M Na_2_CO_3_ (3.04 mL, 6.075 mmol) in degassed 1,4-dioxane (27 mL). The crude was purified by column chromatography (Cy/EtOAc, 90:10) to afford **39** as a yellow solid (0.460 g, 53%). ^1^H NMR (400 MHz, CDCl_3_) δ 10.79 (s, 1H), 7.99 (d, *J* = 8.8 Hz, 1H), 7.82–7.48 (m, 1H), 7.11–6.94 (m, 2H), 3.62 (s, 2H), 2.42 (t, *J* = 6.0 Hz, 2H), 1.98 (p, *J* = 6.1 Hz, 2H), 1.54 (s, 9H). UPLC/MS (*method B*): *R_t_* 1.86 min. MS (ES) C_16_H_20_N_2_O_5_ requires 320, found 321 [M + H]^+^.

#### Synthesis of *tert*-Butyl 3-(4-Amino-3-hydroxyphenyl)piperidine-1-carboxylate (**40**)

Compound **40** was prepared according to general procedure B (method A) using **39** (0.450 g, 1.41 mmol). UPLC/MS (*method B*): *R_t_* 0.73 min. MS (ES) C_16_H_24_N_2_O_3_ requires 292, found 291 [M-H]^−^.

#### Synthesis of *tert*-Butyl 3-(2-Oxo-3*H*-1,3-benzoxazol-6-yl)piperidine-1-carboxylate (**49a**)

Compound **49a** was prepared according to general procedure C using **40** (0.412 g, 1.41 mmol) and CDI (0.229 g, 1.41 mmol) in dry MeCN (14 mL). The crude was purified by column chromatography (Cy/EtOAc, 70:30) to afford **41a** as brown oil (0.403 g, 90% over two steps). ^1^H NMR (400 MHz, CDCl_3_) δ 10.04 (bs, 1H), 7.07–6.88 (m, 3H), 4.20–4.06 (m, 2H), 2.80–2.43 (m, 3H), 2.03–1.93 (m, 1H), 1.72 (dd, *J* = 3.2, 6.4 Hz, 1H), 1.66–1.52 (m, 2H), 1.44 (s, 9H). UPLC/MS (*method A*): *R_t_* 2.19 min. MS (ES) C_17_H_22_N_2_O_4_ requires 318, found 319 [M + H]^+^.

#### Synthesis of 6-(3-Piperidyl)-3*H*-1,3-benzoxazol-2-one Hydrochloride (**41b**)

Compound **41b** was prepared according to general procedure E using **41a** (0.185 g, 0.58 mmol). The crude was used in the next step without further purification. UPLC/MS (*method A*): *R_t_* 0.99 min. MS (ES) C_12_H_14_N_2_O_2_ requires 218, found 219 [M + H]^+^.

#### Synthesis of 6-(1-Methyl-3-piperidyl)-3*H*-1,3-benzoxazol-2-one (**41c**)

Compound **41** was prepared according to general procedure F using **41b** (0.147 g, 0.58 mmol), 37% aqueous solution of formaldehyde (0.03 mL, 1.16 mmol), NaBH(OAc)_3_ (0.370 g, 1.74 mmol), and AcOH (0.07 mL, 0.070 g, 1.16 mmol) in dry MeCN (3 mL). The crude was purified by SCX to afford **41c** as a white solid (0.094 g, 70% over two steps). ^1^H NMR (400 MHz, CDCl_3_) δ 7.09 (s, 1H), 7.02 (d, *J* = 8.2 Hz, 1H), 6.95 (d, *J* = 8.1 Hz, 1H), 3.01–2.81 (m, 3H), 2.31 (s, 3H), 2.03–1.87 (m, 3H), 1.87–1.67 (m, 2H), 1.39 (qd, *J* = 12.6, 4.2 Hz, 1H). UPLC/MS (*method A*): *R_t_* 1.81 min. MS (ES) C_13_H_16_N_2_O_2_ requires 232, found 233 [M + H]^+^.

#### Synthesis of (±)-*N*-Isobutyl-6-(1-methyl-3-piperidyl)-2-oxo-1,3-benzoxazole-3-carboxamide (**27**)

Compound **27** was prepared according to general procedure D (method B) using **41c** (0.088 g, 0.28 mmol) and isobutylamine (0.06 mL, 0.06 g, 0.84 mmol) in dry DCM (4 mL). The crude was purified by column chromatography (DCM/MeOH, 90:10) to afford **27** as a white solid (0.08 g, 68%). ^1^H NMR (400 MHz, CDCl_3_) δ 8.09 (bs, 1H), 7.96 (d, *J* = 8.9 Hz, 1H), 7.17–7.08 (m, 2H), 3.25 (d, *J* = 6.59, 2H), 3.03–2.87 (m, 3H), 2.34 (s, 3H), 2.13–1.68 (m, 6H), 1.41 (qd, *J* = 12.1, 5.5 Hz, 1H), 0.99 (d, *J* = 6.7 Hz, 6H). ^13^C NMR (101 MHz, CDCl_3_) δ 153.50, 150.05, 142.02, 141.71, 126.47, 123.99, 115.48, 108.69, 63.12, 55.85, 47.69, 46.54, 42.82, 31.21, 28.60, 25.64, 20.16. UPLC/MS (*method A*): *R_t_* 1.87 min. MS (ES) C_18_H_25_N_3_O_3_ requires 331, found 332 [M + H]^+^. HRMS C_18_H_26_N_3_O_3_ [M + H]^+^: calculated 332.1974, measured 332.1972, Δppm −0.9.

### In Vitro Pharmacological Assay

#### In Vitro *h*AC Fluorescence Assay

##### Cell Culture Conditions and Preparation of *h*AC-Enriched Lysate

HEK293 cells stably expressing *h*AC were grown in Dulbecco’s modified Eagle medium (DMEM) containing 10% FBS, 1% glutamine, 1 mM sodium pyruvate, and 500 μg mL^–1^ G418. Cells were harvested, and pellets were stored at −80 °C until lysosomal-enriched lysate preparation. Cells were suspended in 20 mM Tris HCl (pH 7.5) with 0.32 M sucrose, sonicated, and centrifuged at 800 × *g* for 30 min at 4 °C. Supernatants were then centrifuged at 12000 × *g* for 30 min at 4 °C. Pellets were resuspended in PBS (pH 7.4) and subjected to three freeze–thaw cycles at −80 °C. The suspension was finally centrifuged at 105000 × *g* for 1 h at 4 °C, and protein concentration was measured in the supernatant with the bicinchoninic acid based protein assay. This *h*AC-enriched preparation allowed us to further optimize the enzymatic assay and to use small amounts of lysate (2 μg per well) at a 5 μM substrate (Rbm14–12) around its *K*_M_ (*K*_M_ = 5.0 μM).

##### Fluorogenic *h*AC Assay

The assay was performed in Optiplate 96-well black plates, with each reaction well containing a mixture of 25 mM NaOAc buffer (pH 4.5) and a fixed amount of protein (2 μg) in a volume of 85 μL. After 10 min of preincubation with test compounds (diluted 20× from DMSO stock solutions at different concentrations), the fluorogenic probe was added (diluted 40× from EtOH stock solution, final concentration 5 μM). After 3 h of incubation at 37 °C, reactions were stopped with 50 μL of MeOH and 100 μL of a 2.5 mg mL^–1^ NaIO_4_ fresh solution in 100 mM glycine/NaOH buffer (pH 10.6). The plates were further incubated for 2 h at 37 °C in the dark, and fluorescence intensities were measured at excitation/emission wavelengths of 355/460 nm. Negative control samples consisted of the same incubation mixture in the absence of protein-enriched extracts. Data were plotted as a function of compound concentrations. IC_50_ values were calculated by nonlinear regression analysis using GraphPad Prism 5 (GraphPad Software Inc., CA, USA) applying a standard slope curve fitting. The reported IC_50_ values are the mean of at least three independent experiments performed in three technical replicates.

#### Kinetic Studies

##### Michaelis–Menten Analysis

Assay conditions for the kinetic studies were the same as those described for the fluorogenic *h*AC assay. Enzyme-enriched lysate (2 μg) was incubated with the following concentrations of substrate Rbm14–12: 0.25, 0.5, 1, 2.5, 5, 10, 12.5, and 15 μM. Compound **22m** was used at final concentrations of 100 and 400 nM. Initial velocities (*V*_0_) were determined and automatically fitted to the Michaelis–Menten equation to obtain the kinetic parameters (*K*_M_ and *V*_max_). The graph is representative of two independent experiments, each performed in three technical replicates. Graphs and data analysis were performed using GraphPad Prism 5 software (GraphPad Software Inc., CA, USA).

##### Determination of Kinetic Parameter *k*_i_/*K*_I_

*h*AC activity was measured as a function of reaction time in the presence of different concentrations of **22m**. The apparent inactivation rate constant of *h*AC (*k*_obs_) was analyzed by nonlinear square fitting each data set to the pseudo-first-order rate equation *Y* = *vi*(1 – exp(−*k*_obs_*t*))/*k*_obs_. Replotting of calculated *k*_obs_ vs [**22m**] was made, and the kinetic parameter *k*_i_/*K*_I_ was calculated by nonlinear square fitting data to the equation *Y* = *k*_i_*I*/(*K*_I_ + *I*). The graphs are representative of two independent experiments, each performed in two technical replicates.

#### In Vitro *h*ASM Assay

*h*ASM activity measurement was conducted using the fluorogenic substrate 6-hexadecanoylamino-4-methylumbelliferylphosphorylcholine (HMU-PC, Toronto Research Chemicals) at 0.5 μM and 1.3 nM purified human full-length ASM enzyme (purified in house) in a buffer containing 50 mM citrate, 150 mM NaCl, 5 mM ZnCl_2_, and 0.43 mM Triton X-100 at pH 4.7 in a final volume of 50 μL. The reaction mixtures were incubated for 45 min at rt and stopped by the addition of 150 μL of 1 M glycine at pH 12.5. The formation of the fluorescent product was monitored by a plate reader at excitation/emission wavelengths of 385/450 nm. The average *h*ASM activity was calculated from two independent experiments, each performed in two technical replicates.

#### In Vitro *h*GCase Assay

*h*GCase activity measurement was conducted using the fluorogenic substrate 4-methylumbelliferyl-β-d-glucuronide hydrate (4-MUG, Merck) at 1 mM and 5 nM purified human full-length GCase enzyme and 50 nM of its natural activator SapC (both GCase and SapC were purified in house) in a buffer containing 50 mM citric acid, 174 mM K_2_HPO_4_, 15 μM phosphatidylserine, and 0.32 mM Triton X-100 at pH 4.7 in a final volume of 50 μL. The reaction mixtures were incubated for 15 min at rt and stopped by the addition of 150 μL of 1 M glycine at pH 12.5. The cleavage of 4-MUG was monitored by a plate reader at excitation/emission wavelengths of 365/440 nm. The average *h*GCase activity was calculated from two independent experiments, each performed in two technical replicates.

#### In Vitro *h*NAAA Fluorescence Assay

##### Cell Culture and Preparation of *h*NAAA-Enriched Lysate

HEK-293 cells stably transfected with the *h*NAAA coding sequence cloned from a human spleen cDNA library (catalog no. 639124, Clontech, Mountain View, CA, USA) were used as the enzyme source. Cells were grown in Dulbecco’s modified Eagle medium (DMEM) containing 10% FBS, 1% glutamine, 1 mM sodium pyruvate, and 500 μg mL^–1^ G418. Cells were harvested, and pellets were stored at −80 °C until lysosomal-enriched lysate preparation. Cells were suspended in 20 mM Tris HCl (pH 7.4) with 0.32 M sucrose, sonicated, and centrifuged at 800 × *g* for 30 min at 4 °C. Supernatants were then ultracentrifuged at 12000 × *g* for 30 min at 4 °C. Pellets were resuspended in PBS buffer (pH 7.4) and subjected to three freeze–thaw cycles at −80 °C. The suspension was finally ultracentrifuged at 105000 × *g* for 1 h at 4 °C, supernatants were collected, protein concentration was measured, and samples were aliquoted and stored at −80 °C until use.

##### Fluorogenic *h*NAAA Assay

The assay was run in 96-well microplates (Black OptiPlate-96F; PerkinElmer, Massachusetts, USA) in a total reaction volume of 200 μL. *h*NAAA protein preparation (4.0 μg) was preincubated for 30 min with various concentrations of test compounds or vehicle control (DMSO 5%) in 100 mM citrate/phosphate buffer (pH 4.5) containing 3.0 mM DTT, 0.1% NP40 0.1%, 0.05% BSA, 150 mM NaCl. *N*-(4-Methyl-2-oxo-chromen-7-yl)-hexadecanamide (PAMCA) was used as a substrate (2.0 μM), and the reaction was carried out for 50 min at 37 °C. Fluorescence was measured with an EnVision 2014 Multilabel Reader (PerkinElmer, Massachusetts, USA) using an excitation wavelength of 355 nm and emission of 460 nm. IC_50_ values were calculated by nonlinear regression analysis of log[concentration]/inhibition curves using GraphPad Prism 5 (GraphPad Software Inc., CA, USA) applying a standard slope curve fitting. The reported IC_50_ values are the mean of at least three independent experiments performed in three technical replicates.

#### In Vitro *h*FAAH Fluorescence Assay

##### Cell Culture and Preparation of *h*FAAH-Enriched Lysate

*h*FAAH was obtained from a HEK-293 FAAH-1 overexpressing stable cell line. Cells were grown in Dulbecco’s modified Eagle medium (DMEM) containing 10% FBS, 1% penicillin/streptomycin, 1% glutamine, 1 mM sodium pyruvate, and 500 μg mL^–1^ G418. Cells were harvested, and pellets were stored at −80 °C until membrane-enriched lysate preparation. The cell pellet was resuspended in 20 mM Tris HCl (pH 7.4, 0.32 M sucrose), sonicated, and centrifuged at 1000 × *g* (10 min, 4 °C). The collected supernatant was centrifuged at 12000 × *g* for 10 min at 4 °C, and the supernatants were further centrifuged at 100000 × *g* for 1 h at 4 °C. Membrane pellets were resuspended in PBS, protein concentration was measured, and samples were aliquoted and stored at −80 °C until use.

##### Fluorogenic *h*FAAH Assay

The fluorescence assay to measure FAAH activity was performed in 96-well black plates (Black OptiPlate-96F; PerkinElmer, Massachusetts, USA): 2.5 μg of *h*FAAH membrane preparation was preincubated for 50 min at 37 °C in 190 μL of assay buffer (50 mM Tris HCl pH 7.4, 0.05% fatty acid free BSA), with 5 μL of inhibitor or 5 μL of DMSO to measure FAAH total activity. The background (no activity) samples were prepared using 190 μL of assay buffer without *h*FAAH and 5 μL of DMSO. The reaction was then started by the addition of 5 μL of substrate (AMC Arachidonyl Amide, A6855, Merck) dissolved in DMSO and used at a final concentration of 800 nM. The reaction was carried out for 45 min at 37 °C, and fluorescence was measured with an EnVision 2014 Multilabel Reader (PerkinElmer, Massachusetts, USA) (excitation wavelength 355 nm/emission wavelength 460 nm). The concentration causing half-maximal inhibition (IC_50_) was determined by nonlinear regression analysis of the log[concentration]/response curves generated with mean replicate values using a four-parameter Hill equation curve fitting with GraphPad Prism 5 (GraphPad Software Inc., CA, USA). The reported IC_50_ values are the mean of at least three independent experiments performed in three technical replicates.

#### In Vitro *h*MAGL Colorimetric Assay

The colorimetric assay to measure *h*MAGL activity was performed using an assay kit provided by Cayman Scientific (item. 705192), according to the manufacturer’s instructions. Briefly, in vitro activity was measured in 96-well plates, and DMSO was used as solvent. 10 μL of DMSO (100% initial activity wells: 100% IA) or compounds at two concentrations (1 and 10 μM) were preincubated for 5 min at rt with 150 μL of diluted assay buffer (10 mM Tris HCl, pH 7.2, containing 1 mM EDTA) containing *h*MAGL. In blank wells, 160 μL of the diluted assay buffer and 10 μL of DMSO were added. The reactions were initiated by adding 10 μL of MAGL substrate to all the wells, and plates were incubated for 10 min at rt. Absorbance values were measured at 405 nm, and percent inhibition was calculated by the following method: 100 – (Inhibitor/100% IA) × 100. The reported percentages of inhibition are the mean of at least three independent experiments performed in three technical replicates.

#### Cell Culture and Treatments

SH-SY5Y cells were purchased from Sigma Aldrich (Italy) and cultured in Dulbecco’s modified Eagle’s medium (DMEM) containing 10% fetal bovine serum at 37 °C and 5% CO_2_. Drugs were dissolved in DMSO (10 mM) and diluted in the cell culture medium with reduced serum (1%) for cell treatments.

#### *h*AC LC/MS-Based Activity Assay

*h*AC activity measurement was performed as previously described.^36,37^ Total lysates from cells were diluted in assay buffer (100 mM sodium phosphate, 0.1% Nonidet P-40, 150 mM NaCl, 3 mM DTT, 100 mM sodium citrate, pH 4.5). Reactions were started by the addition of 50 μM N-lauroyl ceramide (Nu-Chek Prep, Elysian, MN) and carried out for 1 h at 37 °C. Reactions were stopped by addition of a mixture of CHCl_3_/MeOH (2:1) containing 1 nmol of 11-lauroleic acid (Nu-Chek Prep). The organic phases were collected, dried under nitrogen, and analyzed by UPLC/MS (ACQUITY, Waters) in the negative-ion mode monitoring the reaction product (lauric acid, *m*/*z*: 199) using 11-lauroleic acid as internal standard. Lipids were eluted on an ACQUITY UPLC BEH C18 column (50 mm length, 2.1 mm ID, 1.7 μm pore size, Waters) at 0.5 mL min^–1^ for 1.5 min with a gradient of MeCN and H_2_O, both containing 0.25% acetic acid and 5 mM ammonium acetate (70 to 100% MeCN in 0.5 min, 100% MeCN for 0.5 min, 70% MeCN for 0.4 min). The column temperature was 40 °C. Electrospray ionization (ESI) was in the negative mode, capillary voltage was 1 kV, and cone voltage was 50 V. N_2_ was used as drying gas at a flow rate of 500 L h^–1^ and at a temperature of 400 °C. The [M–H]^−^ ion was monitored in the selected-ion monitoring mode (*m*/*z* values: lauric acid 199, 11-lauroleic acid 197.35). Calibration curves were generated with authentic lauric acid (Nu-Chec Prep).

#### Lipid Extraction and Ceramide Analysis

Lipid extraction and sphingolipid measurements were performed as previously described.^36,37^ Lipids were extracted with a CHCl_3_/MeOH mixture (2:1, 3 mL) containing internal standards. The organic phase was collected, dried under nitrogen, and dissolved in CHCl_3_/MeOH (1:3) for LC/MS analyses. Ceramides and sphingosine were analyzed by LC/MS/MS, using a Waters ACQUITY UPLC coupled to a Waters Xevo TQMS and interfaced with an ESI ion source. Separation was performed on a Waters ACQUITY BEH C18 1.7 μm column (2.1 × 50 mm) at 60 °C. A linear gradient of 0.1% formic acid in MeCN/isopropyl alcohol (20:80) as solvent B in 0.1% formic acid in MeCN/H_2_O (20:80) as solvent A was applied at a flow rate of 0.4 mL min^–1^. Detection of sphingolipids was performed in positive-ion mode. Capillary voltage was 3.5 kV, and cone voltage was 25 V. The source temperature and desolvation temperatures were set at 120 and 600 °C, respectively. Desolvation gas and cone gas (N_2_) flows were 800 and 20 L h^–1^, respectively. Ceramides were identified by comparison of their LC retention times and MS/MS fragmentation patterns with those of authentic standards (Avanti Polar Lipids). Multiple Reaction Monitoring (MRM) ion chromatograms were used to quantify myristoyl ceramide (C14:0, *m*/*z*: 492.5 > 264.3), palmitoyl ceramide (C16:0, *m*/*z* 520.3 > 264.3), stearoyl ceramide (C18:0, *m*/*z*: 548.3 > 264.3), lignoceroyl ceramide (C24:0, *m*/*z*: 632.3 > 264.3), and nervonoyl ceramide (C24:1 *m*/*z*: 630.3 > 264.3) using lauroyl ceramide standard (*m*/*z*: 464.5 > 264.3). Detection and analysis were controlled by Waters MassLynx software version 4.1. Sphingosine was identified by comparison of its LC retention times and MS2 fragmentation patterns with those of authentic standards (Avanti Polar Lipids). Extracted ion chromatograms were used to quantify sphingosine standard (d18:1, *m*/*z*: 300.5 > 282.5). Detection and analysis were controlled by Waters MassLynx software version 4.1. Calibration curves were prepared for every experiment.

#### Statistics

GraphPad Prism software (GraphPad Software, Inc., USA) was used for statistical analysis. Data were analyzed using the Student *t* test or one-way ANOVA followed by the Bonferroni post hoc test for multiple comparisons. Differences between groups were considered statistically significant at values of *p* < 0.05. Results are expressed as mean ± S.E.M.

#### EC_50_ Determination in Primary Fibroblast Cells from Krabbe’s Disease Patients

Cells were plated in a 6-well plate. After 24 h, cells were treated with **22m** at different concentrations for 2 h. Next, cells were washed with PBS, and cell pellets were washed, collected, and stored at −80 °C. Finally, cell pellets were lysed, and *h*AC activity in cell lysates was analyzed using the same biochemical fluorogenic assay as described for compound IC_50_ determination. Using this methodology, EC_50_ was determined to be 0.41 ± 0.1 μM. The EC_50_ value is a mean of two independent experiments, each performed in two technical replicates.

### In Vitro Physicochemical and Metabolic Stability Assays

#### Aqueous Kinetic Solubility Assay

The aqueous kinetic solubility was determined from a 10 mM MeCN stock solution of test compound in Phosphate-Buffered Saline (PBS) at pH 7.4. The study was performed by incubation of an aliquot of 10 mM MeCN stock solution in PBS (pH 7.4) at a target concentration of 250 μM. The incubation was carried out under shaking at 25 °C for 1 h followed by centrifugation at 21100 × *g* for 30 min. The supernatant was analyzed by UPLC/MS for the quantification of the dissolved compound (in μM) by UV at a specific wavelength (215 nm). The aqueous kinetic solubility (in μM) was calculated by dividing the peak area of the dissolved test compound (supernatant) by the peak area of the test compound in the reference (250 μM in MeCN) and further multiplied by the target concentration and dilution factor. The UPLC/MS analyses were performed on a Waters ACQUITY UPLC/MS system consisting of a single quadrupole detector (SQD) Mass Spectrometer (MS) equipped with an Electrospray Ionization (ESI) interface and a Photodiode Array Detector (PDA). The PDA range was 210–400 nm. ESI in positive mode was used in the mass scan range of 100–650 Da. The analyses were run on an ACQUITY UPLC BEH C18 column (50 × 2.1 mm ID, particle size 1.7 μm) with a VanGuard BEH C18 precolumn (5 × 2.1 mm ID, particle size 1.7 μm), using 10 mM NH_4_OAc in H_2_O at pH 5 adjusted with AcOH (A) and 10 mM NH_4_OAc in MeCN/H_2_O (95:5) at pH 5 (B) as the mobile phase. Values are reported as mean values of ≥2 experiments performed.

#### Chemical Stability Assay

Chemical stability of selected compounds was evaluated under physiological pH conditions (0.01 M phosphate-buffered saline, pH 7.4) for up to 8 h. The buffer was added with 10% MeCN. Stock solutions of each compound (10 mM) were freshly prepared in MeCN. Each compound was incubated at a final concentration of 1 μM in preheated buffer (37 °C). The sample solutions were divided into aliquots in glass vials (preheated at 37 °C) for each time point. The samples were maintained at 37 °C in the UPLC/MS autosampler during the study (no shaking). A reference solution of each compound (final concentration: 1 μM) in preheated MeCN was prepared from the stock solutions and maintained at 37 °C in the UPLC/MS autosampler during the study. For each time point, the samples were analyzed directly by LC/MS without any further sample preparation. The samples were analyzed by integrating the corresponding MRM peak areas. The relative compound concentration was calculated by dividing the peak area at each time point by the peak area at *t* = 0 min. The reference solution was analyzed at the beginning (*t* = 0 min) and at the end of the study (*t* = 8 h). The apparent half-life (*t*_1/2_) of the disappearance of the compound was calculated using the best fitting equation by GraphPad Prism (GraphPad Software, Inc., USA). The analyses were performed on a Waters ACQUITY UPLC/MS TQD system consisting of a triple quadrupole detector (TQD) MS equipped with an ESI interface and a photodiode array detector. The analyses were run on an ACQUITY UPLC BEH C18 1.7 μm 2.1 × 50 mm column with a VanGuard BEH C18 1.7 μm preolumn at 40 °C. For each compound, the appropriate mobile phase was chosen. ESI was applied in positive mode. Values are the mean of at least two independent experiments performed in two technical replicates.

#### In Vitro Plasma Stability Study

Freshly prepared 10 mM MeCN stock solution of test compound was diluted 50-fold with DMSO/H_2_O (1:1) and incubated at 37 °C for 2 h with mouse plasma added in 5% DMSO (preheated at 37 °C for 10 min). The final concentration was 2 μM. At each time point (0, 5, 15, 30, 60, and 120 min), 50 μL of incubation mixture was diluted with 200 μL of cold MeCN spiked with 200 nM internal standard followed by centrifugation at 3300 × *g* for 20 min. The supernatant was further diluted with H_2_O (1:1) for analysis. The concentration of test compound was quantified by LC/MS/MS on a Waters ACQUITY UPLC/MS TQD system consisting of a TQD MS equipped with an ESI interface. The analyses were run on an ACQUITY UPLC BEH C18 (50 × 2.1 mm ID, particle size 1.7 μm) with a VanGuard BEH C18 precolumn (5 × 2.1 mm ID, particle size 1.7 μm) at 40 °C. For each compound, the appropriate mobile phase was chosen. ESI was applied in positive mode. The response factors, calculated on the basis of the internal standard peak area, were plotted over time. When possible, response vs time profiles were fitted with Prism (GraphPad Software, Inc., USA) to estimate compound *t*_1/2_ in plasma. Values are the mean of at least two independent experiments performed in two technical replicates.

#### In Vitro Microsomal Stability Study

Freshly prepared 10 mM MeCN stock solution of test compound was preincubated at 37 °C for 15 min with mouse liver microsomes added in 0.1 M Tris HCl buffer (pH 7.4). The final concentration was 4.6 μM. After preincubation, the cofactors (NADPH, G6P, G6PDH, and MgCl_2_ predissolved in 0.1 M Tris HCl) were added to the incubation mixture, and the incubation was continued at 37 °C for 1 h. At each time point (0, 5, 15, 30, and 60 min), 30 μL of incubation mixture was diluted with 200 μL of cold MeCN spiked with 200 nM internal standard followed by centrifugation at 3300 × *g* for 15 min. The supernatant was further diluted with H_2_O (1:1) for analysis. The concentration of the test compound was quantified by LC/MS/MS on a Waters ACQUITY UPLC/MS TQD system consisting of a TQD MS equipped with an ESI interface. The analyses were run on an ACQUITY UPLC BEH C18 (50 × 2.1 mm ID, particle size 1.7 μm) with a VanGuard BEH C18 precolumn (5 × 2.1 mm ID, particle size 1.7 μm) at 40 °C. For each compound, the appropriate mobile phase was chosen. ESI was applied in positive mode. The percentage of test compound remaining at each time point relative to *t* = 0 was calculated. *t*_1/2_ was determined by a one-phase decay equation using a nonlinear regression of compound concentration vs time. Values are the mean of at least two independent experiments performed in two technical replicates.

### In Vitro Mouse Plasma and Mouse Brain Tissue Protein Binding

Studies were performed by the DMPK Group at Shanghai ChemPartner Co., Ltd., using the equilibrium dialysis method. Values are the mean of two technical replicates.

### Animal Models

#### In Vivo Pharmacokinetic Study

Male CD1 mice (22–24 g, 6 weeks old, SLAC Laboratory Animal Co. Ltd.) were group-housed in ventilated cages and had free access to water and food. They were maintained under a 24 h light/dark cycle at controlled temperature and relative humidity. All efforts were made to minimize animal suffering and to use the minimal number of animals required to produce reliable results. All procedures were performed in accordance with the Ethical Guidelines on the Protection of Animals Used for Scientific Purposes at the DMPK Group at Shanghai ChemPartner Co., Ltd. **22m** was administrated intravenously (i.v.) at 3 mg kg^–1^ (vehicle: 100% saline at 0.6 mg mL^–1^) via tail vein injection (*N* = 18) and via oral administration (p.o.) at 10 mg kg^–1^ (vehicle: 100% saline at 2.0 mg mL^–1^) by oral gavage (*N* = 18). *Sample collection*. Samples were collected at 0.25, 0.5, 1, 4, 8, and 24 h. Animals were sacrificed 24 h after **22m** administration; plasma, brain, and CSF samples were collected and stored at −80 °C. *Blood collection*: The animal was restrained manually, and approximately 150 μL of blood/time point was collected into the K_2_EDTA tube via retro orbital puncture under anesthesia with isoflurane. The blood sample was put on ice and centrifuged to obtain the plasma sample (2000 × *g*, 5 min under 4 °C) within 15 min and then acidified following 100 μL of plasma + 1.0 μL of formic acid. An aliquot of 20 μL sample (pretreatment with 1% formic acid) was added with 200 μL of IS (propranolol, 40 ng mL^–1^) in MeCN. The mixture was vortexed for 5 min and centrifuged at 6000 rpm for 10 min. The 0.5 μL mixture was injected into LC/MS/MS. *Brain collection:* Brain was removed and immediately homogenized immediately for 2 min with three volumes (v/w) of homogenizing solution (PBS:formic acid = 100:1), and then the solution was stored in tubes under −70 °C until analysis. An aliquot of 20 μL sample was added with 200 μL of MeCN, which contains IS (propranolol, 40 ng mL^–1^) for protein precipitation. The mixture was vortexed for 5 min and centrifuged at 6000 rpm for 10 min. The 0.5 μL mixture was injected into LC/MS/MS. *CSF collection*: A midline incision was made on the neck. The muscle under the skin was cut to expose the cisterna magna. The CSF was collected by capillary. An aliquot of 3 μL sample was added with 90 μL of IS (propranolol, 40 ng mL^–1^) in MeCN:H_2_O = 2:1 (added 1% formic acid). The mixture was vortexed for 5 min and centrifuged at 6000 rpm for 10 min. The 1.5 μL mixture was injected into LC/MS/MS. **22m** sample levels were monitored on an LC/MS/MS-19 (API5500, Qtriple) system, using the calibration curve and propanol as internal standard. Chromatography was carried out on a Waters BEH C18 column (2.1 × 50 mm, 1.7 μm) at 60 °C, setting a flow rate of 0.60 mL min^–1^. Mobile phases were as follows: A = H_2_O/0.025% formic acid/1 mM NH_4_OAc and B = MeOH/0.025% formic acid/1 mM NH_4_OAc. After the initial 0.20 min at 10% of mobile phase B, the percentage of mobile phase B increased at 70% at 0.50 min, reaching steadily 90% in the range of 0.80–1.30 min. Then the system returned to the initial conditions in a single step until 1.80 min. The following parent (*m*/*z*)/daughter (*m*/*z*) transitions were monitored: **22m**: *m*/*z* = 332.20/333.20 Da; propanol (IS): *m*/*z*: 260.30/116.10 Da.

#### Maximum Tolerated Dose (MTD) Study

An MTD study was conducted on male C57BL/6 mice (15–19 g, 5 weeks old, SLAC Laboratory Animal Co. Ltd.). Animals were injected via intraperitoneal (i.p.) injection with single administration at 20 mg kg^–1^ (*N* = 18, vehicle: 100% saline at 0.6 mg mL^–1^) and multiple administrations for a duration of 4 days at 20 (*N* = 18, day 1), 40 (*N* = 18, day 2), 80 (*N* = 18, day 3), and 120 mg kg^–1^ (*N* = 18, day 4). Clinical observations/samples of plasma, CSF, and brain were collected at 0.25, 0.5, 1, 4, 8, and 24 h. All procedures were performed in accordance with the Ethical Guidelines on the Protection of Animals Used for Scientific Purposes at the DMPK Group at Shanghai ChemPartner Co., Ltd.

#### In Vivo Mouse Model Studies

4L;C* mice (C57BL/6 J/129SvEV) were randomly assigned to three treatment groups (*N* = 4–8 with mixed males and females) and dosed once a day i.p. with 90 or 30 mg kg^–1^ of **22m** or vehicle. Treatment started at 5 days of age for a duration of 14 days. The application volume was set to 10 μL per gram of body weight, and dosage was adjusted accordingly. Animals of all groups were sacrificed 1 h after the last dose, and the brain tissues and plasma were collected. The left brain containing cortex, cerebella, thalamus, and brainstem was analyzed for SphL levels by MS. Data were analyzed using the Student *t* test. The right brain containing cortex, cerebella, thalamus, and brainstem and plasma were analyzed for drug levels of **22m**. All mice were housed under pathogen-free conditions in the animal facility, and animal experiment was performed according to the IACUC approved protocol (2018-0056) at Cincinnati Children’s Hospital Research Foundation. Wild Type (WT) (GALC+/+) and Twitcher (Twi) (GALC–/−) mice were genotyped by PCR as previously described.^58^ Twi mice were randomly assigned to three treatment groups (*N* = 3 males + *N* = 3 females for each group) and dosed once a day i.p. with 90 or 30 mg kg^–1^**22m** or vehicle for a duration of 20 days. Treatment started at 10 days of age. The application volume was set to 10 μL per gram of body weight, and dosage was adjusted accordingly. Two groups (*N* = 3 males + *N* = 3 females for each group) of WT controls treated with vehicle or high dose (90 mg kg^–1^) of **22m** were also included in the study. Animals of all groups were sacrificed 1 h after the last dose, and the brain tissues and plasma were collected. The left brain containing cortex, cerebella, thalamus, and brainstem was analyzed for SphL levels by MS. Data were analyzed using the Student *t* test. The right brain containing cortex, cerebella, thalamus, and brainstem and plasma were analyzed for drug levels of **22m**. All animal work in this study was performed in accordance with approved animal protocols from the Animal Care and Use Committee at the University of Illinois at Chicago.

## Supporting Information Available

*V*_max_ and *K*_M_ determinations; concentration–response curve of **22m** in primary fibroblast cells of Krabbe’s patients; ^1^H NMR and ^13^C NMR spectra of the final compounds; retention times and UPLC analytical methods of the final compounds; UPLC traces of the final compounds (PDF)
A csv file containing molecular formula strings (CSV)

## Abbreviations Used

AC: acid ceramidase
Cer: ceramide
GALC: β-galactosyl-ceramidase
GalCer: galactosylceramide
GalSph: galactosylsphingosine
GD: Gaucher’s disease
GCase: β-glucocerebrosidase
GluCer: glucosylceramide
GluSph: glucosylsphingosine
LSDs: lysosomal storage diseases
KD: Krabbe’s disease
SphLs: sphingolipids
Sph1P: sphingosine 1-phosphate
Twi: Twitcher
AcOH: glacial acetic acid
MeCN: acetonitrile
NH_4_OAc: ammonium acetate
NH_4_Cl: ammonium chloride
*n*-BuLi: *n*-butyllithium
CDI: 1′-carbonyldiimidazole
CHCl_3_: chloroform
Cy: cyclohexane
Celite: diatomaceous earth
Et_2_O: diethyl ether
DIPEA: *N*,*N*-diisopropylethylamine
(Pd(dppf)Cl_2_): [1,1′-bis(diphenylphosphino)ferrocene]dichloropalladium(II)
EtOH: ethanol
EtOAc: ethyl acetate
equiv.: equivalent
HCl: hydrochloric acid
LiCl: lithium chloride
MeOH: methanol
MeMgBr: methylmagnesium bromide
Pd/C: palladium on carbon
[B_2_(pin)_2_]: bis(pinacolato)diboron
KOAc: potassium acetate
K_2_CO_3_: potassium carbonate
K_3_PO_4_: potassium phosphate tribasic
*R_t_*: retention time
SiO_2_: silica gel
NaOAc: sodium acetate
NaHCO_3_: sodium bicarbonate
Na_2_CO_3_: sodium carbonate
Na_2_SO_4_: sodium sulfate
NaBH(OAc)_3_: sodium triacetoxyborohydride
Pd(PPh_3_)_4_: tetrakis(triphenylphosphine)palladium(0)
Dess–Martin periodinane: 1,1,1-tris(acetyloxy)-1,1-dihydro-1,2-benziodoxol-3-(1*H*)-one
Et_3_N: trimethylamine
Boc_2_O: di-*tert*-butyl dicarbonate
TZD: 2,4-thiazolidinedione
*p*-TsOH: *p*-toluenesulfonic acid monohydrate
H_2_O: water

## References

[ref1] Gault C. R.; Obeid L. M.; Hannun Y. A. An overview of sphingolipid metabolism: from synthesis to breakdown. Adv. Exp. Med. Biol. 2010, 688, 1–23. 10.1007/978-1-4419-6741-1_1. 20919643 PMC3069696

[ref2] Hannun Y. A.; Obeid L. M. Sphingolipids and their metabolism in physiology and disease. Nat. Rev. Mol. Cell Biol. 2018, 19, 175–191. 10.1038/nrm.2017.107. 29165427 PMC5902181

[ref3] Alberts B.; Johnson A.; Lewis J.; Raff M.; Roberts K.; Walter P. In Molecular Biology of the Cell; 5th ed. th ed.; Garland Science: New York, USA, Ed. 2008, 779–787.

[ref4] Saftig P.; Klumperman J. Lysosome biogenesis and lysosomal membrane proteins: trafficking meets function. Nat. Rev. Mol. Cell Biol. 2009, 10, 623–635. 10.1038/nrm2745. 19672277

[ref5] Bernardo K.; Hurwitz R.; Zenk T.; Desnick R. J.; Ferlinz K.; Schuchman E. H.; Sandhoff K. Purification, characterization, and biosynthesis of human acid ceramidase. J. Biol. Chem. 1995, 270, 11098–11102. 10.1074/jbc.270.19.11098. 7744740

[ref6] Mao C.; Obeid L. M. Ceramidases: regulators of cellular responses mediated by ceramide, sphingosine, and sphingosine-1-phosphate. Biochim. Biophys. Acta, Mol. Cell Biol. Lipids 2008, 1781, 424–434. 10.1016/j.bbalip.2008.06.002. PMC2614331 18619555

[ref7] Coant N.; Sakamoto W.; Mao C.; Hannun Y. A. Ceramidases, roles in sphingolipid metabolism and in health and disease. Adv. Biol. Regul. 2017, 63, 122–131. 10.1016/j.jbior.2016.10.002. 27771292 PMC5330250

[ref8] Hannun Y. A. Functions of ceramide in coordinating cellular responses to stress. Science 1996, 274, 1855–1859. 10.1126/science.274.5294.1855. 8943189

[ref9] Ballou L. R.; Laulederkind S. J. F.; Rosloniec E. F.; Raghow R. Ceramide signalling and the immune response. Biochim. Biophys. Acta 1996, 1301, 273–287. 10.1016/0005-2760(96)00004-5. 8664339

[ref10] Ruvolo P. P. Intracellular signal transduction pathways activated by ceramide and its metabolites. Pharmacol. Res. 2003, 47, 383–392. 10.1016/S1043-6618(03)00050-1. 12676512

[ref11] Huang X.; Withers B. R.; Dickson R. C. Sphingolipids and lifespan regulation. Biochim. Biophys. Acta, Mol. Cell Biol. Lipids 2014, 1841, 657–664. 10.1016/j.bbalip.2013.08.006. PMC3925463 23954556

[ref12] Pettus B. J.; Chalfant C. E.; Hannun Y. A. Ceramide in apoptosis: an overview and current perspectives. Biochim. Biophys. Acta, Mol. Cell Biol. Lipids 2002, 1585, 114–125. 10.1016/S1388-1981(02)00331-1. 12531544

[ref13] Morales A.; Lee H.; Goñi F. M.; Kolesnick R.; Fernandez-Checa J. C. Sphingolipids and cell death. Apoptosis 2007, 12, 923–939. 10.1007/s10495-007-0721-0. 17294080

[ref14] Zhang H.; Desai N. N.; Olivera A.; Seki T.; Brooker G.; Spiegel S. Sphingosine-1-phosphate, a novel lipid, involved in cellular proliferation. J. Cell Biol. 1991, 114, 155–167. 10.1083/jcb.114.1.155. 2050740 PMC2289065

[ref15] Payne S. G.; Milstien S.; Spiegel S. Sphingosine-1-phosphate: dual messenger functions. FEBS Lett. 2002, 531, 54–57. 10.1016/S0014-5793(02)03480-4. 12401202

[ref16] Spiegel S.; Milstien S. Sphingosine-1-phosphate: an enigmatic signalling lipid. Nat. Rev. Mol. Cell Biol. 2003, 4, 397–407. 10.1038/nrm1103. 12728273

[ref17] Takabe K.; Spiegel S. Export of sphingosine-1-phosphate and cancer progression. J. Lipid Res. 2014, 55, 1839–1846. 10.1194/jlr.R046656. 24474820 PMC4617347

[ref18] Realini N.; Palese F.; Pizzirani D.; Pontis S.; Basit A.; Bach A.; Ganesan A.; Piomelli D. Acid ceramidase in melanoma: expression, localization, and effects of pharmacological inhibition. J. Biol. Chem. 2016, 291, 2422–2434. 10.1074/jbc.M115.666909. 26553872 PMC4732224

[ref19] Schuchman E. H. Acid ceramidase and the treatment of ceramide diseases: The expanding role of enzyme replacement therapy. Biochim. Biophys. Acta, Mol. Basis Dis. 2016, 1862, 1459–1471. 10.1016/j.bbadis.2016.05.001. 27155573

[ref20] Iwabuchi K.; Nakayama H.; Oizumi A.; Suga Y.; Ogawa H.; Takamori K. Role of ceramide from glycosphingolipids and its metabolites in immunological and inflammatory responses in humans. Mediators Inflammation 2015, 2015, 1–10. 10.1155/2015/120748. PMC4644562 26609196

[ref21] Gieselmann V. Lysosomal storage diseases. Biochim. Biophys. Acta, Mol. Basis Dis. 1995, 1270, 103–136. 10.1016/0925-4439(94)00075-2. 7727535

[ref22] Vanier M.-T. Disorders of Sphingolipid Metabolism. In Inborn Metabolic Diseases: Diagnosis and Treatment; Fernandes J.; Saudubray J.-M.; van den Berghe G.; Walter J. H., Eds. Springer: Berlin, Heidelberg, 2006, 479–494.

[ref23] Ballabio A.; Gieselmann V. Lysosomal disorders: from storage to cellular damage. Biochim. Biophys. Acta, Mol. Cell Res. 2009, 1793, 684–696. 10.1016/j.bbamcr.2008.12.001. 19111581

[ref24] Cox T. M.; Cachón-González M. B. The cellular pathology of lysosomal diseases. J. Pathol. 2012, 226, 241–254. 10.1002/path.3021. 21990005

[ref25] Brady R. O.; Kanfer J.; Shapiro D. The metabolism of glucocerebrosides. I. Purification and properties of a glucocerebroside-cleaving enzyme from spleen tissue. J. Biol. Chem. 1965, 240, 39–43. 14253443

[ref26] Sorbera L. A.; Sundaravinayagam D.; Dulsat C.; Rosa E. Therapeutic targets for Gaucher′s disease. Drugs Future 2009, 34, 1001 10.1358/dof.2009.034.12.1444443.

[ref27] Stirnemann J.; Belmatoug N.; Camou F.; Serratrice C.; Froissart R.; Caillaud C.; Levade T.; Astudillo L.; Serratrice J.; Brassier A.; Rose C.; de Villemeur T. B.; Berger M. G. A review of Gaucher disease pathophysiology, clinical presentation and treatments. Int. J. Mol. Sci. 2017, 18, 441–471. 10.3390/ijms18020441. PMC5343975 28218669

[ref28] Won J.-S.; Singh A. K.; Singh I. Biochemical, cell biological, pathological, and therapeutic aspects of Krabbe’s disease. J. Neurosci. Res. 2016, 94, 990–1006. 10.1002/jnr.23873. 27638584 PMC5812347

[ref29] Nilsson O.; Svennerholm L. Accumulation of glucosylceramide and glucosylsphingosine (psychosine) in cerebrum and cerebellum in infantile and juvenile Gaucher disease. J. Neurochem. 1982, 39, 709–718. 10.1111/j.1471-4159.1982.tb07950.x. 7097276

[ref30] Sidransky E. Gaucher disease: complexity in a ″simple″ disorder. Mol. Genet. Metab. 2004, 83, 6–15. 10.1016/j.ymgme.2004.08.015. 15464415

[ref31] Ferraz M. J.; Marques A. R. A.; Appelman M. D.; Verhoek M.; Strijland A.; Mirzaian M.; Scheij S.; Ouairy C. M.; Lahav D.; Wisse P.; Overkleeft H. S.; Boot R. G.; Aerts J. M. Lysosomal glycosphingolipid catabolism by acid ceramidase: formation of glycosphingoid bases during deficiency of glycosidases. FEBS Lett. 2016, 590, 716–725. 10.1002/1873-3468.12104. 26898341

[ref32] Murugesan V.; Chuang W.-L.; Liu J.; Lischuk A.; Kacena K.; Lin H.; Pastores G. M.; Yang R.; Keutzer J.; Zhang K.; Mistry P. K. Glucosylsphingosine is a key biomarker of Gaucher disease. Am. J. Hematol. 2016, 91, 1082–1089. 10.1002/ajh.24491. 27441734 PMC5234703

[ref33] Suzuki K.; Suzuki K. The Twitcher Mouse: A model for Krabbe disease and for experimental therapies. Brain Pathol. 1995, 5, 249–258. 10.1111/j.1750-3639.1995.tb00601.x. 8520724

[ref34] Li Y.; Xu Y.; Benitez B. A.; Nagree M. S.; Dearborn J. T.; Jiang X.; Guzman M. A.; Woloszynek J. C.; Giaramita A.; Yip B. K.; Elsbernd J.; Babcock M. C.; Lo M.; Fowler S. C.; Wozniak D. F.; Vogler C. A.; Medin J. A.; Crawford B. E.; Sands M. S. Genetic ablation of acid ceramidase in Krabbe disease confirms the psychosine hypothesis and identifies a new therapeutic target. Proc. Natl. Acad. Sci. U. S. A. 2019, 116, 20097–20103. 10.1073/pnas.1912108116. 31527255 PMC6778236

[ref35] Gebai A.; Gorelik A.; Li Z.; Illes K.; Nagar B. Structural basis for the activation of acid ceramidase. Nat. Commun. 2018, 9, 1621 10.1038/s41467-018-03844-2. 29692406 PMC5915598

[ref36] Realini N.; Solorzano C.; Pagliuca C.; Pizzirani D.; Armirotti A.; Luciani R.; Costi M. P.; Bandiera T.; Piomelli D. Discovery of highly potent acid ceramidase inhibitors with in vitro tumor chemosensitizing activity. Sci. Rep. 2013, 3, 1035 10.1038/srep01035. 23301156 PMC3539145

[ref37] Pizzirani D.; Pagliuca C.; Realini N.; Branduardi D.; Bottegoni G.; Mor M.; Bertozzi F.; Scarpelli R.; Piomelli D.; Bandiera T. Discovery of a new class of highly potent inhibitors of acid ceramidase: synthesis and structure-activity relationship (SAR). J. Med. Chem. 2013, 56, 3518–3530. 10.1021/jm301879g. 23614460

[ref38] Diamanti E.; Bottegoni G.; Goldoni L.; Realini N.; Pagliuca C.; Bertozzi F.; Piomelli D.; Pizzirani D. Pyrazole-based acid ceramidase inhibitors: design, synthesis, and structure-activity relationships. Synthesis 2016, 48, 2739–2756. 10.1055/s-0035-1561456.

[ref39] Pizzirani D.; Bach A.; Realini N.; Armirotti A.; Mengatto L.; Bauer I.; Girotto S.; Pagliuca C.; De Vivo M.; Summa M.; Ribeiro A.; Piomelli D. Benzoxazolone carboxamides: potent and systemically active inhibitors of intracellular acid ceramidase. Angew. Chem., Int. Ed. 2014, 54, 485–489. 10.1002/anie.201409042. PMC4502975 25395373

[ref40] Bach A.; Pizzirani D.; Realini N.; Vozella V.; Russo D.; Penna I.; Melzig L.; Scarpelli R.; Piomelli D. Benzoxazolone carboxamides as potent acid ceramidase inhibitors: synthesis and structure-activity relationship (SAR) studies. J. Med. Chem. 2015, 58, 9258–9272. 10.1021/acs.jmedchem.5b01188. 26560855

[ref41] Ortega J. A.; Arencibia J. M.; La Sala G.; Borgogno M.; Bauer I.; Bono L.; Braccia C.; Armirotti A.; Girotto S.; Ganesan A.; De Vivo M. Pharmacophore identification and scaffold exploration to discover novel, potent, and chemically stable inhibitors of acid ceramidase in melanoma cells. J. Med. Chem. 2017, 60, 5800–5815. 10.1021/acs.jmedchem.7b00472. 28603987

[ref42] Li X.; Russell R. K.; Spink J.; Ballentine S.; Teleha C.; Branum S.; Wells K.; Beauchamp D.; Patch R.; Huang H.; Player M.; Murray W. Process development for scale-up of a novel 3,5-substituted thiazolidine-2,4-dione compound as a potent inhibitor for estrogen-related receptor 1. Org. Process Res. Dev. 2014, 18, 321–330. 10.1021/op400325r.

[ref43] DeOrazio R. J.; Maeng J.-H.; Manning D. D.; Sherer B. A.; Scott I. L.; Nikam S. S. A simple strategy for the preparation of 6-substituted 3H-benzoxazol-2-ones and 3H-benzothiazol-2-ones. Synth. Commun. 2011, 41, 3551–3555. 10.1080/00397911.2010.519093.

[ref44] Rynearson K. D.; Charrette B.; Gabriel C.; Moreno J.; Boerneke M. A.; Dibrov S. M.; Hermann T. 2-Aminobenzoxazole ligands of the hepatitis C virus internal ribosome entry site. Bioorg. Med. Chem. Lett. 2014, 24, 3521–3525. 10.1016/j.bmcl.2014.05.088. 24930829 PMC4114401

[ref45] Toto P.; Gesquière J.-C.; Deprez B.; Willand N. Synthesis of N-(iodophenyl)-amides via an unprecedented Ullmann-Finkelstein tandem reaction. Tetrahedron Lett. 2006, 47, 1181–1186. 10.1016/j.tetlet.2005.12.022.

[ref46] Knöelker H.-J.; Braxmeier T.; Schlechtingen G. A novel method for the synthesis of isocyanates under mild conditions. Angew. Chem., Int. Ed. Engl. 1995, 34, 2497–2500. 10.1002/anie.199524971.

[ref47] Eckert H.; Forster B. Triphosgene, a crystalline phosgene substitute. Angew. Chem., Int. Ed. Engl. 1987, 26, 894–895. 10.1002/anie.198708941.

[ref48] Dementiev A.; Joachimiak A.; Nguyen H.; Gorelik A.; Illes K.; Shabani S.; Gelsomino M.; Ahn E.-Y. E.; Nagar B.; Doan N. Molecular mechanism of inhibition of acid ceramidase by carmofur. J. Med. Chem. 2019, 62, 987–992. 10.1021/acs.jmedchem.8b01723. 30525581 PMC6863082

[ref49] Cho S. M.; Kwon H. J. Acid ceramidase, an emerging target for anti-cancer and anti-angiogenesis. Arch. Pharmacal Res. 2019, 42, 232–243. 10.1007/s12272-019-01114-3. 30661200

[ref50] Ishikawa M.; Hashimoto Y. Improvement in aqueous solubility in small molecule drug discovery programs by disruption of molecular planarity and symmetry. J. Med. Chem. 2011, 54, 1539–1554. 10.1021/jm101356p. 21344906

[ref51] Meanwell N. A. Improving drug design: an update on recent applications of efficiency metrics, strategies for replacing problematic elements, and compounds in nontraditional drug space. Chem. Res. Toxicol. 2016, 29, 564–616. 10.1021/acs.chemrestox.6b00043. 26974882

[ref52] Copeland R. A. Evaluation of Enzyme Inhibitors in Drug Discovery: A Guide for Medicinal Chemists and Pharmacologists; Wiley-Interscience: Hoboken, New Jersey, USA, Ed. 2005, 48–248. 16350889

[ref53] Strelow J. D. W.; Iversen P. W.; Brooks H. B.; Radding J. A.; McGee J.; Weidner J., In Assay Guidance Manual; Sittampalam G. S. G. A.; Brimacombe K.; Arkin M.; Auld D.; Austin C. P.; Baell J.; Bejcek B.; Caaveiro J. M. M.; Chung T. D. Y.; Coussens N. P.; Dahlin J. L.; Devanaryan V.; Foley T. L.; Glicksman M.; Hall M. D.; Haas J. V.; Hoare S. R. J.; Inglese J.; Iversen P. W.; Kahl S. D.; Kales S. C.; Kirshner S.; Lal-Nag M.; Li Z.; McGee J.; McManus O.; Riss T.; Saradjian P.; Trask O. J.; Weidner J. R. Jr.; Wildey M. J.; Xia M.; Xu X., Ed. Bethesda (MD): Eli Lilly & Company and the National Center for Advancing Translational Sciences, 2004, 65–86. 22553861

[ref54] Gorelik A.; Gebai A.; Illes K.; Piomelli D.; Nagar B. Molecular mechanism of activation of the immunoregulatory amidase NAAA. Proc. Natl. Acad. Sci. U. S. A. 2018, 115, E10032–E10040. 10.1073/pnas.1811759115. 30301806 PMC6205464

[ref55] Ahn K.; McKinney M. K.; Cravatt B. F. Enzymatic pathways that regulate endocannabinoid signaling in the nervous system. Chem. Rev. 2008, 108, 1687–1707. 10.1021/cr0782067. 18429637 PMC3150828

[ref56] Blankman J. L.; Simon G. M.; Cravatt B. F. A Comprehensive profile of brain enzymes that hydrolyze the endocannabinoid 2-arachidonoylglycerol. Chem. Biol. 2007, 14, 1347–1356. 10.1016/j.chembiol.2007.11.006. 18096503 PMC2692834

[ref57] Di Marzo V. New approaches and challenges to targeting the endocannabinoid system. Nat. Rev. Drug Discovery 2018, 17, 623–639. 10.1038/nrd.2018.115. 30116049

[ref58] Sun Y.; Liou B.; Ran H.; Skelton M. R.; Williams M. T.; Vorhees C. V.; Kitatani K.; Hannun Y. A.; Witte D. P.; Xu Y.-H.; Grabowski G. A. Neuronopathic Gaucher disease in the mouse: viable combined selective saposin C deficiency and mutant glucocerebrosidase (V394L) mice with glucosylsphingosine and glucosylceramide accumulation and progressive neurological deficits. Hum. Mol. Genet. 2010, 19, 1088–1097. 10.1093/hmg/ddp580. 20047948 PMC2830832

