# Supplementary material for: Lead Optimization of Benzoxazolone Carboxamides as Orally Bioavailable and CNS Penetrant Acid Ceramidase Inhibitors
Source: J Med Chem. 2020 Mar 16;63(7):3634–64. doi: 10.1021/acs.jmedchem.9b02004 (PMC7997574; doi:10.1021/acs.jmedchem.9b02004)

Supporting information

Lead Optimization of Benzoxazolone Carboxamides as Orally Bioavailable and CNS Penetrant Acid Ceramidase Inhibitors.

Simona Di Martino,[a,¥#] *Piero Tardia,*[a,¥#] *Vincenzo Cilibrasi,*[a,¥] *Samantha Caputo,*[a,¥] *Marco Mazzonna,*[a,¥] *Debora Russo,*[a,§] *Ilaria Penna,*[a,§] *Natalia Realini,*[a,¥] *Natasha Margaroli,*[a,¥] *Marco Migliore,*[a,¥] *Daniela Pizzirani,*[a,¥] *Giuliana Ottonello,*[a,^] *Sine Mandrup Bertozzi,*[a,^] *Andrea Armirotti,*[a,^] *Duc Nguyen,*[c] *Ying Sun,*[d] *Ernesto R. Bongarzone,*[c] *Peter Lansbury,*[b] *Min Liu,*[b] *Renato Skerlj*[b,†*] *and Rita Scarpelli*[a,¥*]

Table of contents

Table S1. V_{max} and K_M determinations S2

Figure S1. Concentration- response curve of **22m** in primary fibroblast cells S3

^{1}H NMR and ^{13}C NMR spectra of the final compounds S4

Table S2. Retention times and UPLC analytical methods of the final compounds S48

UPLC traces of the final compounds S49

Table S1. V_{max} and K_M determinations.

		DMSO	**22m** (100 nM)	**22m** (400 nM)
*h*AC	V_{max} (μM/h)	15708	14577	11346
	K_M (μM)	4.913	5.258	5.475

Figure S1. Concentration- response curve of **22m** in primary fibroplast cells of Krabbe's patients (EC_{50} = 0.4±0.1 μM).

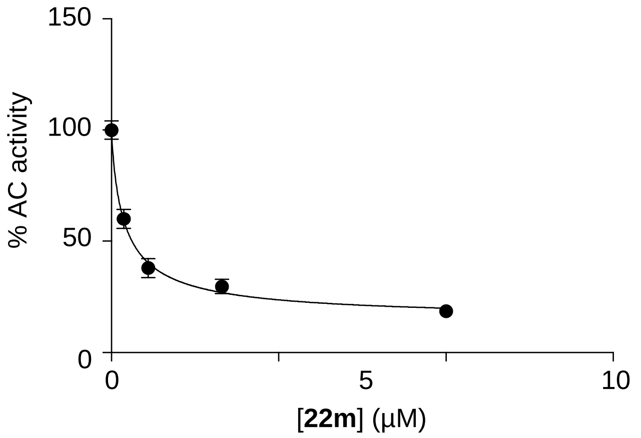

^1H NMR and ^{13}C NMR spectra of the final compounds.

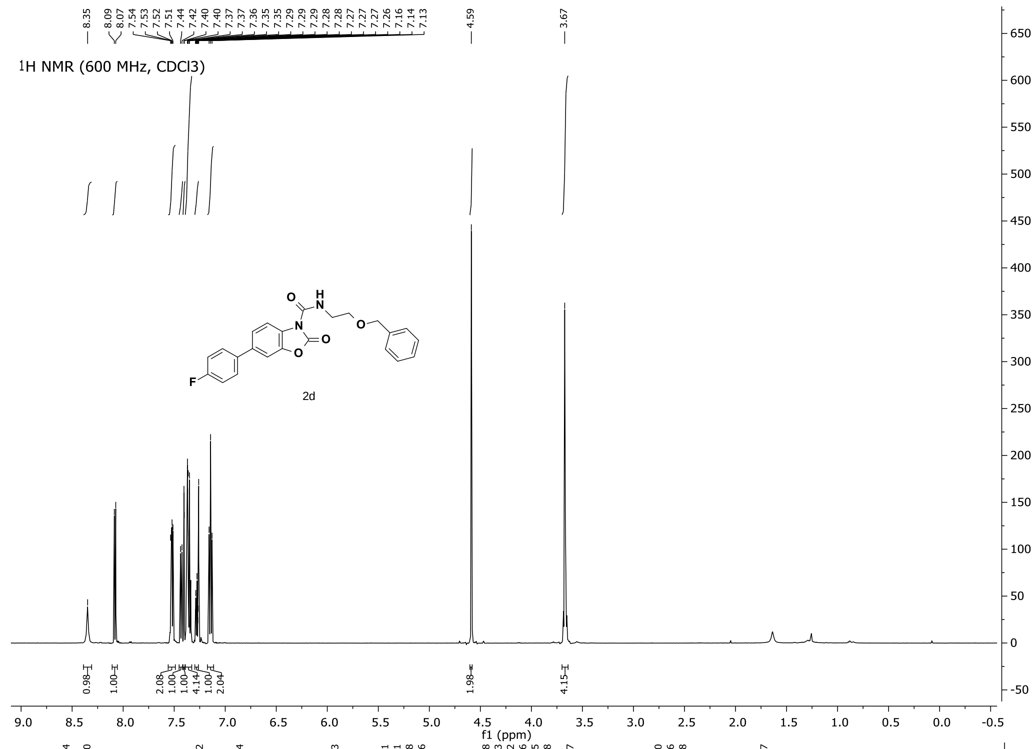

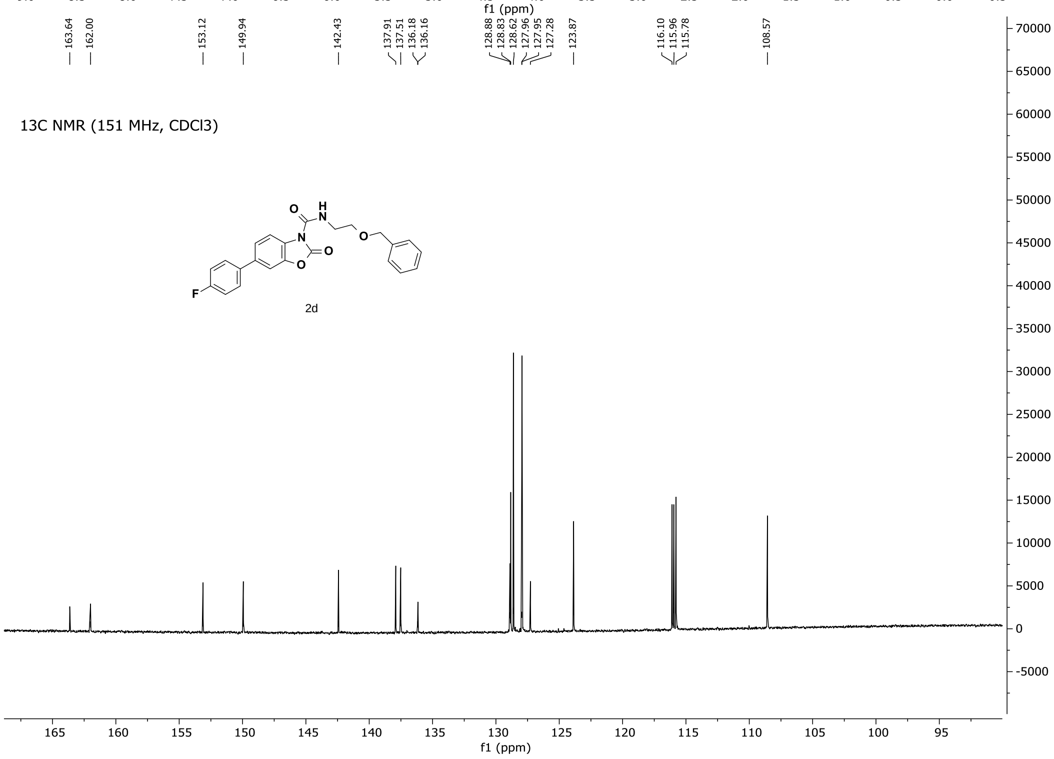

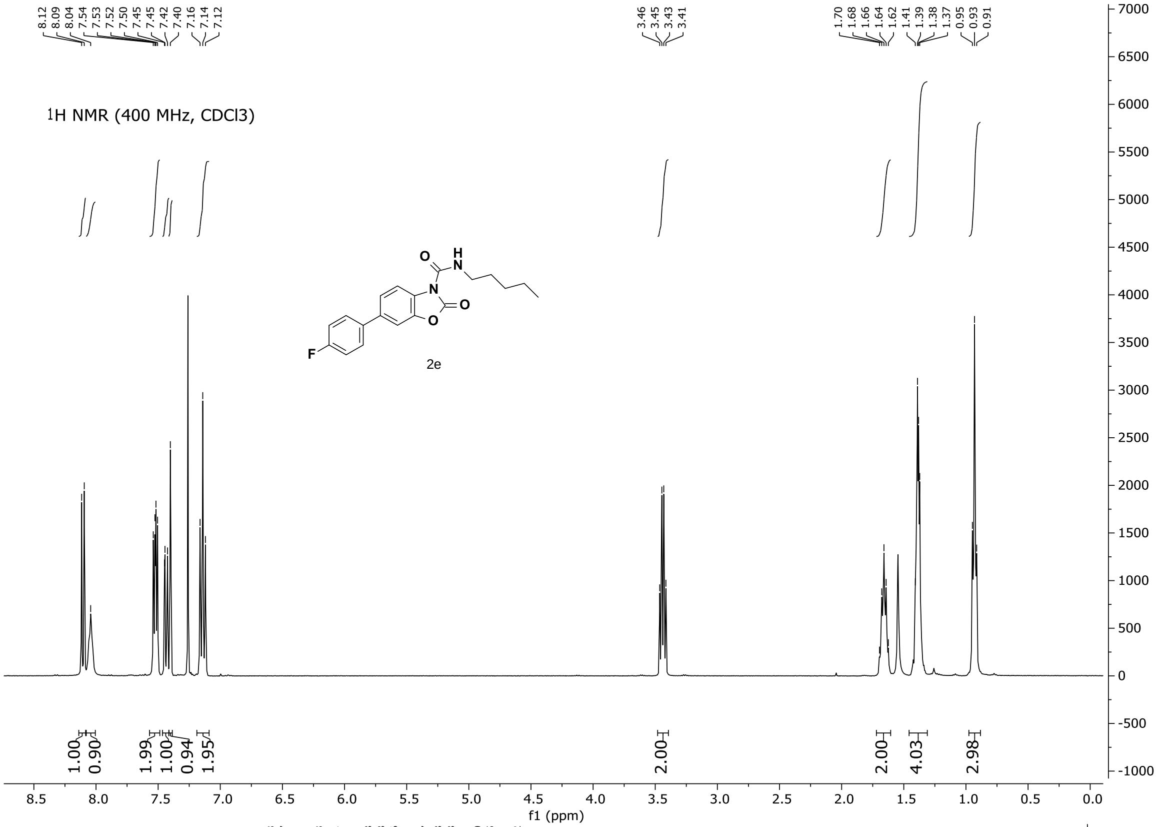
1H NMR (400 MHz, CDCl3)
8.12 8.09 8.04 7.54 7.53 7.52 7.50 7.45 7.45 7.42 7.40 7.16 7.14 7.12
3.46 3.45 3.43 3.41
1.70 1.68 1.66 1.64 1.62 1.41 1.39 1.38 1.37 0.95 0.93 0.91
1.00 0.90 1.99 1.00 0.94 1.95 2.00 2.00 4.03 2.98
2e
f1 (ppm)

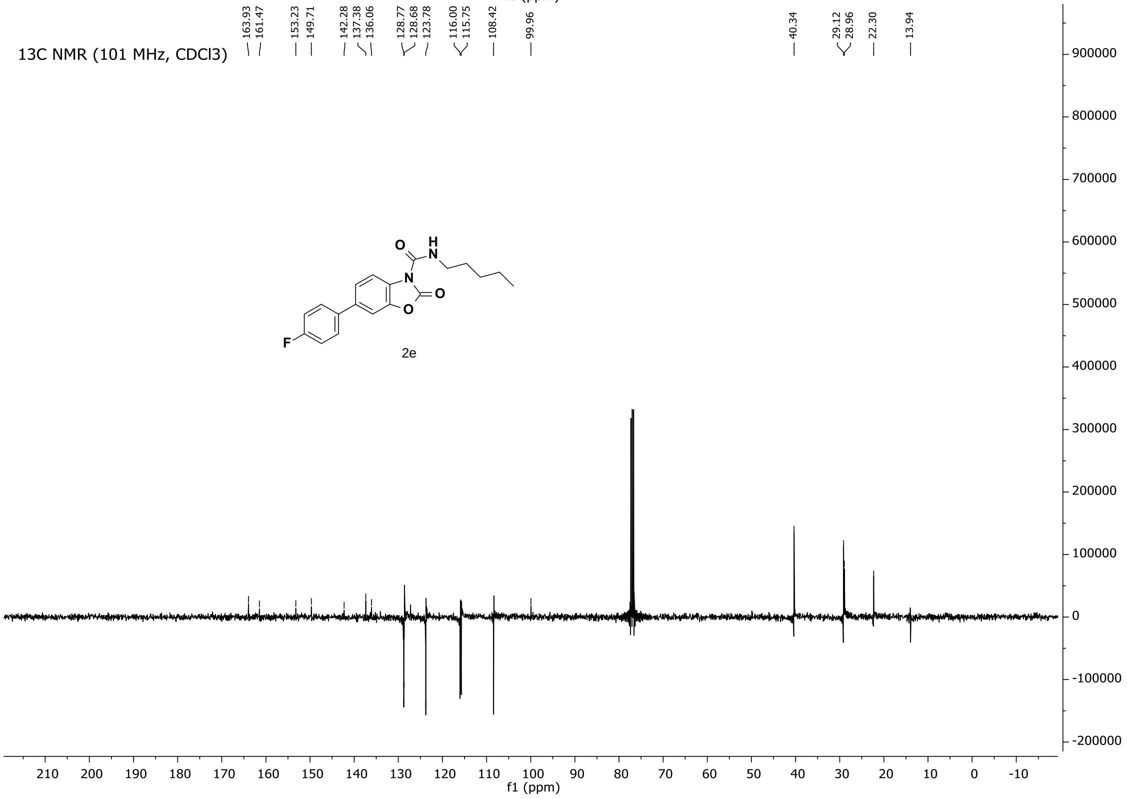
13C NMR (101 MHz, CDCl3)
163.93 161.47 153.23 149.71 142.28 137.38 136.06 128.77 128.68 123.78 116.00 115.75 108.42 99.96
40.34 29.12 28.96 22.30 13.94
2e
f1 (ppm)

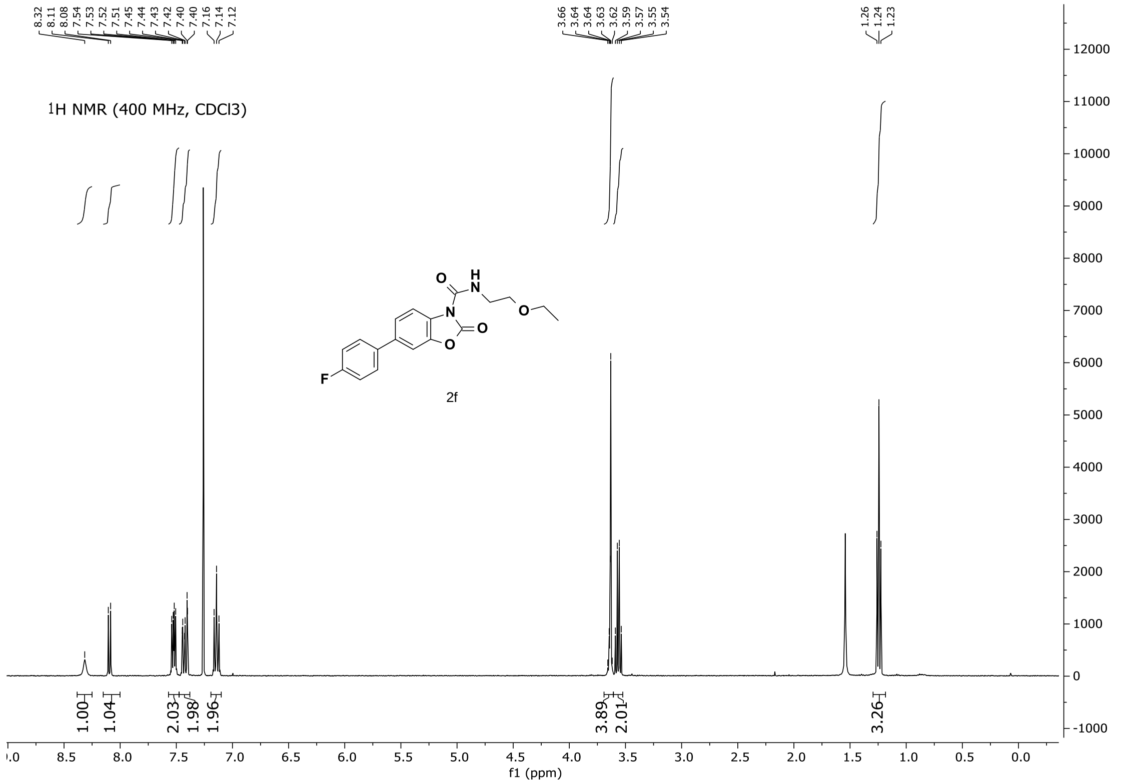
1H NMR (400 MHz, CDCl3)
2f
f1 (ppm)

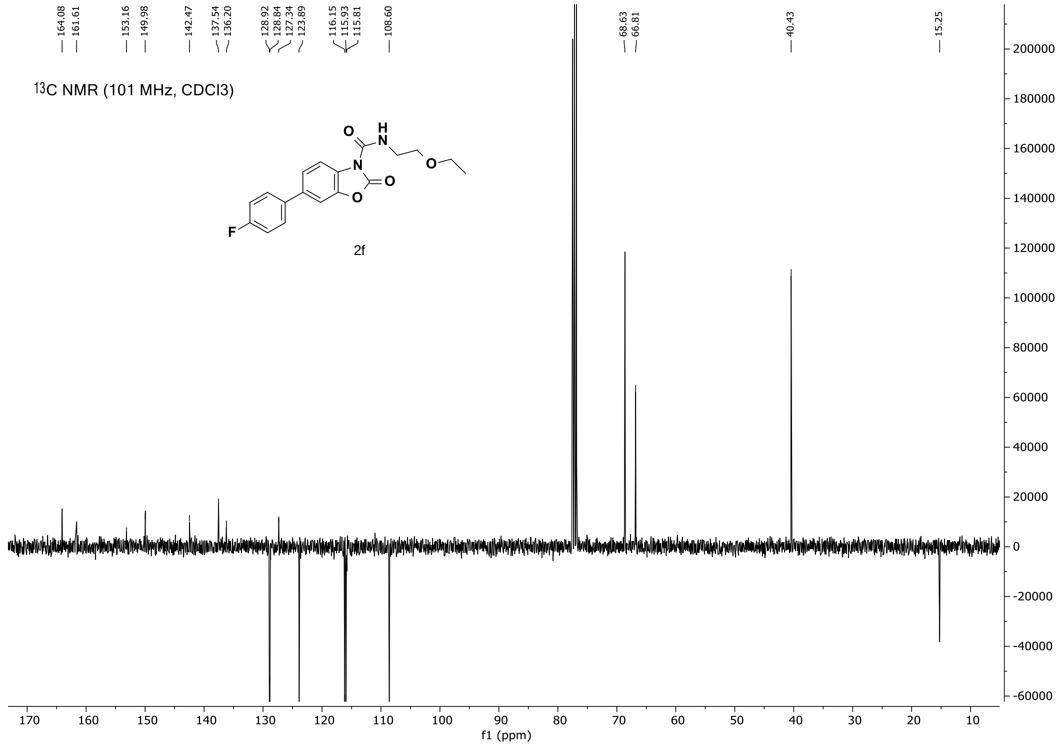
13C NMR (101 MHz, CDCl3)
2f
f1 (ppm)

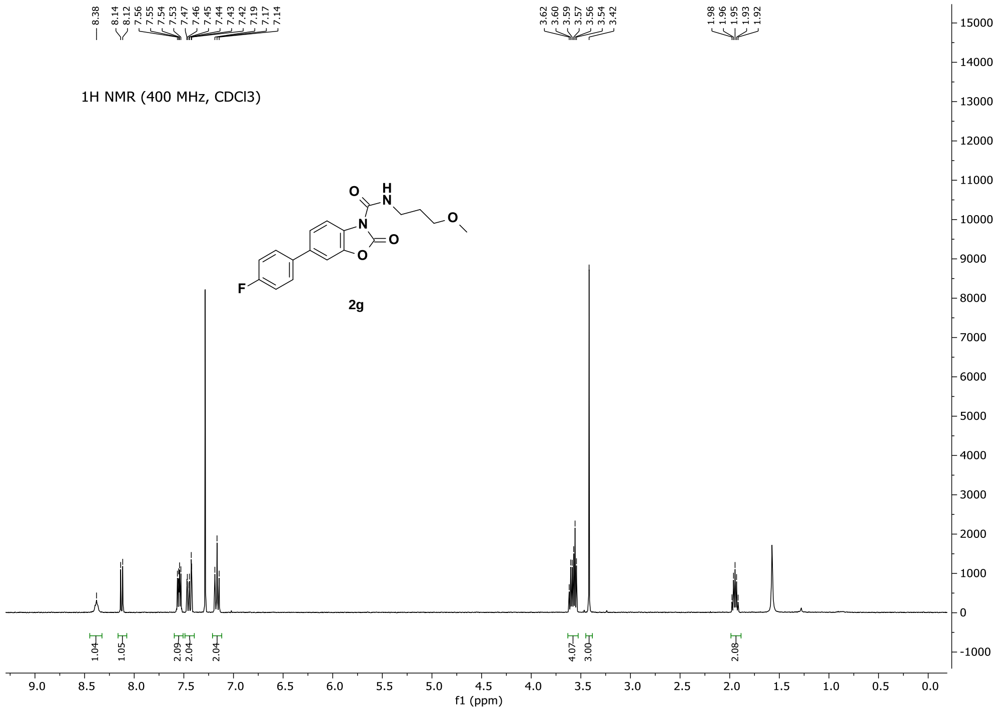
8.38
8.14
8.12
7.56
7.55
7.54
7.53
7.47
7.46
7.45
7.44
7.43
7.42
7.19
7.17
7.14
3.62
3.60
3.59
3.57
3.56
3.54
3.42
1.98
1.96
1.95
1.93
1.92
1H NMR (400 MHz, CDCl3)
O
H
N
N
O
O
O
F
2g
1.04
1.05
2.09
2.04
2.04
4.07
3.00
2.08
f1 (ppm)

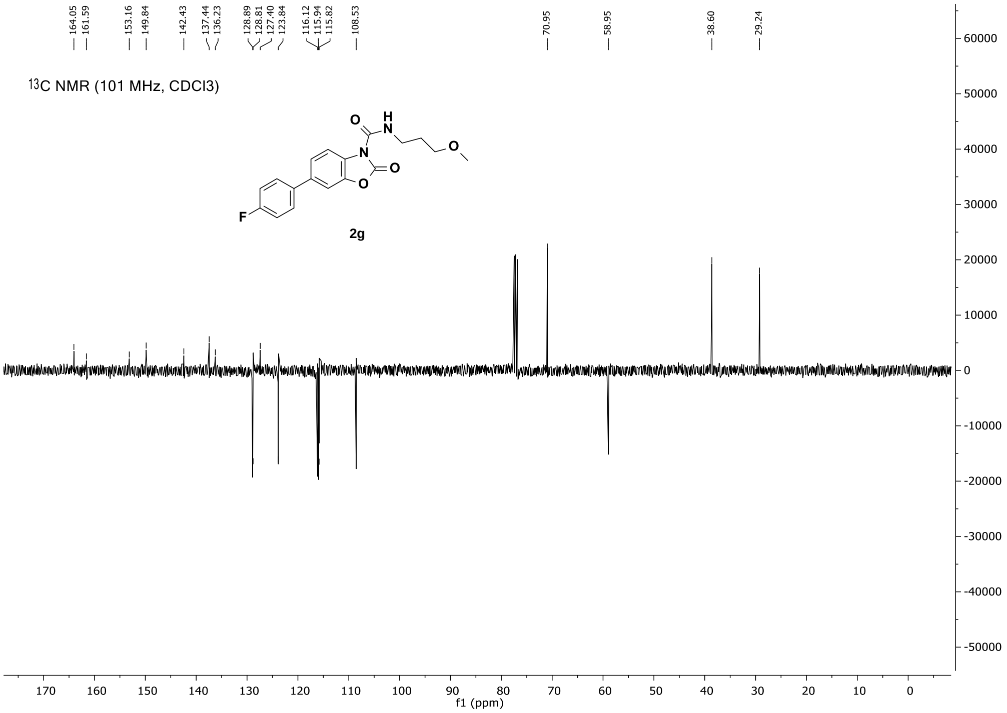
164.05
161.59
153.16
149.84
142.43
137.44
136.23
128.89
128.81
127.40
123.84
116.12
115.94
115.82
108.53
70.95
58.95
38.60
29.24
13C NMR (101 MHz, CDCl3)
O
H
N
N
O
O
O
F
2g
f1 (ppm)

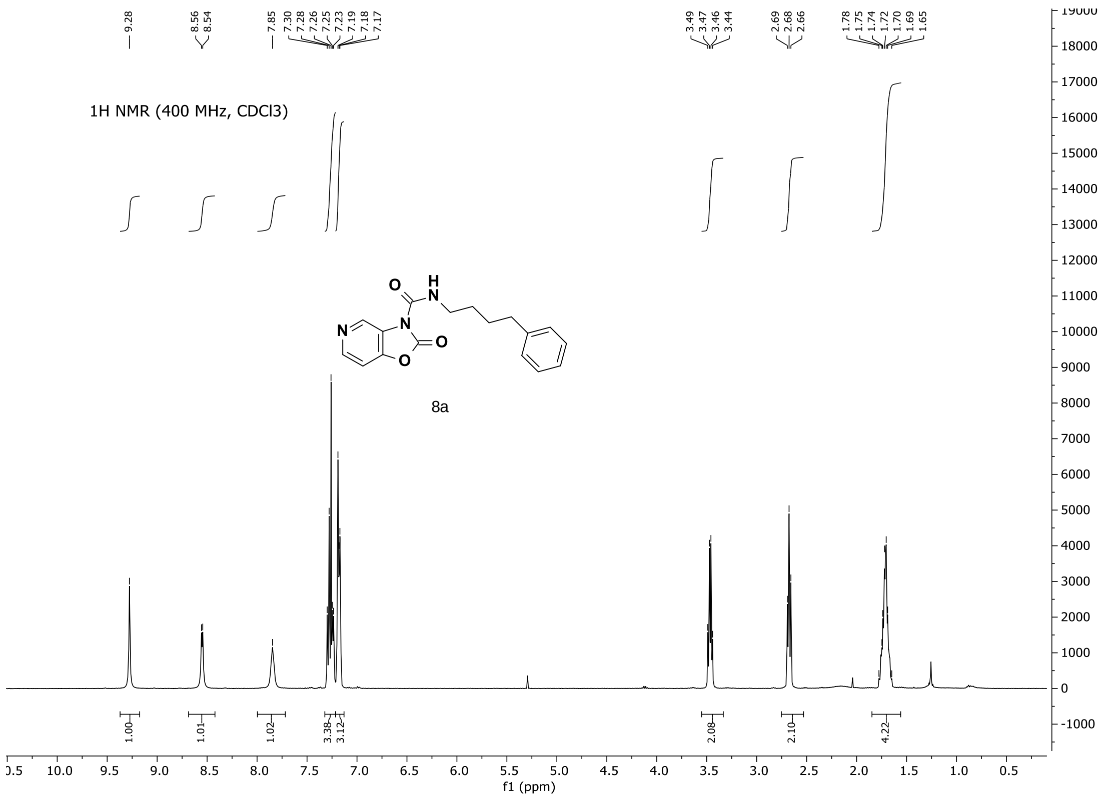
1H NMR (400 MHz, CDCl3)
8a
f1 (ppm)

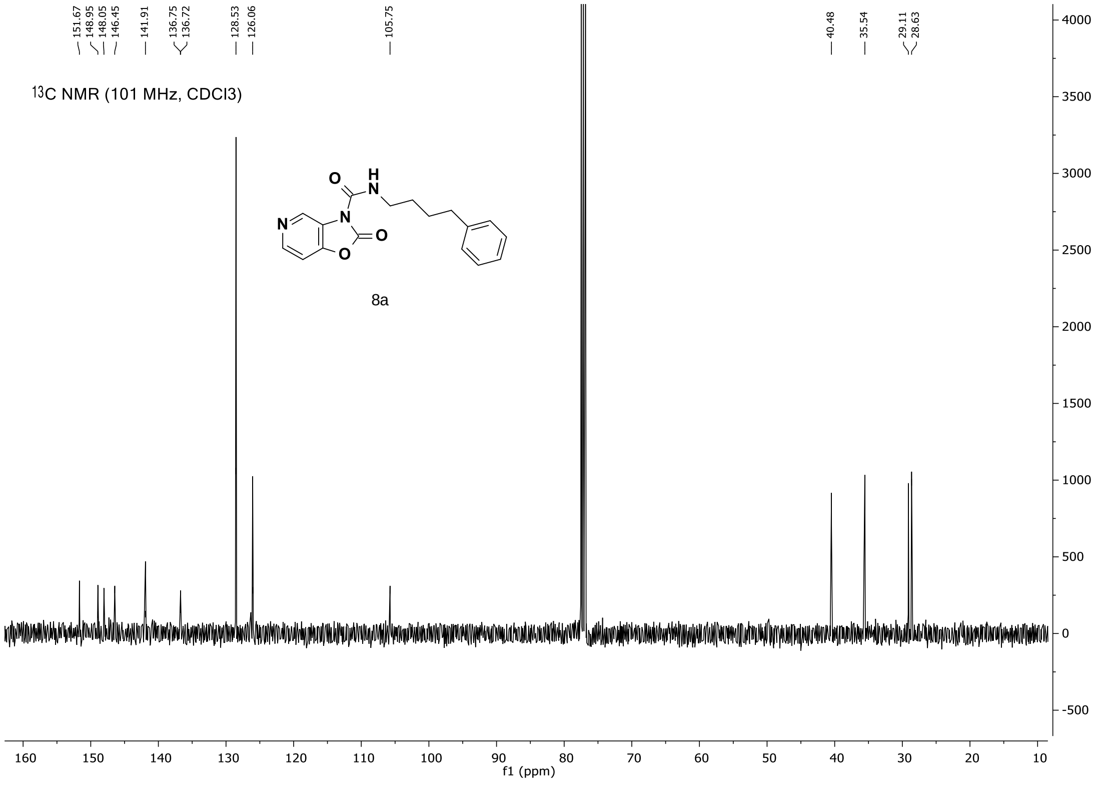
13C NMR (101 MHz, CDCl3)
8a
f1 (ppm)

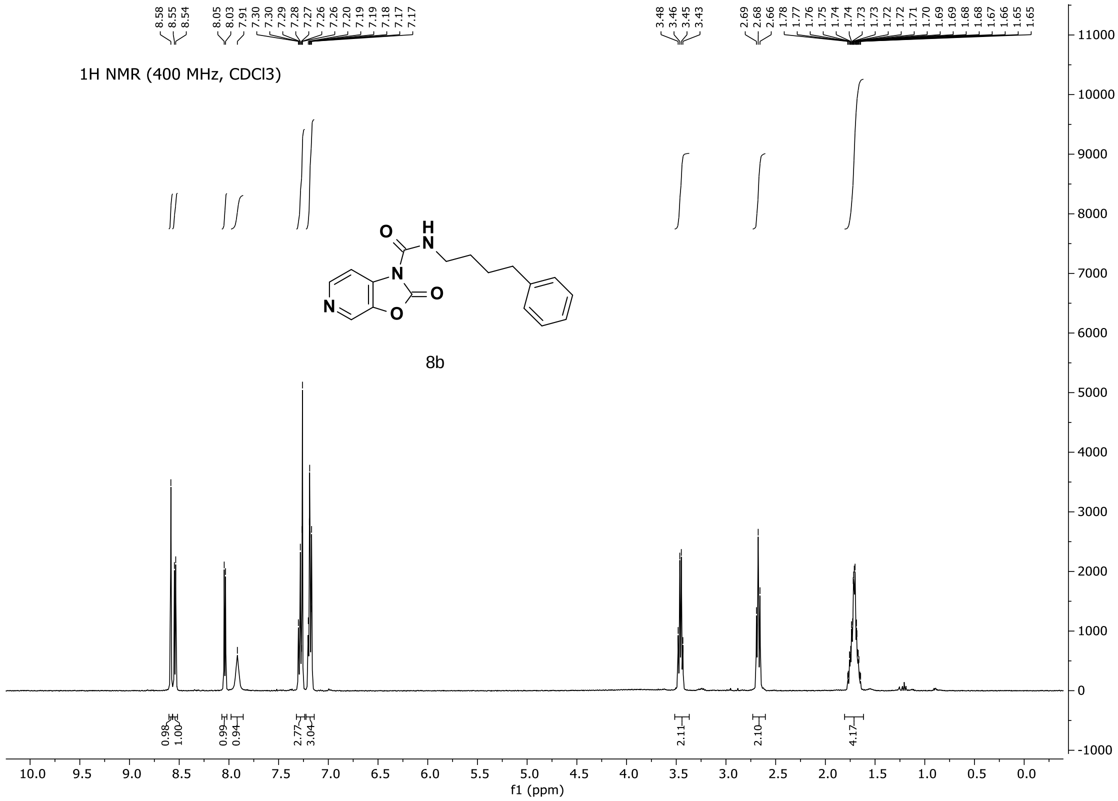
1H NMR (400 MHz, CDCl3)
8.58 8.55 8.54 8.05 8.03 7.91 7.30 7.29 7.28 7.27 7.26 7.26 7.20 7.19 7.19 7.18 7.17 7.17
3.48 3.46 3.45 3.43 2.69 2.68 2.66 1.78 1.77 1.76 1.75 1.74 1.74 1.73 1.73 1.72 1.72 1.71 1.70 1.69 1.69 1.68 1.68 1.67 1.66 1.65 1.65
8b
0.98 1.00 0.99 0.94 2.77 3.04 2.11 2.10 4.17
f1 (ppm)

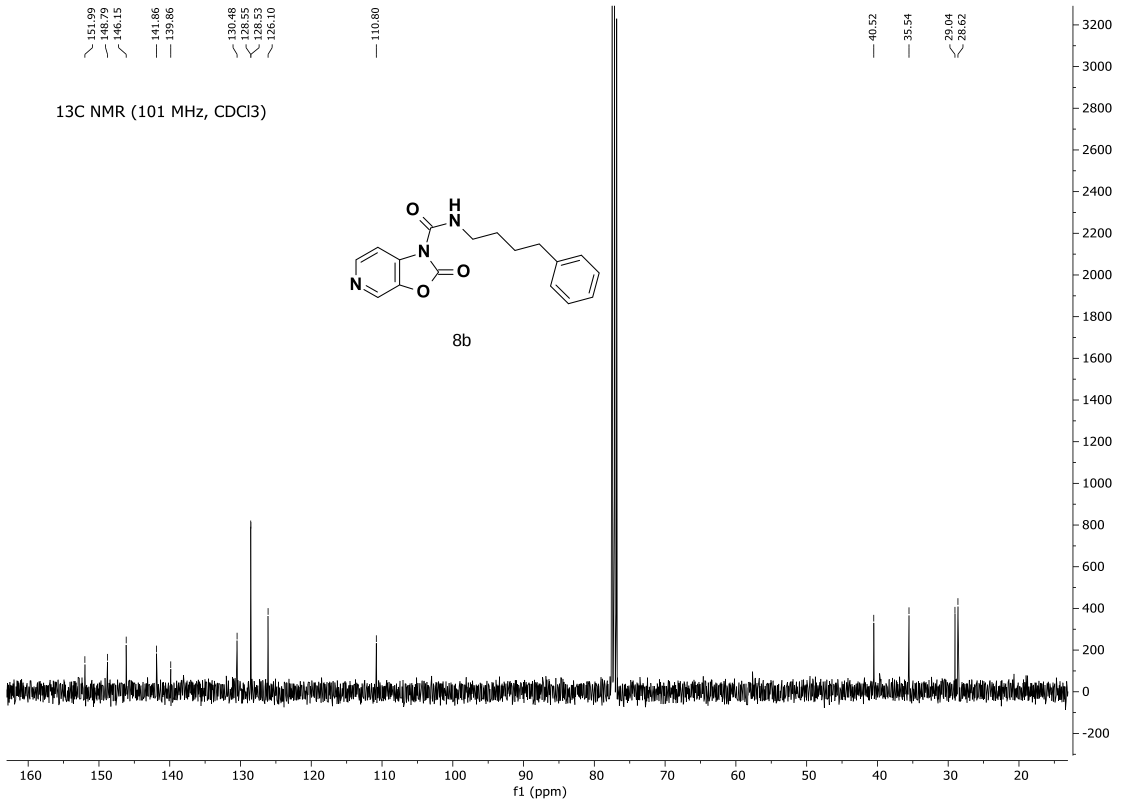
151.99 148.79 146.15 141.86 139.86 130.48 128.55 128.53 126.10 110.80 40.52 35.54 29.04 28.62
13C NMR (101 MHz, CDCl3)
8b
f1 (ppm)

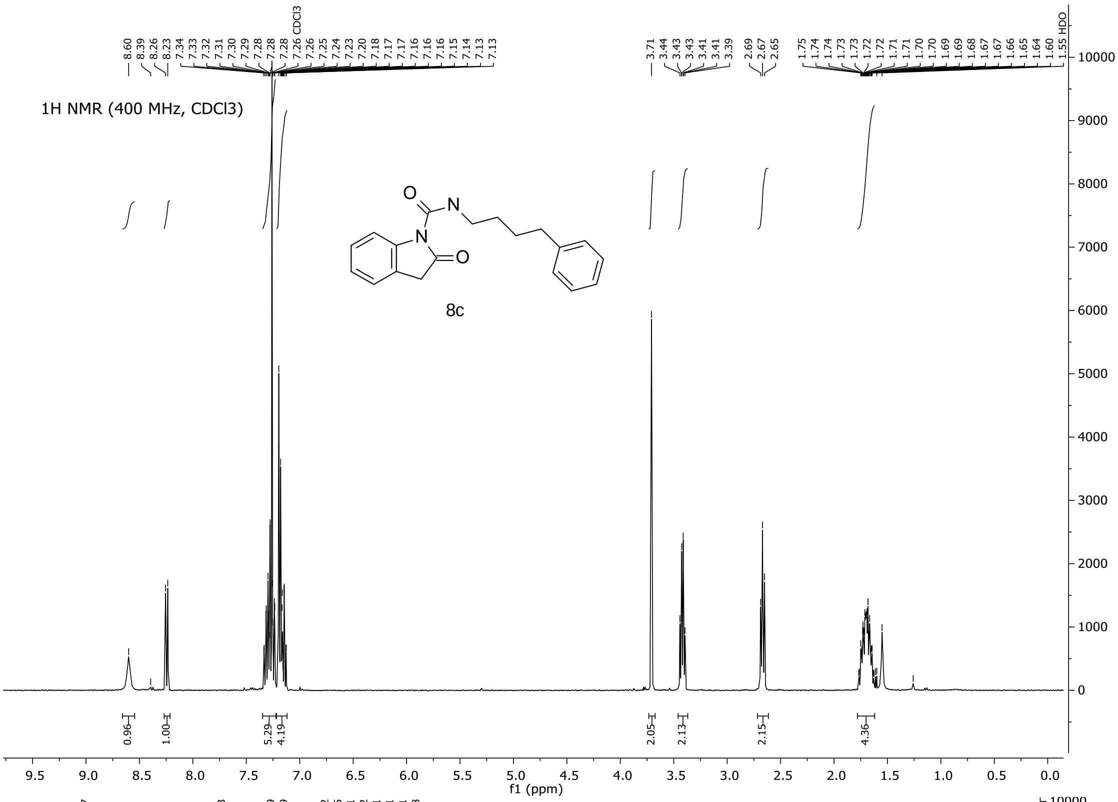
1H NMR (400 MHz, CDCl3)
8.60 8.39 8.26 8.23 7.34 7.33 7.32 7.31 7.30 7.29 7.28 7.28 7.28 7.26 CDCl3 7.26 7.25 7.24 7.23 7.20 7.18 7.17 7.17 7.16 7.16 7.16 7.15 7.14 7.13 7.13
3.71 3.44 3.43 3.43 3.41 3.41 3.39 2.69 2.67 2.65
1.75 1.74 1.74 1.73 1.73 1.72 1.72 1.71 1.71 1.70 1.70 1.69 1.69 1.68 1.67 1.67 1.66 1.65 1.64 1.60 1.55 HDO
0.96 1.00 5.29 4.19 2.05 2.13 2.15 4.36
8c
f1 (ppm)

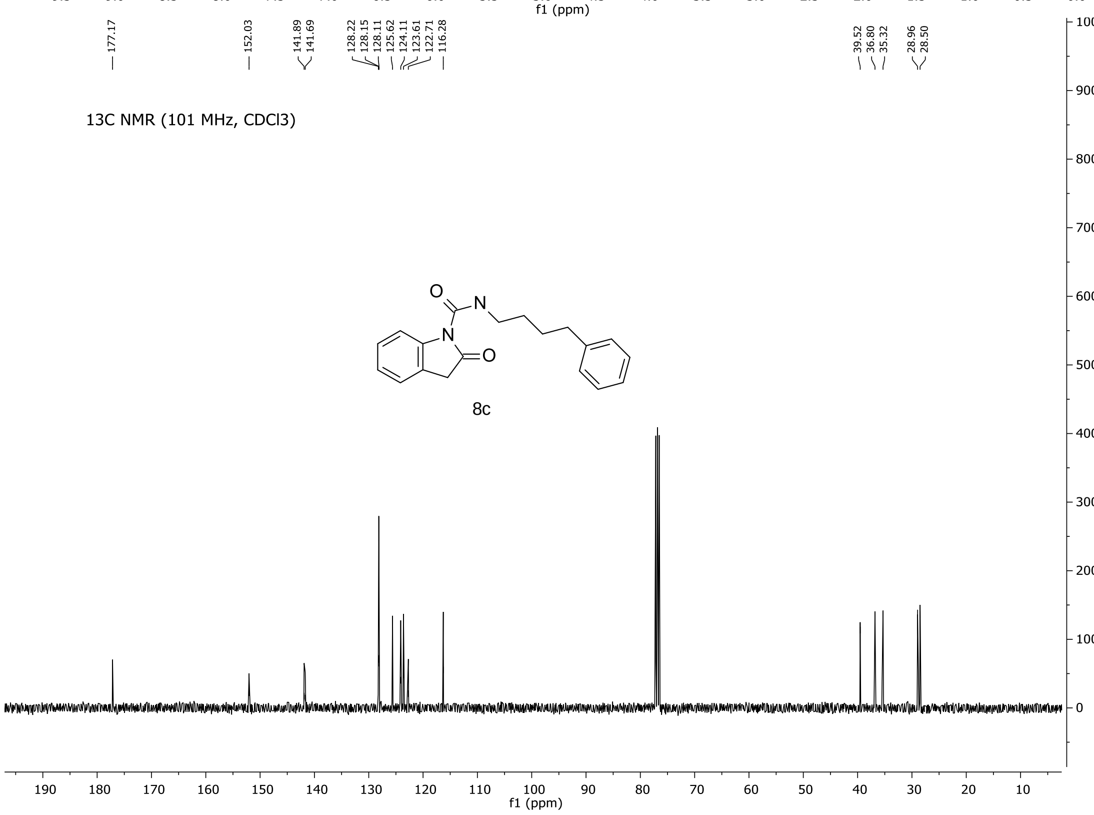
13C NMR (101 MHz, CDCl3)
177.17 152.03 141.89 141.69 128.22 128.15 128.11 125.62 124.11 123.61 122.71 116.28
39.52 36.80 35.32 28.96 28.50
8c
f1 (ppm)

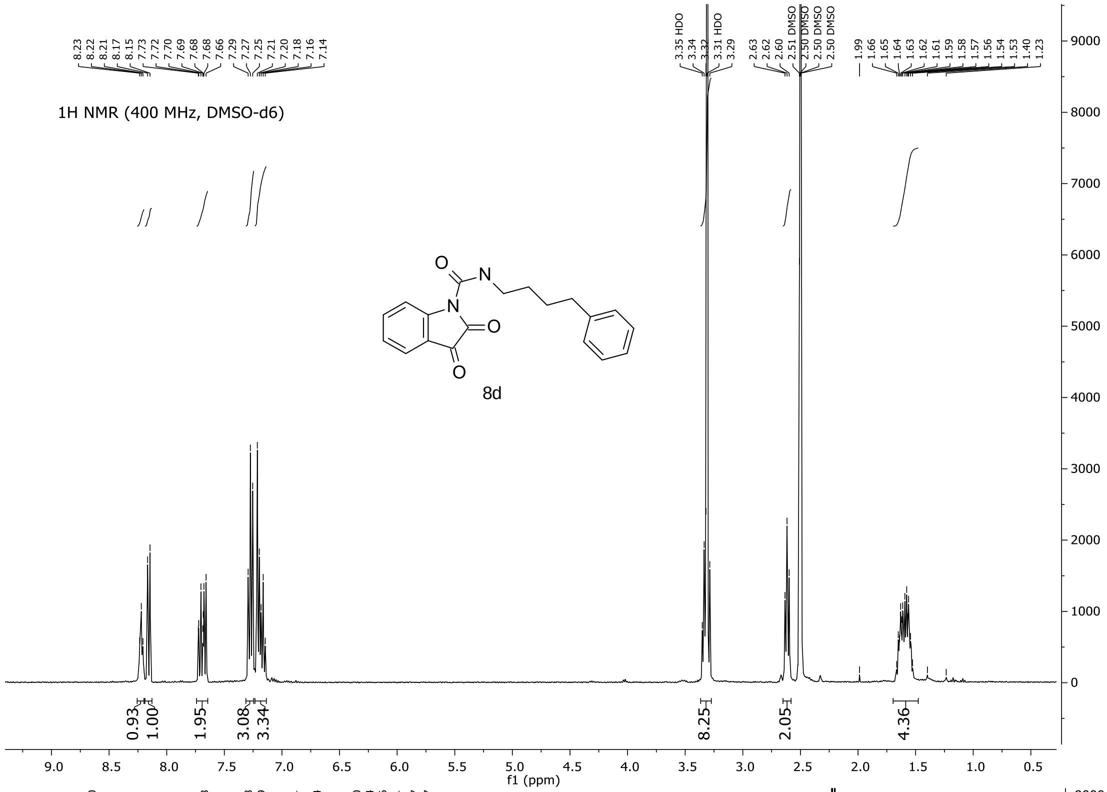
1H NMR (400 MHz, DMSO-d6)
8d
f1 (ppm)

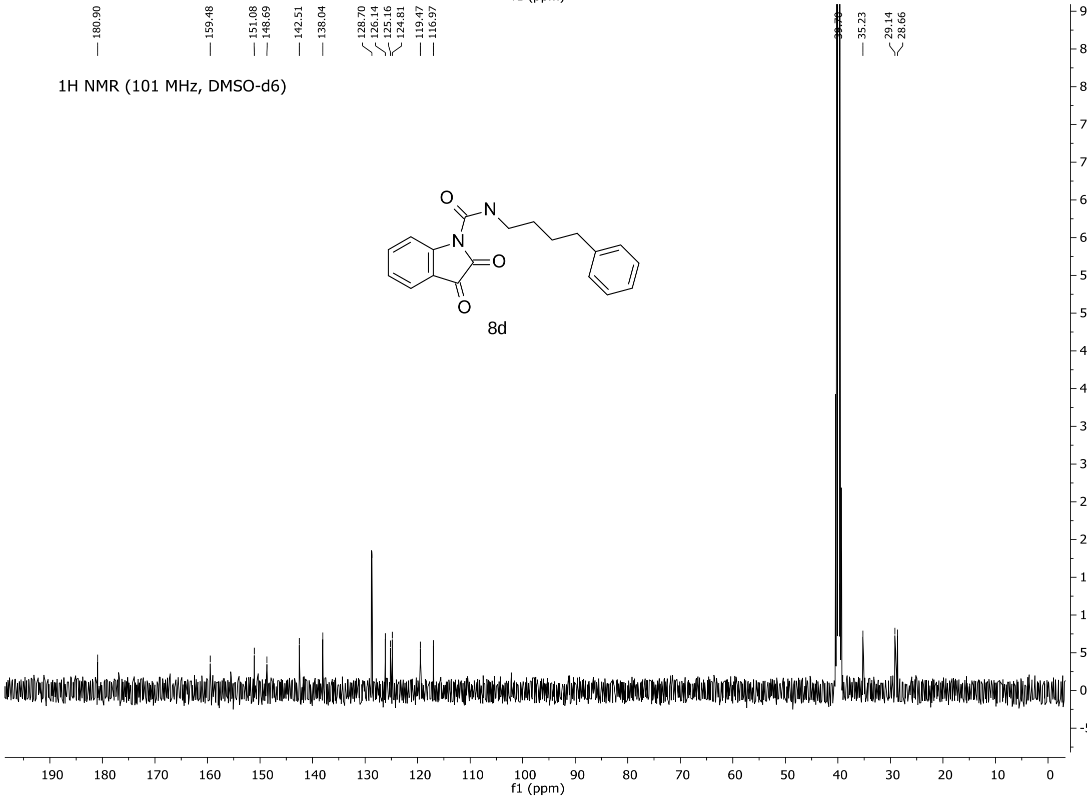
1H NMR (101 MHz, DMSO-d6)
8d
f1 (ppm)

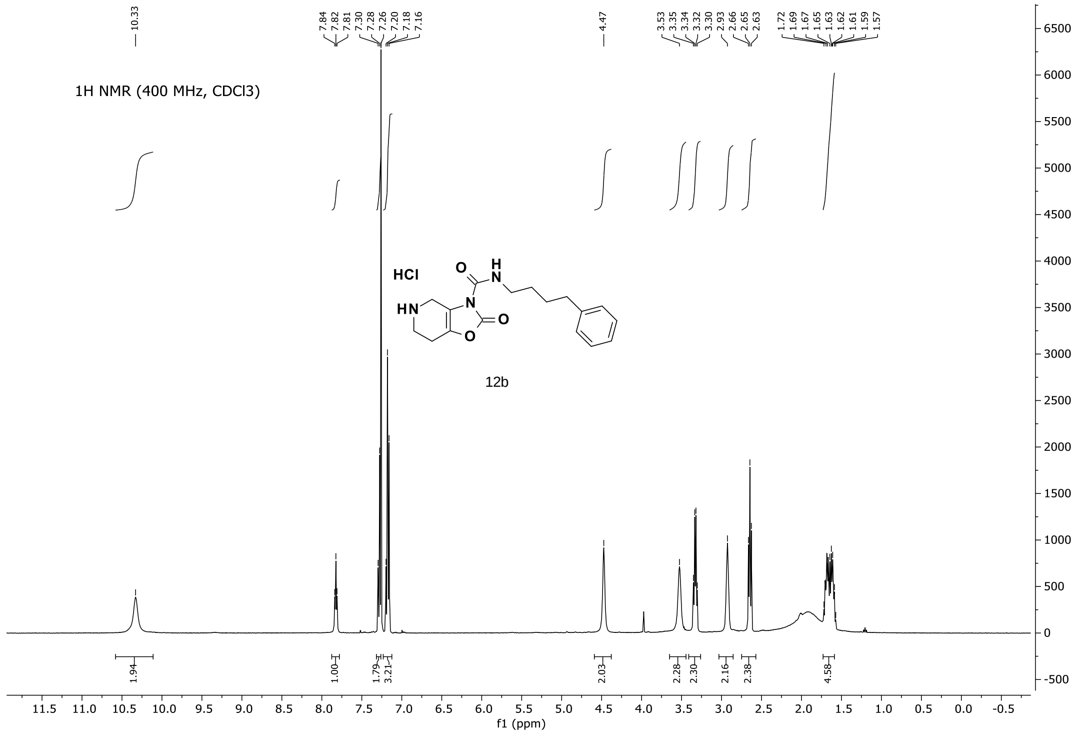
1H NMR (400 MHz, CDCl3)
HCl
12b
10.33
7.84 7.82 7.81 7.30 7.28 7.26 7.20 7.18 7.16
4.47
3.53 3.35 3.34 3.32 3.30 2.93 2.66 2.65 2.63
1.72 1.69 1.67 1.65 1.63 1.62 1.61 1.59 1.57
1.94 1.00 1.79 3.21 2.03 2.28 2.30 2.16 2.38 4.58
f1 (ppm)

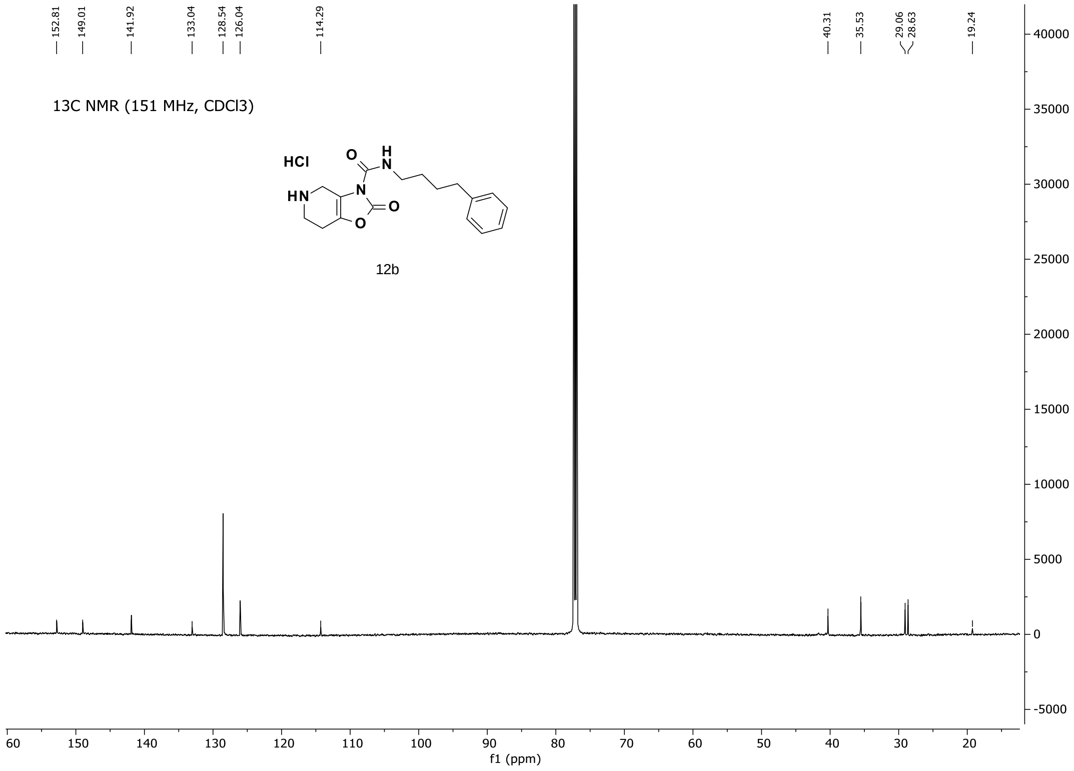
13C NMR (151 MHz, CDCl3)
HCl
12b
152.81 149.01 141.92 133.04 128.54 126.04 114.29
40.31 35.53 29.06 28.63 19.24
f1 (ppm)

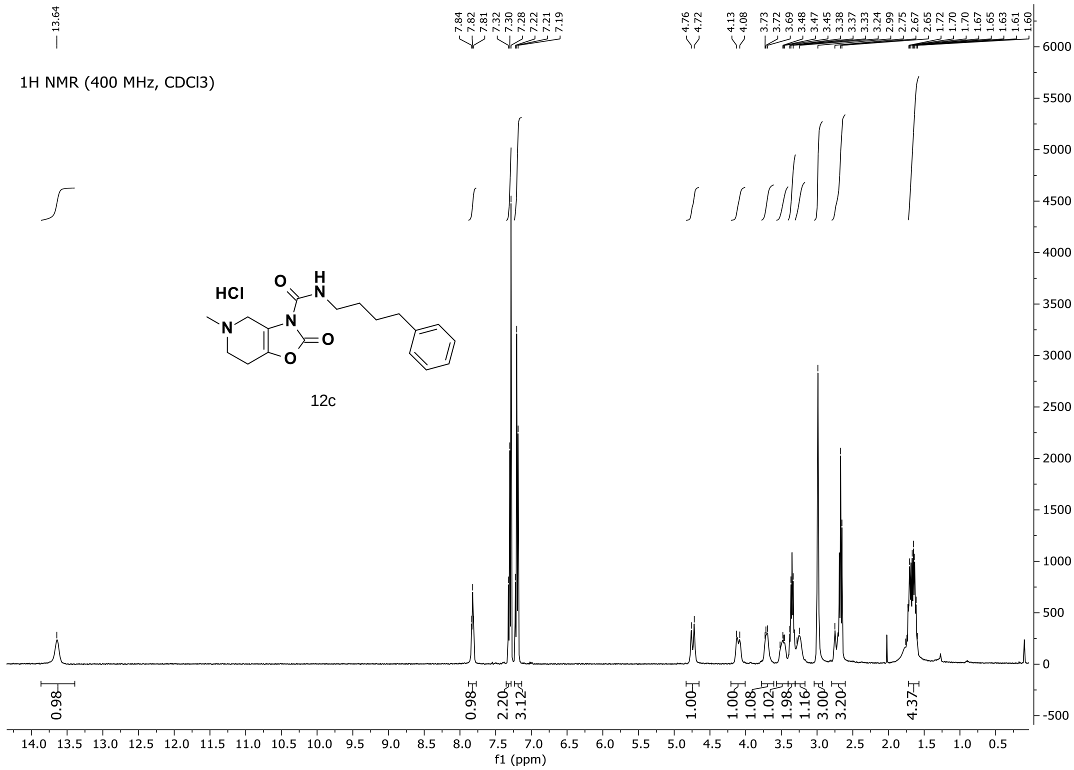
1H NMR (400 MHz, CDCl3)
HCl
12c
f1 (ppm)

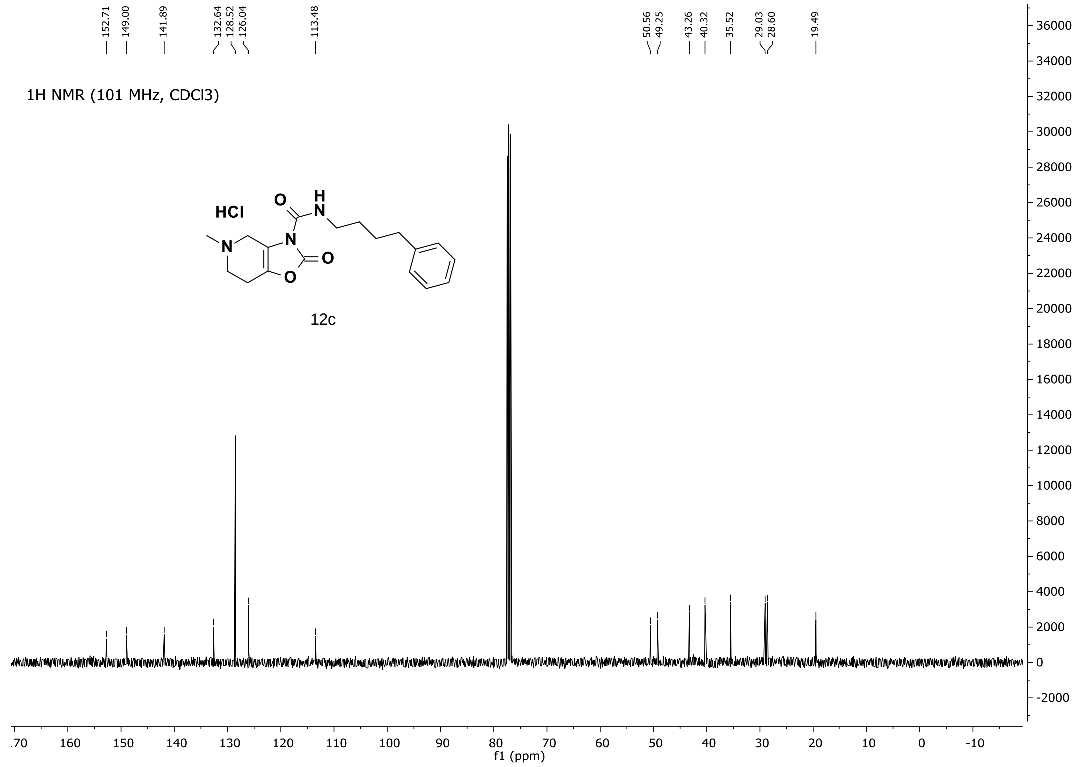
1H NMR (101 MHz, CDCl3)
HCl
12c
f1 (ppm)

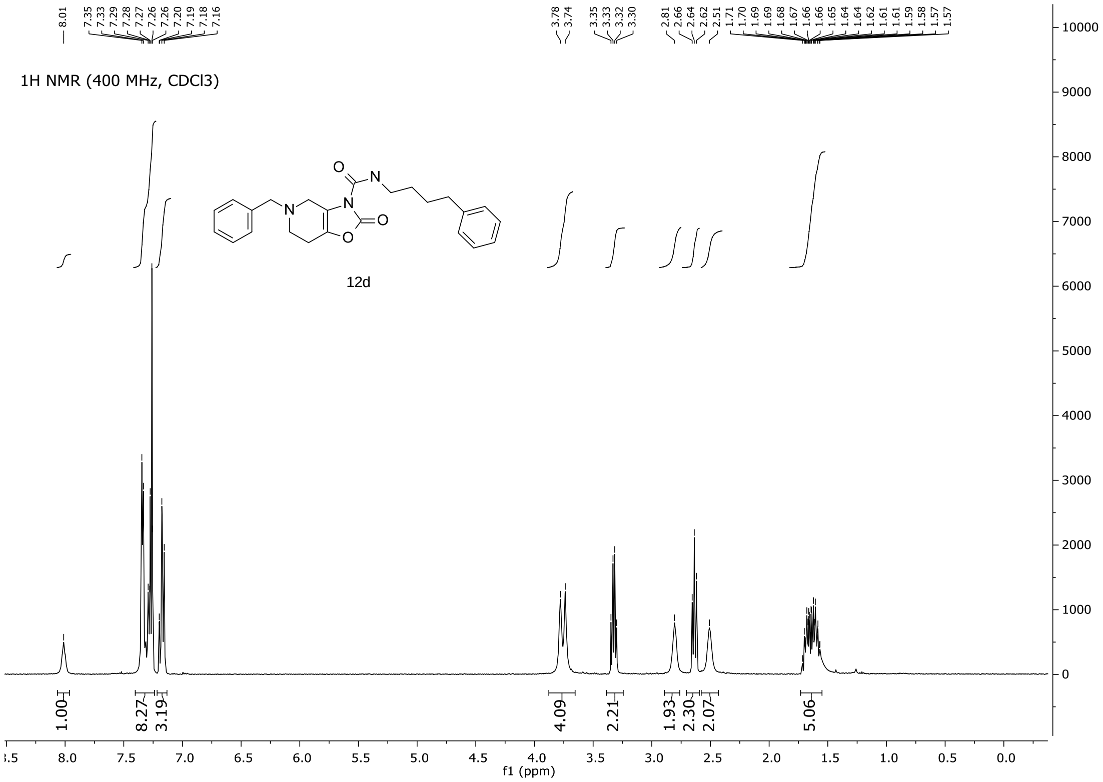
1H NMR (400 MHz, CDCl3)
12d
f1 (ppm)

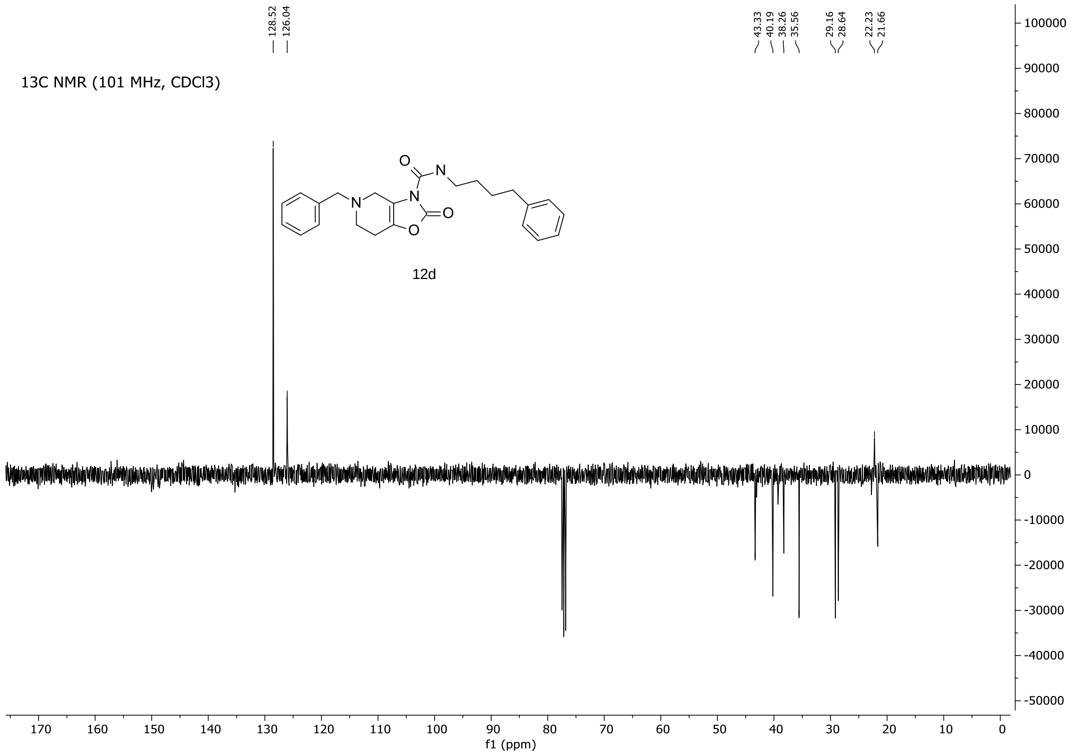
13C NMR (101 MHz, CDCl3)
12d
f1 (ppm)

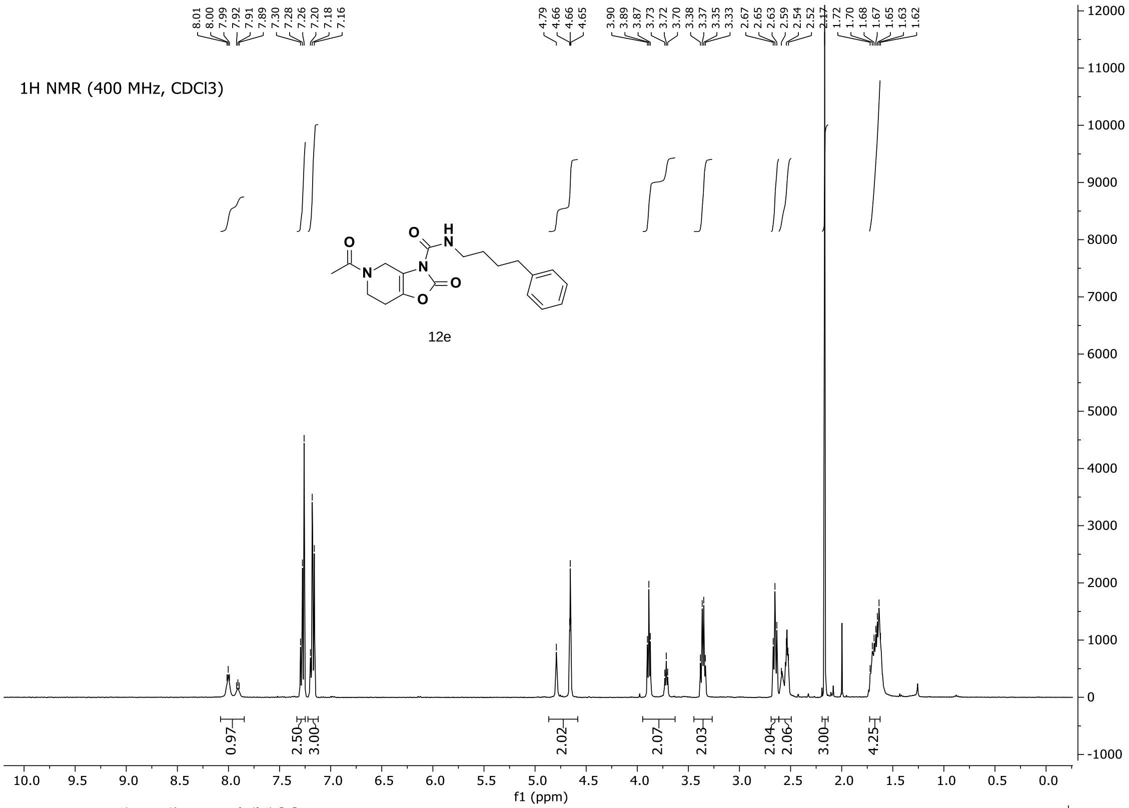
1H NMR (400 MHz, CDCl3)
8.01 8.00 7.99 7.92 7.91 7.89 7.30 7.28 7.26 7.20 7.18 7.16
4.79 4.66 4.66 4.65
3.90 3.89 3.87 3.73 3.72 3.70 3.38 3.37 3.35 3.33
2.67 2.65 2.63 2.59 2.54 2.52 2.17
1.72 1.70 1.68 1.67 1.65 1.63 1.62
12e
0.97 2.50 3.00 2.02 2.07 2.03 2.04 2.06 3.00 4.25
f1 (ppm)

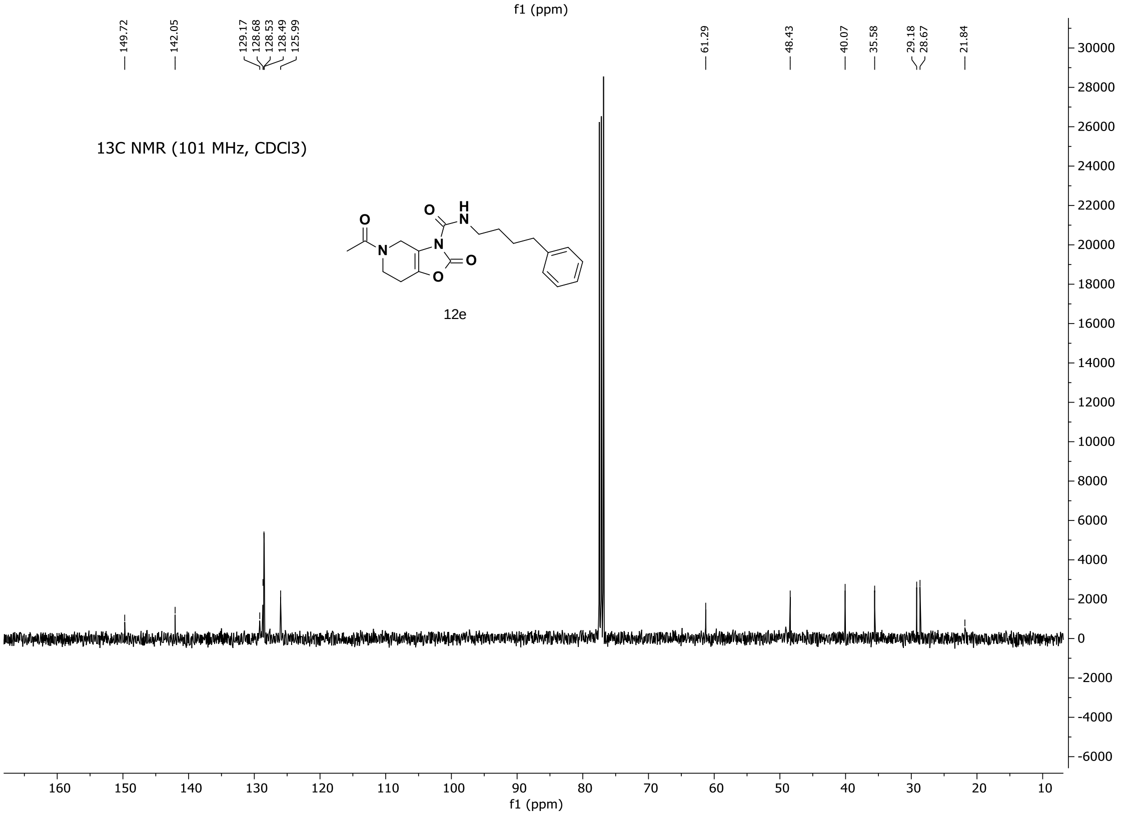
13C NMR (101 MHz, CDCl3)
149.72
142.05
129.17 128.68 128.53 128.49 125.99
61.29
48.43
40.07
35.58
29.18 28.67
21.84
12e
f1 (ppm)

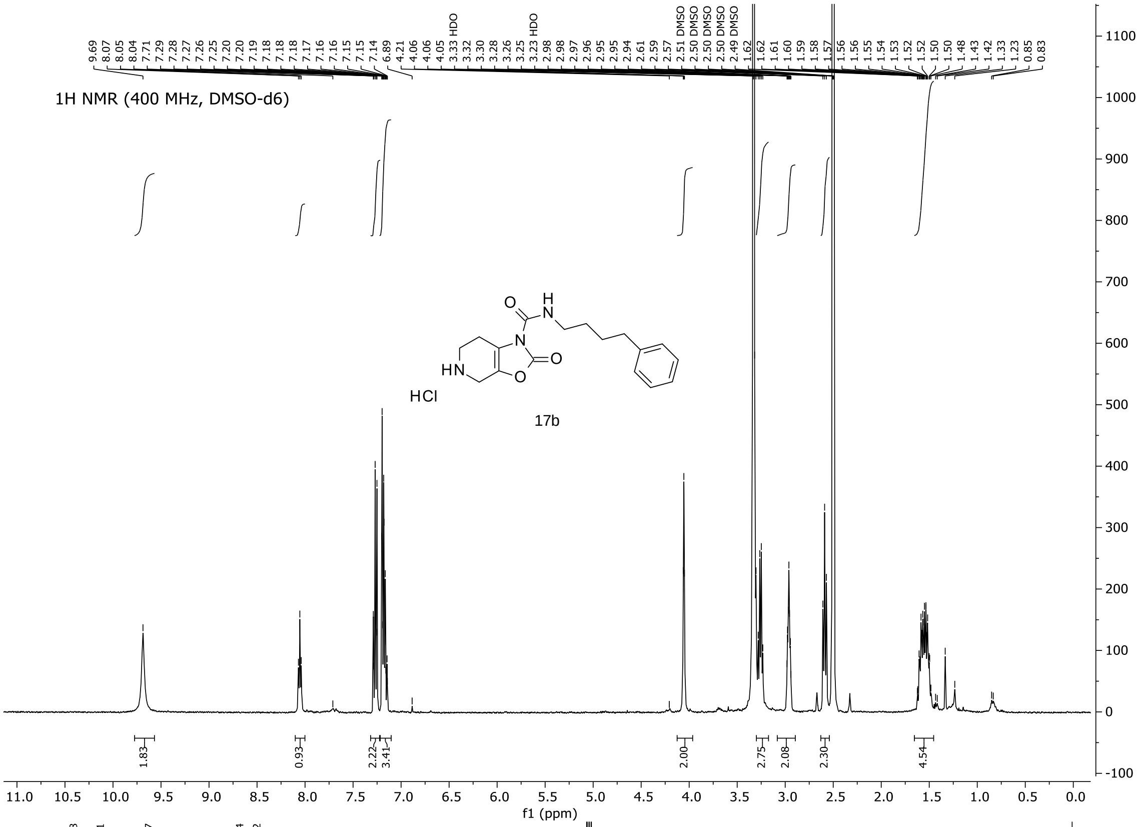
1H NMR (400 MHz, DMSO-d6)
HCl
17b
f1 (ppm)

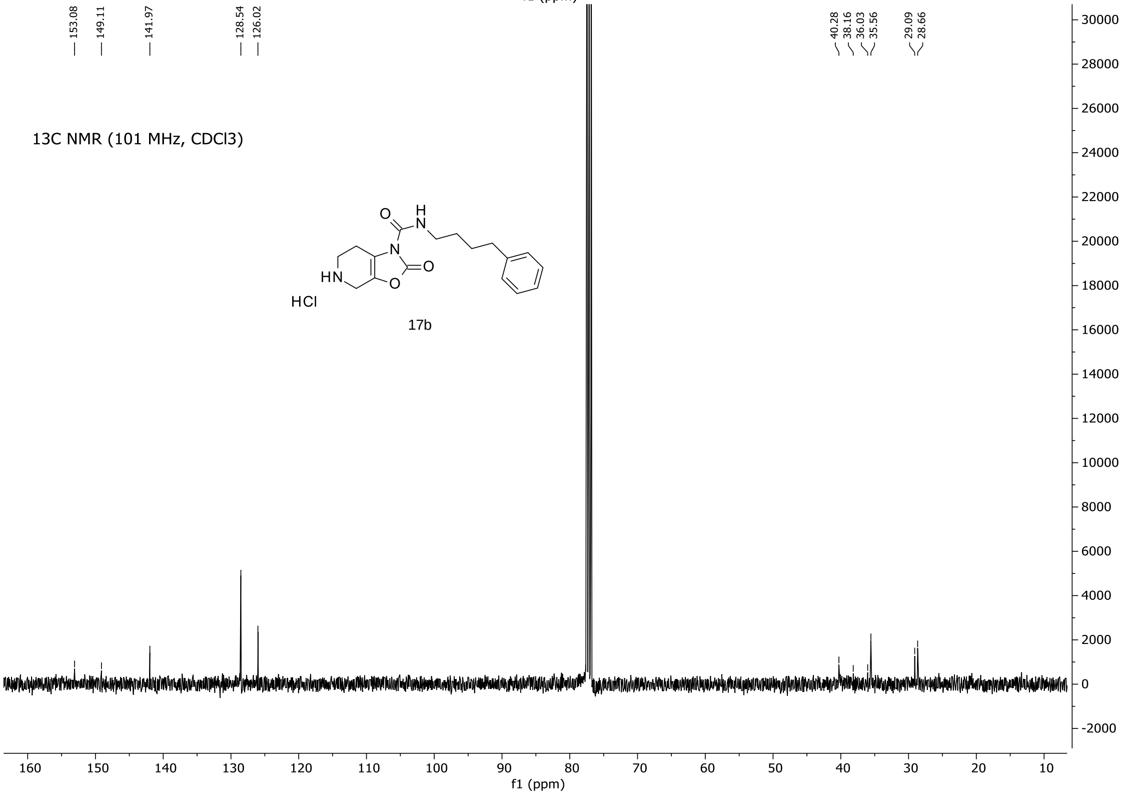
13C NMR (101 MHz, CDCl3)
HCl
17b
f1 (ppm)

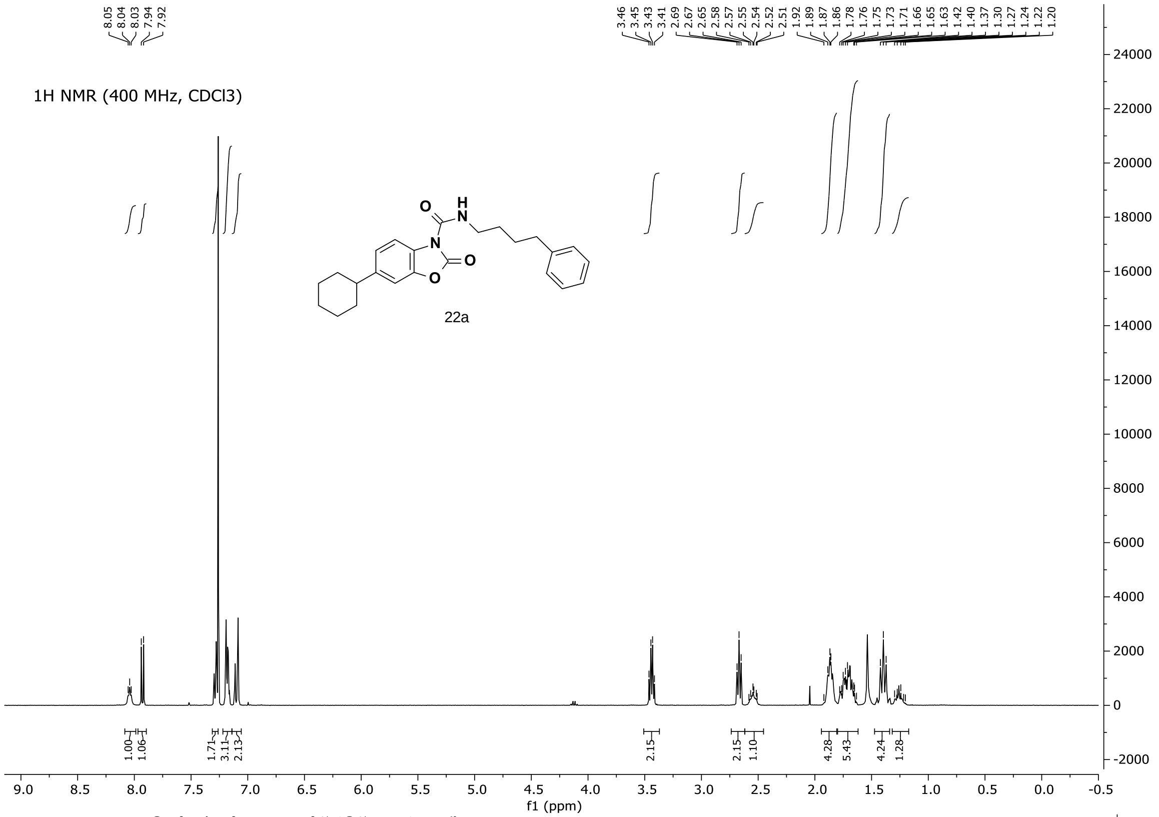

1H NMR (400 MHz, CDCl3)
8.05
8.04
8.03
7.94
7.92
3.46
3.45
3.43
3.41
2.69
2.67
2.65
2.58
2.57
2.55
2.54
2.52
2.51
1.92
1.89
1.87
1.86
1.78
1.76
1.75
1.73
1.71
1.66
1.65
1.63
1.42
1.40
1.37
1.30
1.27
1.24
1.22
1.20
22a
1.00
1.06
1.71
3.11
2.13
2.15
2.15
1.10
4.28
5.43
4.24
1.28
f1 (ppm)

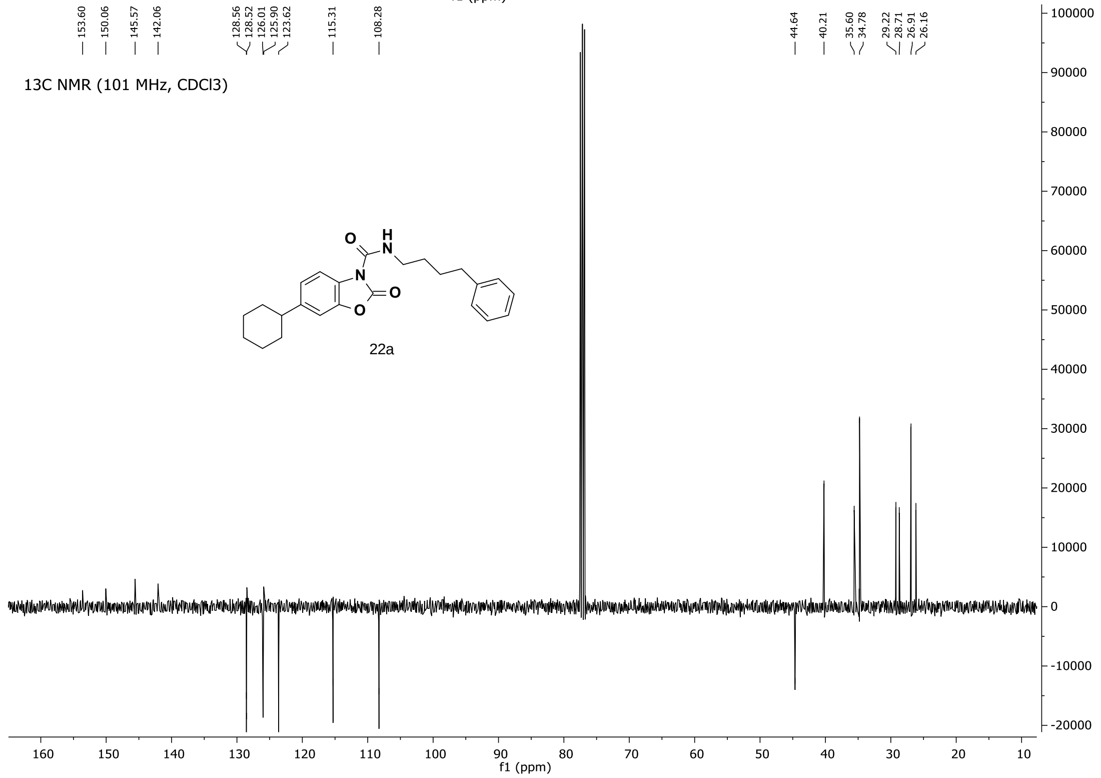

13C NMR (101 MHz, CDCl3)
153.60
150.06
145.57
142.06
128.56
128.52
126.01
125.90
123.62
115.31
108.28
44.64
40.21
35.60
34.78
29.22
28.71
26.91
26.16
22a
f1 (ppm)

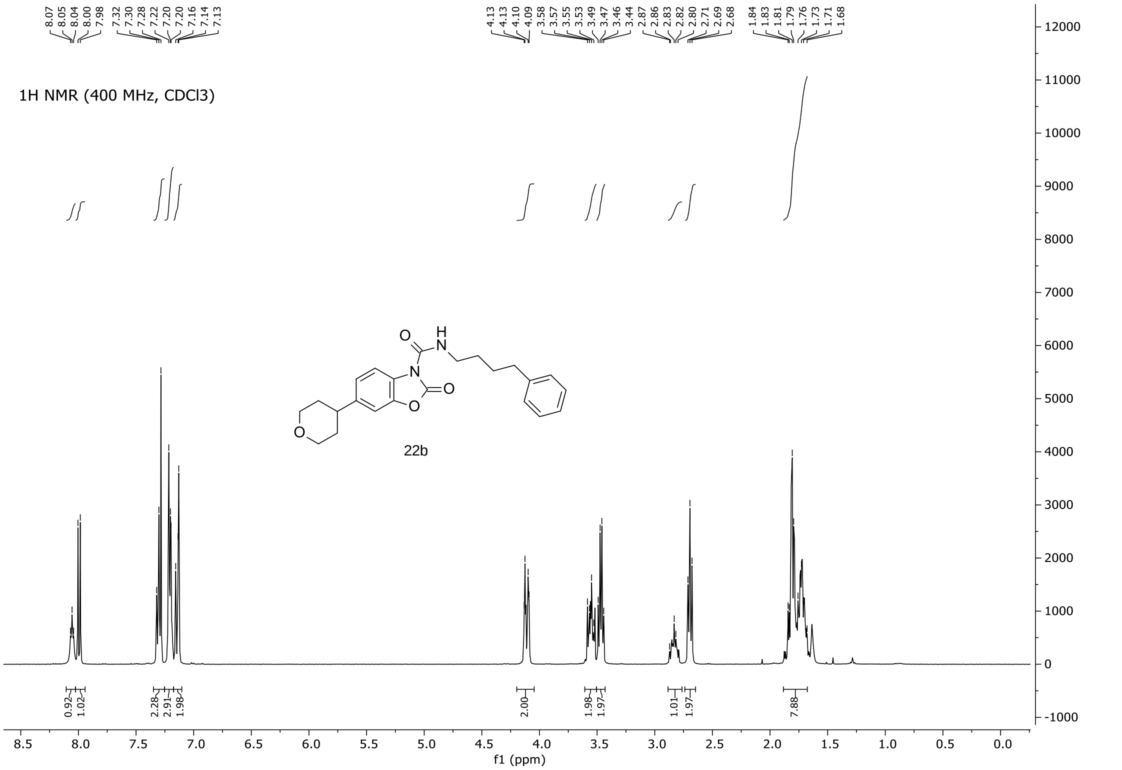
1H NMR (400 MHz, CDCl3)
8.07 8.05 8.04 8.00 7.98 7.32 7.30 7.28 7.22 7.20 7.20 7.16 7.14 7.13
4.13 4.13 4.10 4.09 3.58 3.57 3.55 3.53 3.49 3.47 3.46 3.44 2.87 2.86 2.83 2.82 2.80 2.71 2.69 2.68
1.84 1.83 1.81 1.79 1.76 1.73 1.71 1.68
22b
f1 (ppm)

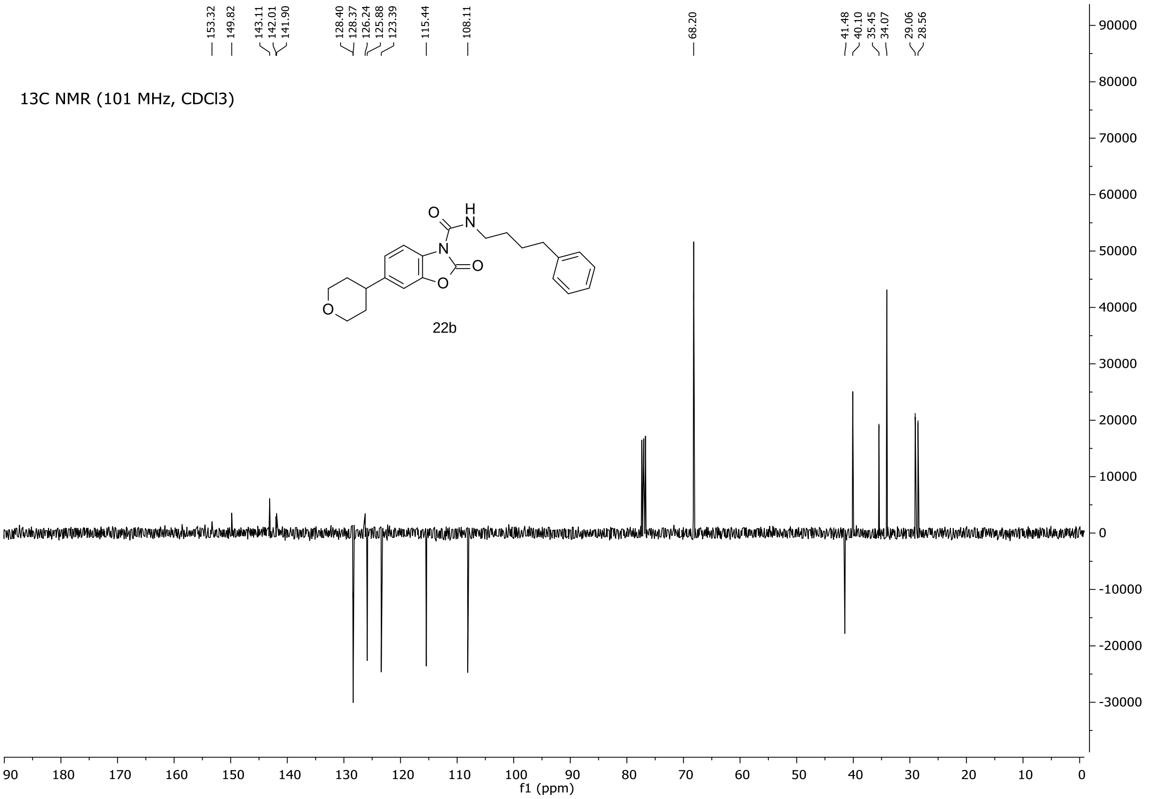
13C NMR (101 MHz, CDCl3)
153.32 149.82 143.11 142.01 141.90 128.40 128.37 126.24 125.88 123.39 115.44 108.11
68.20
41.48 40.10 35.45 34.07 29.06 28.56
22b
f1 (ppm)

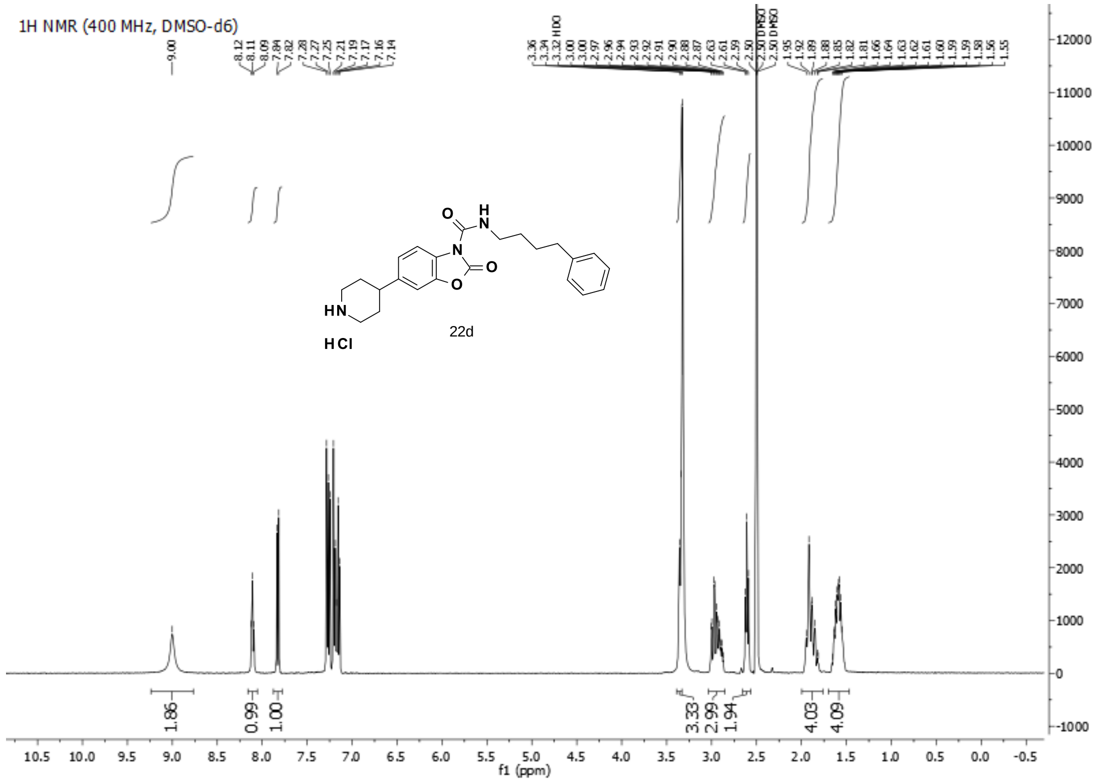
1H NMR (400 MHz, DMSO-d6)
22d
HCl

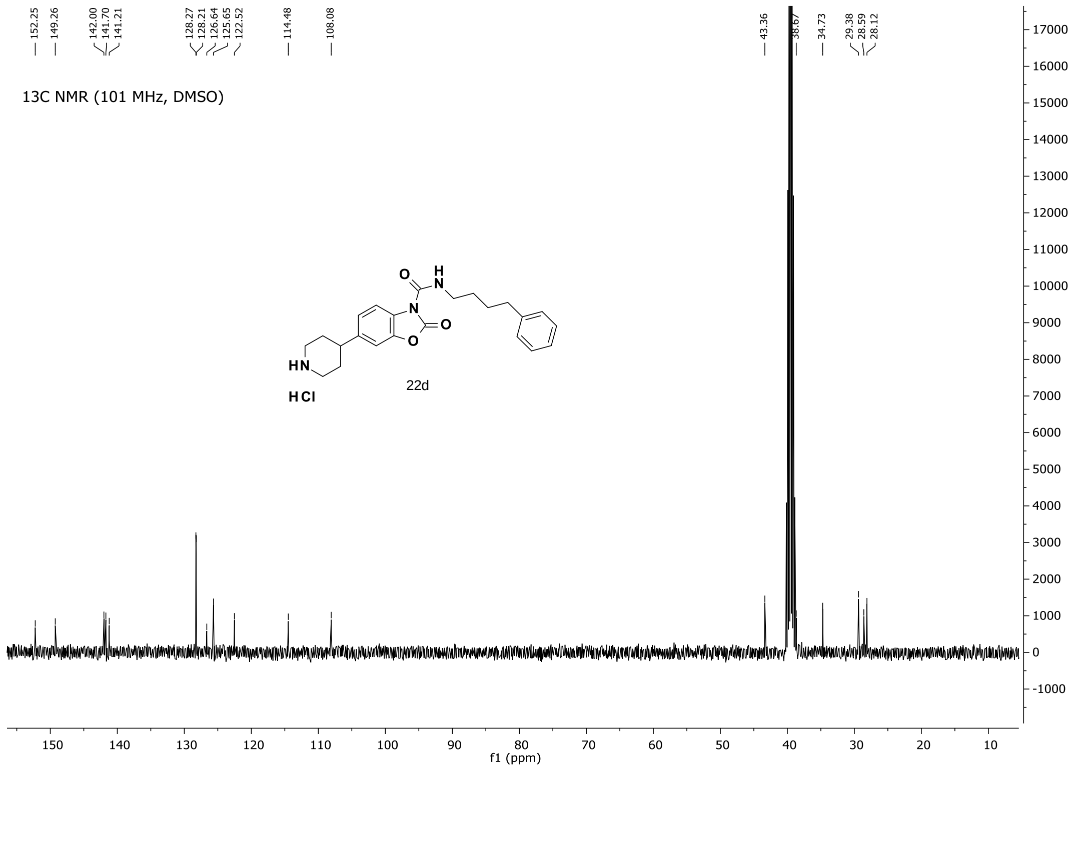
13C NMR (101 MHz, DMSO)
22d
HCl

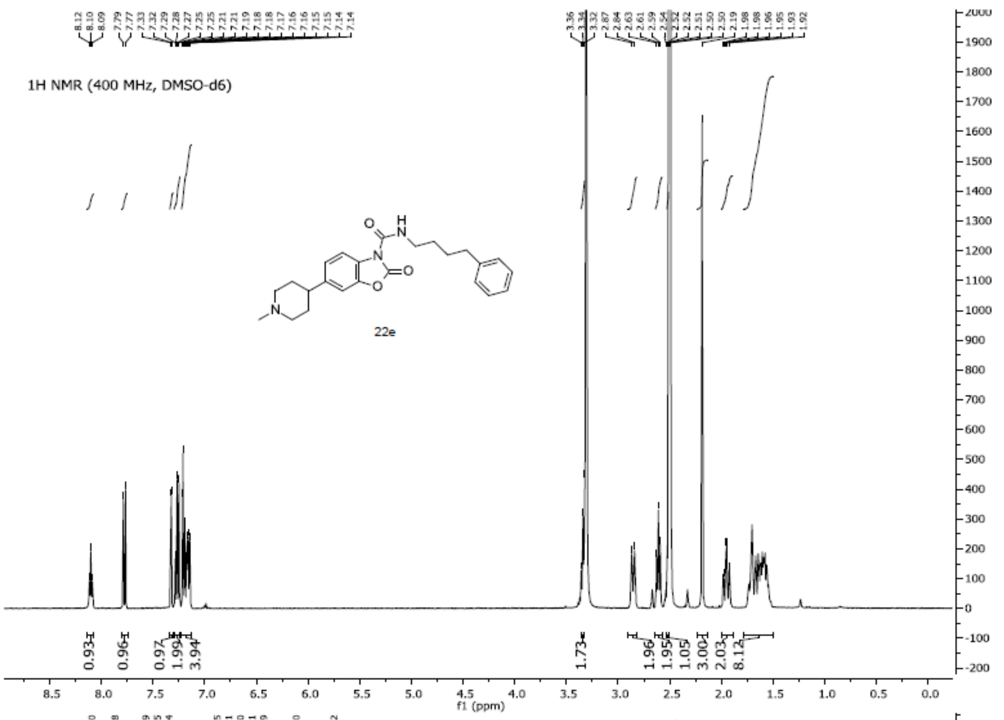
1H NMR (400 MHz, DMSO-d6)
22e
f1 (ppm)

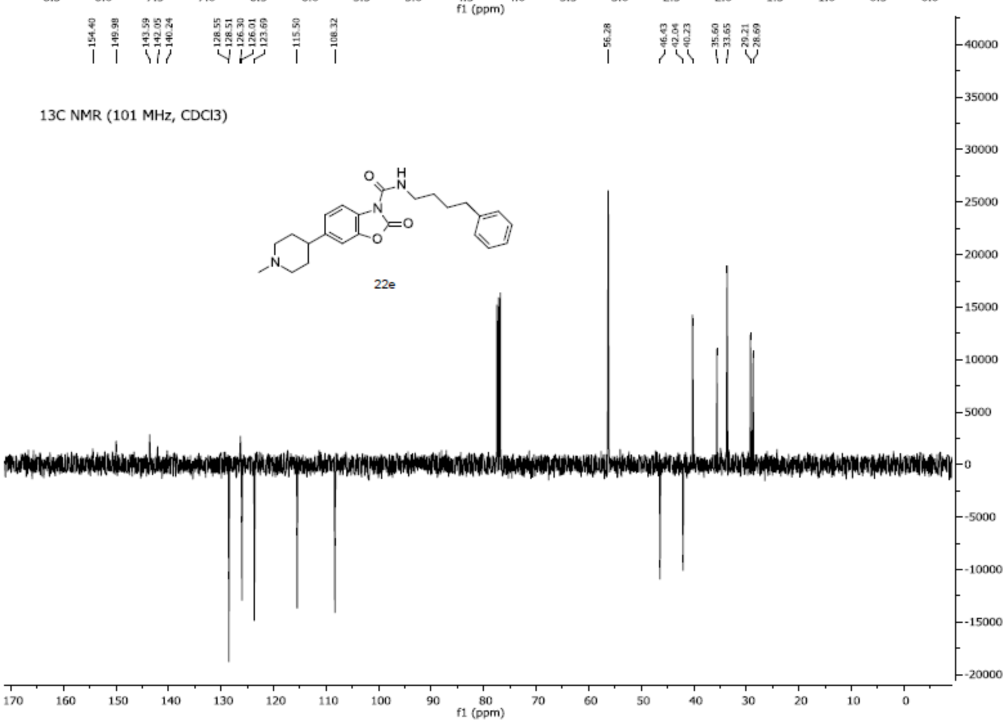
13C NMR (101 MHz, CDCl3)
22e
f1 (ppm)

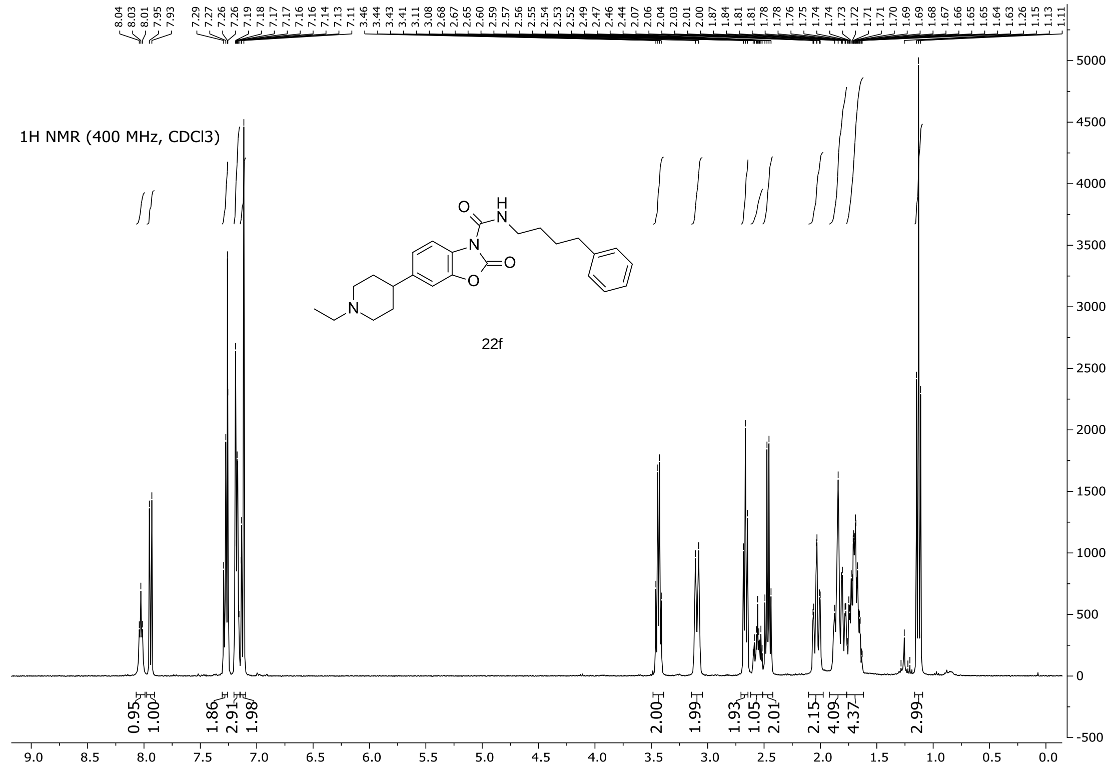
1H NMR (400 MHz, CDCl3)
22f
8.04 8.03 8.01 7.95 7.93 7.29 7.27 7.26 7.26 7.19 7.18 7.17 7.17 7.16 7.16 7.14 7.13 7.11 3.46 3.44 3.43 3.41 3.11 3.08 2.68 2.67 2.65 2.60 2.59 2.57 2.56 2.55 2.54 2.53 2.52 2.49 2.47 2.46 2.44 2.07 2.06 2.04 2.03 2.01 2.00 1.87 1.84 1.81 1.81 1.78 1.78 1.76 1.75 1.74 1.74 1.73 1.72 1.72 1.71 1.71 1.70 1.69 1.69 1.68 1.67 1.66 1.65 1.65 1.64 1.63 1.26 1.15 1.13 1.11
0.95 1.00 1.86 2.91 1.98 2.00 1.99 1.93 1.05 2.01 2.15 4.09 4.37 2.99
f1 (ppm)

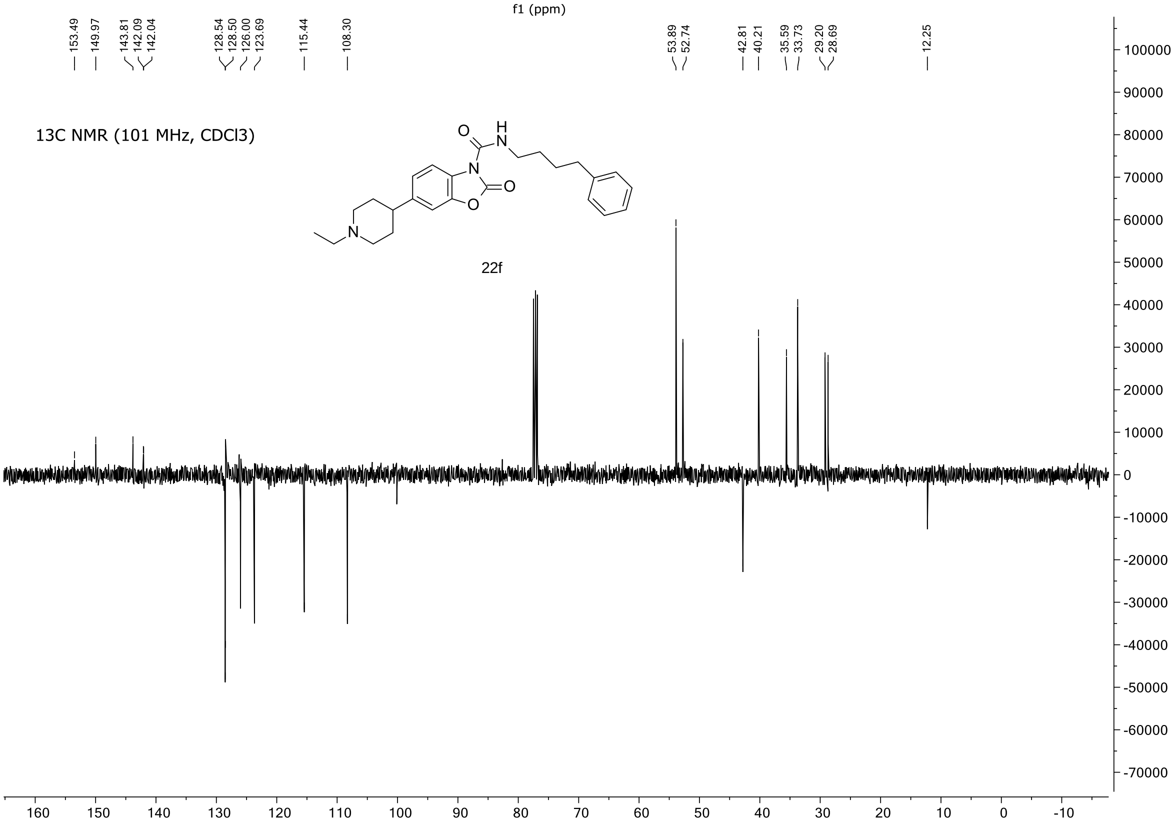
13C NMR (101 MHz, CDCl3)
22f
153.49 149.97 143.81 142.09 142.04 128.54 128.50 126.00 123.69 115.44 108.30 53.89 52.74 42.81 40.21 35.59 33.73 29.20 28.69 12.25
f1 (ppm)

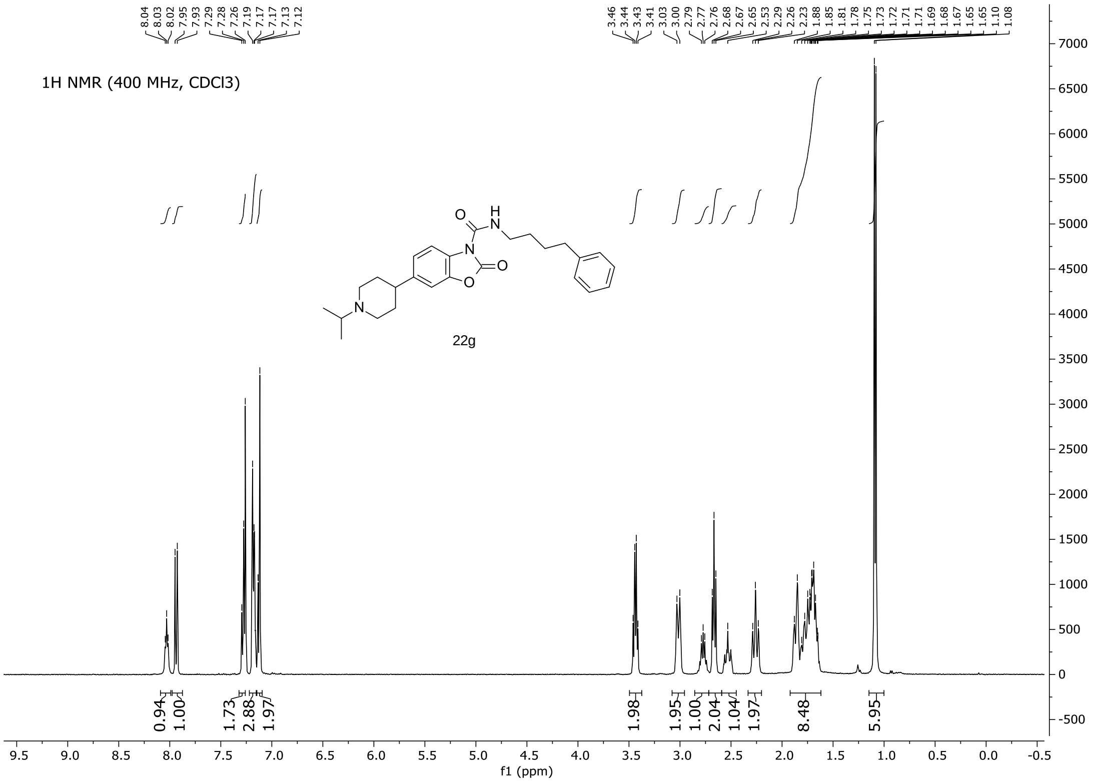

1H NMR (400 MHz, CDCl3)
22g
f1 (ppm)

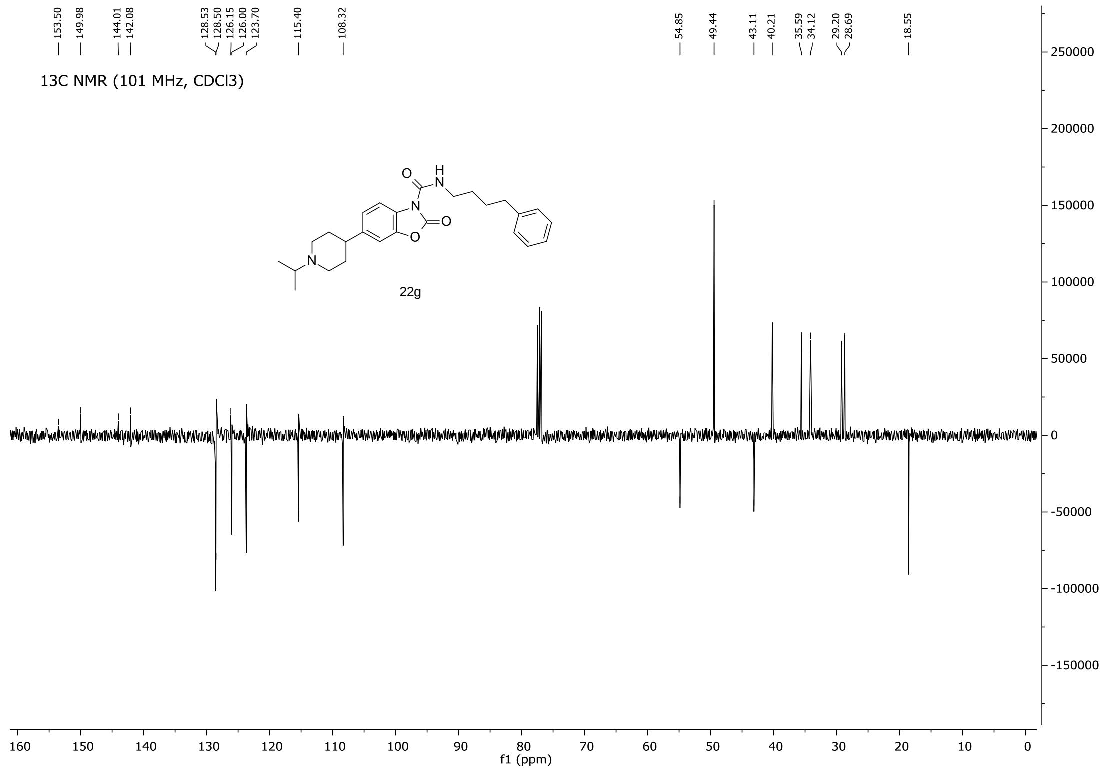

13C NMR (101 MHz, CDCl3)
22g
f1 (ppm)

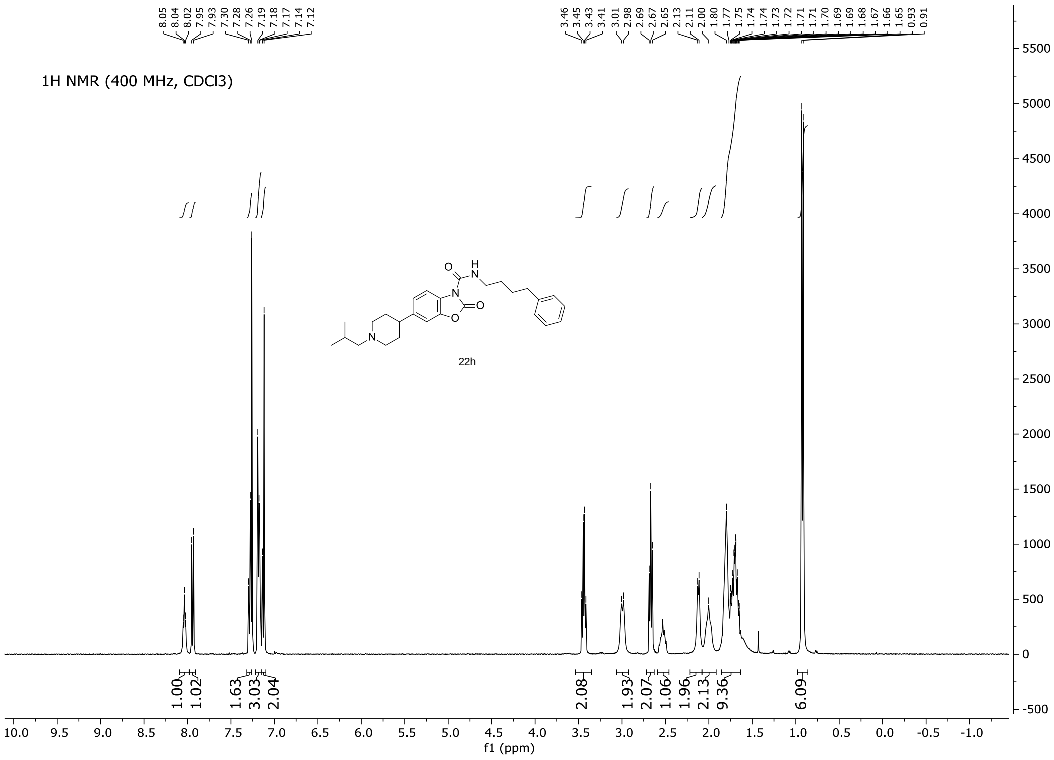
1H NMR (400 MHz, CDCl3)
22h
f1 (ppm)

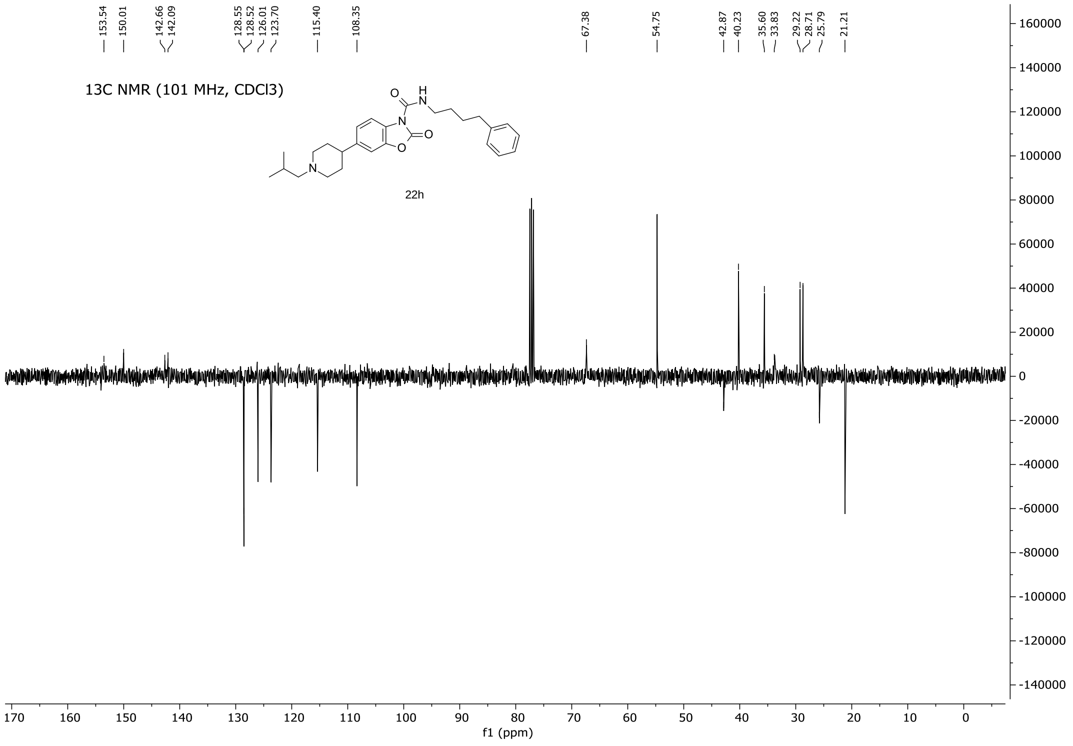
13C NMR (101 MHz, CDCl3)
22h
f1 (ppm)

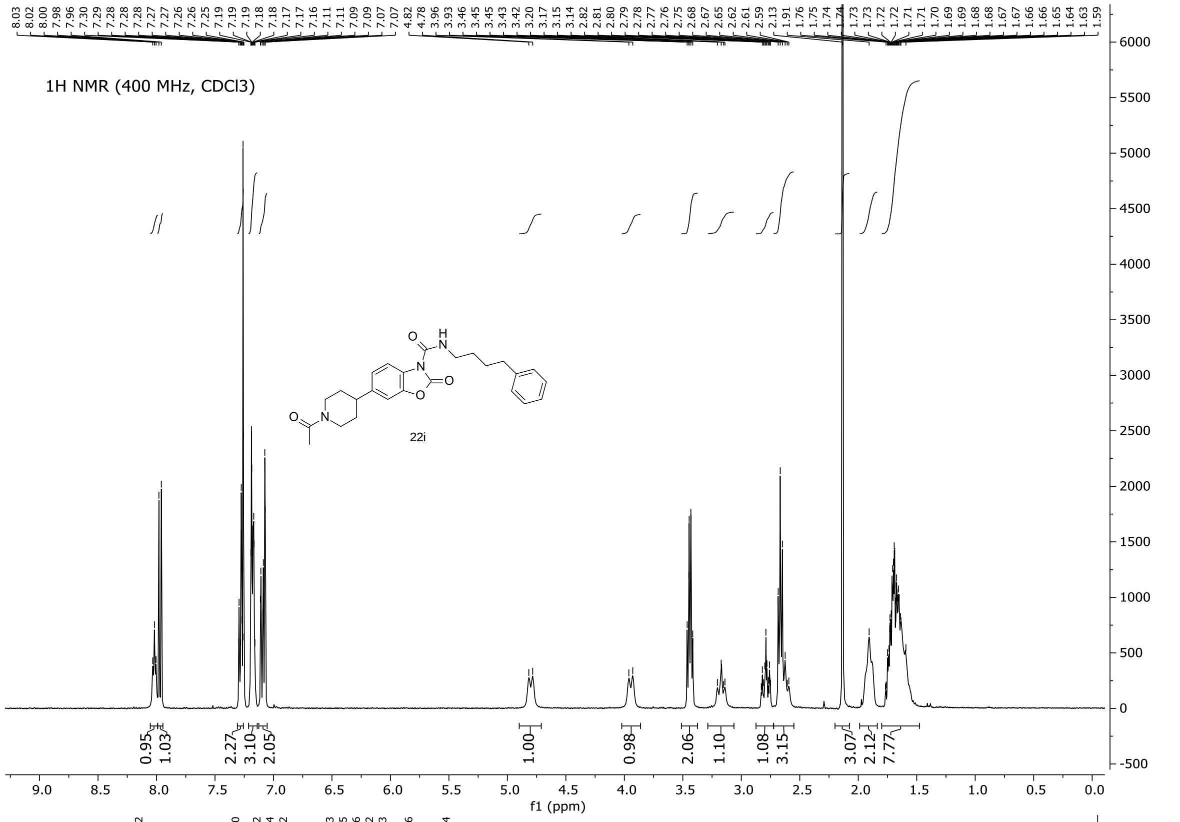

1H NMR (400 MHz, CDCl3)
22i
f1 (ppm)

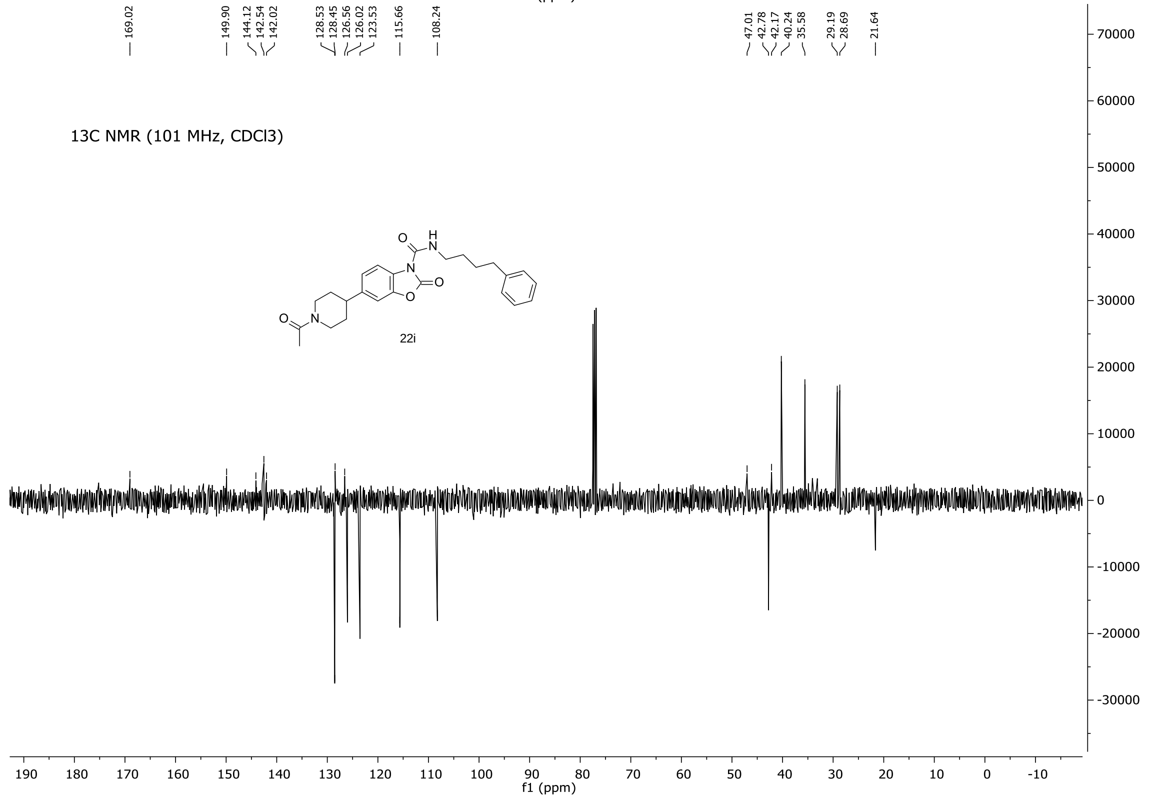

13C NMR (101 MHz, CDCl3)
22i
169.02
149.90
144.12
142.54
142.02
128.53
128.45
126.56
126.02
123.53
115.66
108.24
47.01
42.78
42.17
40.24
35.58
29.19
28.69
21.64
f1 (ppm)

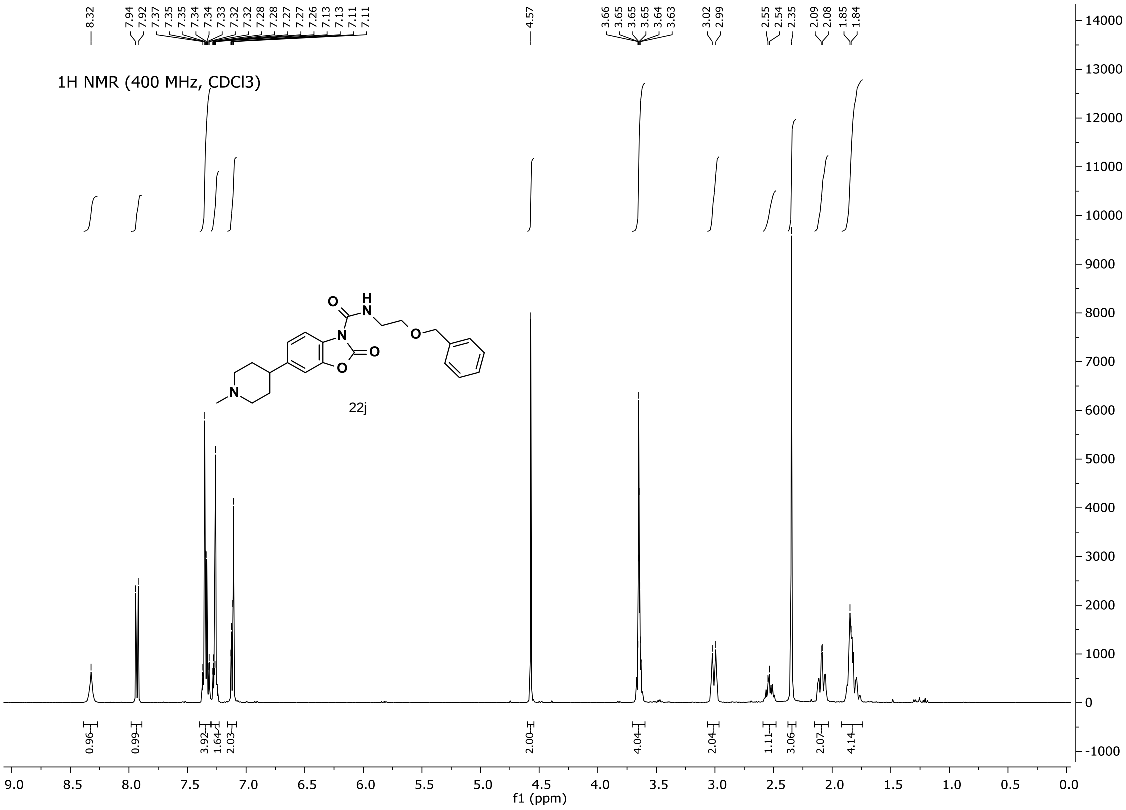
1H NMR (400 MHz, CDCl3)
22j
8.32
7.94
7.92
7.37
7.35
7.35
7.34
7.34
7.33
7.32
7.32
7.28
7.28
7.27
7.27
7.26
7.13
7.13
7.11
7.11
4.57
3.66
3.65
3.65
3.65
3.64
3.63
3.02
2.99
2.55
2.54
2.35
2.09
2.08
1.85
1.84
0.96
0.99
3.92
1.64
2.03
2.00
4.04
2.04
1.11
3.06
2.07
4.14
f1 (ppm)

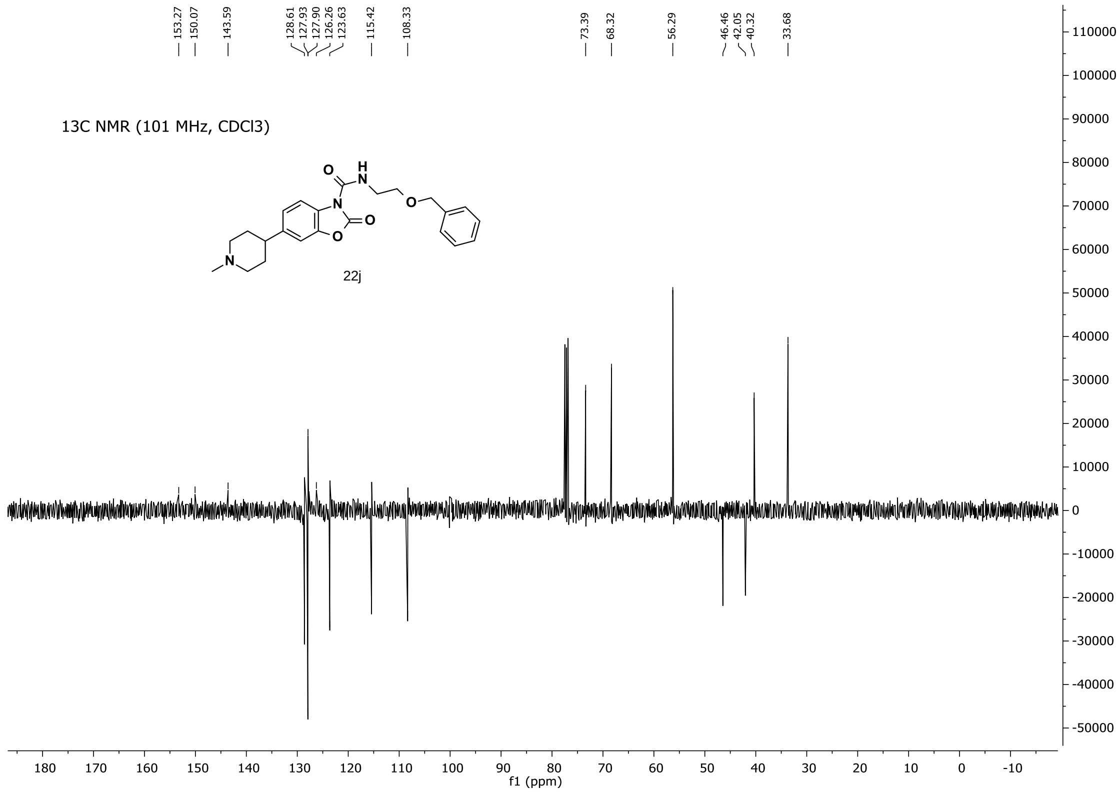
13C NMR (101 MHz, CDCl3)
22j
153.27
150.07
143.59
128.61
127.93
127.90
126.26
123.63
115.42
108.33
73.39
68.32
56.29
46.46
42.05
40.32
33.68
f1 (ppm)

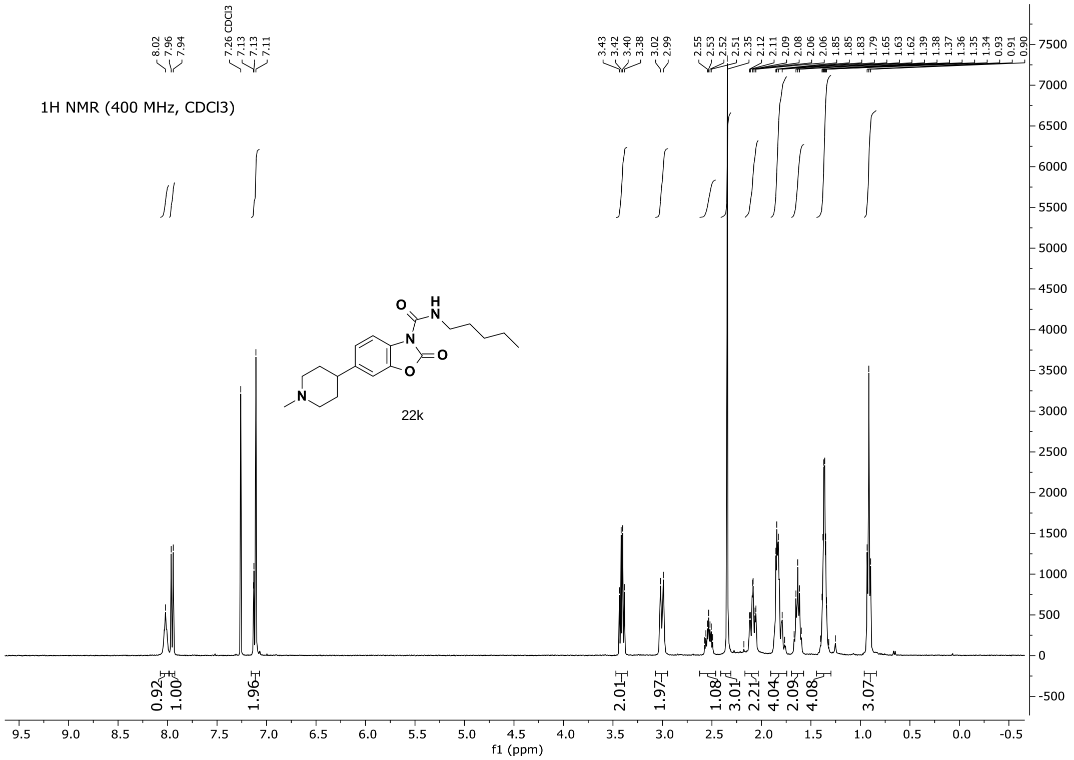
1H NMR (400 MHz, CDCl3)
8.02 7.96 7.94 7.26 CDCl3 7.13 7.13 7.11
3.43 3.42 3.40 3.38 3.02 2.99
2.55 2.53 2.52 2.51 2.35 2.12 2.11 2.09 2.08 2.06 2.06 1.85 1.85 1.83 1.79 1.65 1.63 1.62 1.39 1.38 1.37 1.36 1.35 1.34 0.93 0.91 0.90
0.92 1.00 1.96 2.01 1.97 1.08 3.01 2.21 4.04 2.09 4.08 3.07
22k
f1 (ppm)

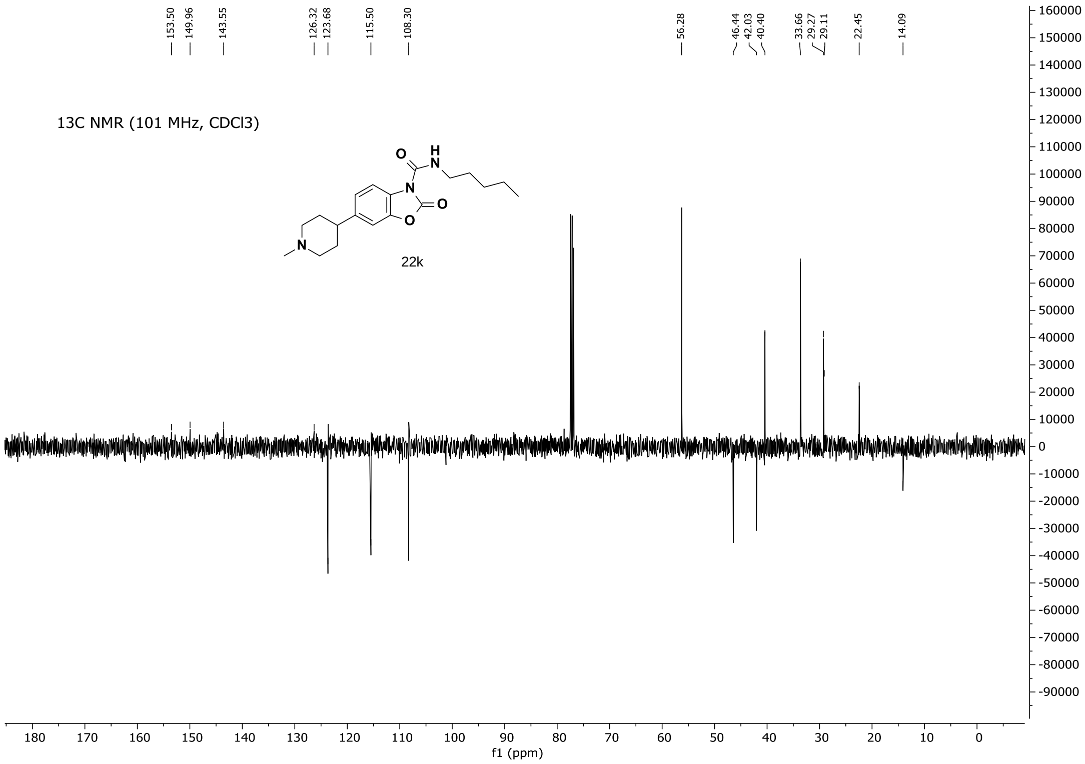
13C NMR (101 MHz, CDCl3)
153.50 149.96 143.55 126.32 123.68 115.50 108.30
56.28 46.44 42.03 40.40 33.66 29.27 29.11 22.45 14.09
22k
f1 (ppm)

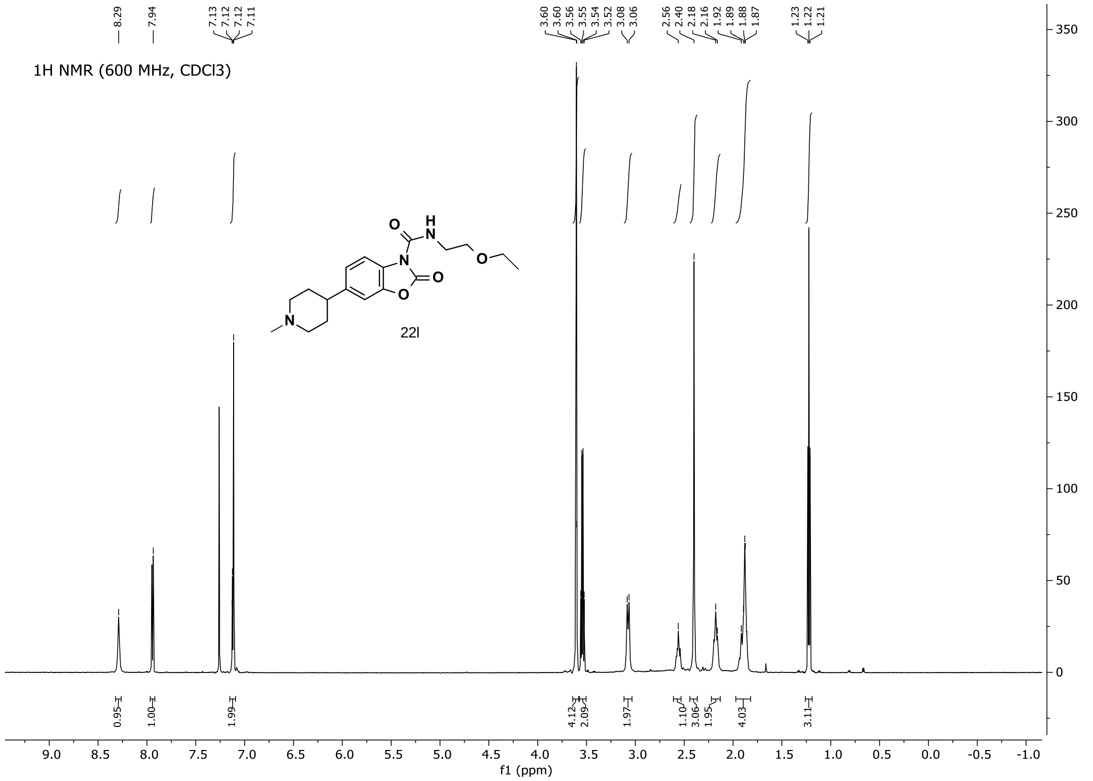
1H NMR (600 MHz, CDCl3)
22l
f1 (ppm)

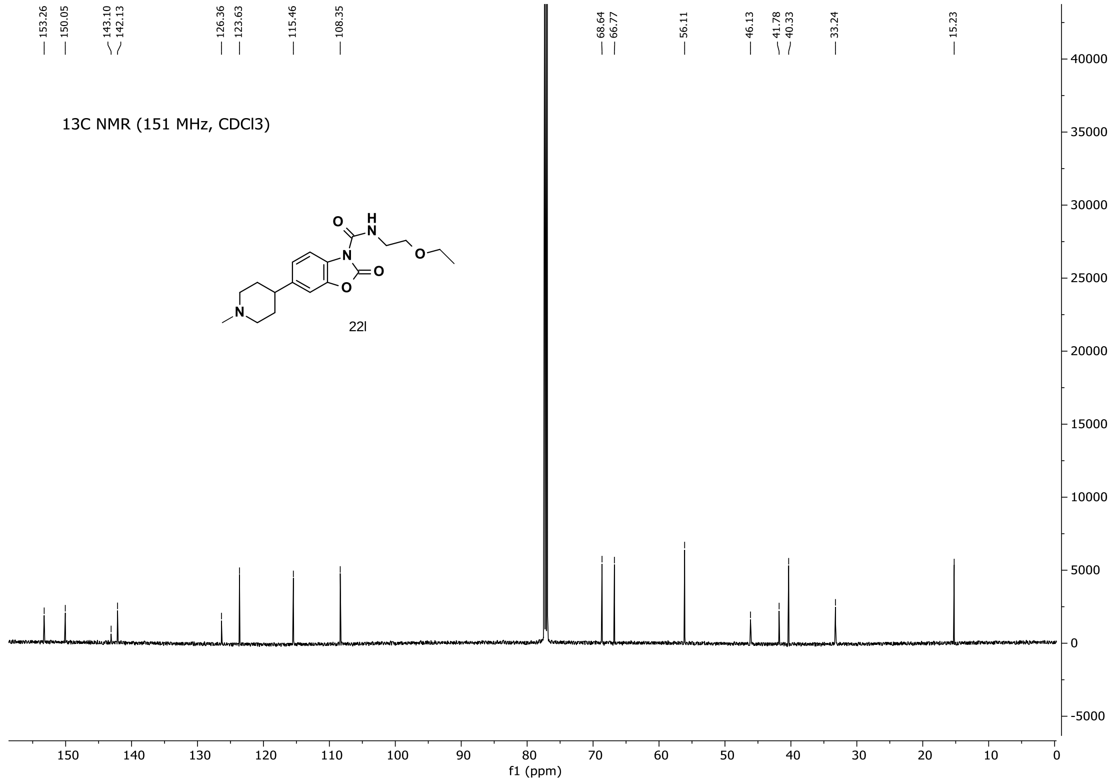
13C NMR (151 MHz, CDCl3)
22l
153.26
150.05
143.10
142.13
126.36
123.63
115.46
108.35
68.64
66.77
56.11
46.13
41.78
40.33
33.24
15.23
f1 (ppm)

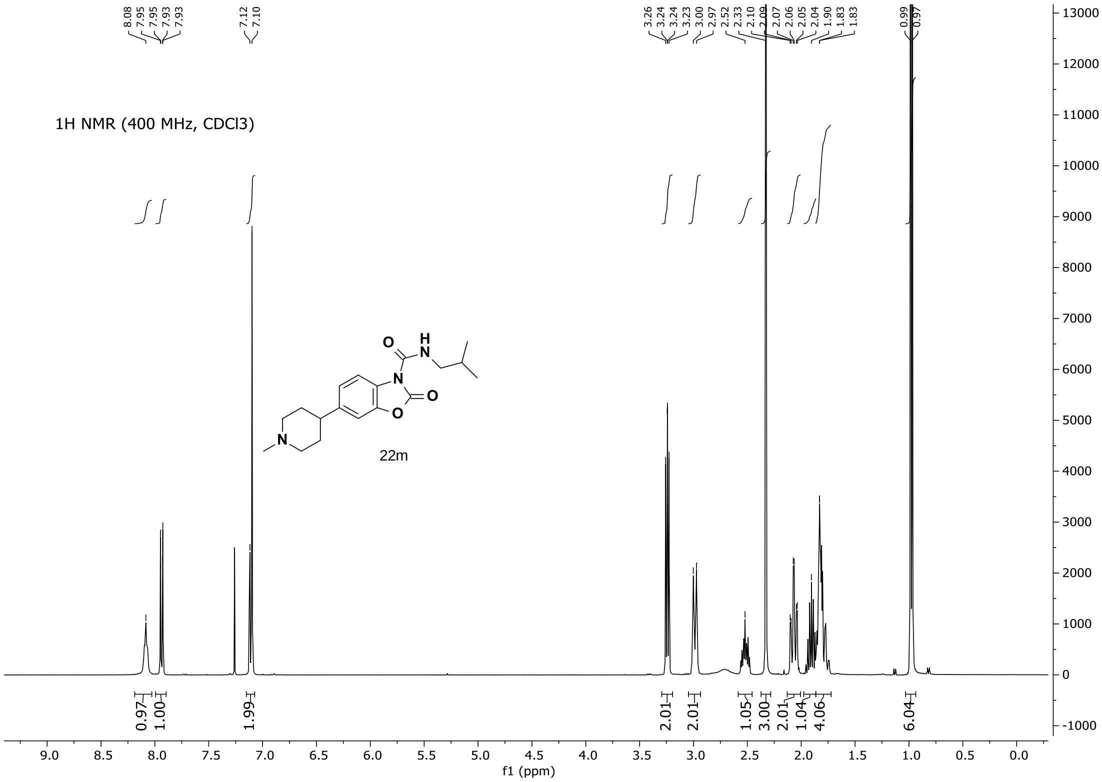
1H NMR (400 MHz, CDCl3)
22m
8.08
7.95
7.95
7.93
7.93
7.12
7.10
3.26
3.24
3.24
3.23
3.00
2.97
2.52
2.33
2.10
2.09
2.07
2.06
2.05
2.04
1.90
1.83
1.83
0.99
0.97
0.97
1.00
1.99
2.01
2.01
1.05
3.00
2.01
1.04
4.06
6.04
f1 (ppm)

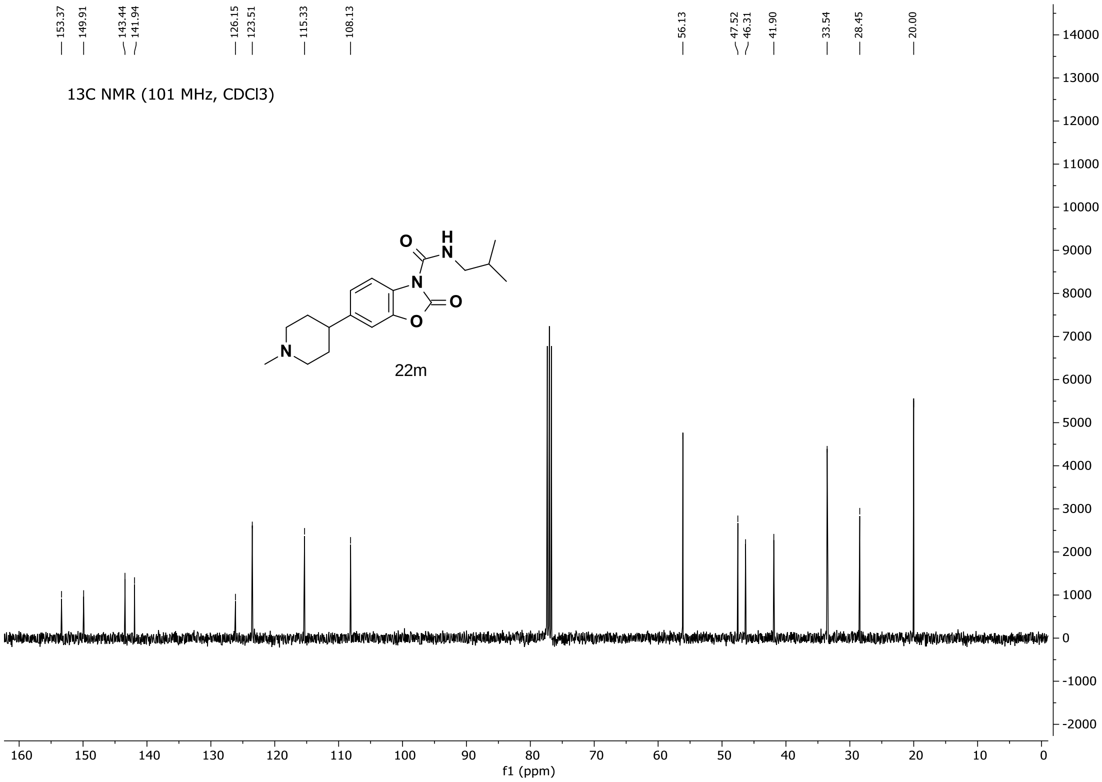
13C NMR (101 MHz, CDCl3)
22m
153.37
149.91
143.44
141.94
126.15
123.51
115.33
108.13
56.13
47.52
46.31
41.90
33.54
28.45
20.00
f1 (ppm)

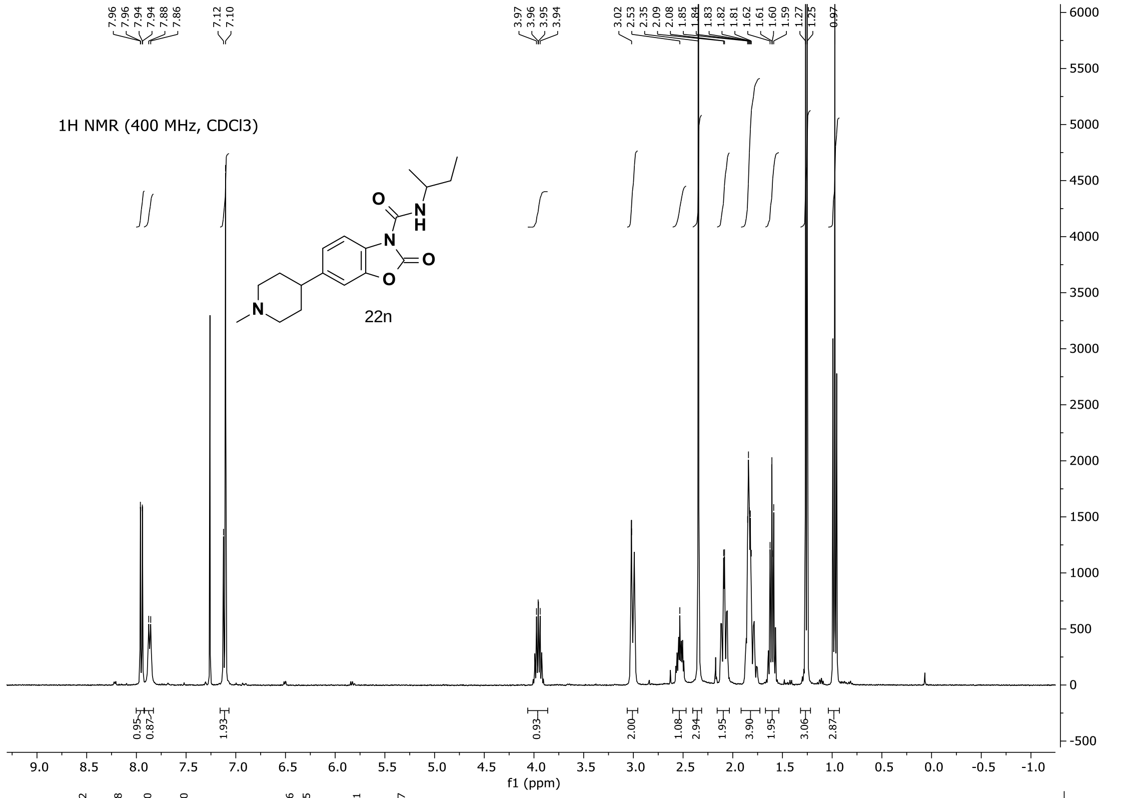
1H NMR (400 MHz, CDCl3)
22n
f1 (ppm)

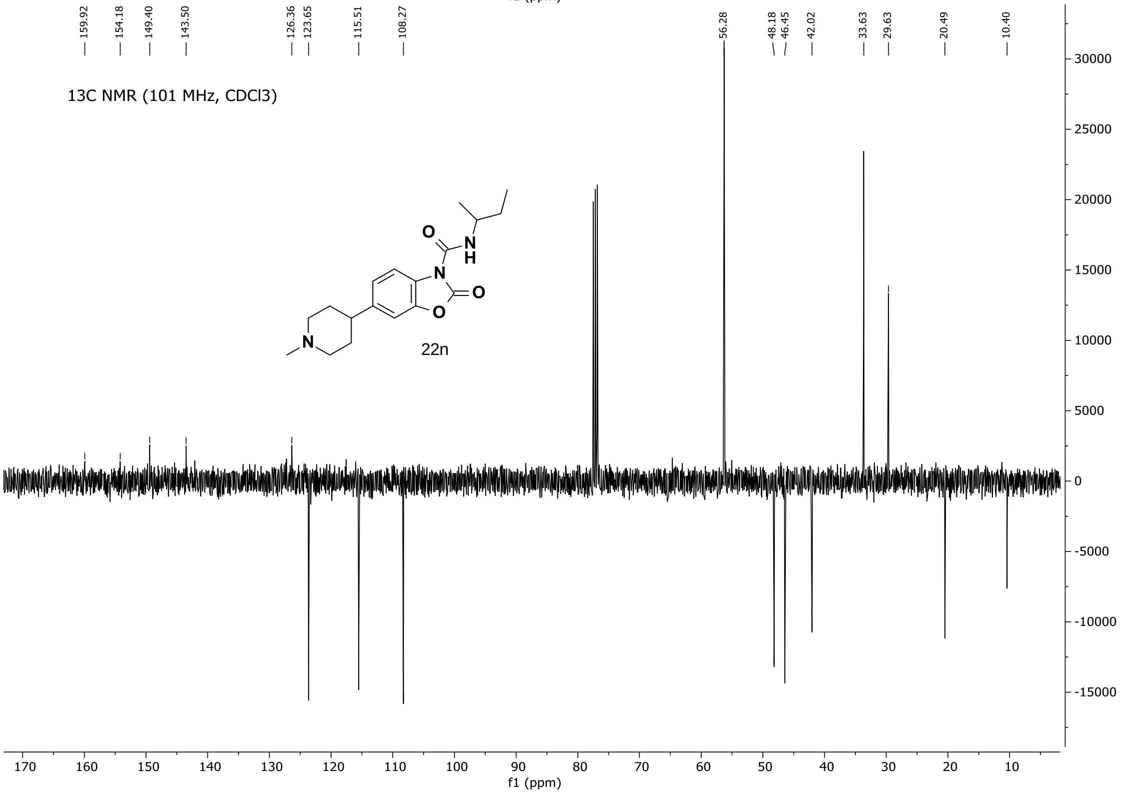
13C NMR (101 MHz, CDCl3)
22n
f1 (ppm)

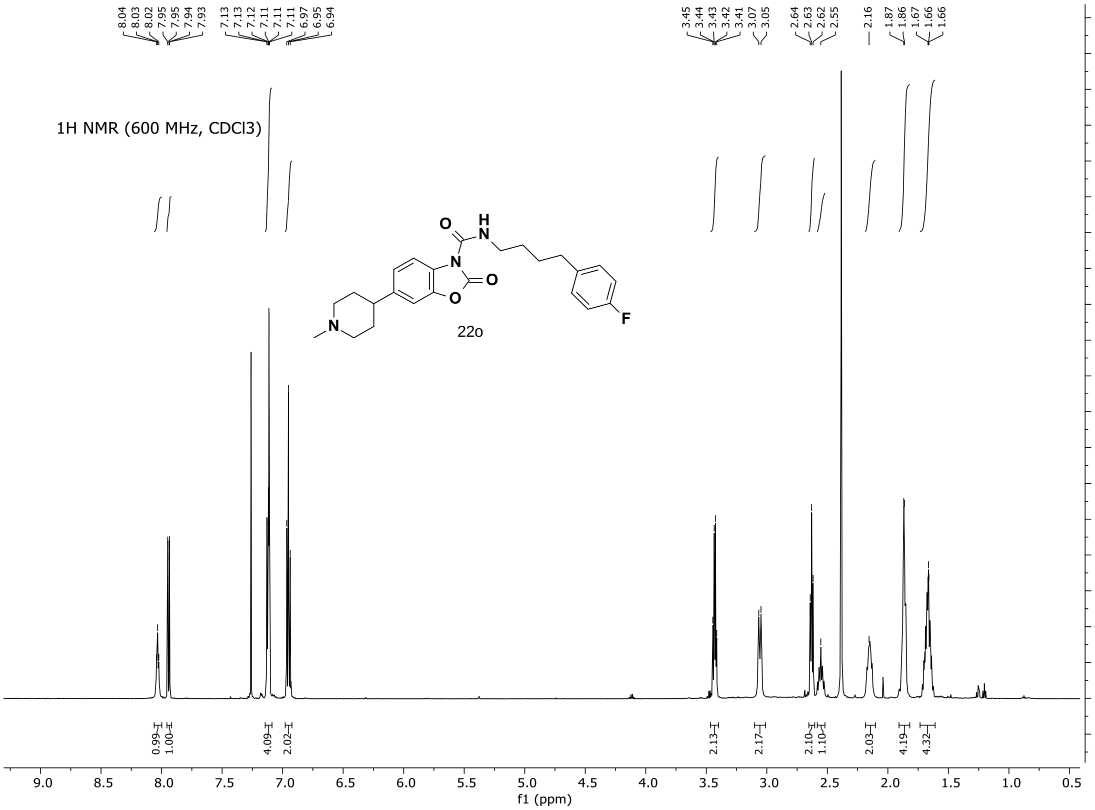

1H NMR (600 MHz, CDCl3)
8.04 8.03 8.02 7.95 7.95 7.94 7.93 7.13 7.13 7.12 7.11 7.11 7.11 6.97 6.95 6.94
3.45 3.44 3.43 3.42 3.41 3.07 3.05 2.64 2.63 2.62 2.55 2.16 1.87 1.86 1.67 1.66 1.66
0.99 1.00 4.09 2.02 2.13 2.17 2.10 1.10 2.03 4.19 4.32
O
H
N
N
O
O
N
F
22o
f1 (ppm)

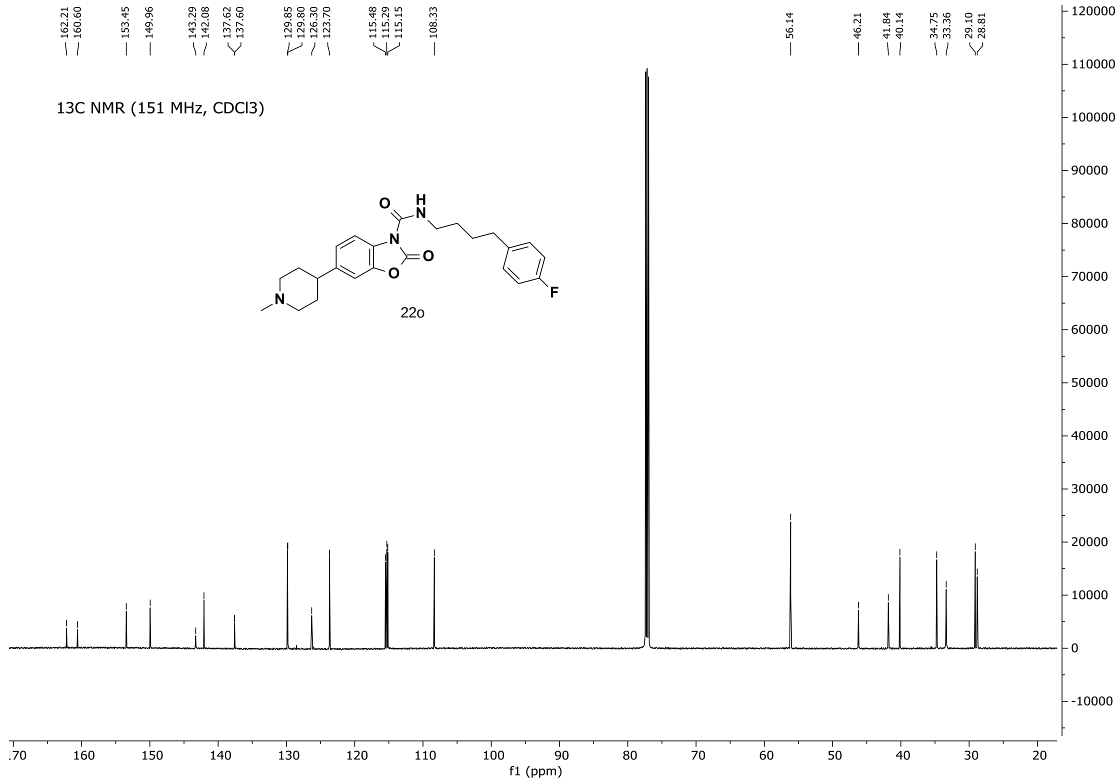

13C NMR (151 MHz, CDCl3)
162.21 160.60 153.45 149.96 143.29 142.08 137.62 137.60 129.85 129.80 126.30 123.70 115.48 115.29 115.15 108.33
56.14 46.21 41.84 40.14 34.75 33.36 29.10 28.81
O
H
N
N
O
O
N
F
22o
f1 (ppm)

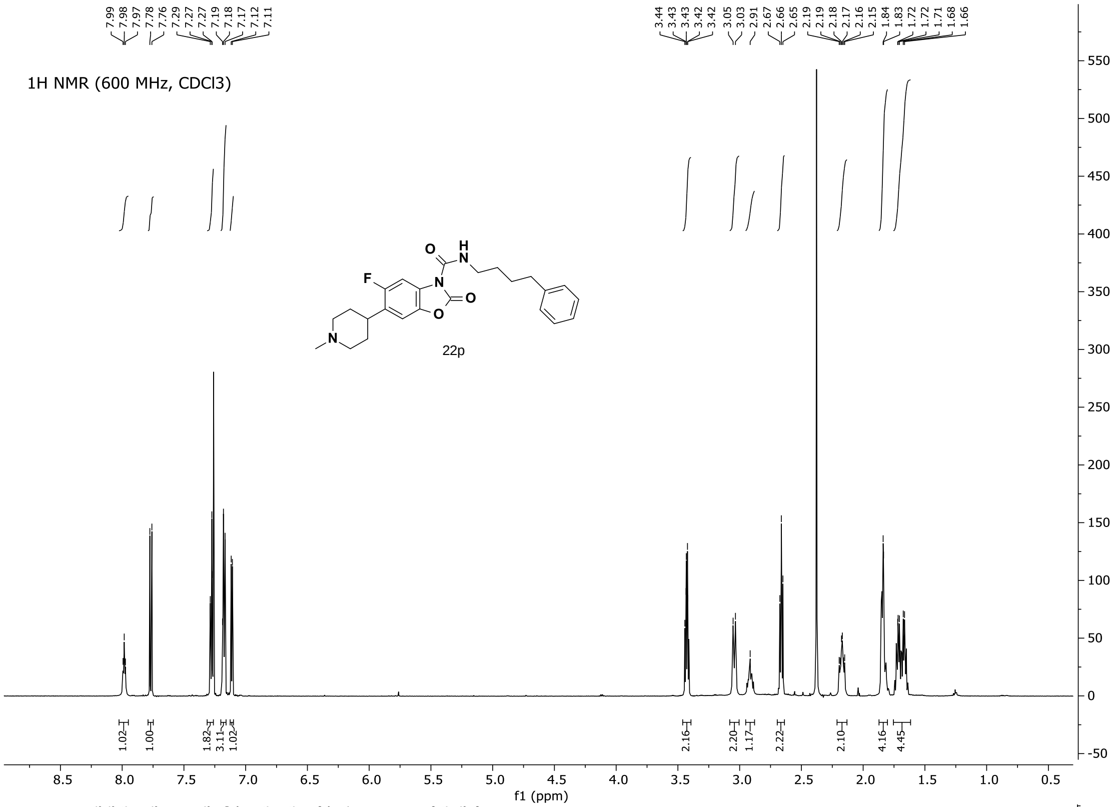
7.99 7.98 7.97 7.78 7.76 7.29 7.27 7.27 7.19 7.18 7.17 7.12 7.11
3.44 3.43 3.43 3.42 3.42 3.05 3.03 2.91 2.67 2.66 2.65 2.19 2.19 2.18 2.17 2.16 2.15 1.84 1.83 1.72 1.72 1.71 1.68 1.66
1H NMR (600 MHz, CDCl3)
22p
1.02 1.00 1.82 3.11 1.02 2.16 2.20 1.17 2.22 2.10 4.16 4.45
f1 (ppm)

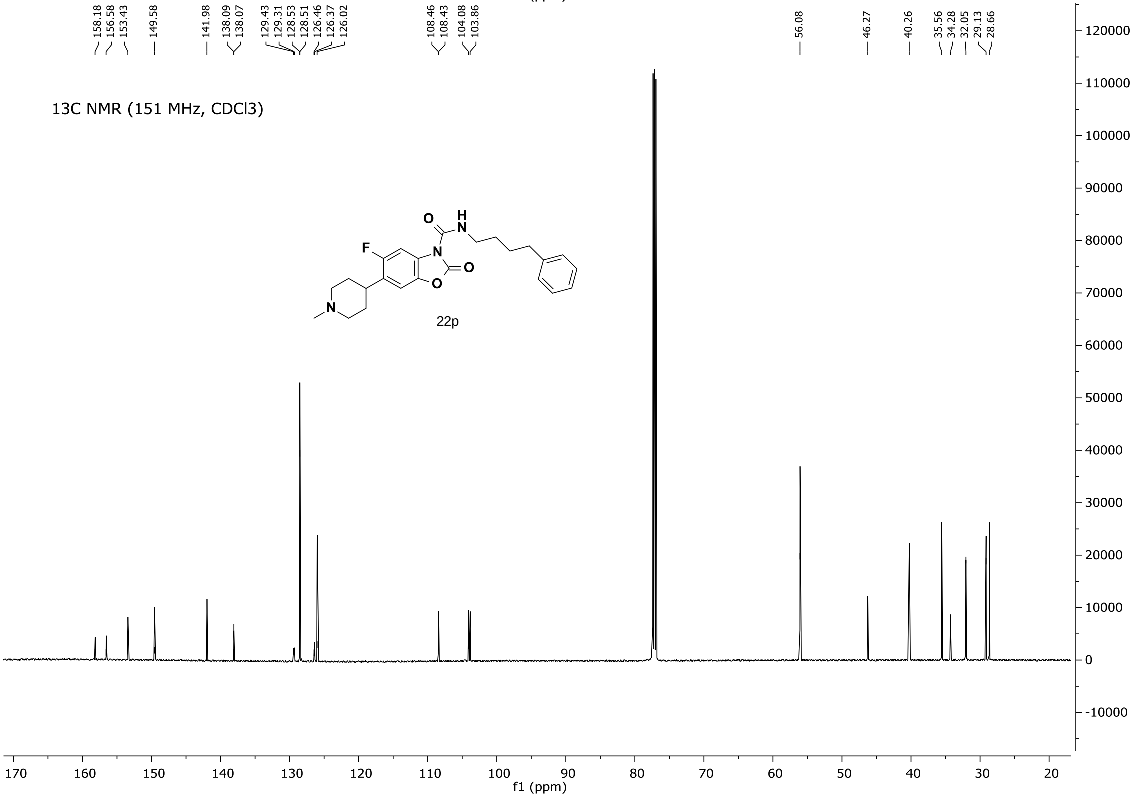
158.18 156.58 153.43 149.58 141.98 138.09 138.07 129.43 129.31 128.53 128.51 126.46 126.37 126.02 108.46 108.43 104.08 103.86 56.08 46.27 40.26 35.56 34.28 32.05 29.13 28.66
13C NMR (151 MHz, CDCl3)
22p
f1 (ppm)

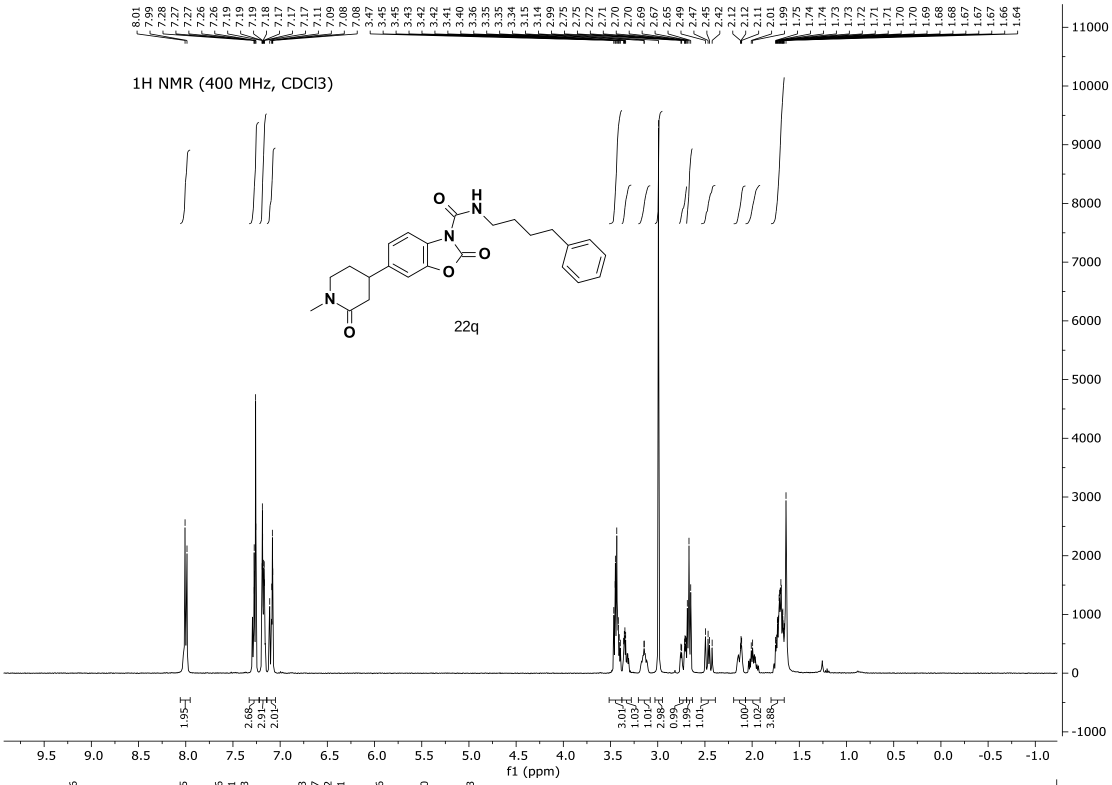
1H NMR (400 MHz, CDCl3)
22q
f1 (ppm)

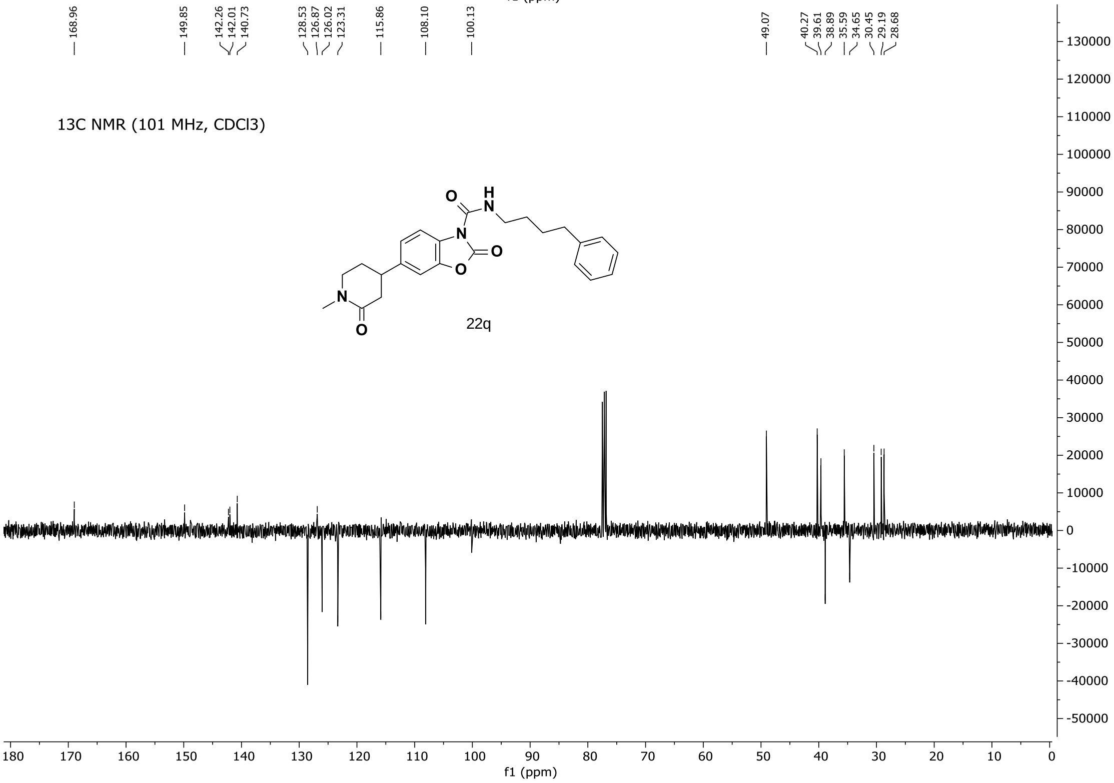
13C NMR (101 MHz, CDCl3)
22q
f1 (ppm)

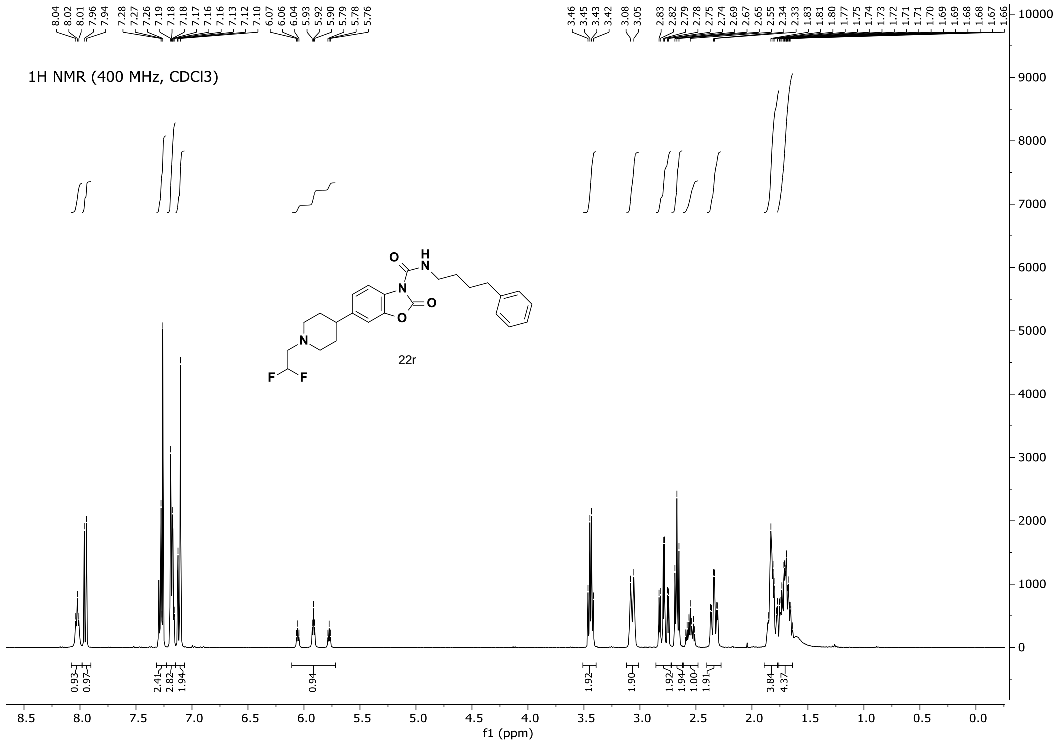
1H NMR (400 MHz, CDCl3)
8.04 8.02 8.01 7.96 7.94 7.28 7.27 7.26 7.19 7.18 7.18 7.17 7.16 7.16 7.13 7.12 7.10 6.07 6.06 6.04 5.93 5.92 5.90 5.79 5.78 5.76
3.46 3.45 3.43 3.42 3.08 3.05 2.83 2.82 2.79 2.78 2.75 2.74 2.69 2.67 2.65 2.55 2.34 2.33 1.83 1.81 1.80 1.77 1.75 1.74 1.73 1.72 1.71 1.71 1.70 1.69 1.69 1.68 1.67 1.66
0.93 0.97 2.41 2.82 1.94 0.94 1.92 1.90 1.92 1.94 1.00 1.91 3.84 4.37
22r
f1 (ppm)

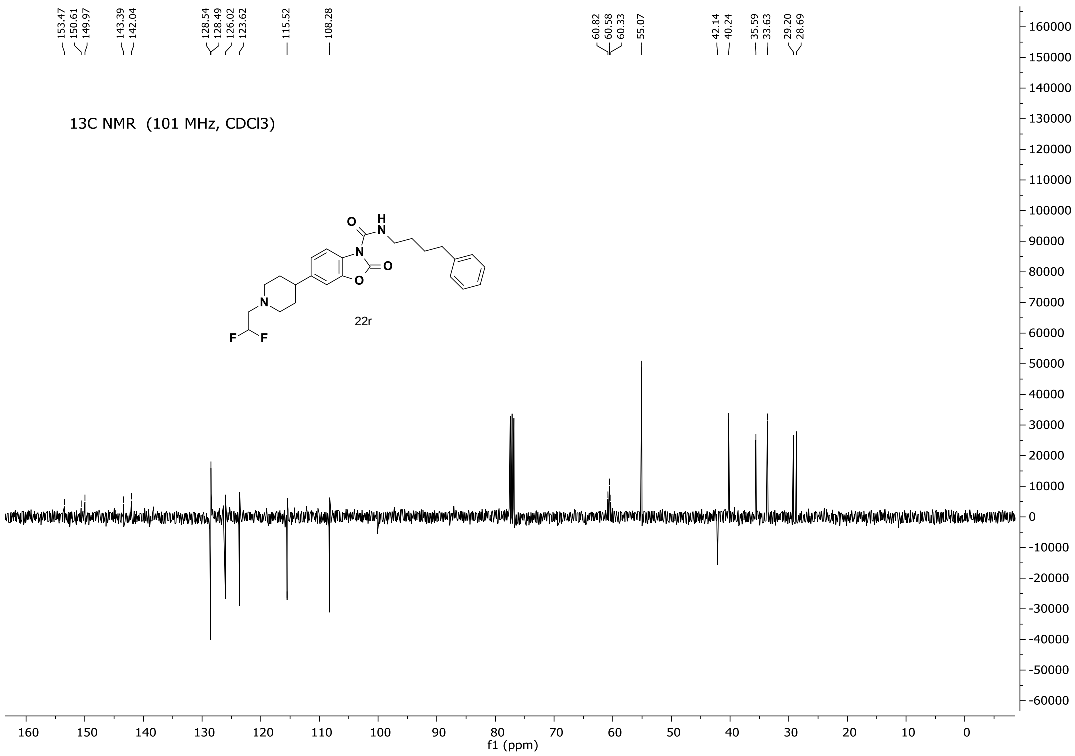
13C NMR (101 MHz, CDCl3)
153.47 150.61 149.97 143.39 142.04 128.54 128.49 126.02 123.62 115.52 108.28
60.82 60.58 60.33 55.07 42.14 40.24 35.59 33.63 29.20 28.69
22r
f1 (ppm)

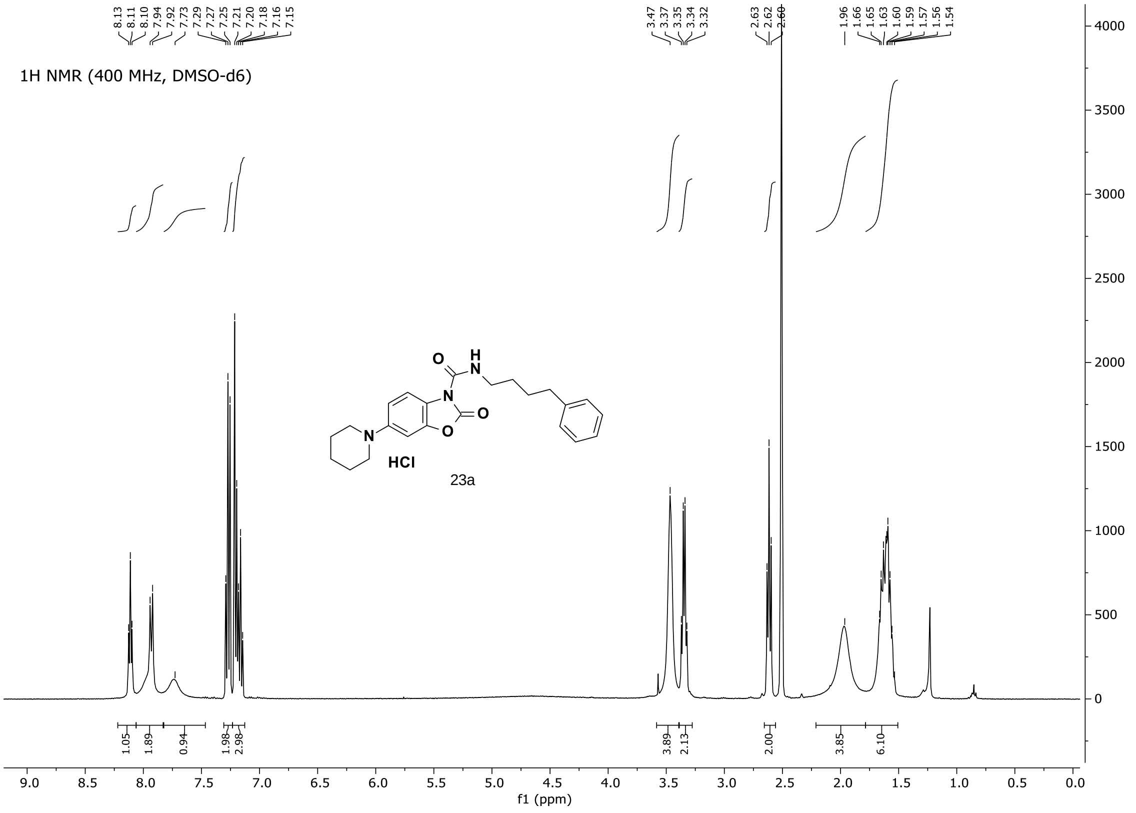

1H NMR (400 MHz, DMSO-d6)
HCl
23a
f1 (ppm)

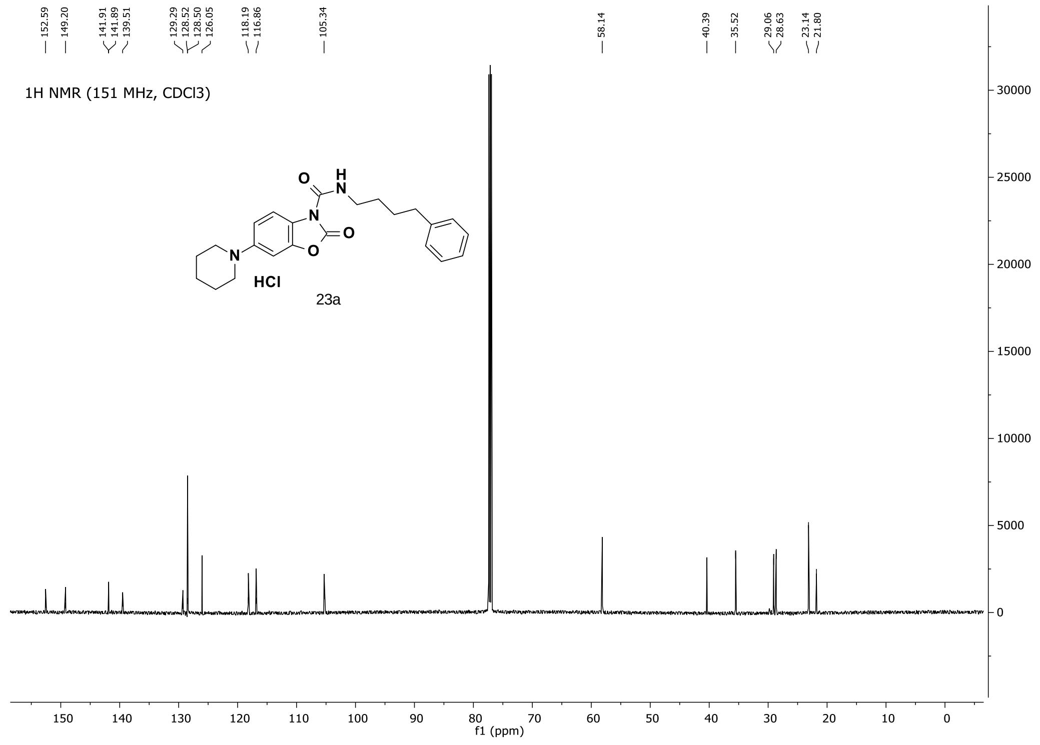

1H NMR (151 MHz, CDCl3)
HCl
23a
f1 (ppm)

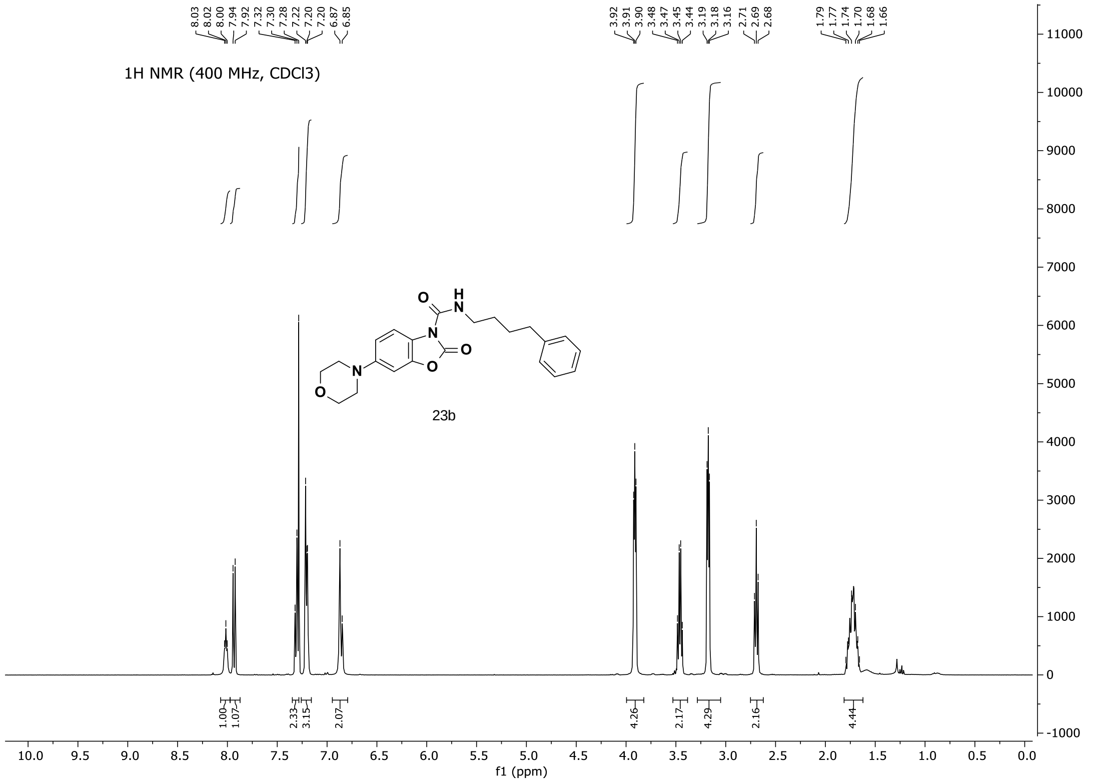
1H NMR (400 MHz, CDCl3)
8.03 8.02 8.00 7.94 7.92 7.32 7.30 7.28 7.22 7.20 7.20 6.87 6.85
3.92 3.91 3.90 3.48 3.47 3.45 3.44 3.19 3.18 3.16 2.71 2.69 2.68
1.79 1.77 1.74 1.70 1.68 1.66
1.00 1.07 2.33 3.15 2.07 4.26 2.17 4.29 2.16 4.44
23b
f1 (ppm)

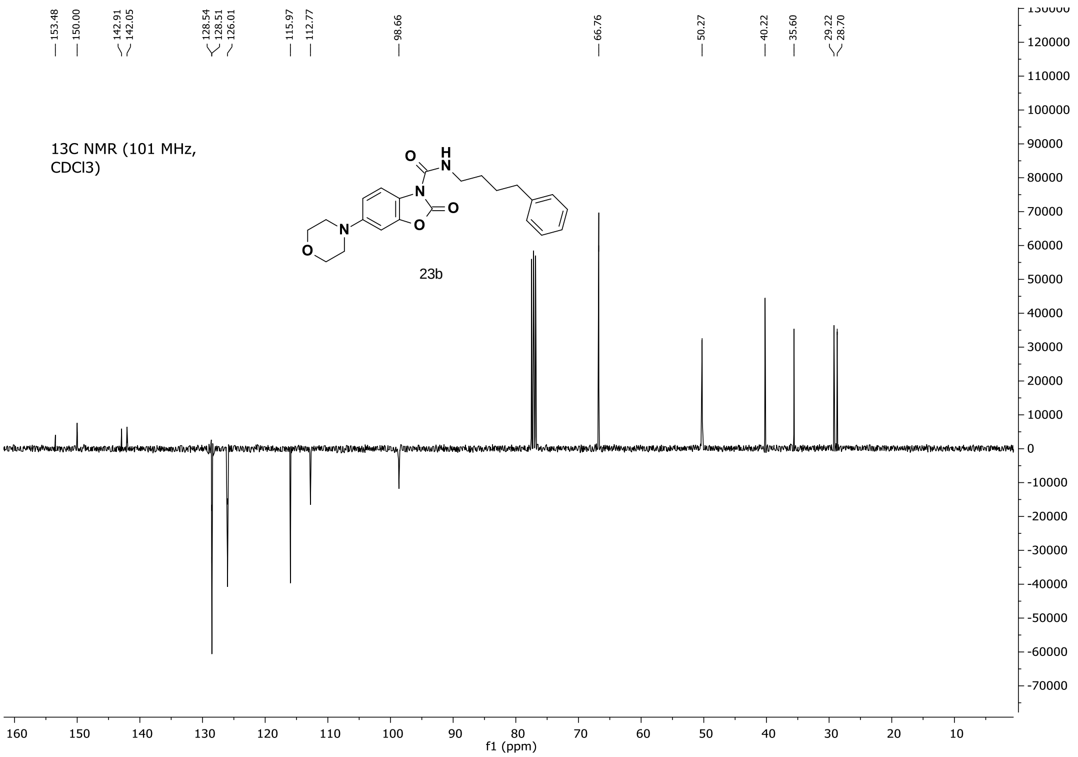
13C NMR (101 MHz, CDCl3)
153.48 150.00 142.91 142.05 128.54 128.51 126.01 115.97 112.77 98.66 66.76 50.27 40.22 35.60 29.22 28.70
23b
f1 (ppm)

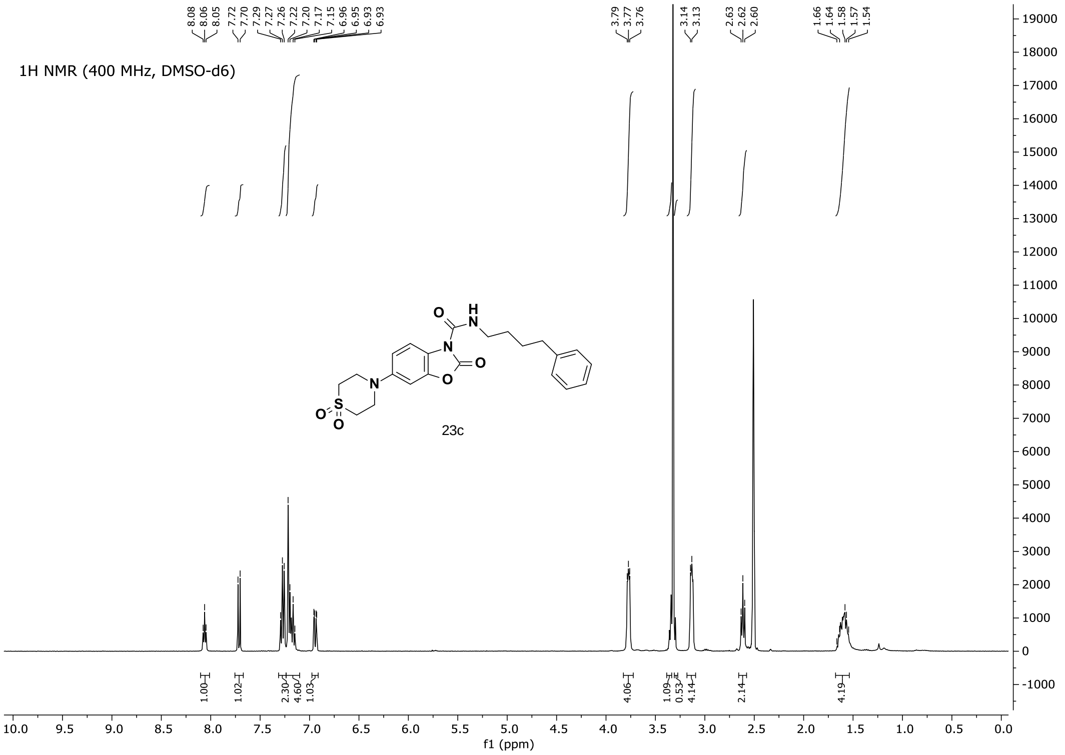
1H NMR (400 MHz, DMSO-d6)
8.08 8.06 8.05 7.72 7.70 7.29 7.27 7.26 7.22 7.20 7.17 7.15 6.96 6.95 6.93 6.93
3.79 3.77 3.76 3.14 3.13 2.63 2.62 2.60 1.66 1.64 1.58 1.57 1.54
1.00 1.02 2.30 4.60 1.03 4.06 1.09 0.53 4.14 2.14 4.19
23c
f1 (ppm)

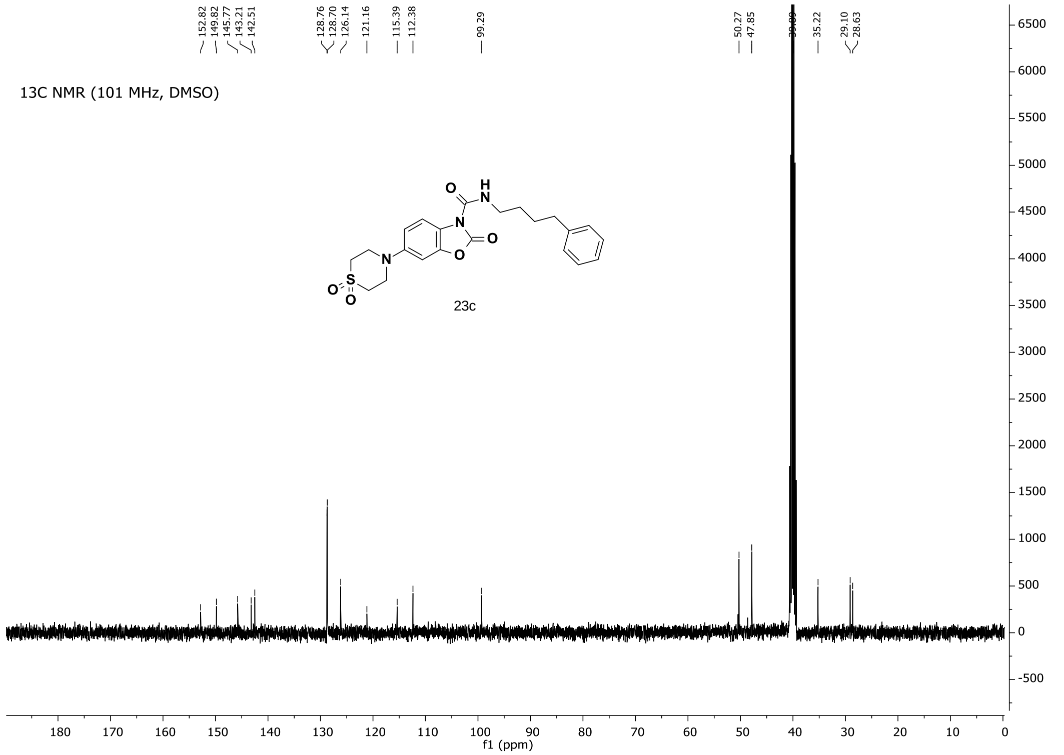
13C NMR (101 MHz, DMSO)
152.82 149.82 145.77 143.21 142.51 128.76 128.70 126.14 121.16 115.39 112.38 99.29
50.27 47.85 35.22 29.10 28.63
23c
f1 (ppm)

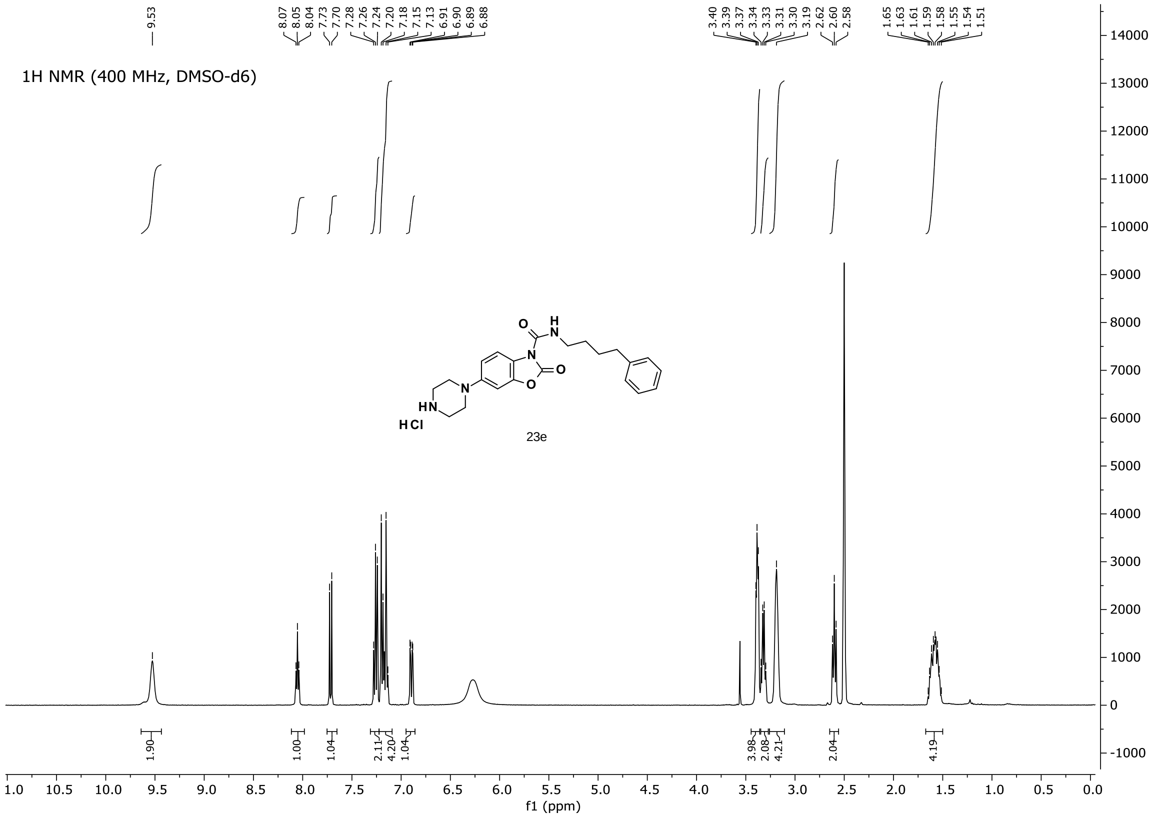
1H NMR (400 MHz, DMSO-d6)
9.53
8.07 8.05 8.04 7.73 7.70 7.28 7.26 7.24 7.20 7.18 7.15 7.13 6.91 6.90 6.89 6.88
3.40 3.39 3.37 3.34 3.33 3.31 3.30 3.19 2.62 2.60 2.58
1.65 1.63 1.61 1.59 1.58 1.55 1.54 1.51
1.90 1.00 1.04 2.11 4.20 1.04 3.98 2.08 4.21 2.04 4.19
HCl
23e
f1 (ppm)

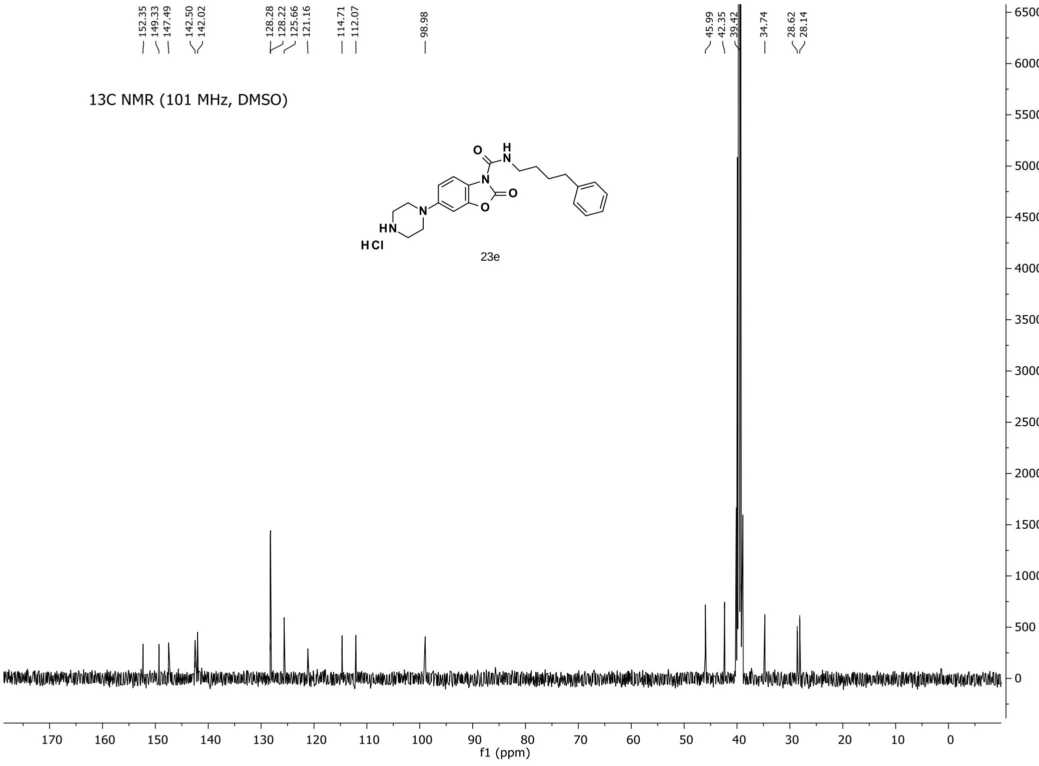
13C NMR (101 MHz, DMSO)
152.35 149.33 147.49 142.50 142.02
128.28 128.22 125.66 121.16 114.71 112.07 98.98
45.99 42.35 39.42 34.74 28.62 28.14
HCl
23e
f1 (ppm)

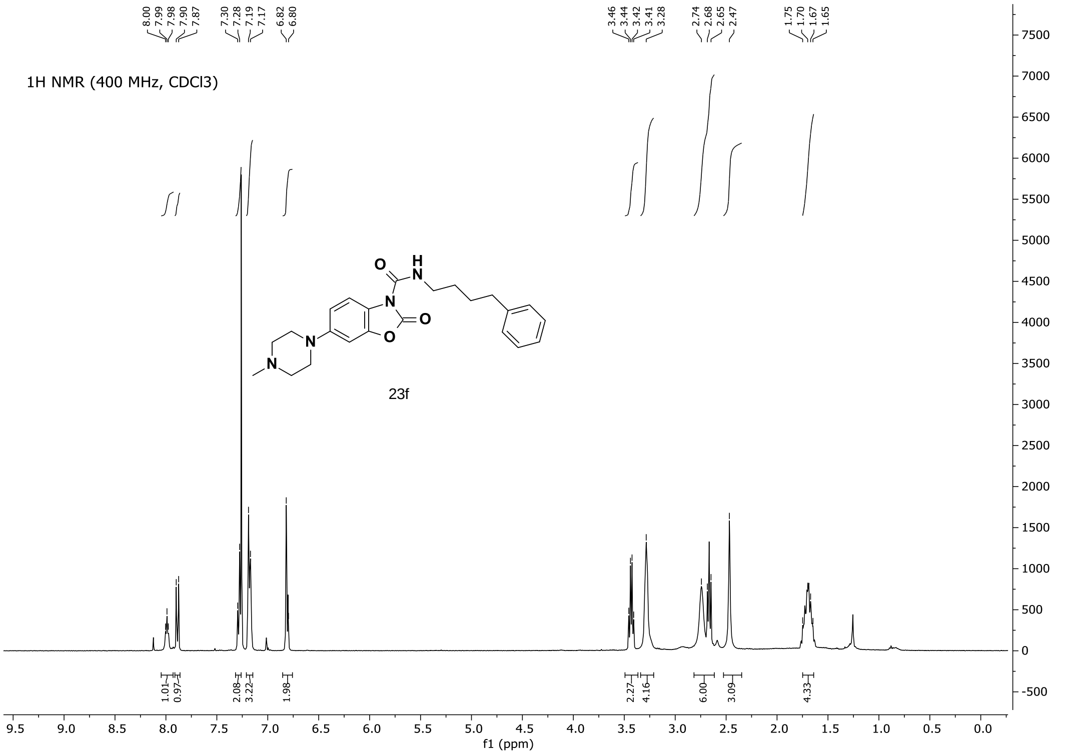
1H NMR (400 MHz, CDCl3)
8.00 7.99 7.98 7.90 7.87 7.30 7.28 7.19 7.17 6.82 6.80
3.46 3.44 3.42 3.41 3.28 2.74 2.68 2.65 2.47 1.75 1.70 1.67 1.65
1.01 0.97 2.08 3.22 1.98 2.27 4.16 6.00 3.09 4.33
23f
f1 (ppm)

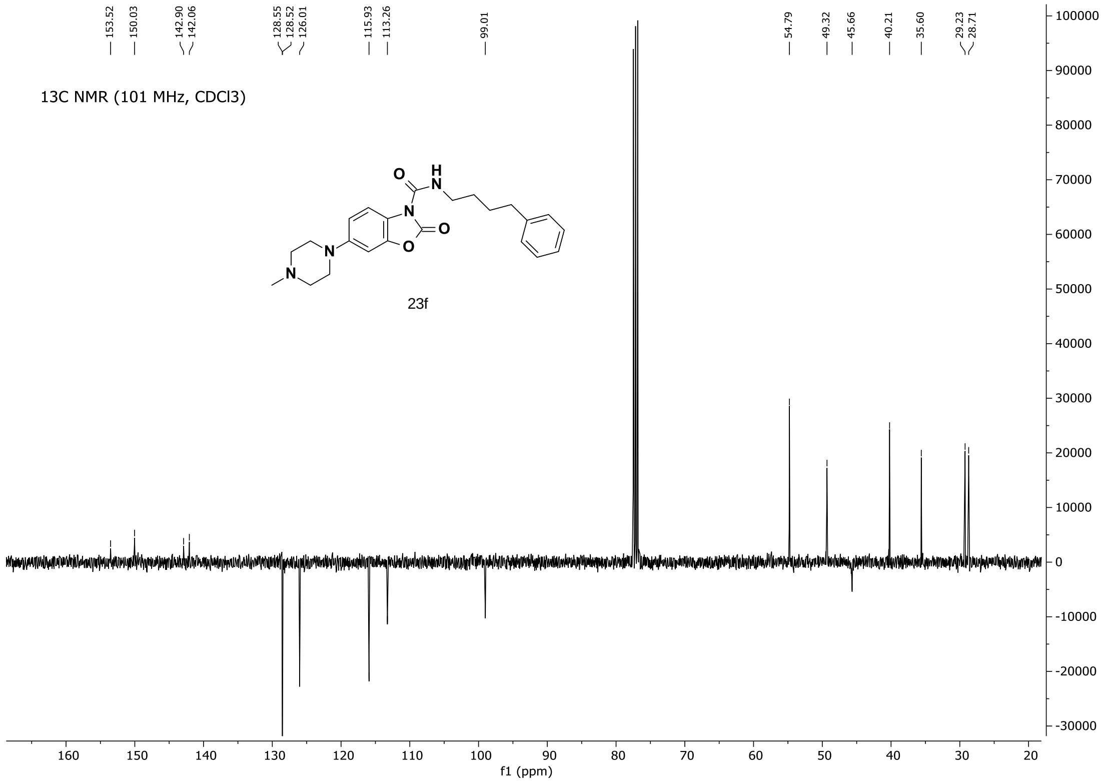
13C NMR (101 MHz, CDCl3)
153.52 150.03 142.90 142.06 128.55 128.52 126.01 115.93 113.26 99.01
54.79 49.32 45.66 40.21 35.60 29.23 28.71
23f
f1 (ppm)

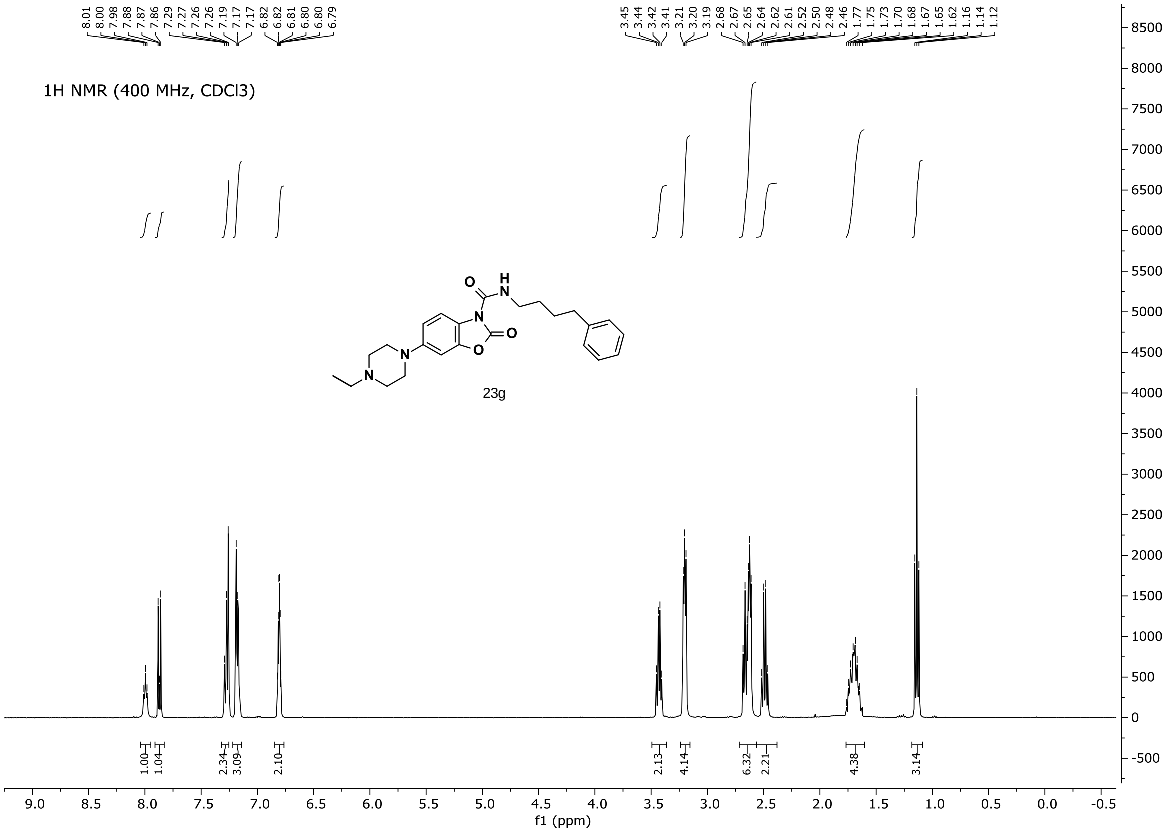
1H NMR (400 MHz, CDCl3)
23g
f1 (ppm)

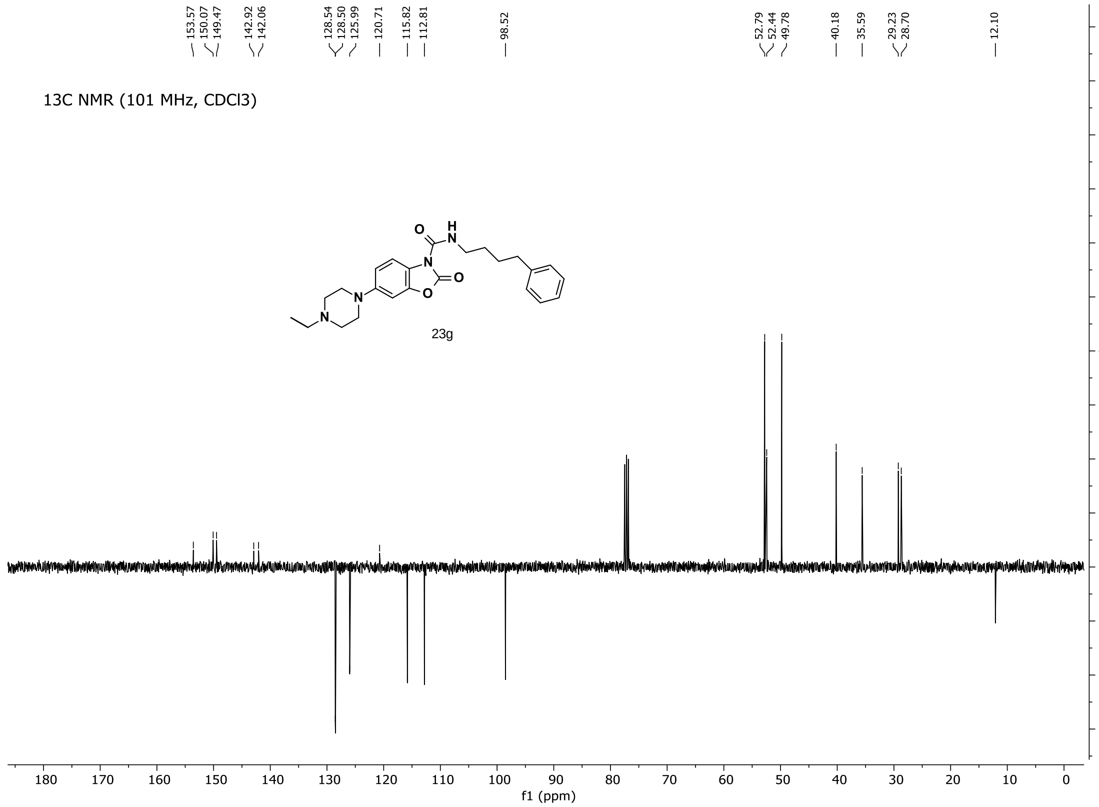
13C NMR (101 MHz, CDCl3)
23g
f1 (ppm)

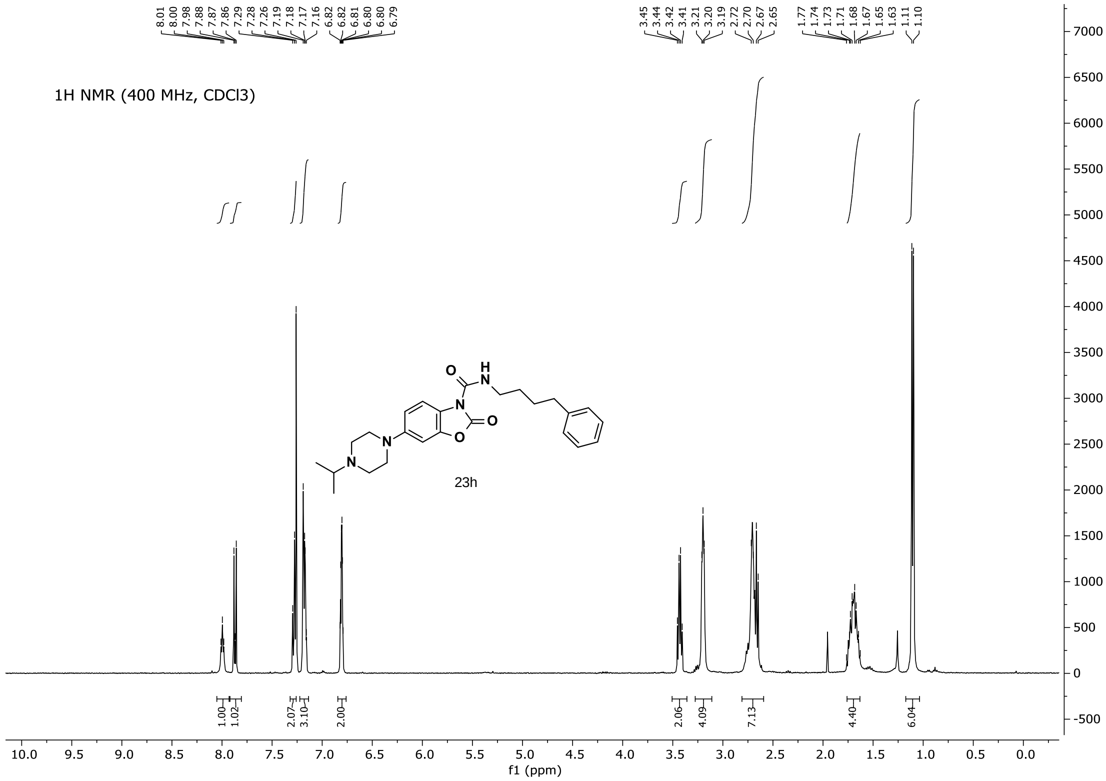
1H NMR (400 MHz, CDCl3)
8.01 8.00 7.98 7.88 7.87 7.86 7.29 7.28 7.26 7.19 7.18 7.17 7.16 6.82 6.82 6.81 6.80 6.80 6.79
3.45 3.44 3.42 3.41 3.21 3.20 3.19 2.72 2.70 2.67 2.65
1.77 1.74 1.73 1.71 1.68 1.67 1.65 1.63 1.11 1.10
1.00 1.02 2.07 3.10 2.00 2.06 4.09 7.13 4.40 6.04
23h
f1 (ppm)

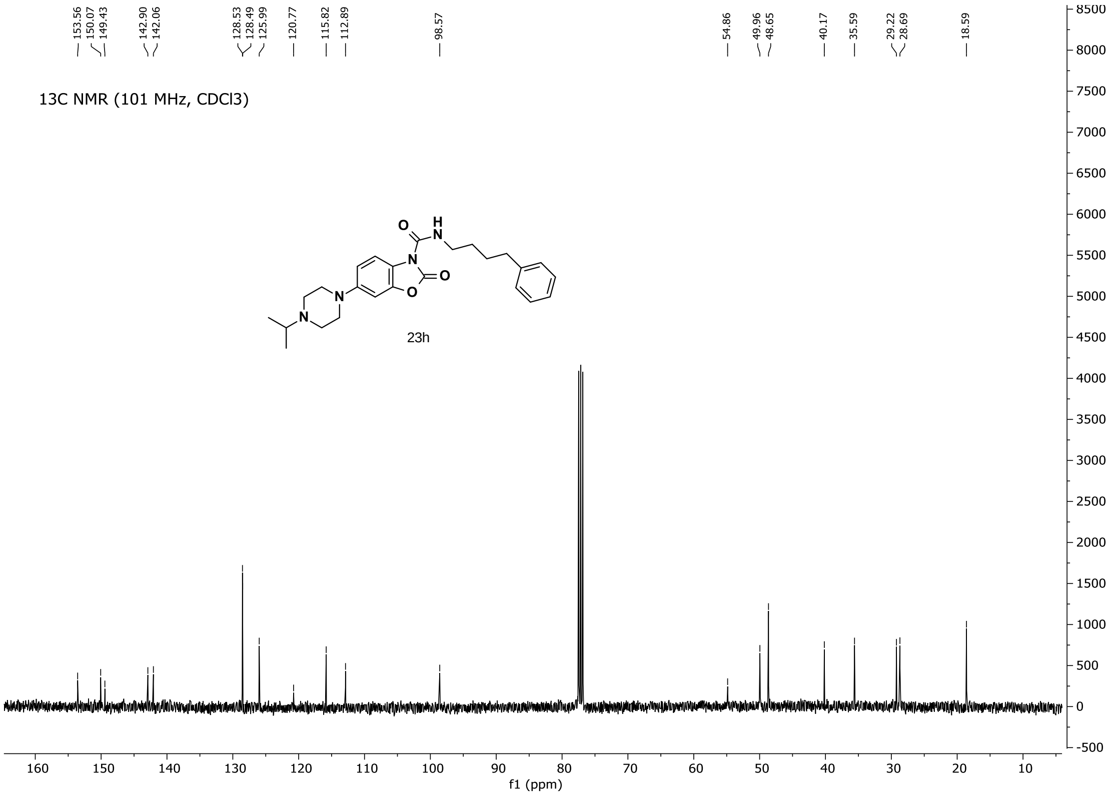
13C NMR (101 MHz, CDCl3)
153.56 150.07 149.43 142.90 142.06 128.53 128.49 125.99 120.77 115.82 112.89 98.57
54.86 49.96 48.65 40.17 35.59 29.22 28.69 18.59
23h
f1 (ppm)

1H NMR (400 MHz, CDCl3)

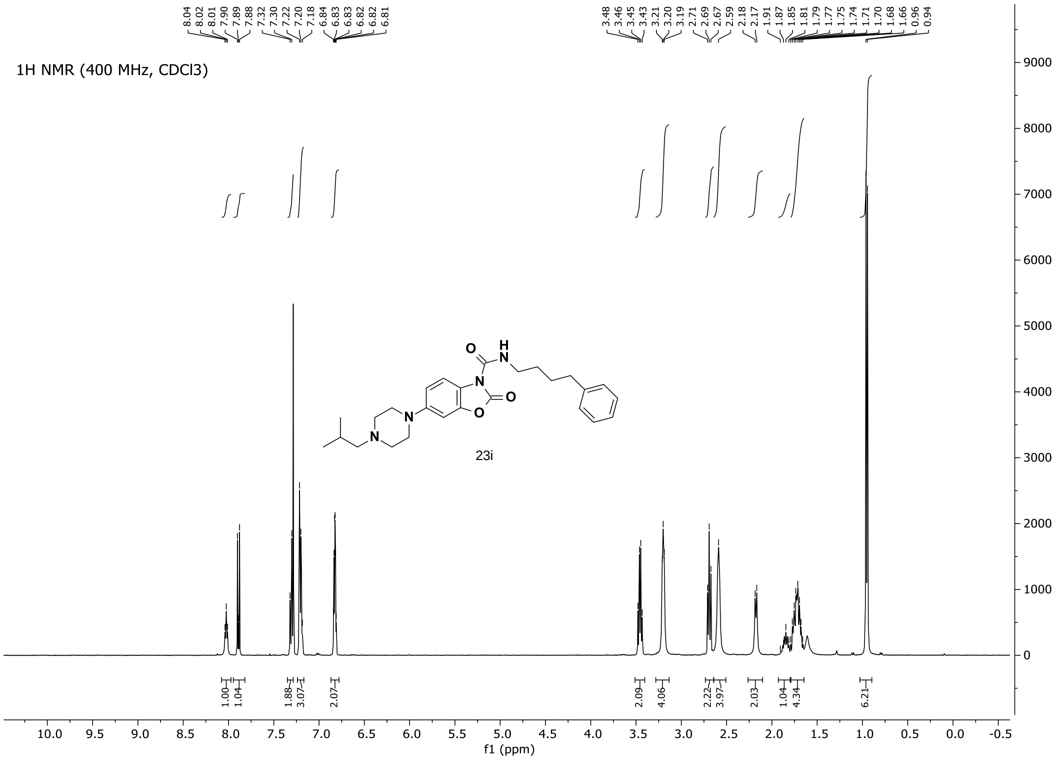

13C NMR (101 MHz, CDCl3)

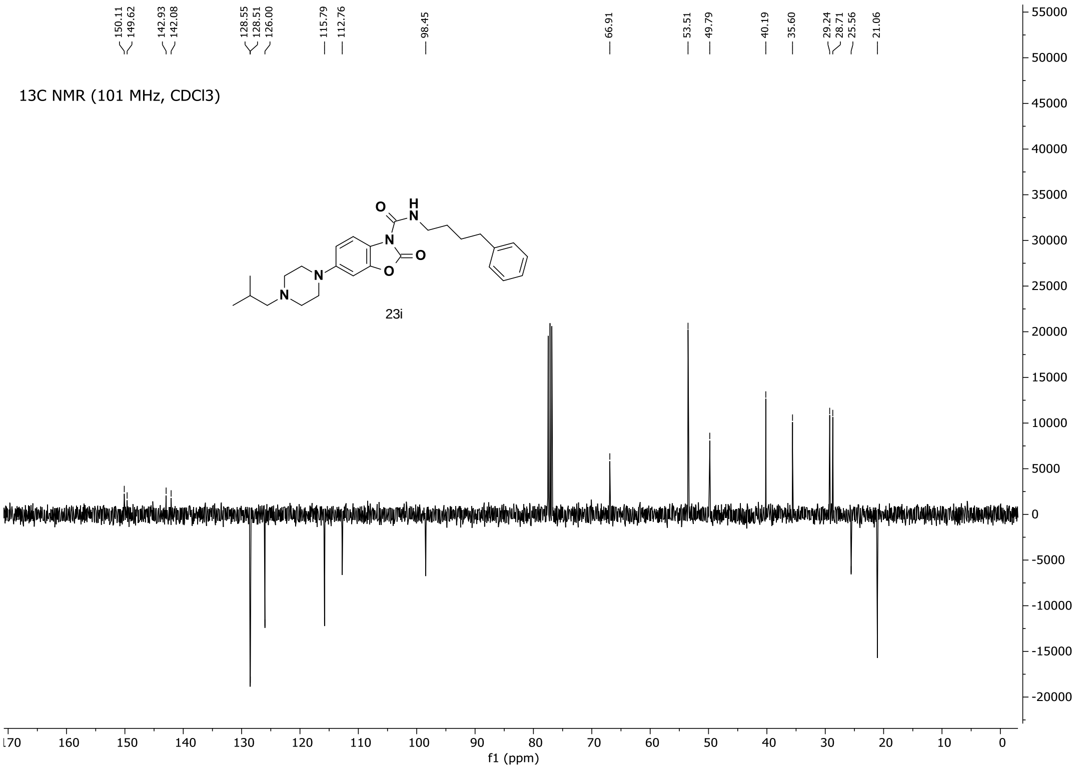

1H NMR (400 MHz, CDCl3)

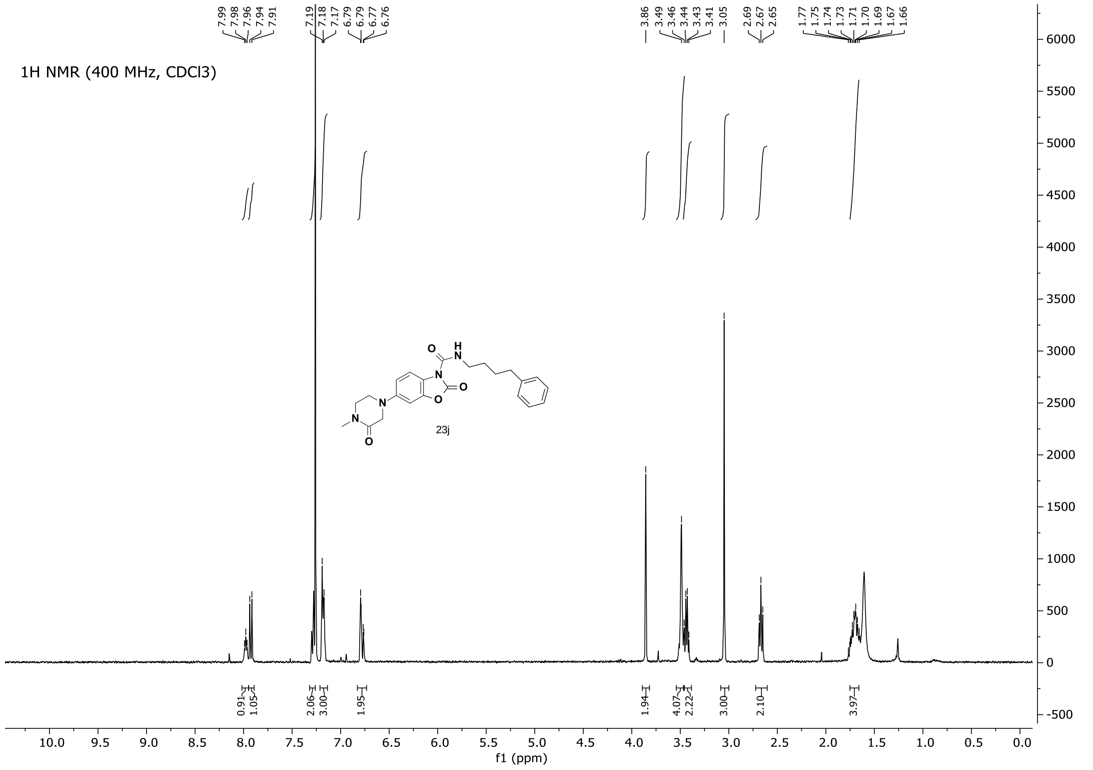

13C NMR (101 MHz, CDCl3)

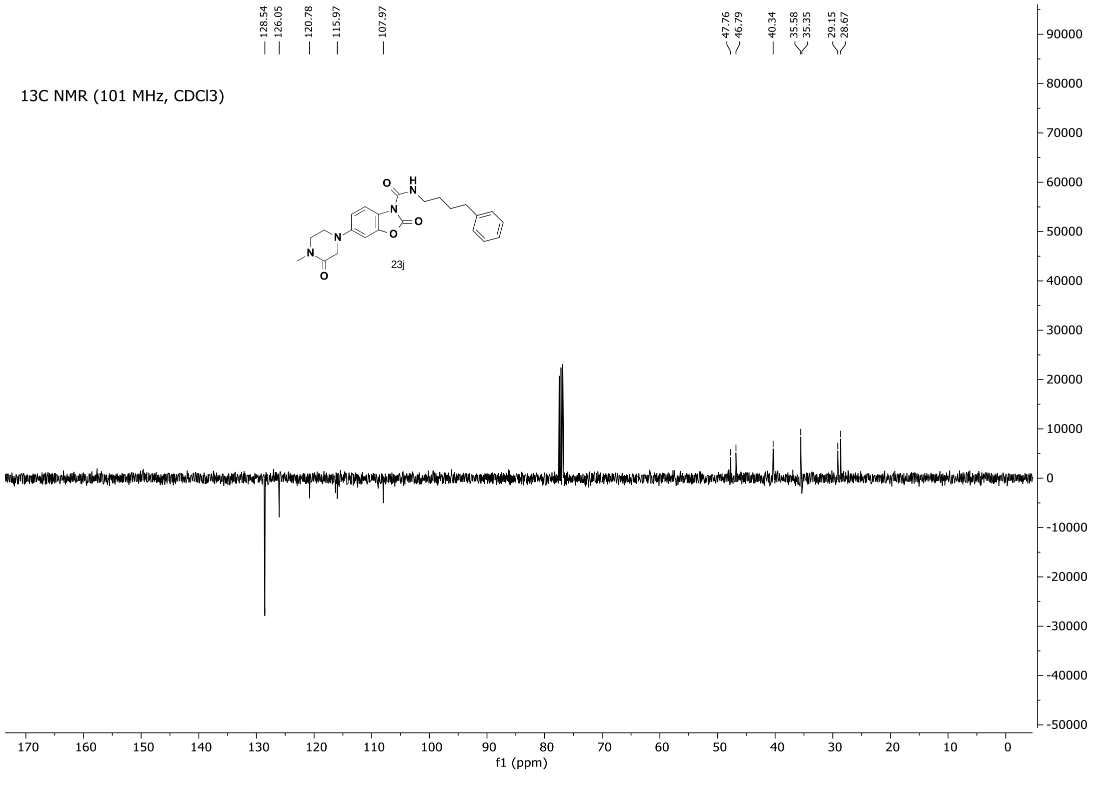

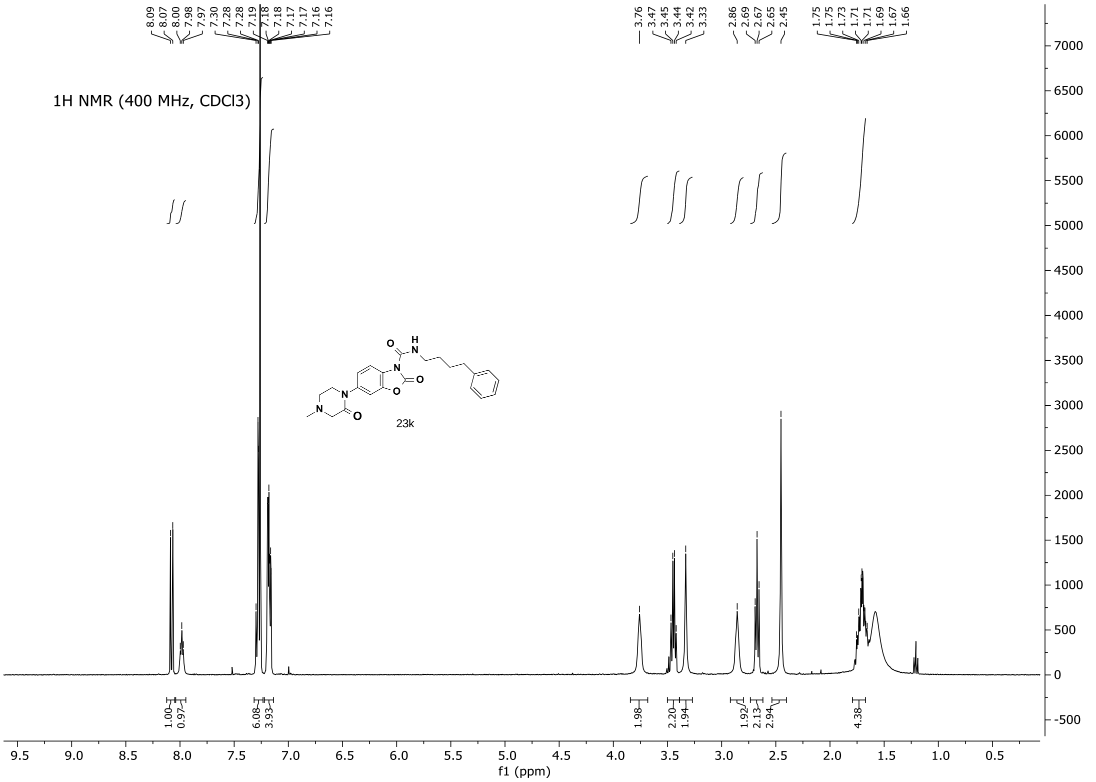
1H NMR (400 MHz, CDCl3)
23k
f1 (ppm)

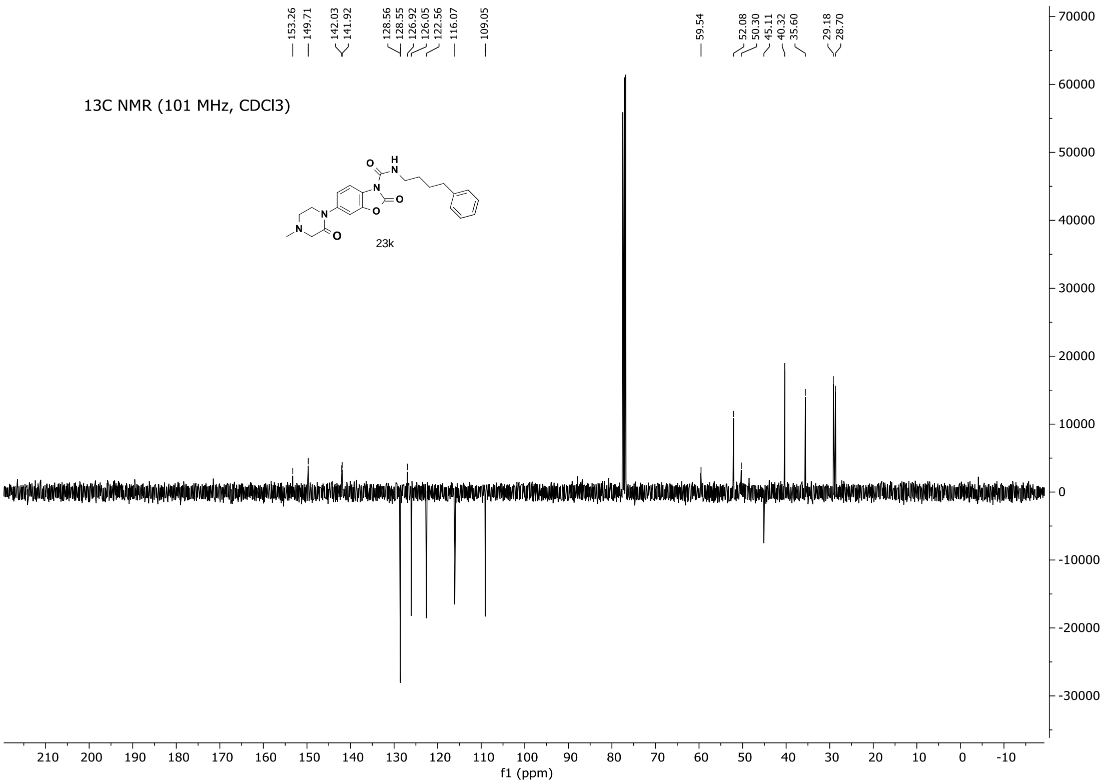
13C NMR (101 MHz, CDCl3)
23k
f1 (ppm)

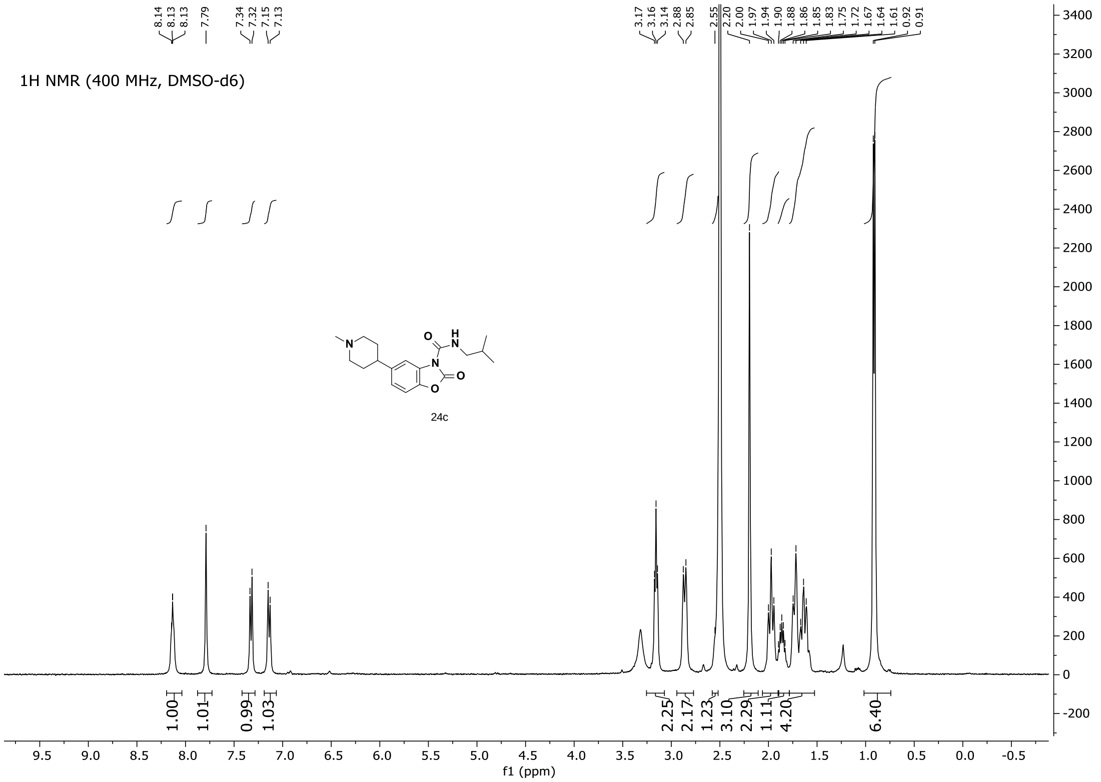
1H NMR (400 MHz, DMSO-d6)
8.14
8.13
8.13
7.79
7.34
7.32
7.15
7.13
3.17
3.16
3.14
2.88
2.85
2.55
2.20
2.00
1.97
1.94
1.90
1.88
1.86
1.85
1.83
1.75
1.72
1.67
1.64
1.61
0.92
0.91
1.00
1.01
0.99
1.03
2.25
2.17
1.23
3.10
2.29
1.11
4.20
6.40
24c
f1 (ppm)

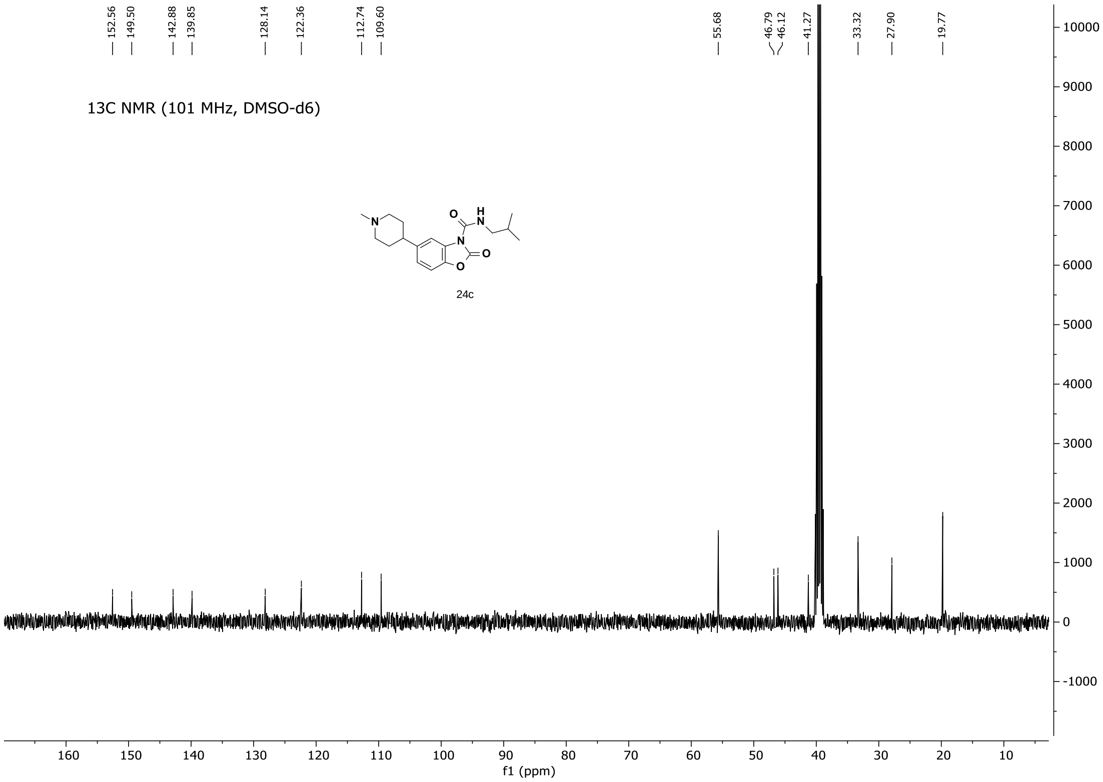
13C NMR (101 MHz, DMSO-d6)
152.56
149.50
142.88
139.85
128.14
122.36
112.74
109.60
55.68
46.79
46.12
41.27
33.32
27.90
19.77
24c
f1 (ppm)

1H NMR (400 MHz, CDCl3)

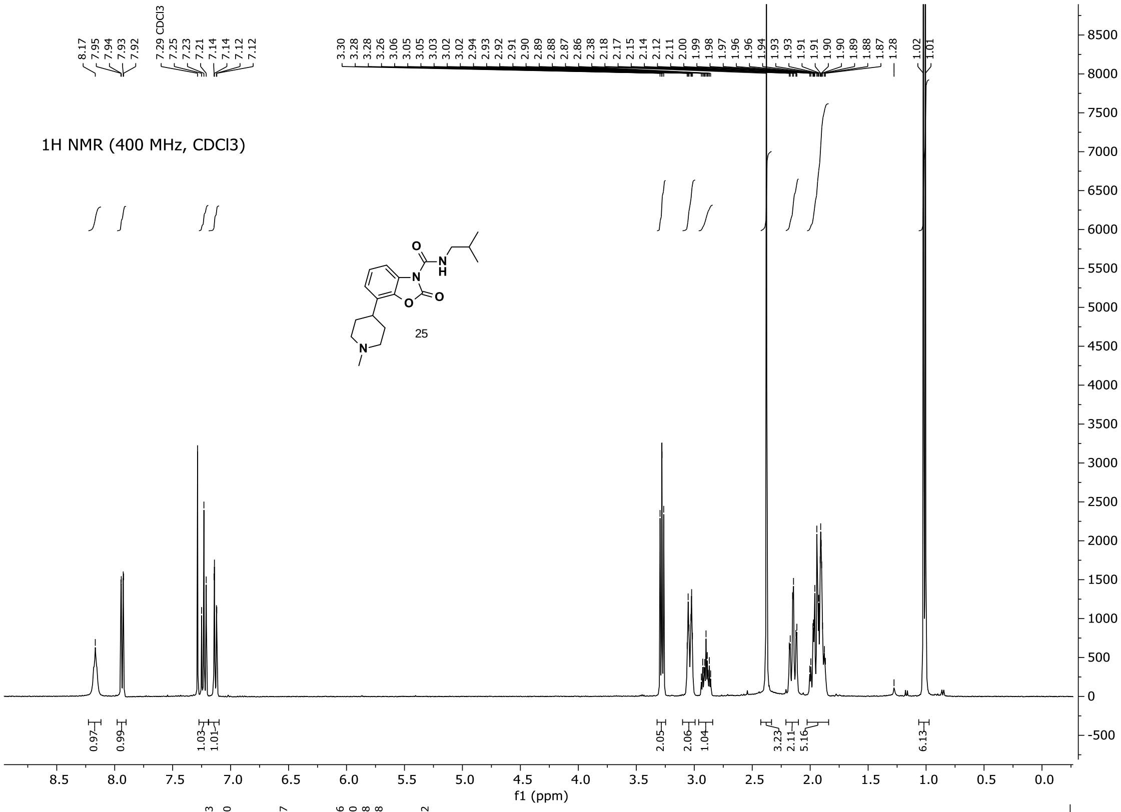

13C NMR (101 MHz, CDCl3)

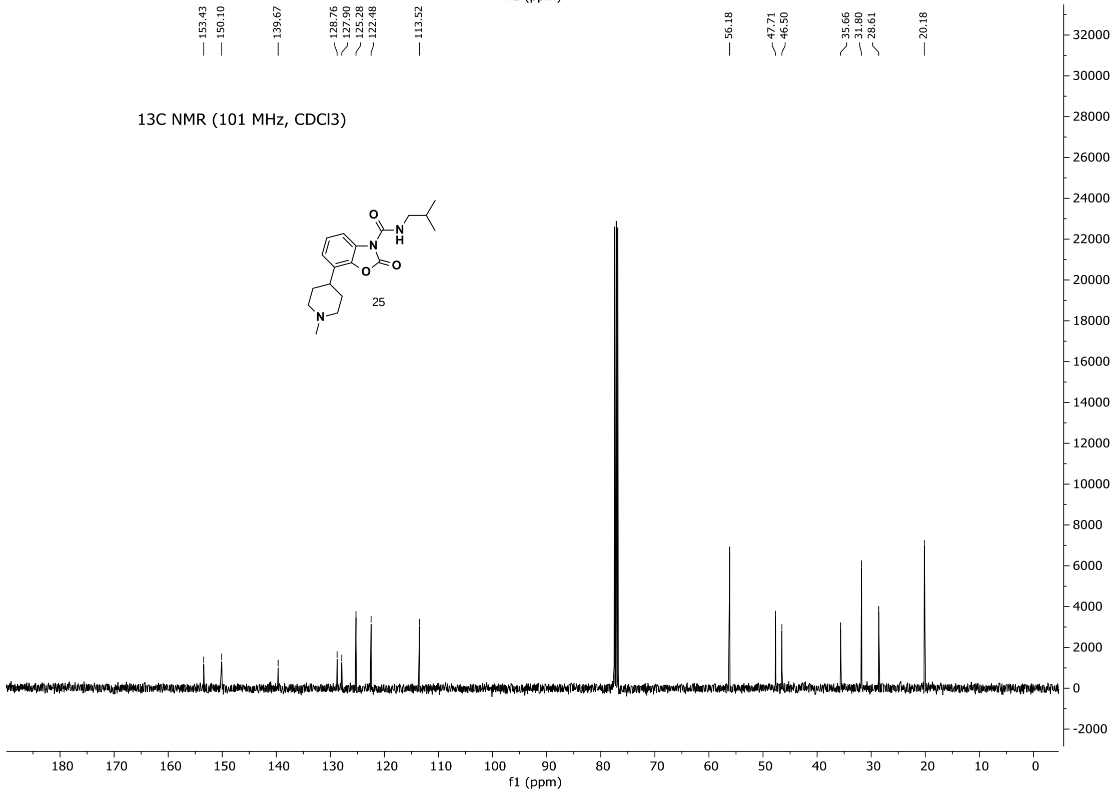

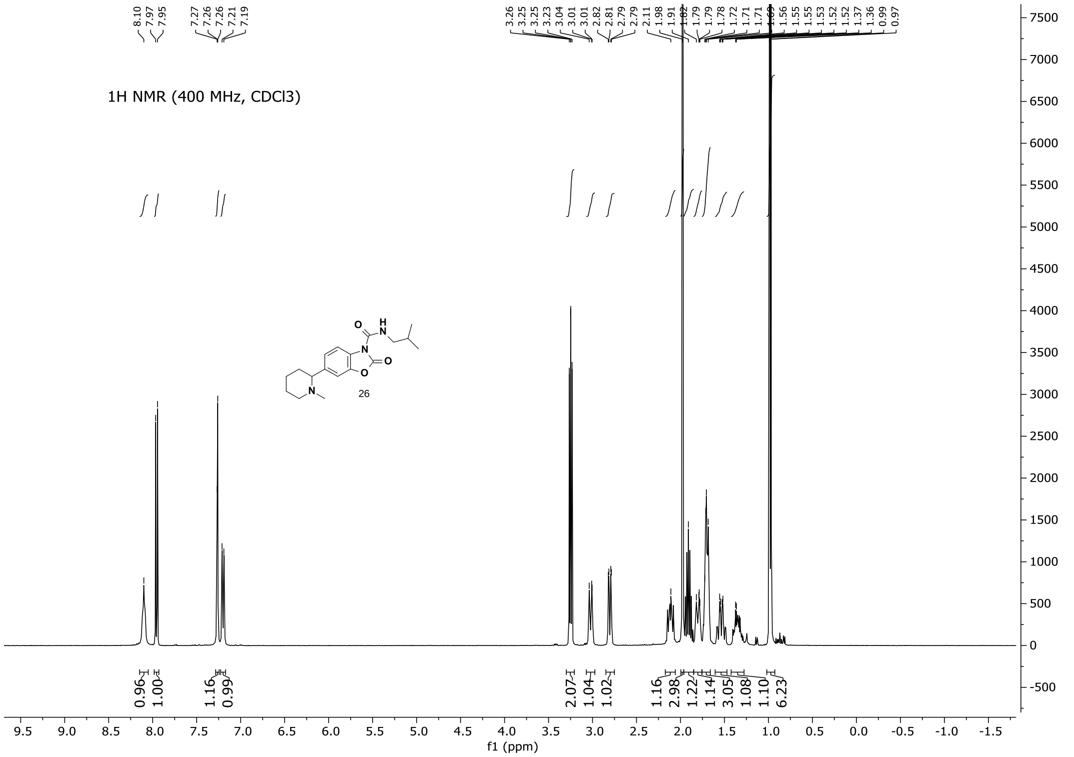
1H NMR (400 MHz, CDCl3)
26
f1 (ppm)

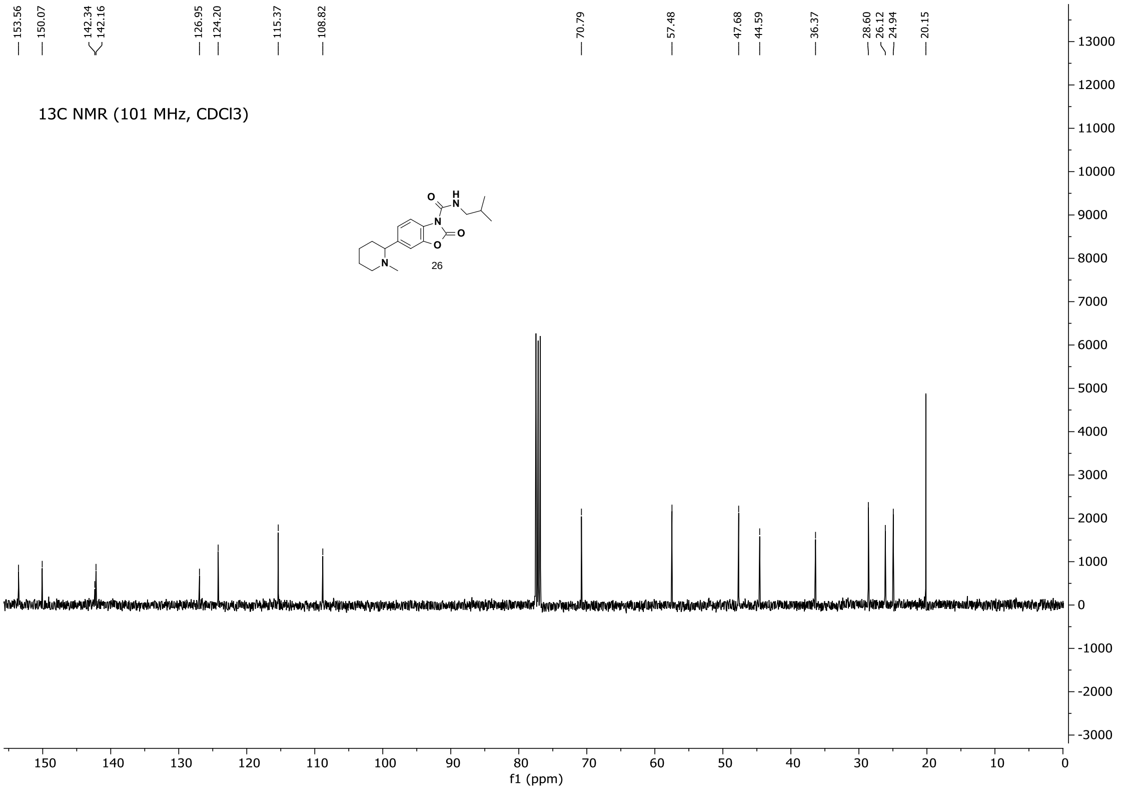
13C NMR (101 MHz, CDCl3)
26
f1 (ppm)

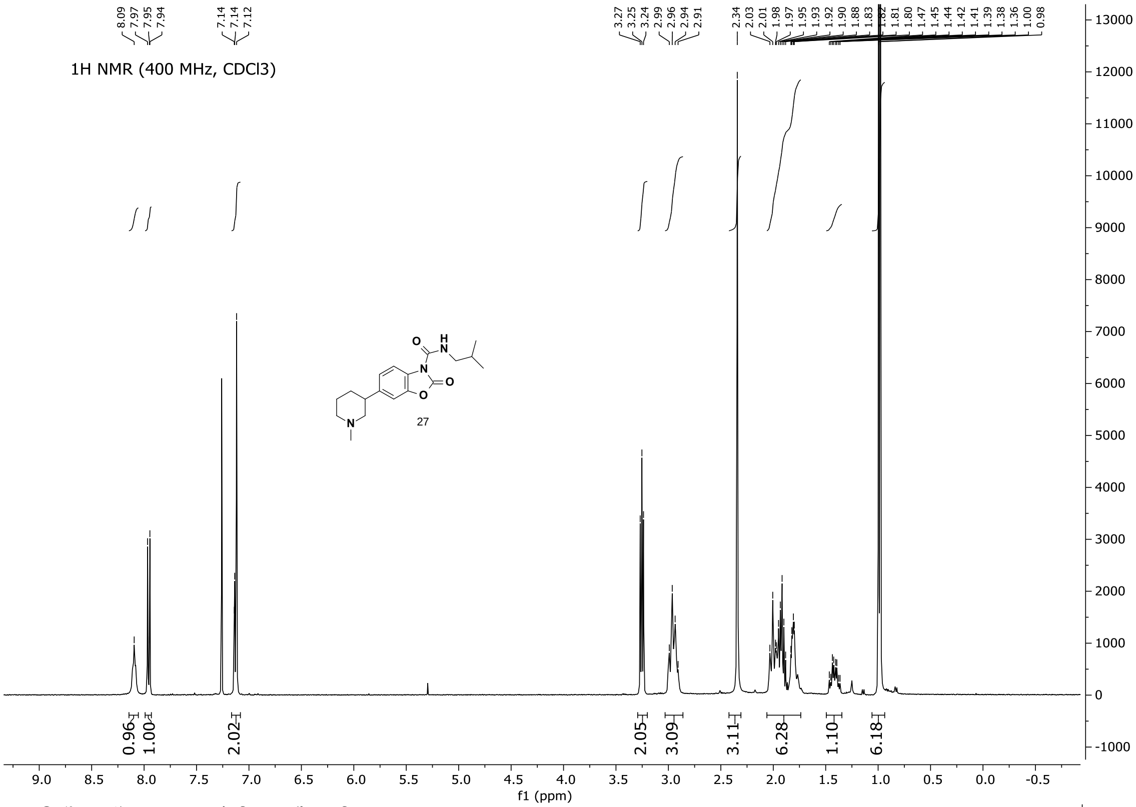
1H NMR (400 MHz, CDCl3)
8.09 7.97 7.95 7.94
7.14 7.14 7.12
3.27 3.25 3.24 2.99 2.96 2.94 2.91
2.34 2.03 2.01 1.98 1.97 1.95 1.93 1.92 1.90 1.88 1.83 1.82 1.81 1.80 1.47 1.45 1.44 1.42 1.41 1.39 1.38 1.36 1.00 0.98
O
H
N
N
O
O
N
27
0.96
1.00
2.02
2.05
3.09
3.11
6.28
1.10
6.18
f1 (ppm)

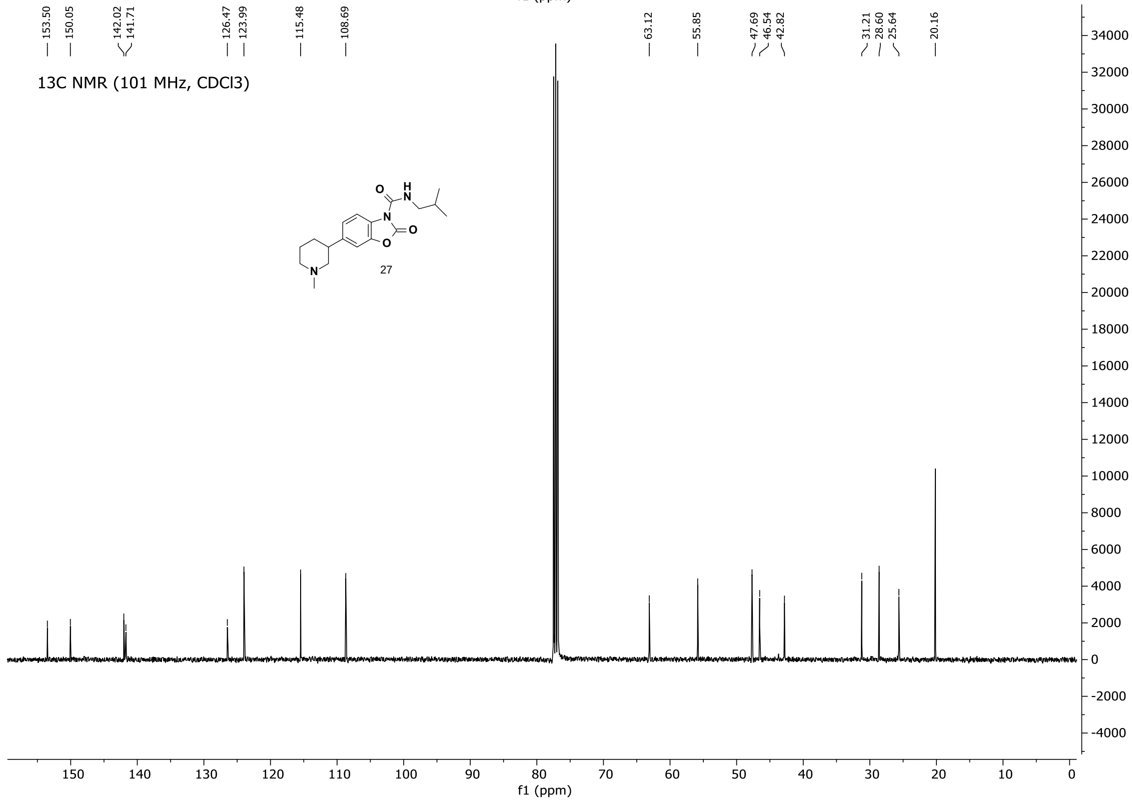
13C NMR (101 MHz, CDCl3)
153.50 150.05 142.02 141.71 126.47 123.99 115.48 108.69
63.12 55.85 47.69 46.54 42.82 31.21 28.60 25.64 20.16
O
H
N
N
O
O
N
27
f1 (ppm)

Table S2. Retention times and UPLC analytical methods of the final compounds.[a]

compound	Rt (min)	method	compound	Rt (min)	method
**2d**	3.70	F	**22k**	3.83	E
**2e**	4.29	F	**22l**	2.60	E
**2f**	5.32	E	**22m**	3.39	E
**2g**	5.16	E	**22n**	3.33	E
**8a**	4.73	E	**22o**	4.33	E
**8b**	4.77	E	**22p**	4.47	E
**8c**	5.33	E	**22q**	4.98	E
**8d**	5.57	E	**22r**	4.07	E
**12b**	3.50	E	**23a**	4.78	F
**12c**	4.48	E	**23b**	5.49	E
**12d**	4.06	F	**23c**	5.08	E
**12e**	4.33	E	**23e**	4.16	E
**17b**	3.81	E	**23f**	4.41	E
**22a**	5.69	F	**23g**	4.49	E
**22b**	5.92	E	**23h**	4.83	E
**22d**	4.32	E	**23i**	3.68	F
**22e**	4.34	E	**23j**	4.80	E
**22f**	4.53	E	**23k**	4.43	E
**22g**	4.53	E	**24c**	3.51	E
**22h**	5.06	E	**25**	3.53	E
**22i**	5.16	E	**26**	3.47	E
**22j**	3.59	E	**27**	3.45	E

[a]Freshly prepared 10 mM DMSO-d_6 stock solutions of the final compounds (used for biological screenings) were diluted 20- fold or 100 fold in MeCN/H_2O (1:1) and directly analyzed by ACQUITY UPLC BEH C18 (100x2.1 mm ID, particle size 1.7 µm) with a VanGuard BEH C18 pre-column (5x 2.1 mm ID, particle size 1.7 µm). Mobile phase was 10 mM NH_4OAc in H_2O at pH 5 adjusted with AcOH (A) and 10 mM NH_4OAc in MeCN-H_2O (95: 5) at pH 5 (B). *Method E*: Gradient: 10 to 90% B over 6 min. *Method F*: Gradient: 50 to 100% B over 6 min. Flow rate 0.5 mL/min; temperature 40 °C.

UPLC traces of the final compounds.

Compound **2d**

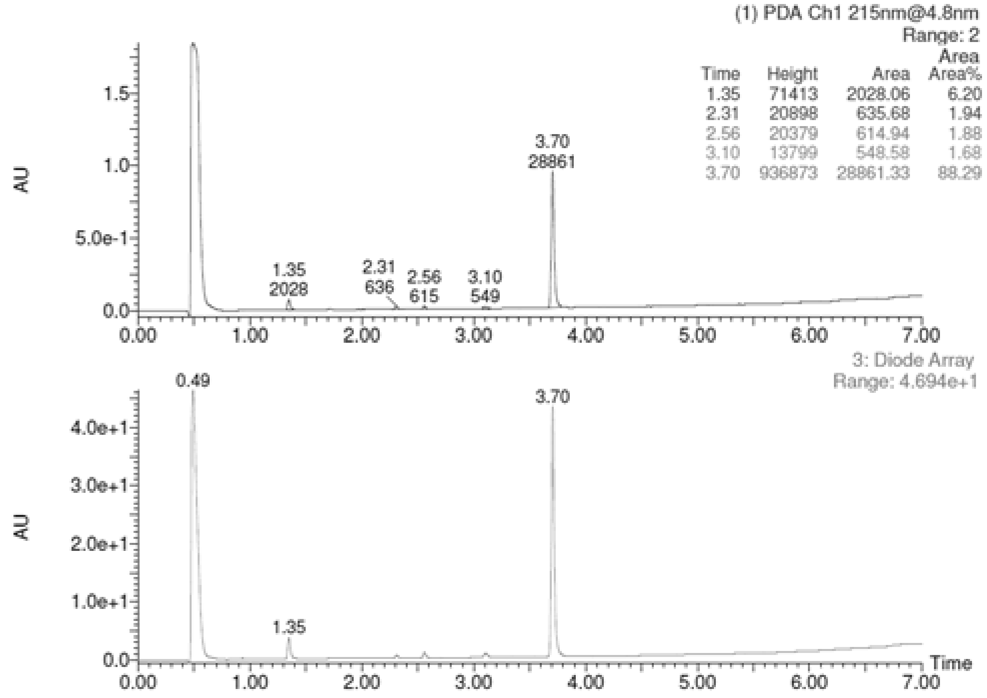

Compound **2e**

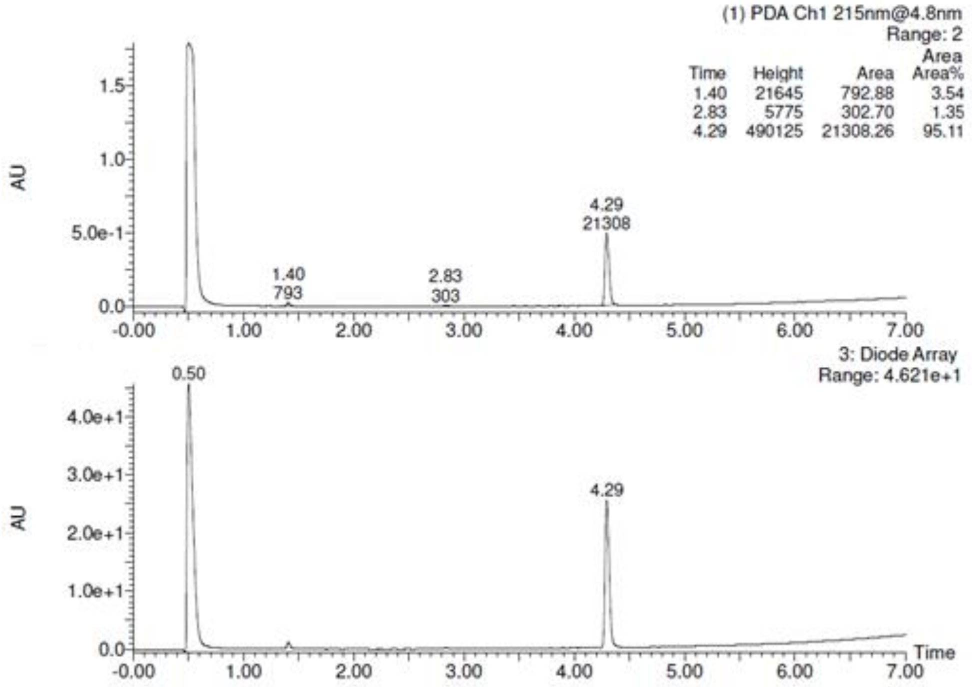

Compound **2f**

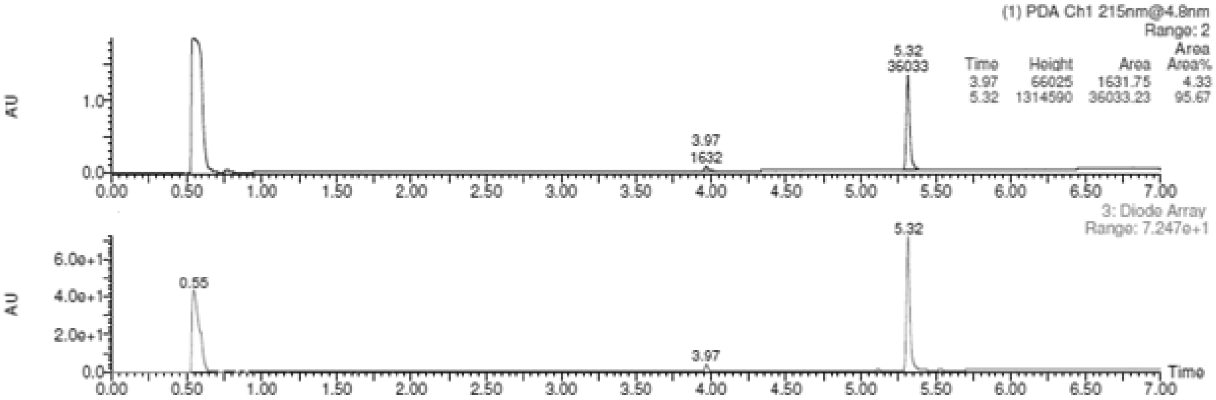

Compound **2g**

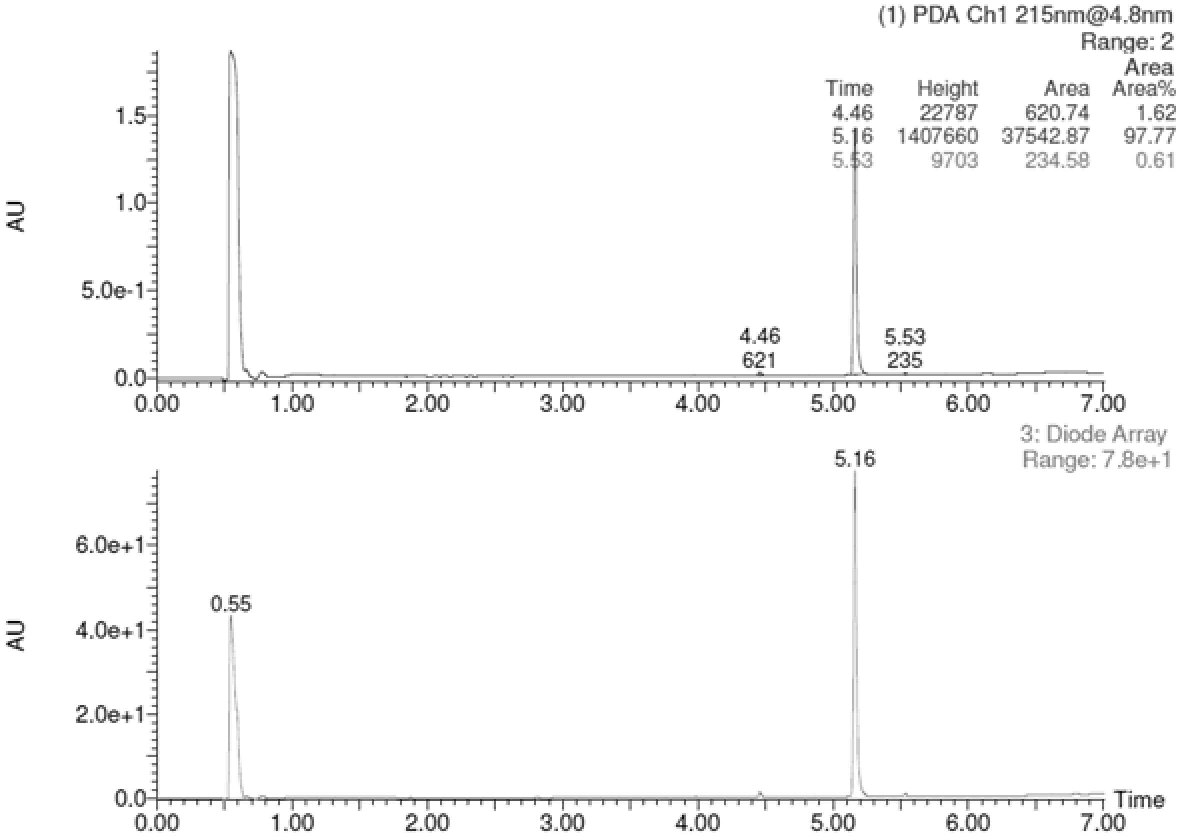

Compound **8a**

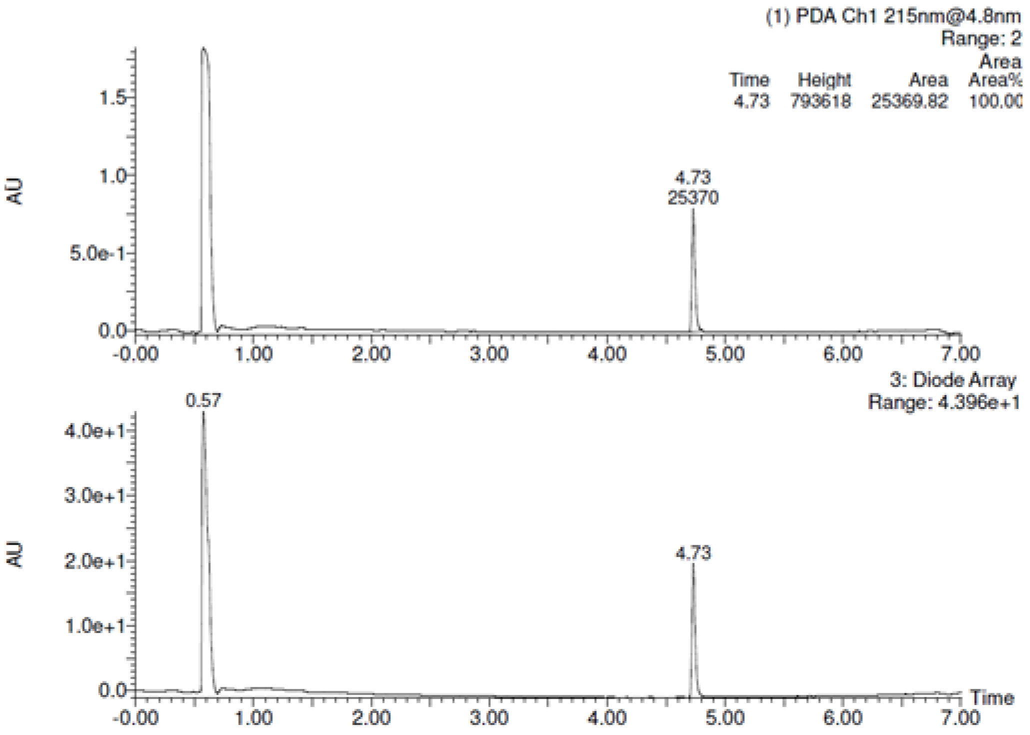

Compound **8b**

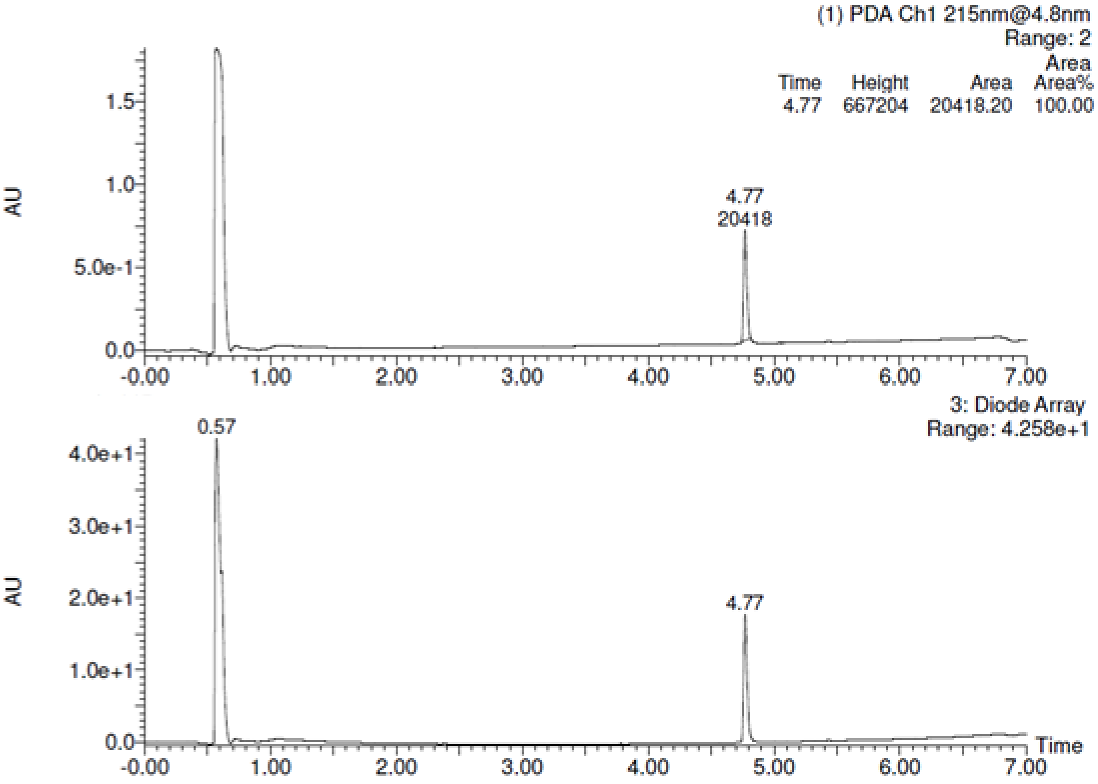

Compound **8c**

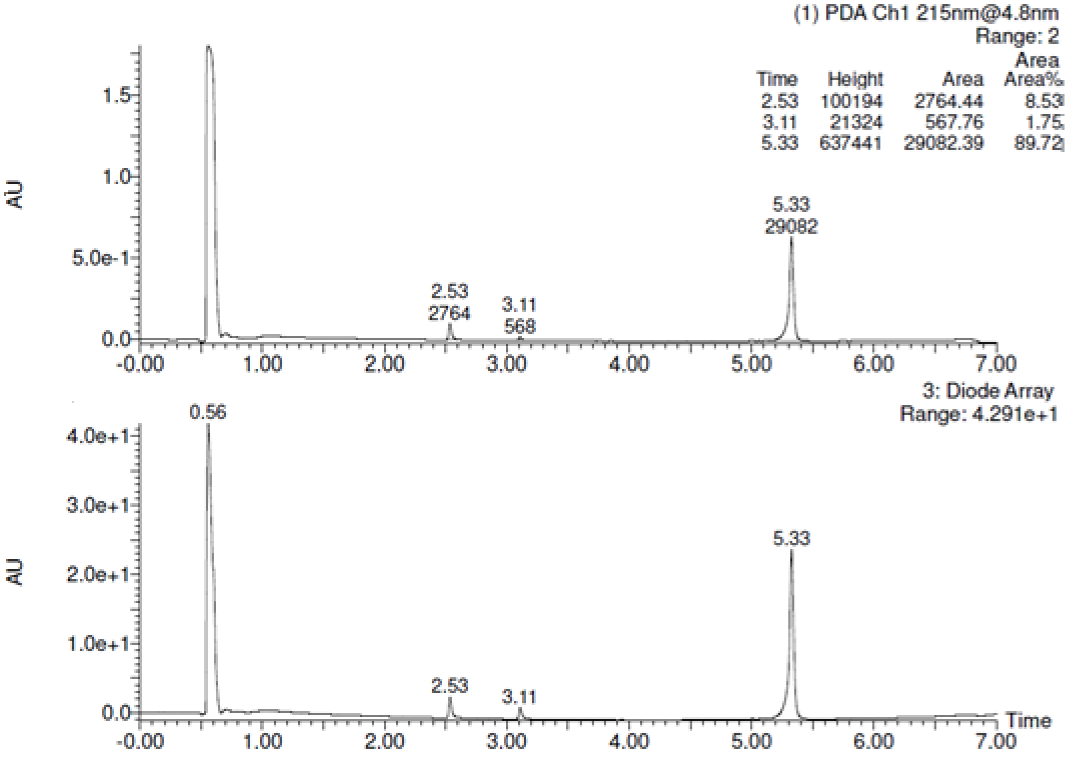

Compound **8d**

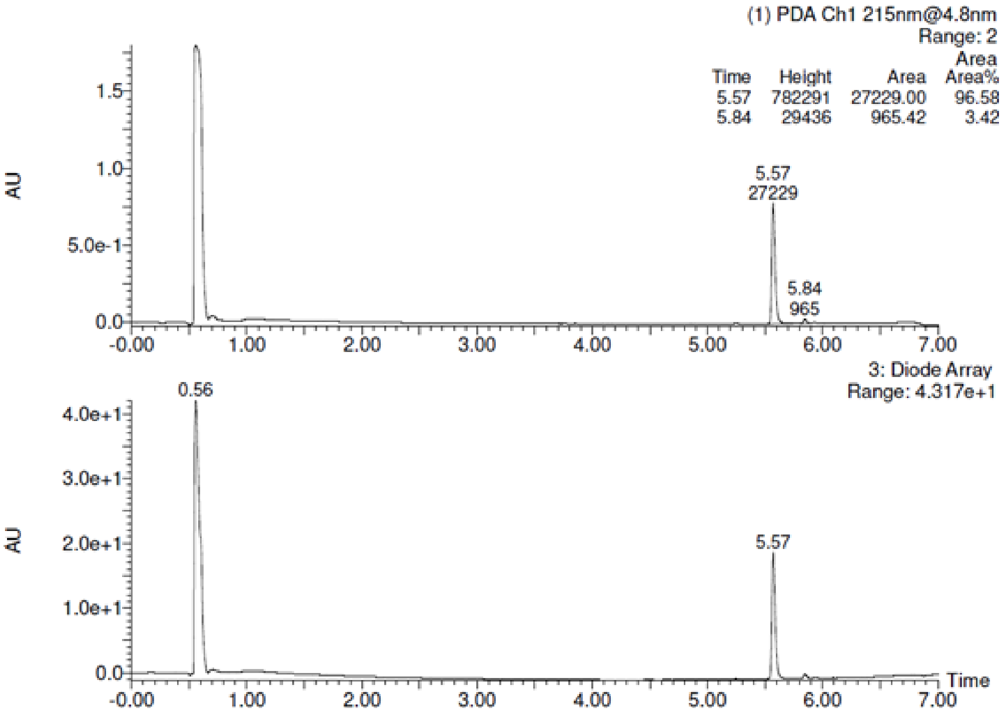

Compound **12b**

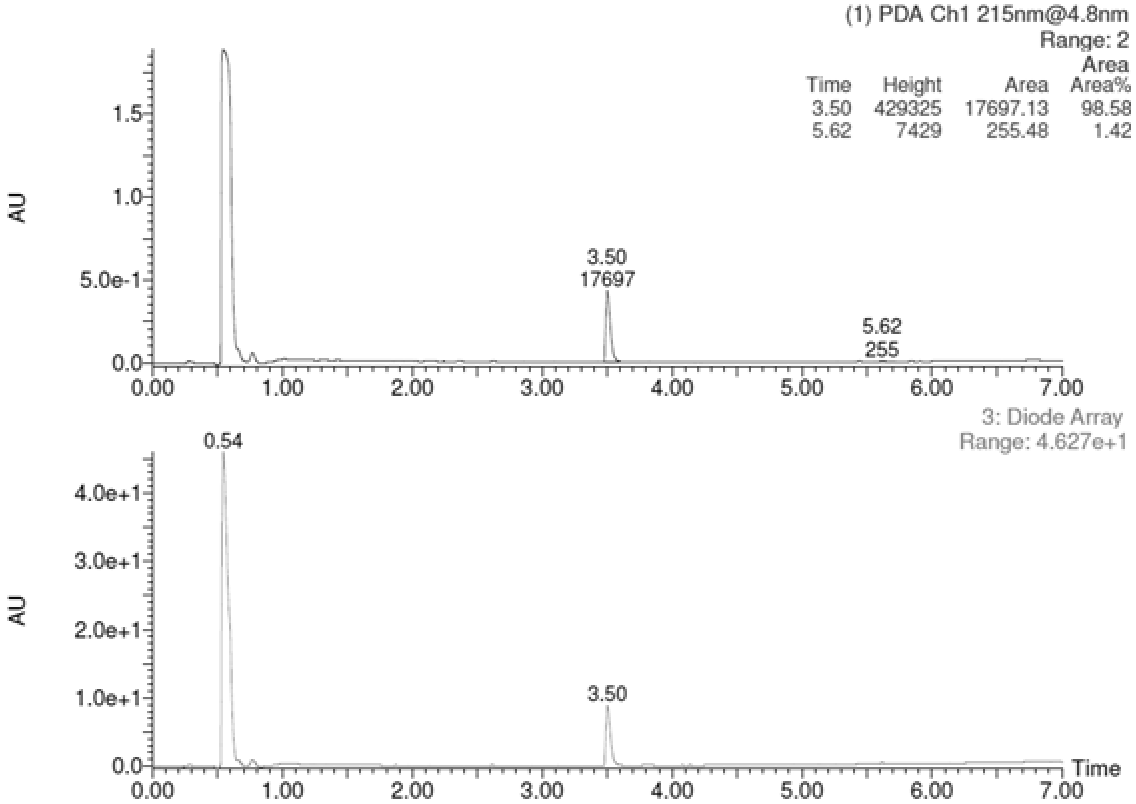

Compound **12c**

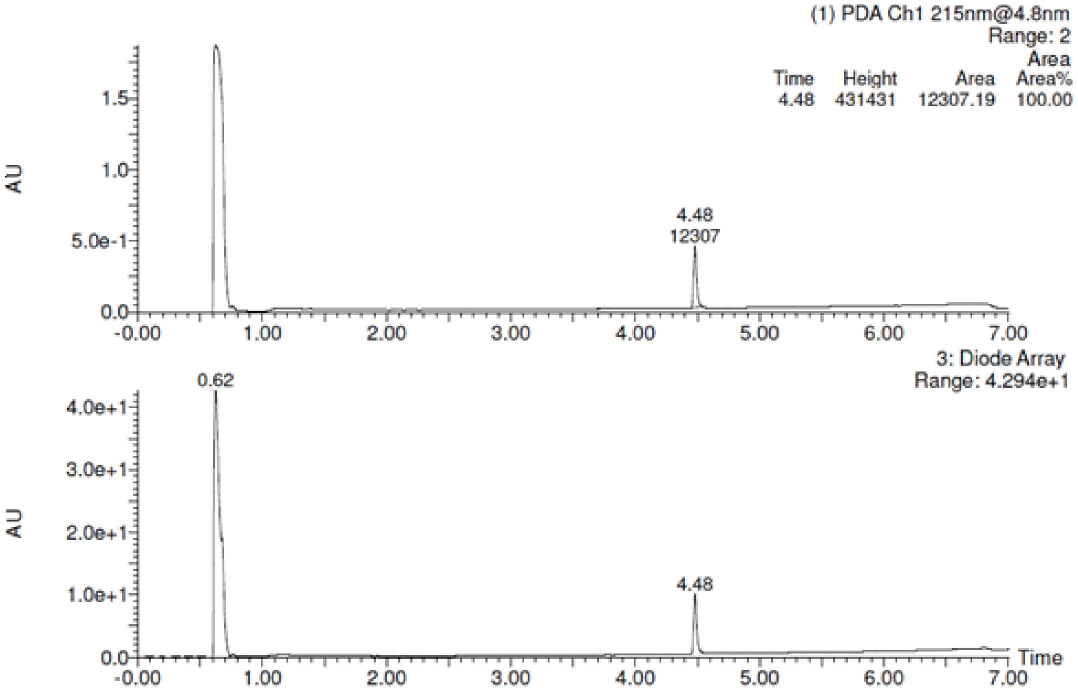

Compound **12d**

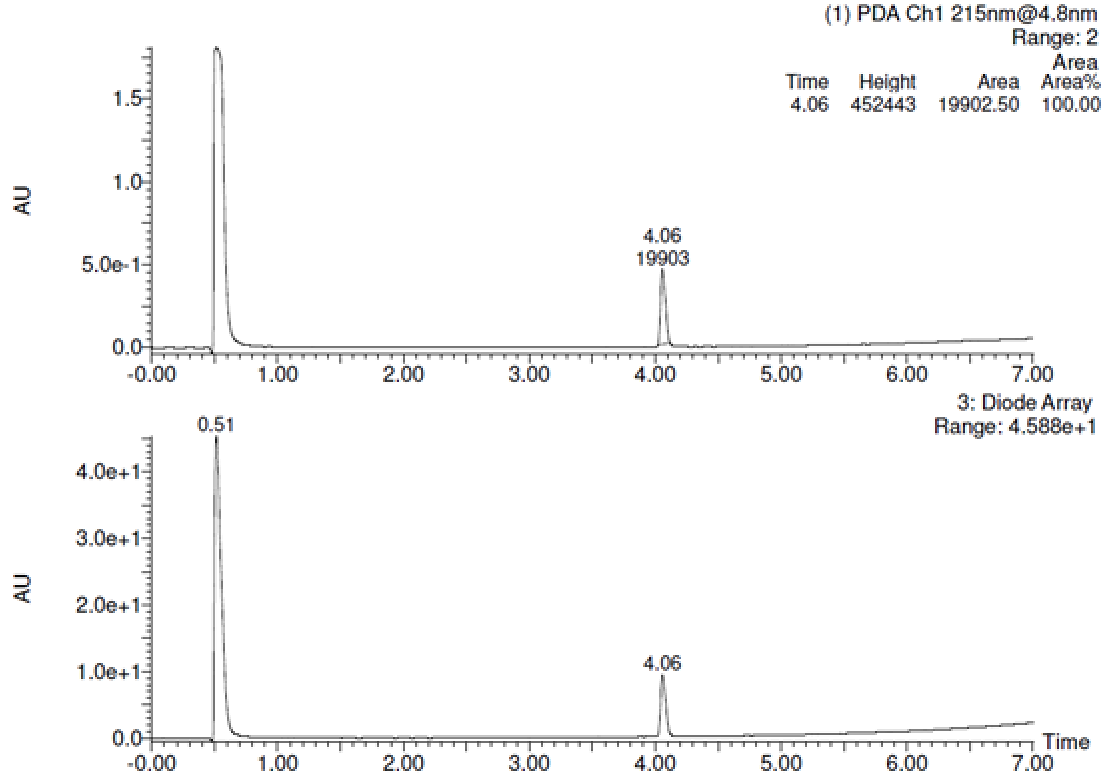

Compound **12e**

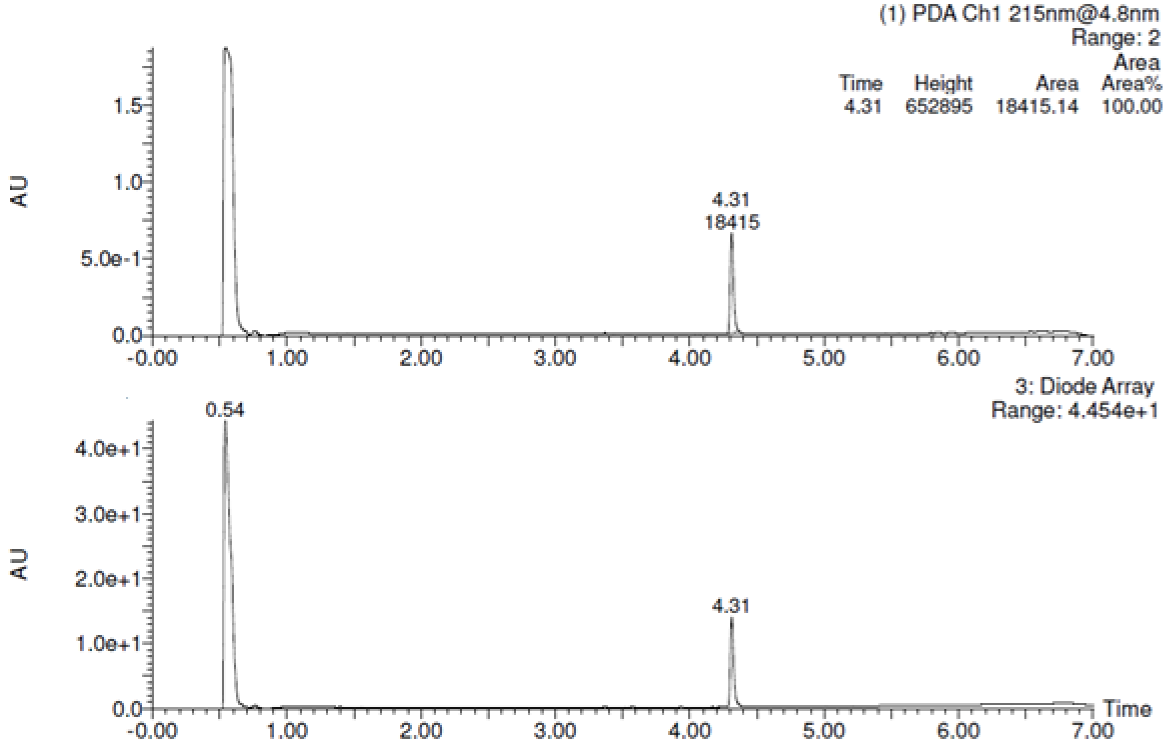

Compound **17b**

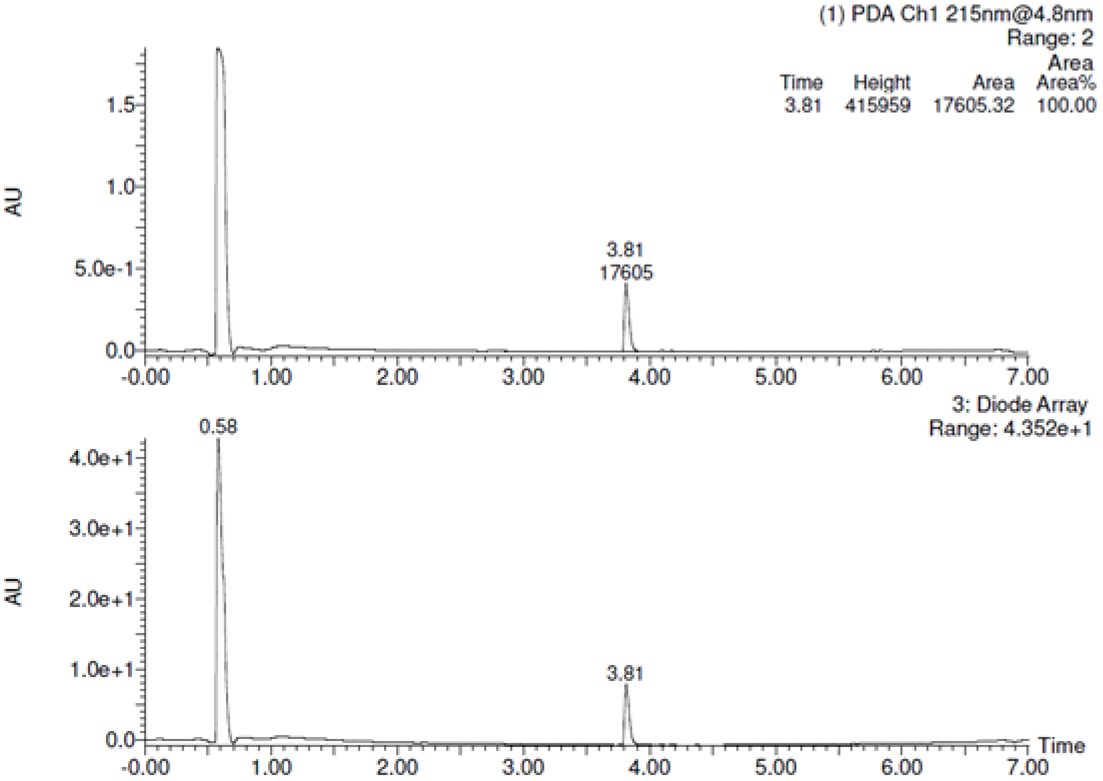

Compound **22a**

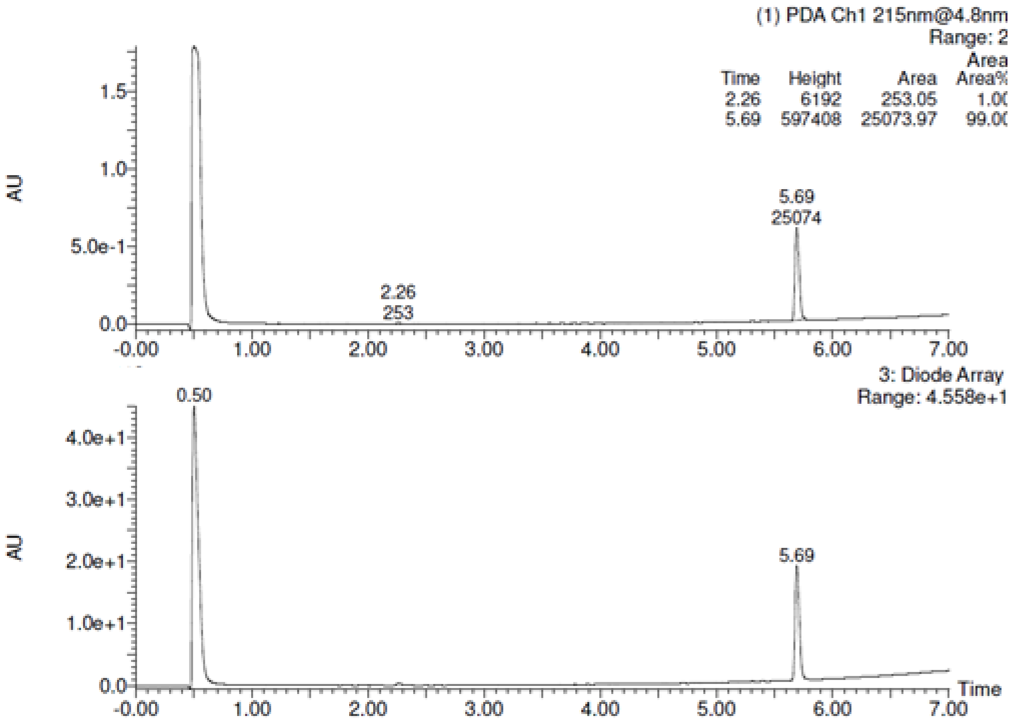

Compound **22b**

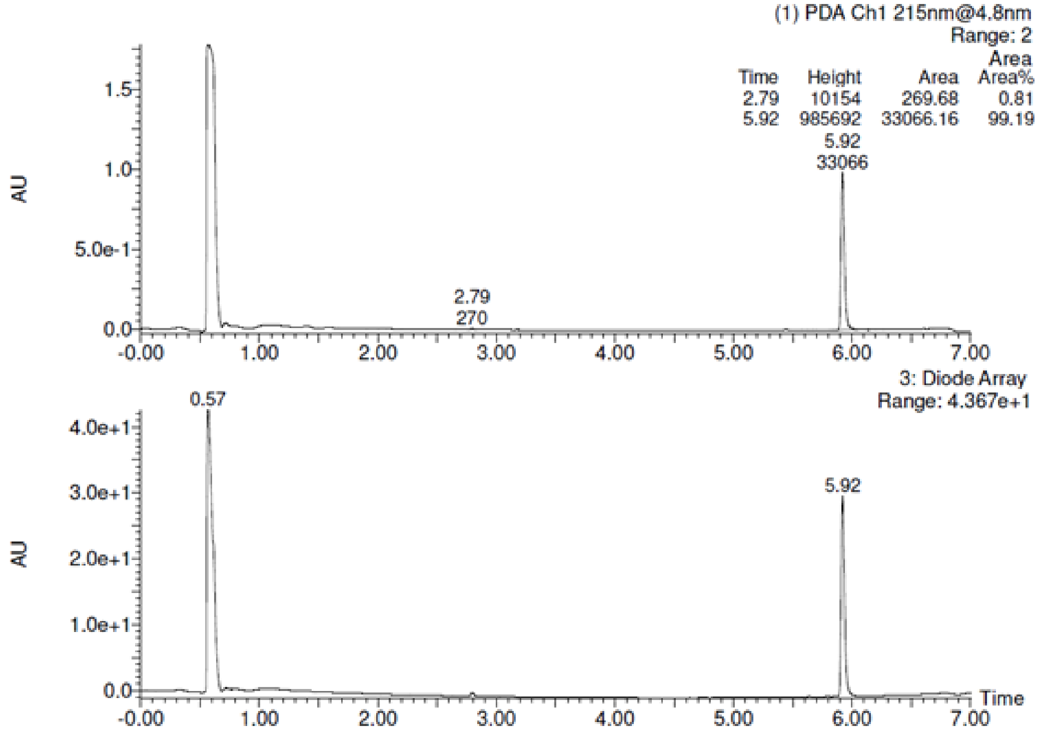

Compound **22d**

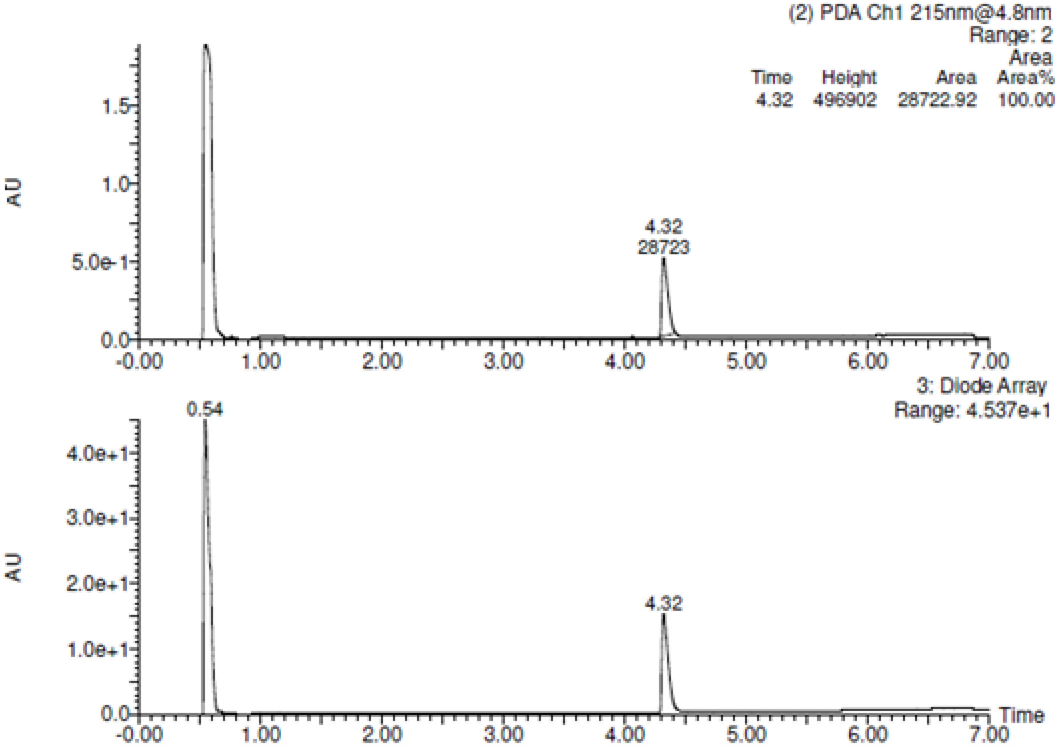

Compound **22e**

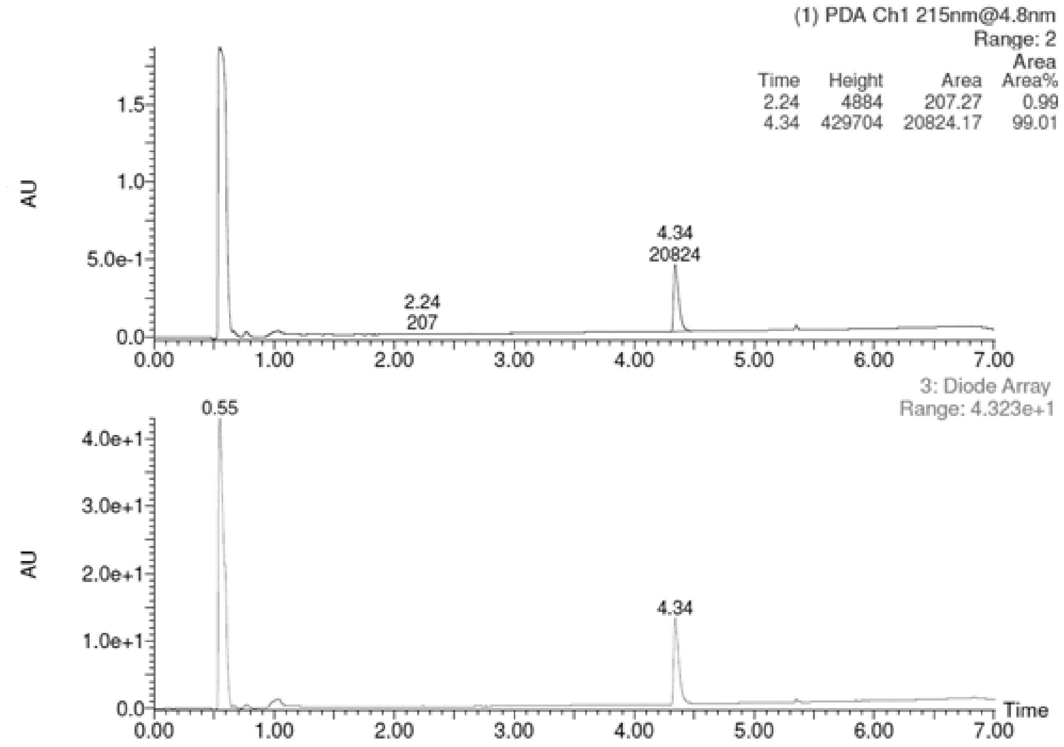

Compound **22f**

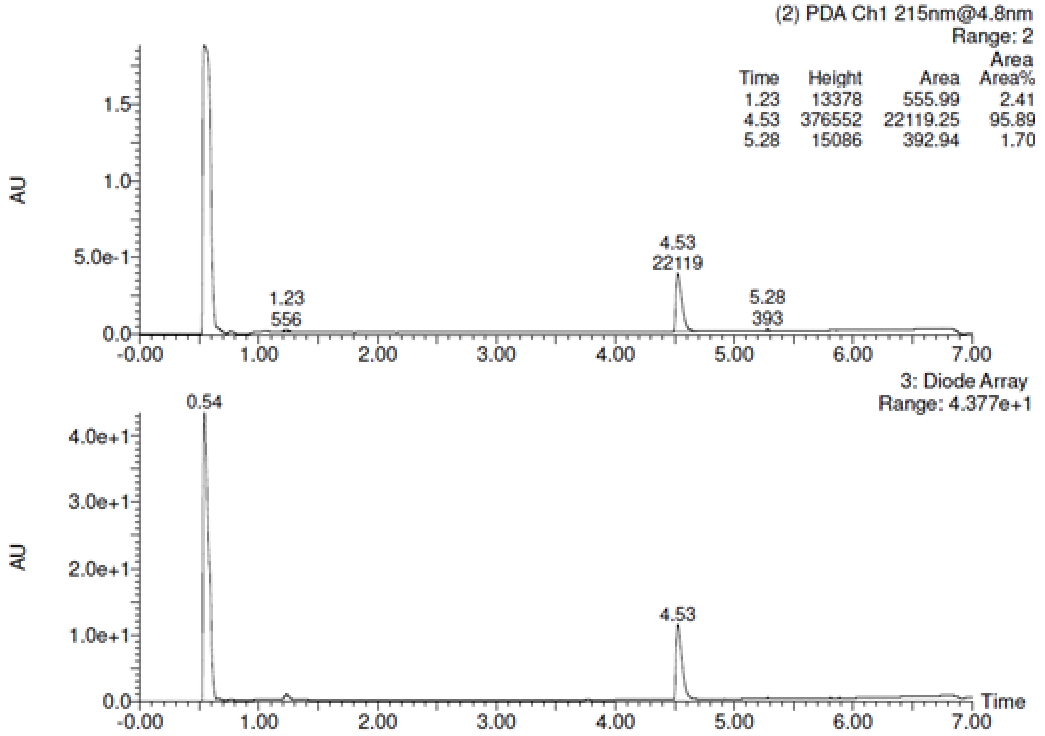

Compound **22g**

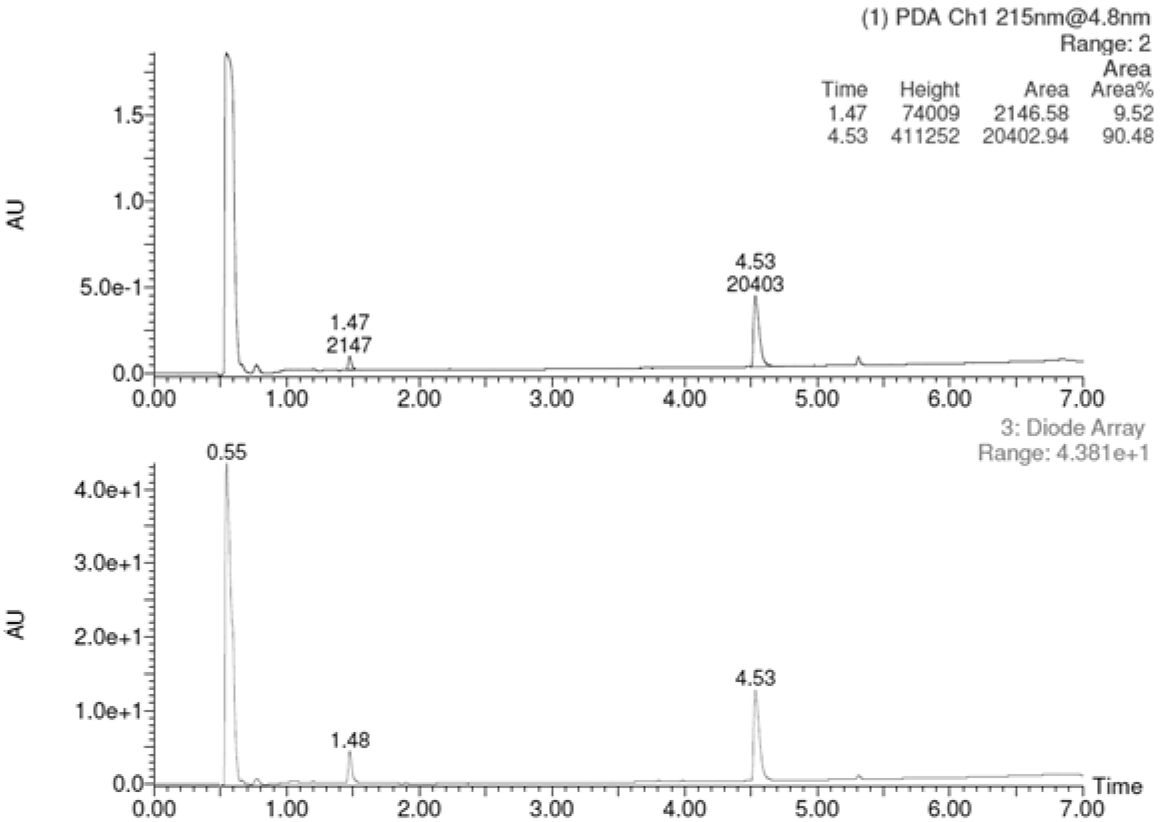

Compound **22h**

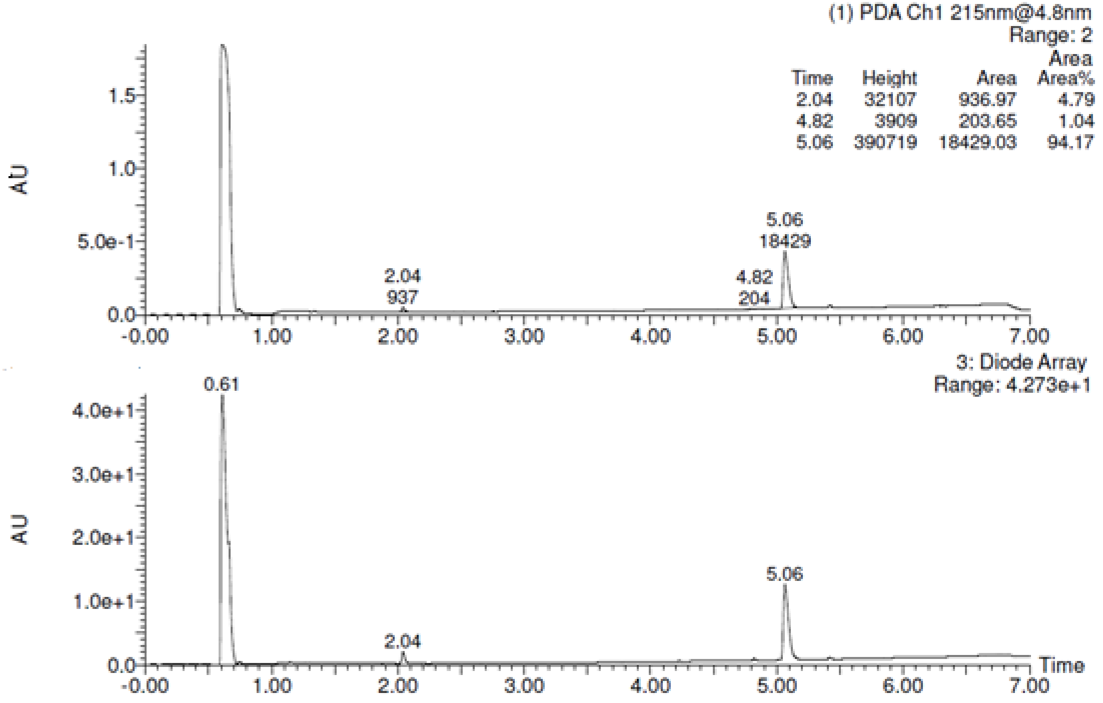

Compound **22i**

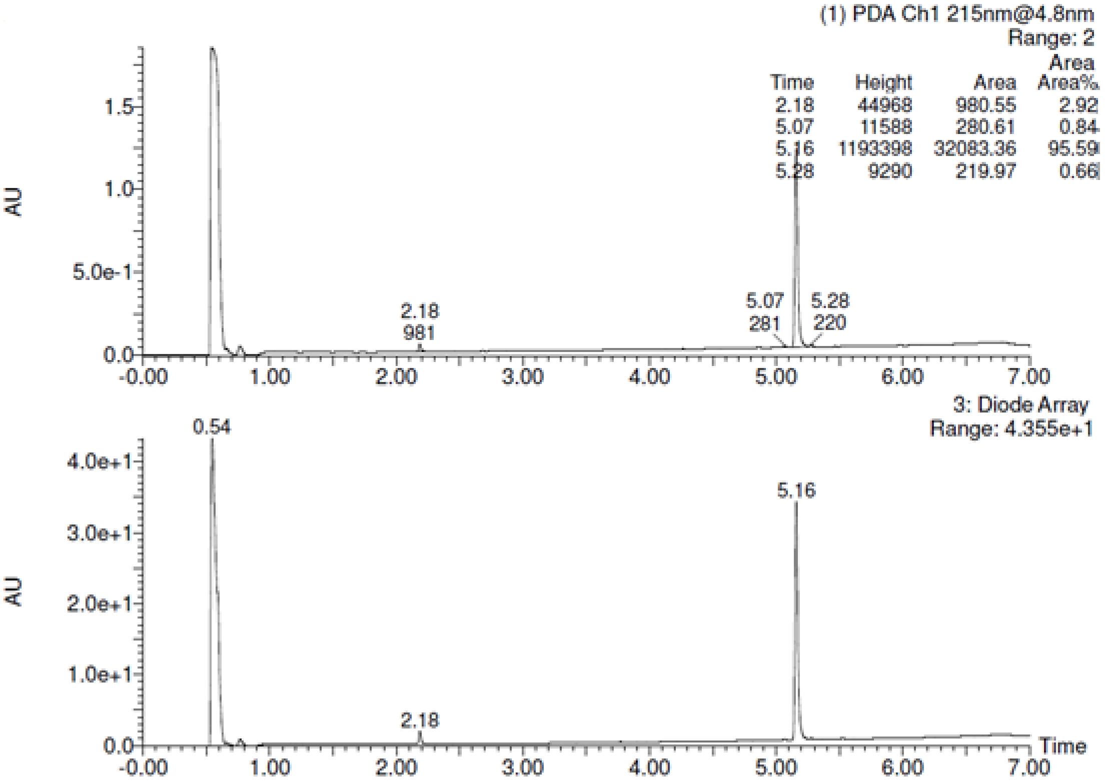

Compound **22j**

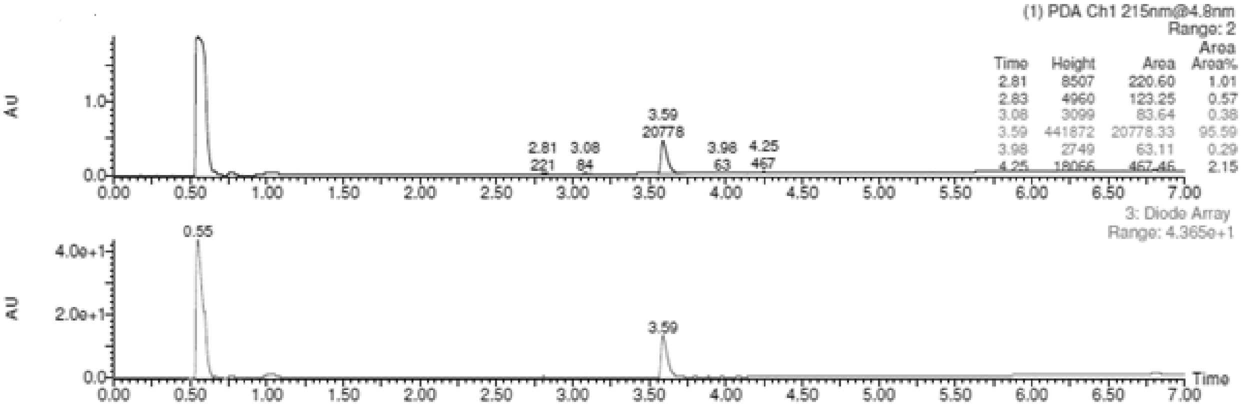

Compound **22k**

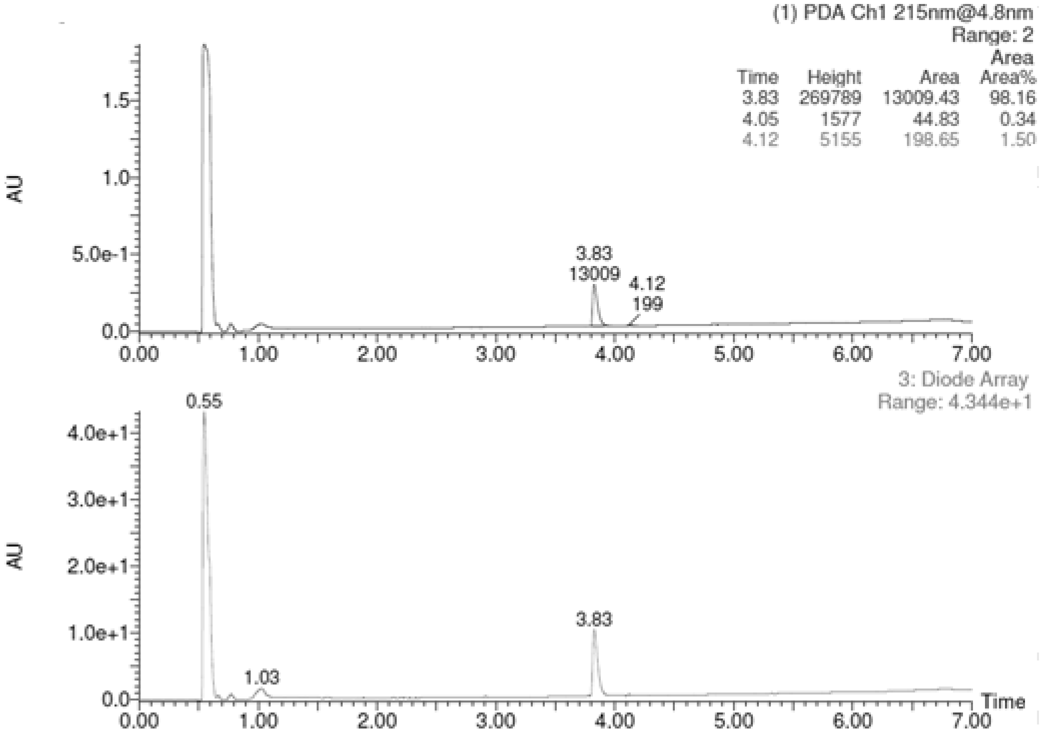

Compound **22l**

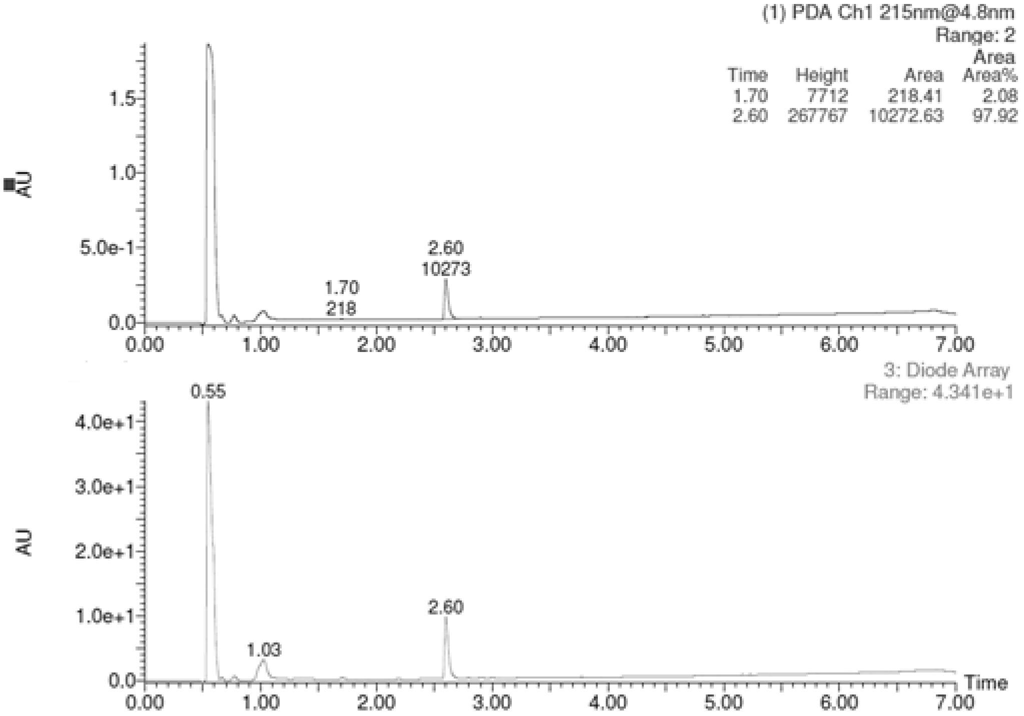

Compound **22m**

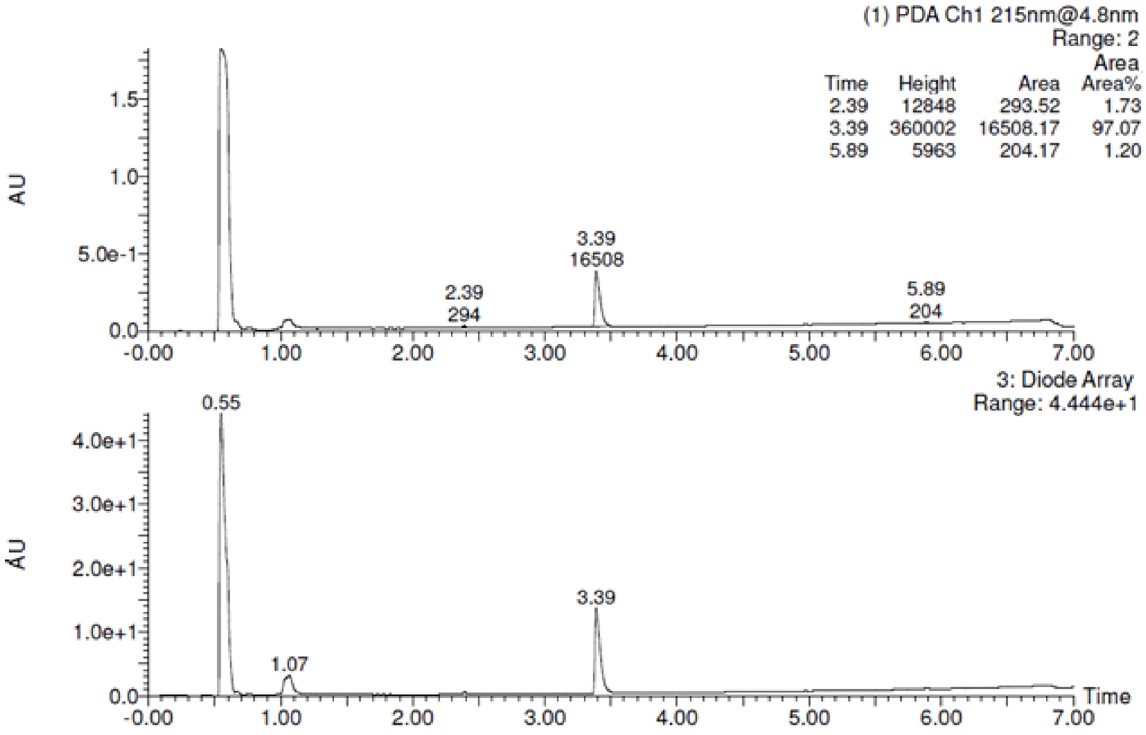

Compound **22n**

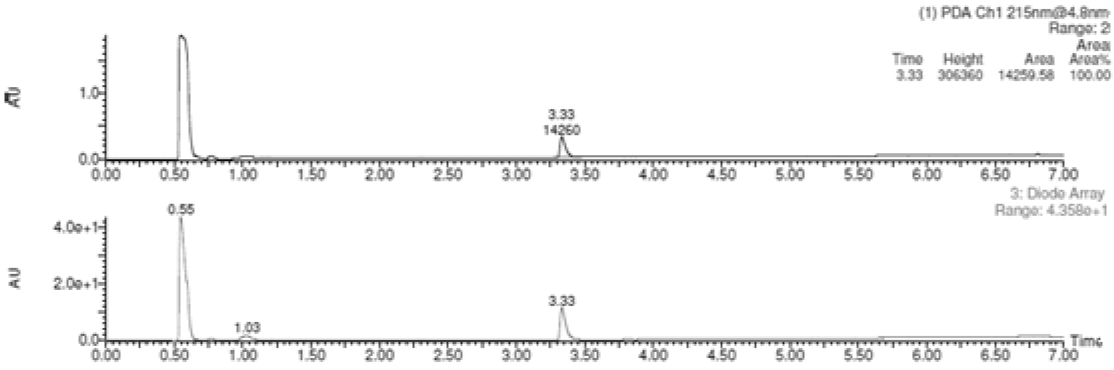

Compound **22o**

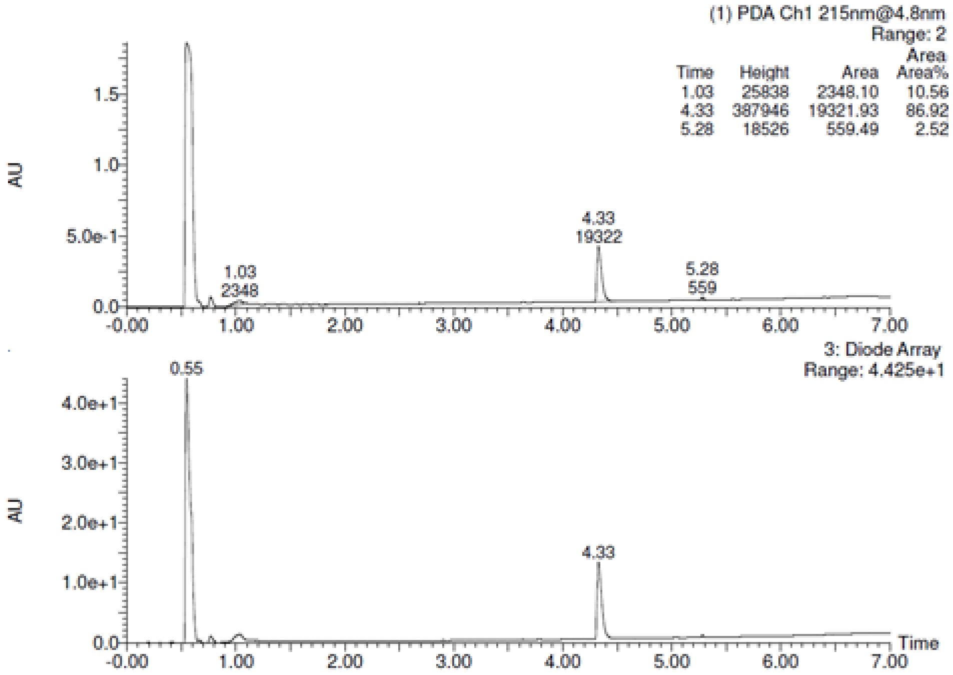

Compound **22p**

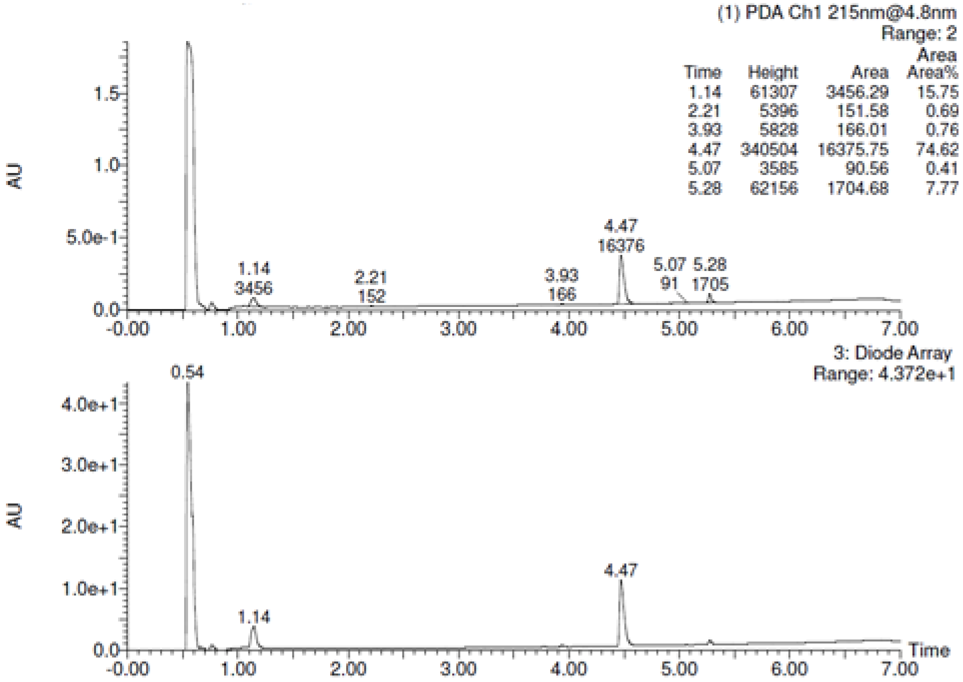

Compound **22q**

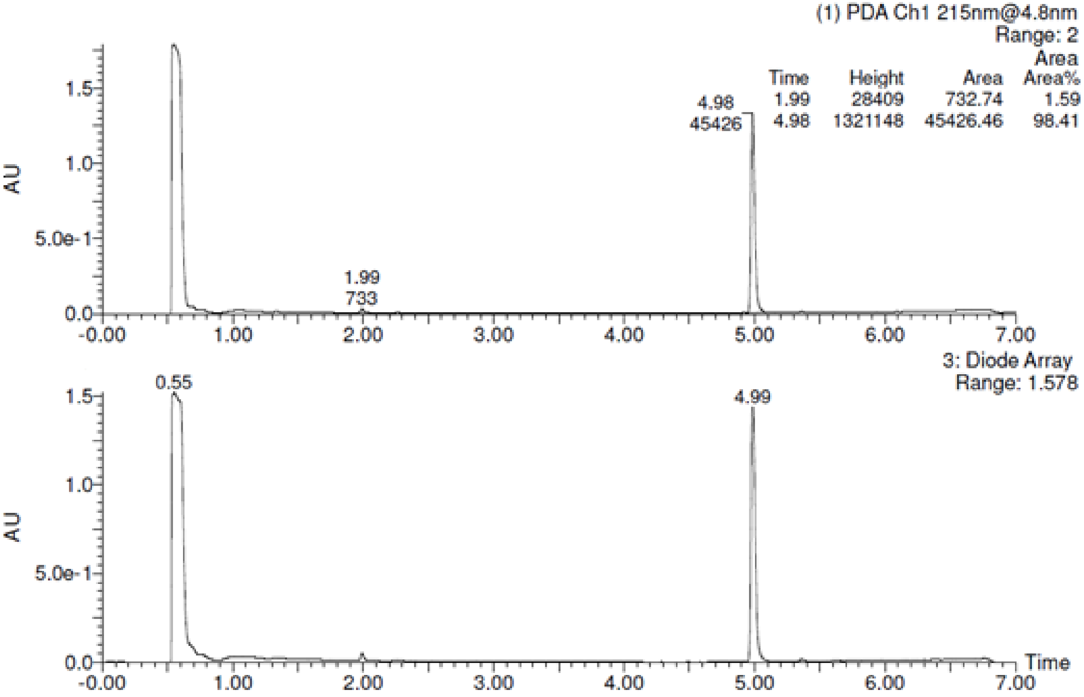

Compound **22r**

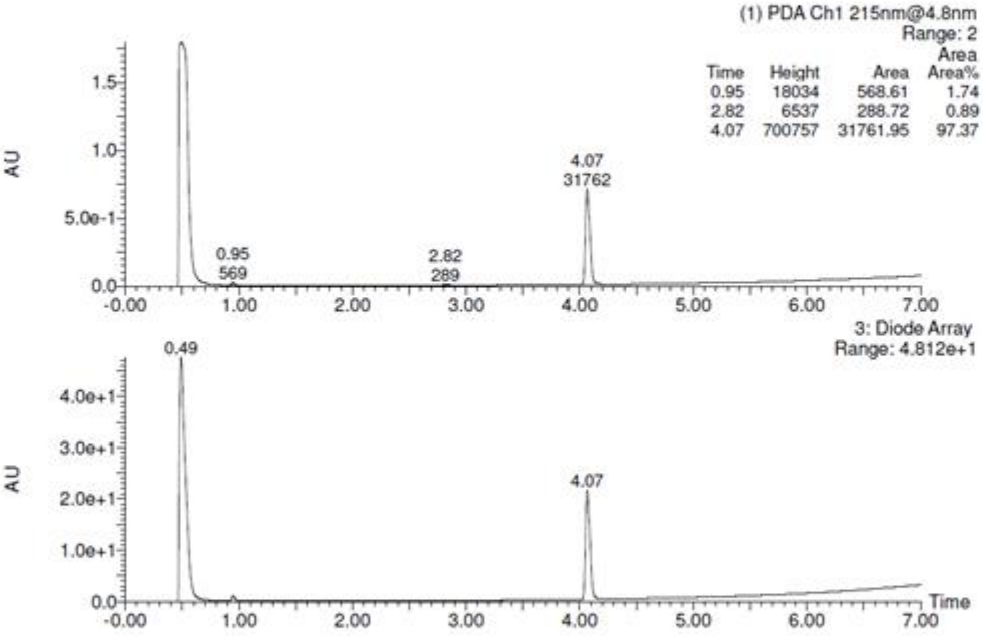

Compound **23a**

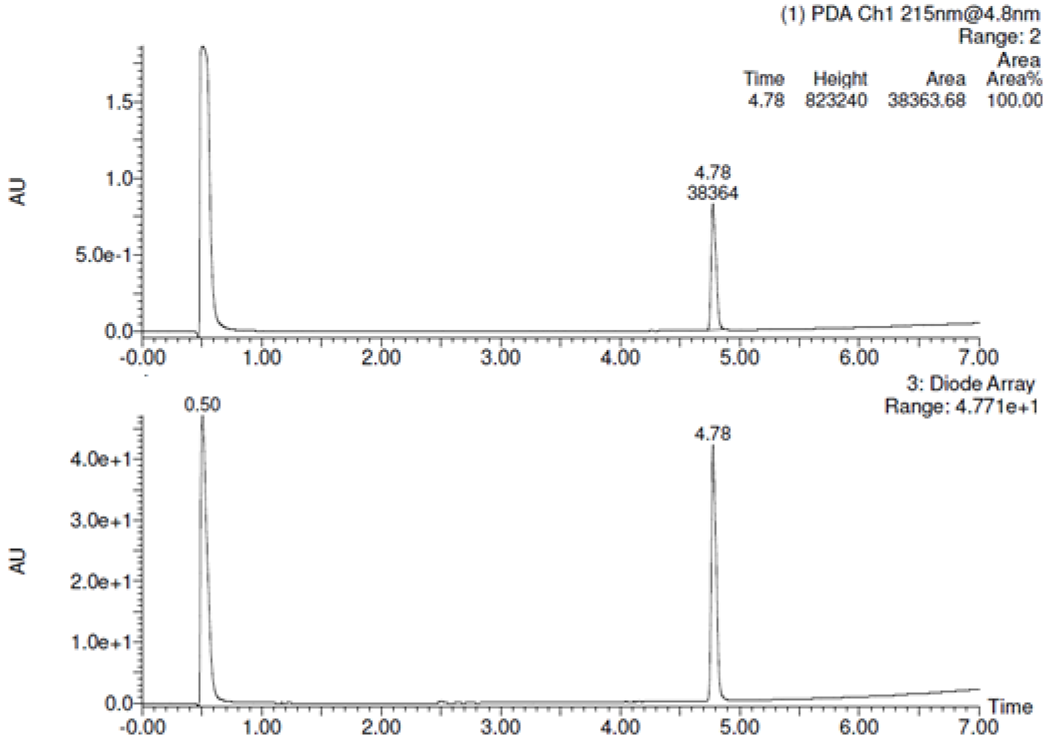

Compound **23b**

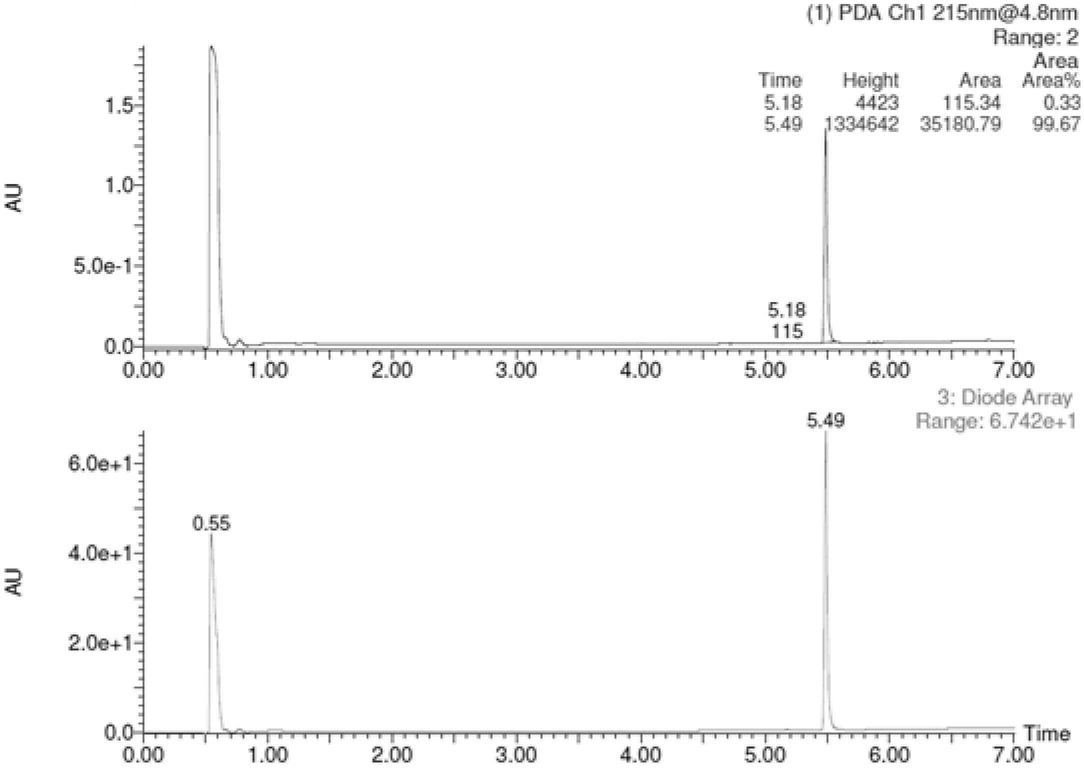

Compound **23c**

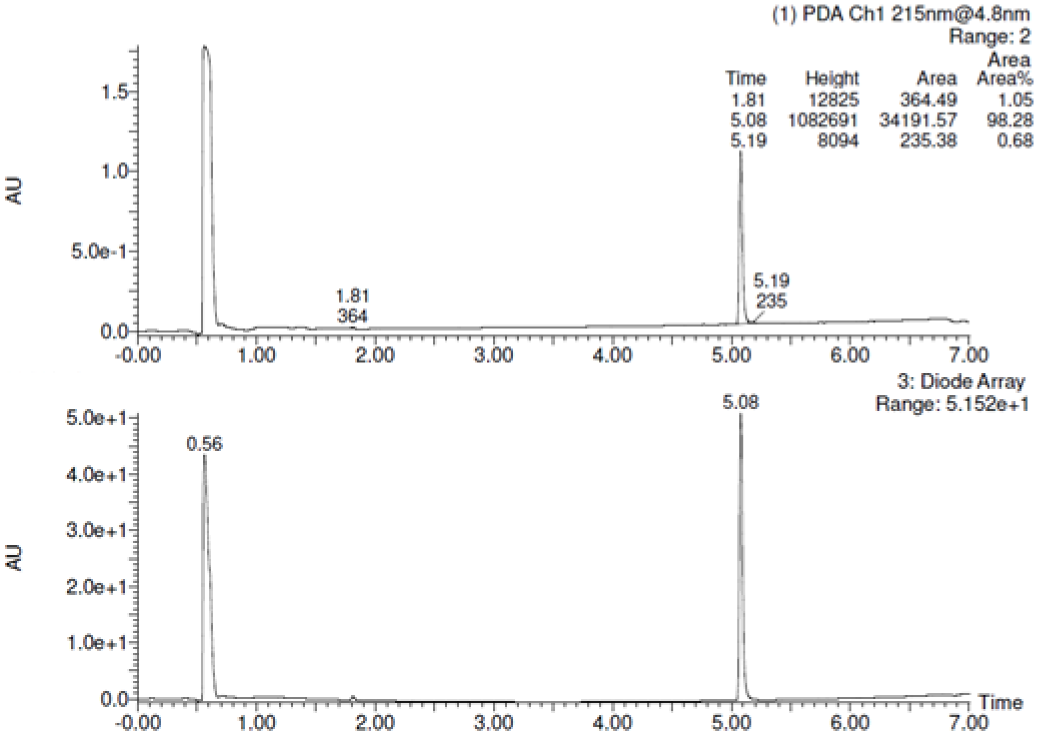

Compound **23e**

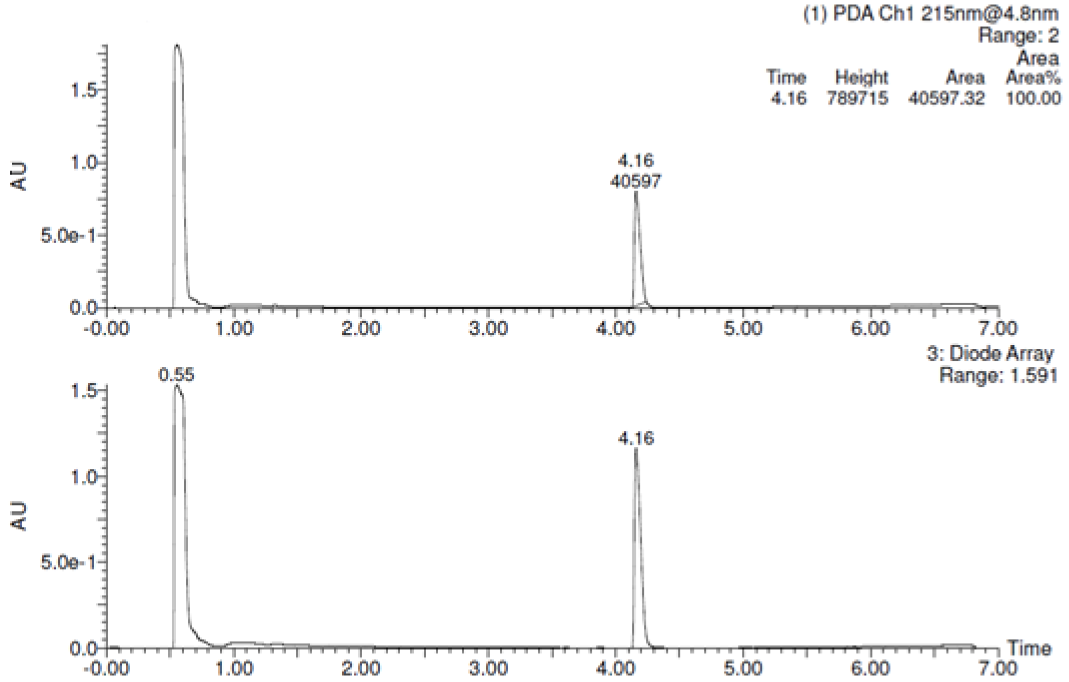

Compound **23f**

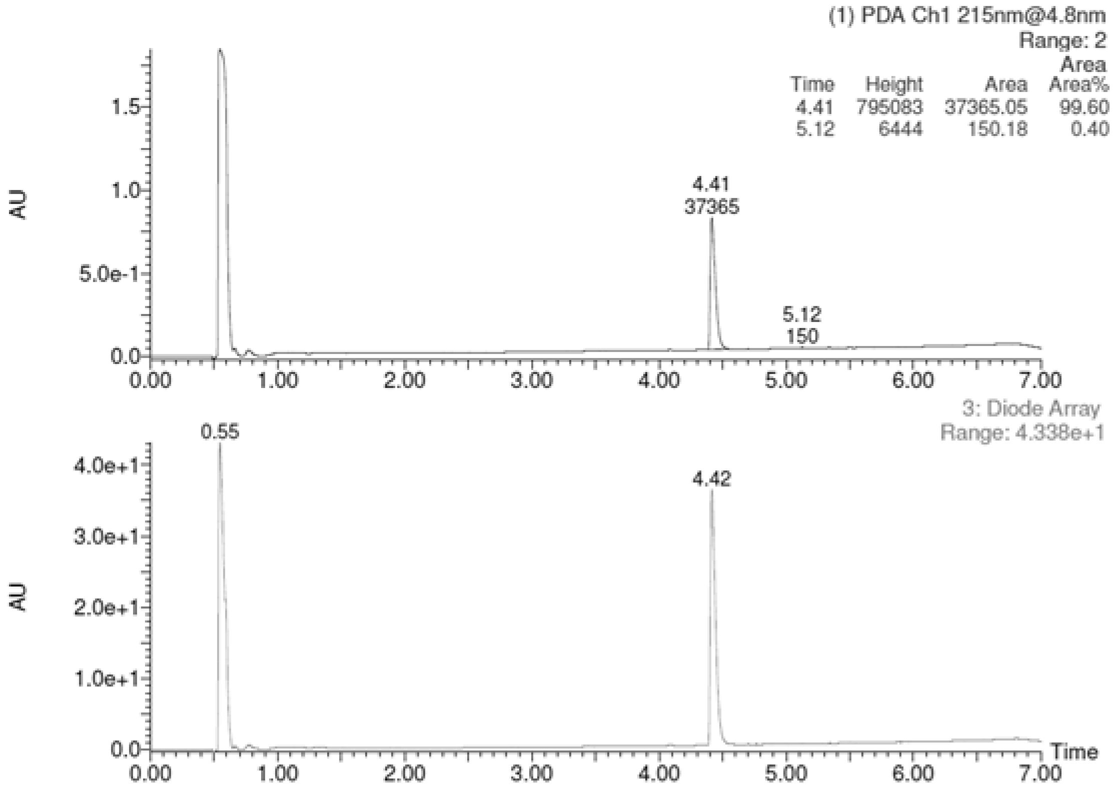

Compound **23g**

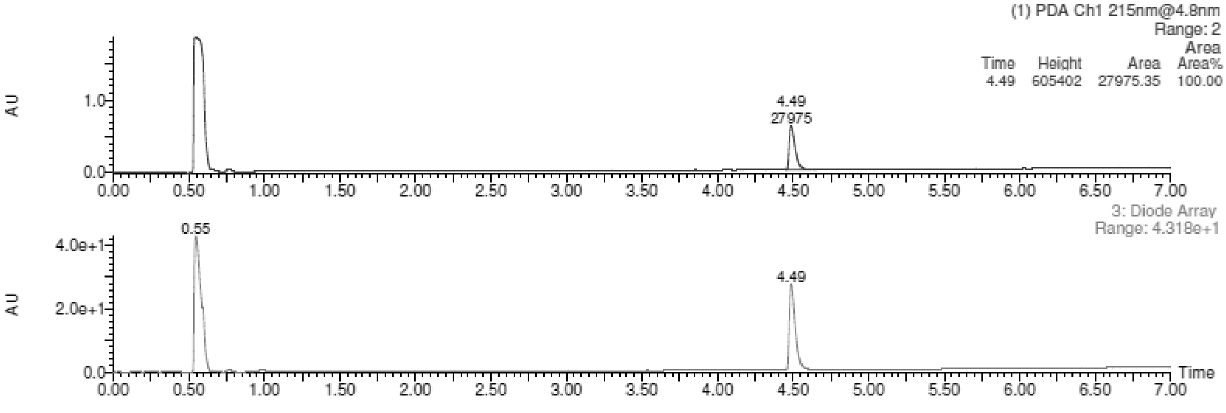

Compound **23h**

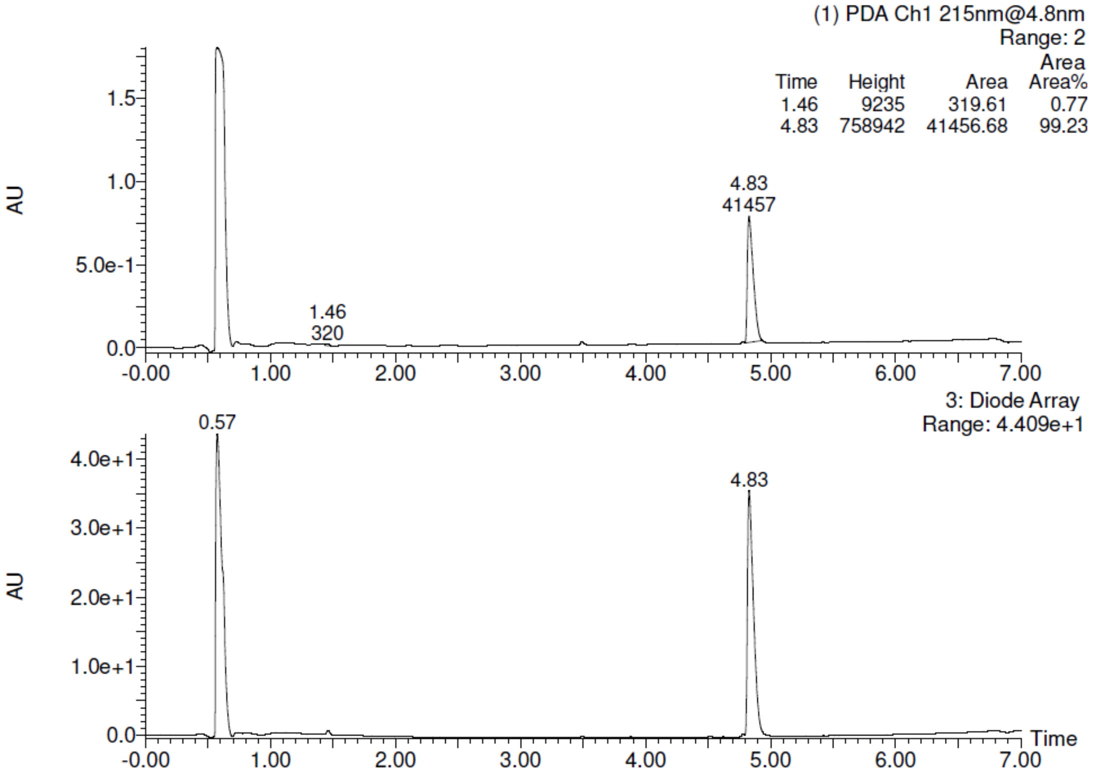

Compound **23i**

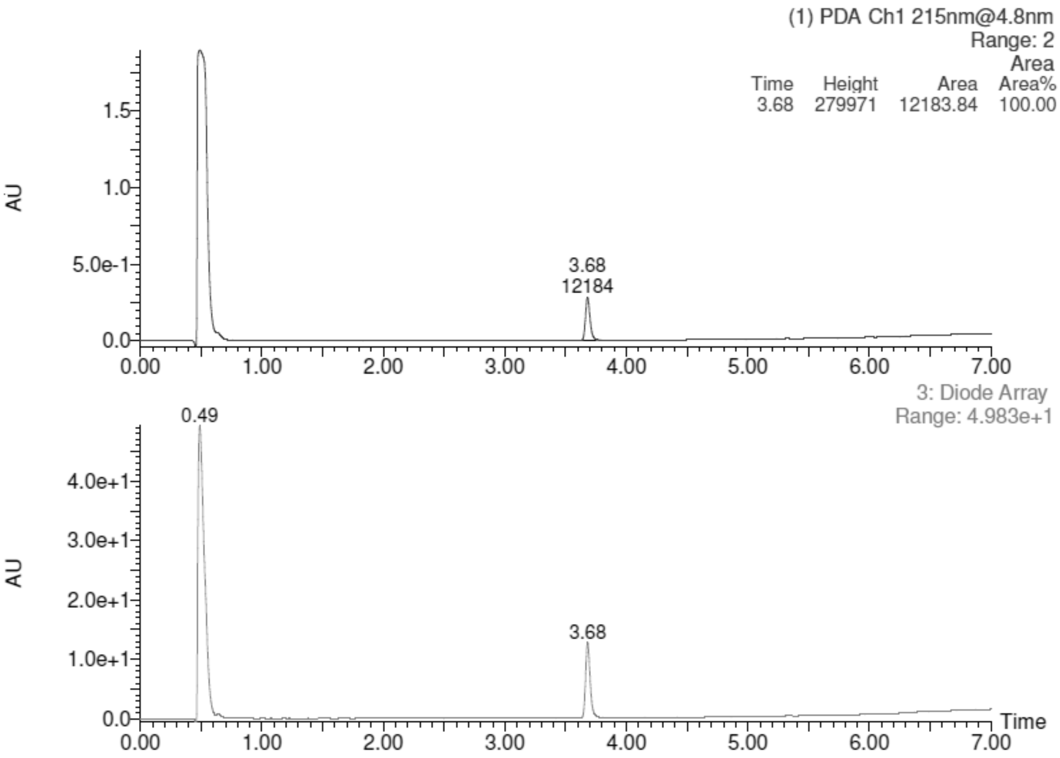

Compound **23j**

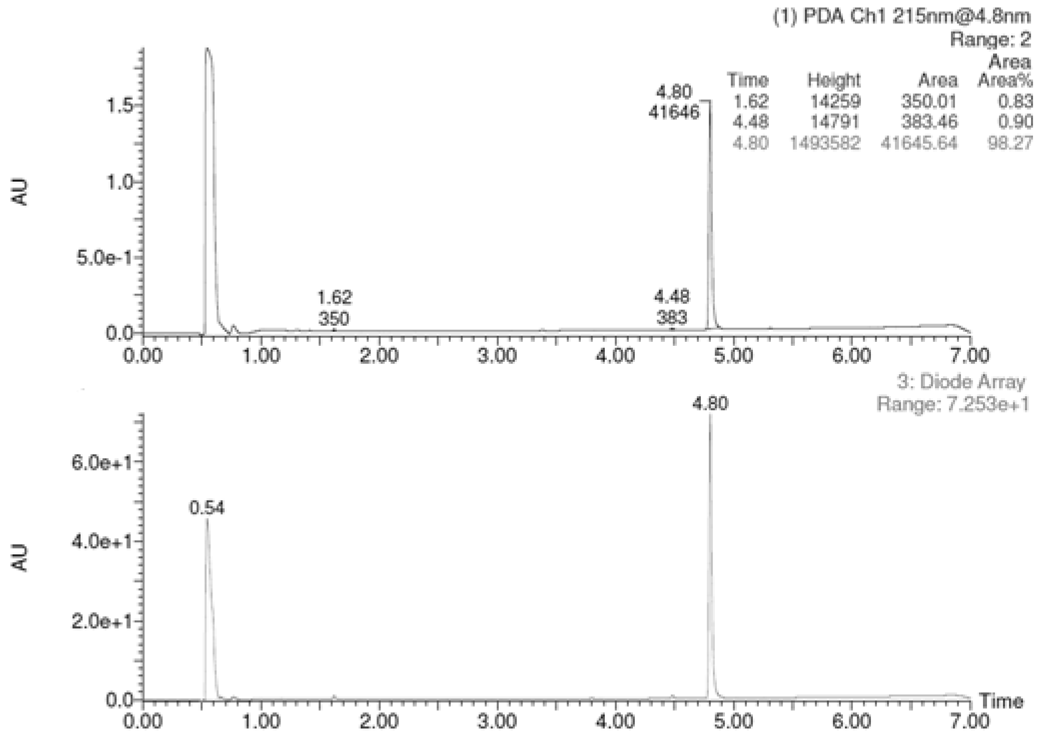

Compound **23k**

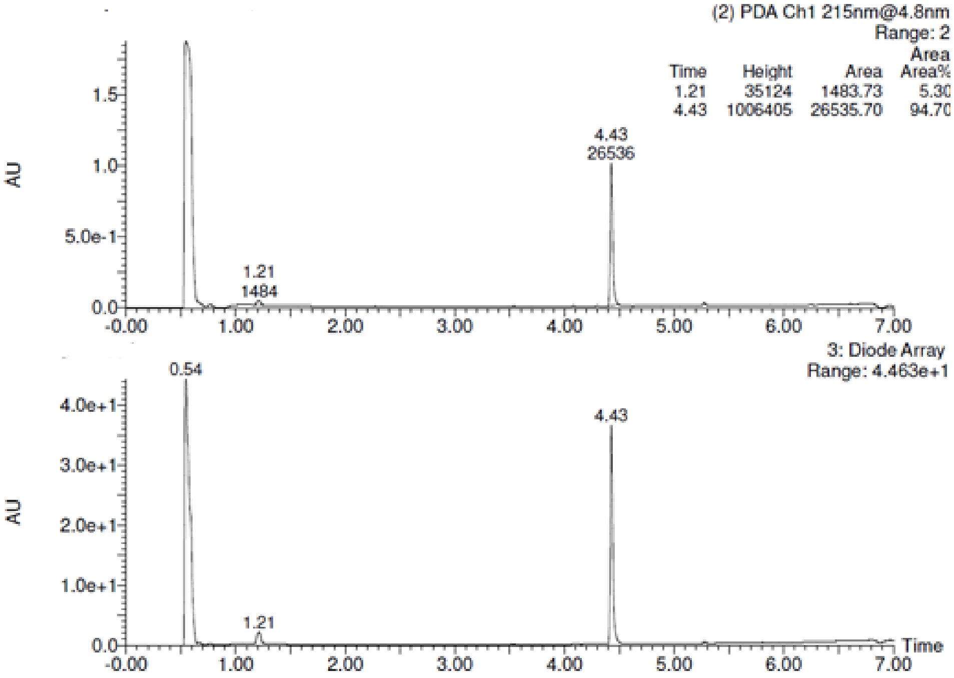

Compound **24c**

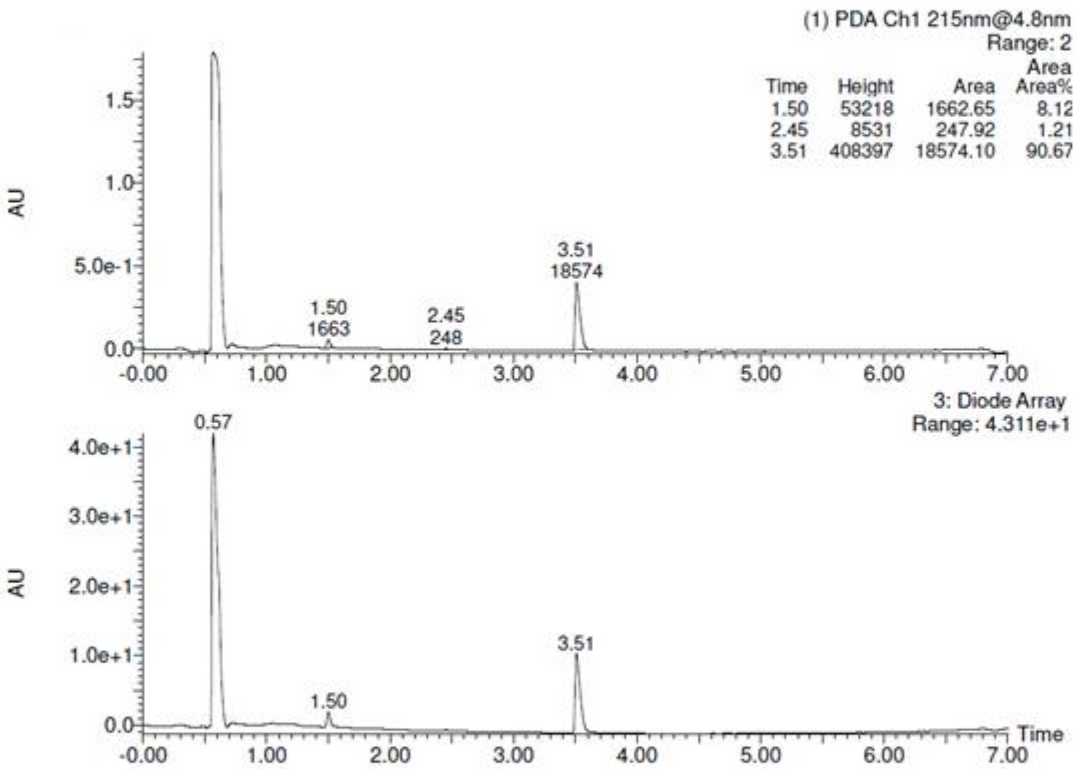

Compound **25**

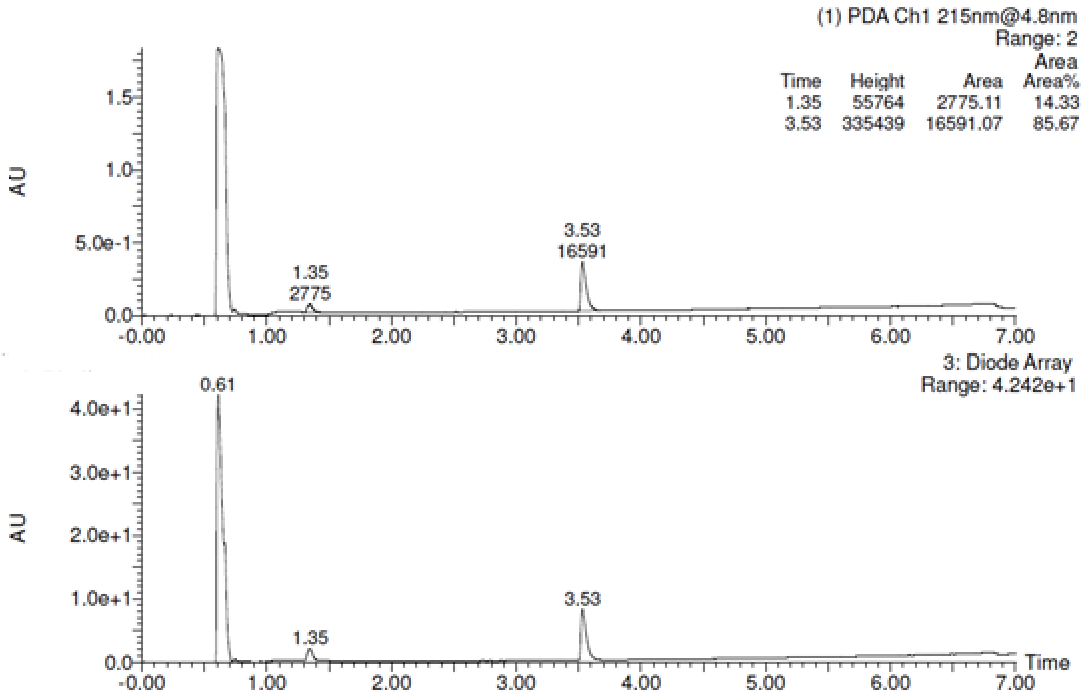

Compound **26**

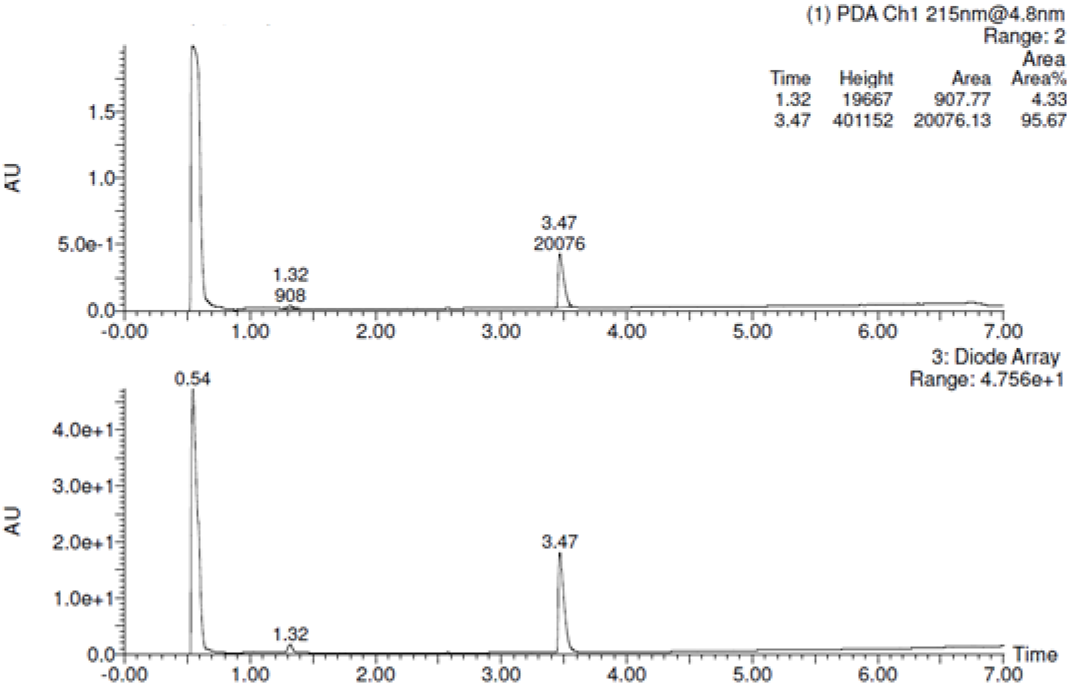

Compound **27**

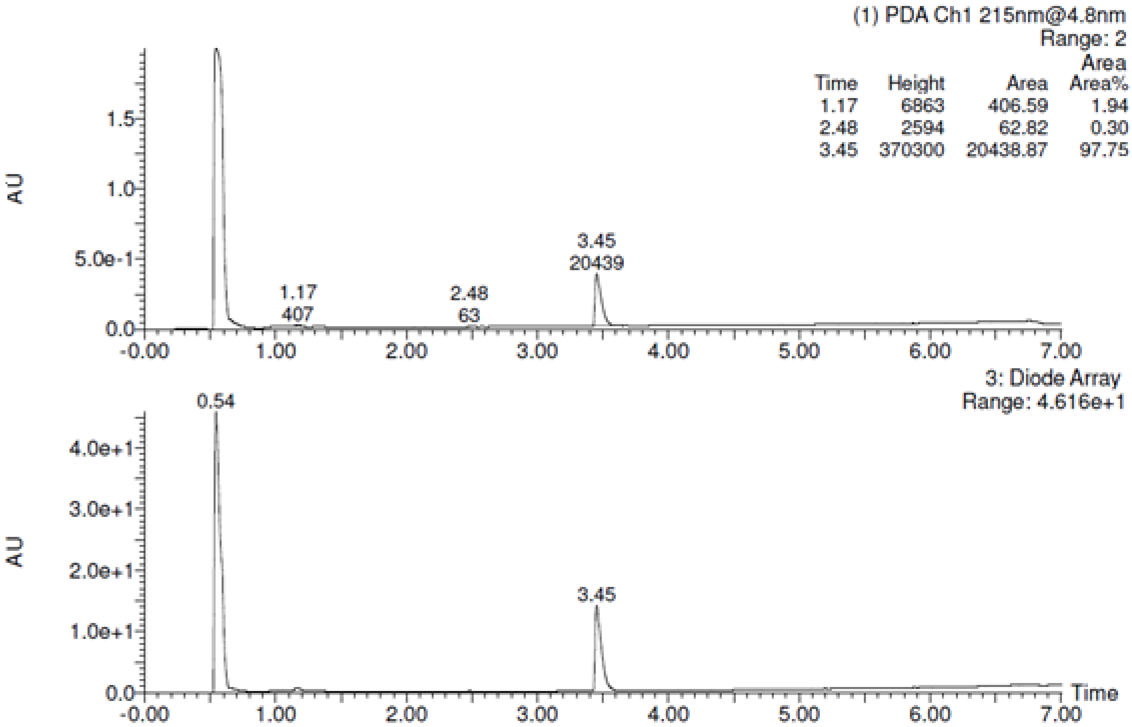

Supplement: Supplementary file 1 — jm9b02004_si_001.pdf [file jm9b02004_si_001.pdf]
